# Supplementary material for: Integrating adaptation pathways and Ostrom’s framework for sustainable governance of social-ecological systems in a changing world
Source: PeerJ. 2025 Feb 24;13:e18938. doi: 10.7717/peerj.18938 (PMC11867035; doi:10.7717/peerj.18938)
Supplement: Supplemental Information 1 — This compressed file contains: (1) A spreadsheet Presenting the details of the 4th tier SESF analysis, (2) A pdf file of the semi-structured interview guide that was used to obtain some of the SES variables. The unstructured part of the interviews contained identifiable personal information and thus was removed. It can be shared on demand only for actors who sign a sharing-consent agreement. (3) A pdf file in French language of the original M.S. report presenting elements of social-ecological analysis of the study site that was conducted during 2021. It includes the analysis of local and regional gray literature and anonymized interviews with some local stakeholders. This data represent one of the source of information for performing the socio-ecological system (SES) framework analysis across the two sites under investigation. The analysis of stakeholders encompassed both qualitative and quantitative data, facilitating an examination of their capacity to identify various types of hedgerows, their relationships with these features, preferences regarding ecosystem services associated with hedgerows, and their inclinations toward different governance arrangements and developmental trajectories. Details regarding the survey methodology and utilized documents are provided within the document. The Author of the report is Elise Krief, M.S. [file peerj-13-18938-s001.zip › Interview.Guide.English.pdf]

(Semi-)Structured Interview Guide

(the unstructured part of the interviews can be shared on demand for actors who signed the sharing-consent agreement)

I. Objectives of the guide

Objectives of the interview guide	Associated questions
Knowing and quantifying ecosystem services and disservices (ES+/ES-) of importance to the actor	2. a) b)<br>3.
Describe the current state in terms of hedgerow types (variables used for modeling) and the viability of this state for the actor	4. a) b)
Understand the actor's preferences regarding hedgerow types + their spatial distribution	5. 6. 7.
Assess the capacity and levers of action of the actor on the hedgerow network	8.
Probe the preferences of the actors among several land use	12. a) b)
Investigate the actions considered in relation to each scenario	12. c) d)
Identify perceived vulnerabilities and risks related to climate change on the territory	9. 10.
Identify the presence or absence of individual and collective adaptation strategies in response to these vulnerabilities	11.

II. Targetted Audience

Farmers
Elected officials of the municipalities (primarily La Sauvetat, Veyre-Monton, Saint-Saturnin, and Manglieu) and Mond'Arverne
Residents and associations (through field meetings and contacts)
Mission officers of the Territorial communities
NGO « Mission Haie Auvergne »
Conservatory of Natural Areas of Auvergne (Conservatoire des Espaces Naturels d'Auvergne)
NGO « Terre de Liens »

Agricultural Chamber of Puy-de-Dôme county/district (Chambre d'Agriculture du Puy-de-Dôme)

III. Materials Used

- Illustrated descriptive sheets presenting different ecosystem services and disservices
- Series of photos of hedges in the area, corresponding to the six variables of the modeling (no hedgerows, species rich, species poor, low, tall, punctuated, old, young, tree alignments)
- Maps of the selected sub-territories (optional)
- Quantitative scale of importance of ecosystem services and disservices
- Scale of presence by hedgerow type
- Illustrated descriptive sheets of different scenarios of land-use, climate change and collective action

IV. Série de questions

Introduction : Short presentation of the interview framework and the project.

- Research project to develop a tool for territorial decision-making support (in the form of a computer model) related to ecosystem adaptation to global change
- The study area is the Mond'Arverne Community of Communes, with four targeted municipalities
- Initially, testing of the modeling tool on hedgerows and an opportunity for a first contact with local actors, especially to address other themes related to the project (scenarios)

A. The ecosystem services of interest related to hedgerows and their min/max delivery

1. [Not for elected officials] Here are the four municipalities that we are studying as part of our project [*Show municipality maps*].
a) Can you tell me if you live, work, or regularly visit each of these areas? If yes, for what reasons? *[Identification of the connection to the study area(s)]*

[…]

b) [*If multiple answers*] For which use and which municipality do you feel most comfortable discussing hedgerow management?

[…]

The following questions will therefore focus exclusively on the municipality you just mentioned, in your capacity as [...].

2. I will present several sheets, each corresponding to a function fulfilled by hedges or nuisances [*show ES sheets*]. I let you read them.

a) Can you rank these features and drawbacks according to the importance you attribute to them? [*show the scale*]. *[dentification of important ES and their quantification]*

b) Which functions (ES) do you consider particularly important to preserve?

3. Are there any other functions provided by the hedgerows that are not included in these sheets? *[Search for a comprehensive view of ES+ and ES- of interest to the actor]*

4. I will now show you a series of photos representing different types of hedgerows [*Show the photos and explain the different types of hedgerows*].

a) Among these photos, which ones are closest to the hedgerow network you can observe in the municipality of [...] or Mond'Arverne? Do these types of hedgerows seem satisfactory to you?

[...]

b) Do you think it allows for the functions related to hedges that you consider to be the most important? *[State them again, maximum 3 ES].*
[Survey of the viability of the current state]

[…]

5. Now, can you set aside the photos that do not reflect your preferences at all regarding hedgerows, and leave those that you find satisfactory?
[Survey of unacceptable states]

[…]

6. For the remaining photos, can you rank them in order of your preferences? *[Minimum and maximum bounds of the ES satisfaction space variables]*

7. What would be the ideal distribution of the different types of hedgerows in the municipality, excluding urban areas? [Classify using the presence/absence scale]
[Minimum and maximum bounds of the hedgerow state variables]

8. Do you feel capable of acting to ensure the functions related to hedges that we have discussed? If yes, how? If no, why?
[Control space variables]

B. Vulnerabilities and adaptation strategies to climate change :

9. Do you think your territory is vulnerable to climate change? If yes, what events or observations lead you to believe this?
[Perception of current risks]

10. What future risks related to climate change do you anticipate at the territorial level?

[Perception of future risks]

11. In response to these risks, would you take any specific actions on a personal level? If yes, what? What would you like to see implemented or be ready to participate to at a collective level?
[Adaptation strategies]

C. Land-Use Scenarios

12. Here are several land use scenarios based on several institutional documents from the Mond'Arverne Community of Communes [show the scenario sheets and explain them one by one, with the data sources].

a) Among them, are there any scenarios that you would not want to see realized in the territorial?

b) Which one, or ones, do you consider to be the most important to implement? Why?

c) What individual and collective actions do you consider a priority to implement to achieve this/these scenario(s)?

d) What individual and collective actions do you think can address multiple scenarios at the same time?
