# Supplementary material for: Integrating adaptation pathways and Ostrom’s framework for sustainable governance of social-ecological systems in a changing world
Source: PeerJ. 2025 Feb 24;13:e18938. doi: 10.7717/peerj.18938 (PMC11867035; doi:10.7717/peerj.18938)

Mémoire de Master 2

Gestion des Territoires et Développement Local, parcours Dynamique Territoriale, Agriculture et Aménagement Rural

LES INFRASTRUCTURES AGROÉCOLOGIQUES DANS LES STRATÉGIES D'ADAPTATION AU DÉRÈGLEMENT CLIMATIQUE

Le cas des haies dans une intercommunalité périurbaine du Puy-de-Dôme

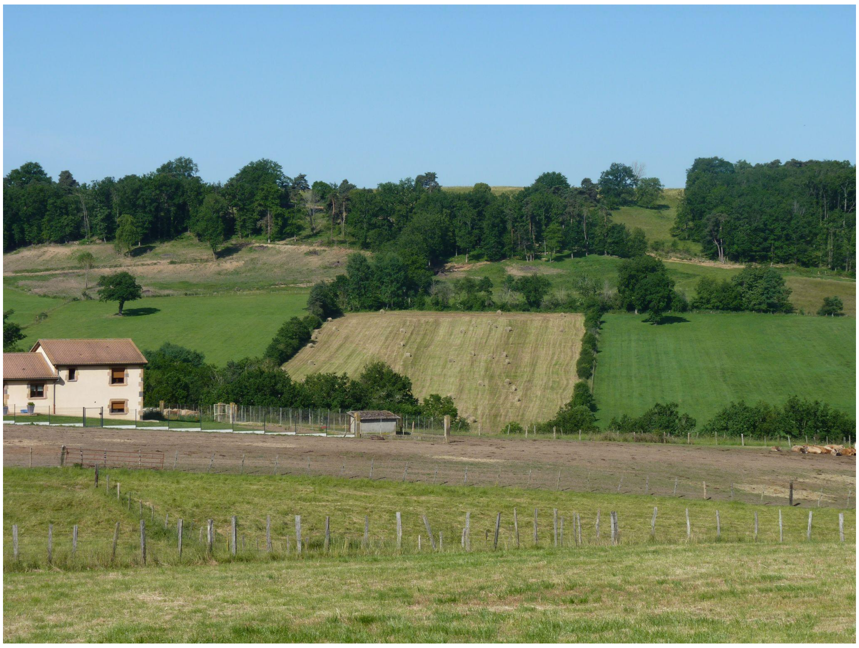

Elise KRIEF

Tuteur universitaire : Karim BERTHOMÉ

Tuteurs professionnels : Jean-Baptiste PICHANCOURT, Antoine BRIAS, Anne BONIS

Stage effectué au Laboratoire d'Ingénierie des Systèmes Complexes (LISC-INRAE)
Du 06 avril 2021 au 30 septembre 2021
Année universitaire 2020/2021

Remerciements

Je souhaite remercier en premier lieu mes tuteurs et tutrice de stage pour la grande confiance accordée et leur disponibilité : Jean-Baptiste Pichancourt pour les nombreux échanges instructifs, Antoine Brias pour sa pédagogie à multiples reprises concernant la modélisation, et Anne Bonis pour avoir toujours été de bon conseil dans l'organisation et la conduite des entretiens.

J'adresse des remerciements à Lisa Minaca, pour ses conseils, son écoute, les sorties sur le terrain, et qui a été un binôme idéal pour ce stage de fin d'études.

Je remercie mon tuteur pédagogique Karim Berthomé, pour sa grande disponibilité durant ce stage et ses nombreux conseils qui ont été déterminants dans l'avancée du mémoire.

Les conditions d'accueil dont j'ai bénéficié ont largement contribué au bon déroulement de ce stage : un grand merci à tout le Laboratoire d'Ingénierie des Systèmes Complexes de m'avoir accueillie pendant ces six mois, et en particulier à Sylvie Huet pour son soutien dans le cadre du projet.

J'exprime ma gratitude à toutes les personnes qui m'ont aidée de près ou de loin, en me permettant de tester mes entretiens, en transmettant leur passion pour les SIG, ou tout simplement en partageant leurs réflexions : Pauline, Gwénaëlle, Perrine, Coline, Lili, Florian, Merveille, Rémi, Méri. Je souhaite également remercier les personnes qui m'ont accordé du temps lors des entretiens ou sur le terrain, et pour la richesse de chacun des échanges.

Enfin, j'adresse des remerciements particuliers à Manon Soupizon et Rik Verhoeve, qui m'ont accompagnée du début de mon stage à la fin de la rédaction de ce mémoire, et ce au quotidien.

Mots clés analytiques : adaptation, services écosystémiques, infrastructures agroécologiques, haies, changement climatique, système socio-écologique, périurbain, développement territorial.

Analytical keywords : adaptation, ecosystem services, semi-natural infrastructures, hedgerows, climate change, socio-ecological system, peri-urban area, territorial development.

Mots clés géographiques / geographical keywords : Mond'Arverne Communauté, Puy-de-Dôme, Auvergne-Rhône-Alpes, France.

Résumé

Les pressions anthropiques effectuées sur les écosystèmes mettent en péril la fourniture d'une multitude de services écosystémiques. Dans un contexte de changements globaux, le maintien de ces services pose la question de l'usage des sols et de la place allouée aux infrastructures agroécologiques (IAE) qui sont pourvoyeuses de services. Ce mémoire a été rédigé dans le cadre du projet de recherche PACSEN, dont l'objectif est de modéliser différentes trajectoires d'usage des terres ainsi que les services écosystémiques associés à chacune d'elles. Notre travail s'attache plus particulièrement à chercher quelle peut être la place des infrastructures agroécologiques dans les stratégies d'adaptation au dérèglement climatique, en se concentrant sur une IAE, la haie, et en choisissant pour territoire d'étude une intercommunalité périurbaine du Puy-de-Dôme. La conduite de plusieurs entretiens permet d'alimenter cette réflexion à deux échelles - celle d'une IAE et celle d'un territoire.

Abstract

Anthropic pressure on ecosystems endangers the provision of a multitude of ecosystem services. In a context of global change, the preservation of those services raises the question of land use such as the place allowed to agroecological infrastructures, which provide ecosystem services. This dissertation takes place within the research project PACSEN, whose aim is to model a couple of land use paths, as well as the related ecosystem services. More specifically, our work tends to investigate what could be the place of agroecological infrastructures within local adaptation strategies to climate change. We chose to focus on a particular agroecological infrastructure, hedgerow, and on a study area, a peri-urban inter-municipality. The reflection is based on a literature review and on several interviews with local stakeholders.

Liste des abréviations

AFB : Agence Française de la Biodiversité

BCAE : Bonnes Conditions Agricoles et Environnementales

CEN : Conservatoire des Espaces Naturels

DATAR : Délégation à l'Aménagement du Territoire et à l'Action Régionale

DRAAF : Direction Régionale de l'Agriculture, de l'Alimentation et de la Forêt

DREAL : Direction Régionale de l'Environnement, de l'Aménagement et du Logement

EPCI : Établissement Public de Coopération Intercommunale

GIEC : Groupe Intergouvernemental d'Experts sur l'Evolution du Climat

IAE : Infrastructures agroécologiques

LPO : Ligue de Protection des Oiseaux

MAEC : Mesures agro-environnementales et climatiques

NI : Infrastructures naturelles

PAC : Politique Agricole Commune

PAEC : Plan agro-environnemental et climatique

PCAET : Plan Climat Air Énergie Territorial

PLH : Plan Local de l'Habitat

PLUi : Plan Local d'Urbanisme Intercommunal

PNR : Parc Naturel Régional

SAGE : Schéma d'Aménagement et de Gestion des Eaux

SDAGE : Schéma Départemental d'Aménagement et de Gestion des Eaux

SE : Services écosystémiques

SES : Système socio-écologique

SMVVA : Syndicat Mixte des Vallées de la Veyre et de l'Auzon

SNI : Infrastructures semi-naturelles

SRADDET : Schéma Régional de l'Aménagement, du Développement Durable et de l'Egalité des Territoires

SRCAE : Schéma Régional Climat Air Energie

TVB : Trame Verte et Bleue

ZNIEFF : Zone Naturelle à Intérêt Floristique et Faunistique

Table des matières

Remerciements 3

Liste des abréviations 5

Table des figures 8

INTRODUCTION 10

1. CONTEXTE DU STAGE ET CADRES D'ANALYSE POUR L'ÉTUDE D'UN TERRITOIRE PÉRIURBAIN 13

1.1. Présentation du projet de recherche et de ses cadres d'analyse 13

1.1.1. Le projet PACSEN – Volet écologie 13
1.1.2. Cadre d'analyse historique des systèmes socio-écologiques 16
1.1.3. Cadre d'analyse contemporain des systèmes socio-écologiques 17
1.1.4. Contraintes de modélisation sur le cadre d'analyse SES 21
1.1.5. Déroulé des missions de stage 23

1.2. L'étude d'un territoire périurbain : Mond'Arverne Communauté 24

1.2.1. Un territoire institutionnel récent 24
1.2.2. Un territoire structuré par plusieurs entités paysagères 25
1.2.3. Les grands enjeux territoriaux 27

1.3. Apports théoriques complémentaires du mémoire 29

1.3.1. Choix analytiques préliminaires 29
1.3.2. Adapter les territoires au dérèglement climatique : un enjeu alliant gouvernance prospective et gestion des conflits d'usage 31

2. MÉTHODES D'ENQUÊTE : ANALYSE BIBLIOGRAPHIQUE ET CONDUITE D'ENTRETIENS 39

2.1. Récolte de données quantitatives sur les haies et plusieurs services écosystémiques associés 39

2.1.1. Des choix méthodologiques déterminés par les contraintes de modélisation 39
2.1.2. L'élaboration du guide d'entretien 47

2.2. Récolte de données qualitatives sur le positionnement d'acteurs au regard de scénarios territoriaux plus larges 55

2.2.1. Élaboration de scénarios d'usage des terres et du guide d'entretien 57
2.2.2. Élaboration d'une grille de traitement et d'analyse des données 60

2.3. Points de vigilance concernant l'analyse des résultats 62

2.3.1. Des difficultés d'ordre pratique 62
2.3.2. Des difficultés d'ordre méthodologique 63

3. ANALYSE DES RÉSULTATS 65

3.1. Les haies sur le territoire de Mond'Arverne : état des lieux et perspectives pour une infrastructure agroécologique multi-services 65

3.1.1. Un état des lieux des haies sur le territoire de Mond'Arverne : analyse par les services écosystémiques 65
3.1.2. Agir sur les haies et penser leur gestion : analyse par les acteurs en présence 71
3.1.3. La place des haies dans les stratégies d'adaptation de Mond'Arverne 78

3.2. Quelles stratégies d'adaptation pour Mond'Arverne ? 83

3.2.1. Les vulnérabilités liées au dérèglement climatique identifiées 84
3.2.2. Analyse des actions d'adaptation 86
3.2.3. Quelles trajectoires d'usage des terres pour favoriser l'adaptation ? 91
3.2.4. Appréhender les tensions et conflits liés à l'usage des terres : une prise en compte nécessaire 95

Conclusion 101

Bibliographie 104

Table des annexes 109

Table des figures

Figure 1. Représentation synthétique d'un système socio-écologique adapté au territoire de Mond'Arverne Communauté, d'après Resilience Alliance (2007). 16

Figure 2. Les services écosystémiques en lien avec les composantes du bien-être humain, extrait du MEA 2005. Source : MEA (2005). 17

Figure 3. Représentation générique d'un système socio-écologique dans un territoire périurbain d'après Anderies & Ostrom (2002). Réalisation : Jean-Baptiste Pichancourt (2021). 19

Figure 4. Cycle de maintien ou d'adaptation des infrastructures, et de leur relation avec les agents qui les composent. D'après le cadre d'analyse morphogénétique des systèmes sociaux et culturels développé par Margaret Archer (1995). Réalisation : Jean-Baptiste Pichancourt (2021). 20

Figure 5. Exemple d'actions et de contraintes de viabilité dans le cadre de la gestion de deux services écosystémiques : la production agricole et le maintien du sol. Réalisation : Antoine Brias (2021). 22

Figure 6. Les intercommunalités du Puy-de-Dôme. Source : Wikipédia. 25

Figure 7. Les six entités paysagères de Mond'Arverne Communauté. Source : Diagnostic PLUi Mond'Arverne Communauté, Even Conseil (2018). 26

Figure 8. Les principaux enjeux territoriaux de Mond'Arverne Communauté à travers quatre prismes : agriculture, milieux naturels, paysage, et dynamiques territoriales. 28

Figure 9. Schéma représentant la multi-fonctionnalité des haies sur un bassin versant. Source : Le Guillou, 2019, p. 37. 41

Figure 10. Localisation des quatre communes d'étude au sein de Mond'Arverne Communauté. 47

Figure 11. Importance moyenne des six services écosystémiques étudiés sur une jauge de 0 à 5. 65

Figure 12. La haie sur le territoire de Mond'Arverne Communauté : synthèse des principaux acteurs et cadres législatifs en présence. 73

Figure 13. Schéma conceptuel représentant la complexité de l'aspect réglementaire de la haie. Source : Le Guillou, 2019, p. 91. 75

Figure 14. Les leviers d'action sur les haies ressortis lors des entretiens. 76

Figure 15. Les freins à l'action sur les haies relevés par les acteurs en entretien. 77

Figure 16. Les services écosystémiques les plus impactés par le dérèglement climatique sur le territoire de Mond'Arverne, d'après une série d'entretiens. 85

Figure 17. Cartographie des d'adaptation recensées sur le territoire de Mond'Arverne. 86

Figure 18. Les principales tensions liées à l'usage des terres à l'échelle d'une intercommunalité, d'après une série d'entretiens. 97

INTRODUCTION

Les activités anthropiques créent de multiples pressions sur les écosystèmes, impactant de manière différenciée les sociétés humaines. Ces pressions ont lieu dans un contexte de changements globaux, à savoir « *d'un ensemble de changements induits dans la dynamique de la biosphère par les activités humaines, directement ou non […]. Changement climatique, pollution des eaux et des sols, changements d'usage des terres, déforestation, surexploitation des ressources renouvelables, relèvent du changement global [...]* » (Trommetter & Weber, 2004, p. 137). Parce que ces changements se produisent de manière plus rapide et intense, une attention croissante est portée au concept d'adaptation des sociétés humaines, aussi bien dans les sphères scientifiques que politiques (Simonet, 2010). Considérant que les efforts d'atténuation des changements globaux n'ont pas permis de les endiguer, la capacité des sociétés à s'adapter, c'est-à-dire à « *ajuster les systèmes naturels ou humains en réponse à des stimuli [climatiques] présents ou futurs ou à leurs effets, afin d'atténuer les effets néfastes ou d'exploiter des opportunités bénéfiques* » (GIEC, 2014), paraît déterminante.

En effet, les impacts de ces changements peuvent être particulièrement négatifs pour les sociétés humaines, notamment parce qu'ils viennent altérer les systèmes écologiques. Les écosystèmes fournissent de multiples services, dits services écosystémiques, qui sont importants, voire indispensables, au bien-être humain. En modifiant plus ou moins intensément la biodiversité et les habitats naturels, les activités humaines ont donc une influence sur la fourniture qualitative et quantitative de services écosystémiques. Dans ce contexte, les infrastructures agroécologiques (IAE) ont une place importante. Il s'agit « *de tout habitat d'un agroécosystème dans ou autour duquel se développe une végétation spontanée essentiellement composée d'espèces bisannuelles, pluri-annuelles ou pérennes […]. Elles peuvent avoir diverses formes : (i) linéaire comme les alignements d'arbres et leurs bandes herbeuses [...] ; (ii) surfacique comme les prairies inondables, prés-vergers […], (iii) ponctuelle comme les mares, sources, arbres isolés, rochers […]* » (Sarthou, 2016). Au regard des pressions environnementales énoncées ci-dessus, les IAE semblent alors

présenter un double enjeu. Non seulement leur conservation paraît indispensable pour pérenniser les services qu'elles rendent aux sociétés humaines, mais leur capacité à atténuer les impacts négatifs de ces pressions en font des ressources clés à mobiliser, dans une perspective d'adaptation aux changements globaux.

Si la préservation de ces infrastructures agroécologiques est donc requise pour répondre à ces deux enjeux, elle peut toutefois entrer en tension avec d'autres préoccupations des territoires ruraux et périurbains. La relocalisation d'activités économiques, notamment industrielles, la pression démographique, ou encore la production d'une alimentation locale et diversifiée, sont autant de problématiques qui mettent en débat la conservation de ces infrastructures. Par conséquent, les territoires font face à des arbitrages permanents en matière d'occupation et d'usage des sols, notamment par le biais de leurs démarches de planification territoriale. Néanmoins, la question de l'adaptation reste encore peu abordée et détaillée de façon opérationnelle dans les documents programmatiques, même si les enjeux liés au dérèglement climatique et à l'érosion de la biodiversité y sont systématiquement évoqués.

Ce mémoire constitue l'aboutissement d'un stage de Master 2 Gestion des Territoires et Développement Local effectué au sein d'un projet de recherche pluridisciplinaire. En s'intéressant à une infrastructure agroécologique particulière, les haies, ce dernier cherche à modéliser la fourniture de plusieurs services écosystémiques selon différentes trajectoires d'usage des terres. Notre travail s'attache plus précisément à répondre à la question suivante :

Quelle place peuvent prendre les infrastructures agro-écologiques, en particulier les haies, dans les stratégies d'adaptation des territoires périurbains, notamment au dérèglement climatique ?

Le territoire étudié pour ce travail est celui de Mond'Arverne Communauté, une intercommunalité périurbaine et rurale située au sud de la métropole Clermont Auvergne. Dans un premier temps, nous présenterons plus en détail le projet de recherche, les missions effectuées au sein de celui-ci, ainsi que les cadres d'analyse mobilisés. Nous dresserons également un portrait du territoire d'étude et de ses principaux enjeux. Par la suite, nous reviendrons sur les méthodes de recueil de données utilisées, à savoir des entretiens semi-directifs ainsi qu'une analyse bibliographique des documents programmatiques du

territoire. Enfin, nous présenterons les résultats obtenus qui s'articulent autour de deux dimensions : l'une centrée sur une infrastructure agroécologique particulière, la haie, et l'autre plus large, à l'échelle du territoire d'étude et de ses choix d'utilisation des terres.

1. CONTEXTE DU STAGE ET CADRES D’ANALYSE POUR L’ÉTUDE D’UN TERRITOIRE PÉRIURBAIN

1.1. Présentation du projet de recherche et de ses cadres d’analyse

1.1.1. Le projet PACSEN – Volet écologie

Le présent mémoire est la contribution de mon stage de Master 2 Gestion des Territoires et Développement Local au projet de recherche PACSEN, qui est composé de trois volets : écologie, médias/communication et littérature. J’ai intégré pendant six mois l’équipe de scientifiques du volet écologie qui associe le Laboratoire d’Ingénierie sur les Systèmes Complexes (LISC-INRAE) et l’UMR GEOLAB (UCA et CNRS), conférant au projet une double compétence en modélisation socio-écologique et en écologie. Lors de mon stage, l’équipe était composée de deux responsables scientifiques : Anne Bonis, chargée de recherche en écologie au CNRS et Jean-Baptiste Pichancourt, chargé de recherche au LISC ; d’Antoine Brias, post-doctorant en mathématiques-informatique recruté sur toute la durée du projet ; de Lisa Minaca, stagiaire au GEOLAB en Master 2 Man & Biosphere, et de moi-même.

PACSEN se décline sur deux ans et a été proposé dans le cadre de l’appel à projets Contrats de Plan Etat-Région (CPER) 2015-2020. Il vise à remédier au constat suivant : la faible conscience des populations vis-à-vis des risques naturels et environnementaux présents à l’échelle de leur territoire de vie, ainsi qu’un partage limité des informations et des données susceptibles d’orienter les prises de décision. Cet état de fait exacerbe les vulnérabilités

socio-économiques des différentes populations, notamment dans un contexte de changements globaux où les écosystèmes font l'objet de multiples pressions (MEA, 2005). En d'autres termes, PACSEN est dédié « *aux enjeux d'appropriation et d'évaluation des risques naturels et environnementaux par les sociétés et les individus, et à la proposition d'outils pour accompagner la réflexion collective vers des solutions d'adaptation à ces périls* » (Résumé du projet PACSEN, 2020, p. 1). Au bout du compte, le projet a vocation à produire un cadre méthodologique et d'analyse pour l'élaboration d'un outil d'aide à la décision, en mesure de promouvoir « *la prise de conscience des effets liés à la réduction des ressources naturelles et de la réduction de la biodiversité, et l'élaboration de solutions accroissant la résilience socio-écologique* » (Document de présentation PACSEN CPER Auvergne-Rhône-Alpes, 2020, p. 5).

En ce qui concerne plus précisément le volet écologie, les trois grands objectifs sont les suivants :

1) Établir un état des lieux des éléments structurels et dynamiques du système socio-écologique du territoire d'étude

- En évaluant les services écosystémiques (voir *infra*) présents sur le territoire d'étude : par la description du contexte biophysique et institutionnel, ainsi que par l'évaluation des principaux risques qui pèsent sur la fourniture de ces services ;
- En analysant les variables institutionnelles d'intérêt (voir *infra*) et les points critiques du système socio-écologique pour en déduire si la gouvernance à long terme de la ressource et des services associés est robuste ;
- En modélisant et projetant les conséquences de ces choix de gouvernance sur la dynamique des services écosystémiques en réponse à des pressions environnementales.

2) Proposer des scénarios d'adaptation pour aider à viabiliser sur le long terme un ensemble de services écosystémiques

- En identifiant les principales contraintes des acteurs[1] sur leur environnement biophysique ;
- En identifiant les besoins/attendus des acteurs au regard des différents services écosystémiques possibles ;
- En évaluant leur capacité d'action individuelle et collective sur le maintien de ces différents services ;
- En créant des « cartes de gouvernance » représentant l'ensemble des trajectoires possibles d'adaptation des pratiques et infrastructures pour permettre de maintenir la viabilité d'un ensemble de services écosystémiques, et ce malgré les aléas naturels et environnementaux.

3) Développer une première preuve de concept d'un outil d'aide à la décision combinant les résultats de l'état des lieux et les scénarios d'adaptation

Des cadres d'analyse définis

Afin de répondre aux objectifs précédemment cités, plusieurs cadres d'analyse sont mobilisés au sein du projet de recherche. Commençons par préciser que le projet s'inscrit dans un contexte et une perspective pluridisciplinaires, mobilisant des apports théoriques issus des sciences écologiques, décisionnelles et sociales. Ainsi, les différents cadres d'analyse utilisés découlent de ce souhait. Mond'Arverne Communauté, le territoire choisi pour l'étude, est analysé sous l'angle de la théorie des *systèmes socio-écologique* ou *SES* (Liu et *al*., 2007), c'est-à-dire comme un système impliquant des interactions entre, d'un côté, un système social (acteurs, structures sociales et institutionnelles, normes socio-culturelles) et de l'autre, un système écologique (organismes et espèces, structures écologiques, lois de la nature).

[1] Dans ce mémoire, le terme « acteurs » est utilisé, pour faciliter la lecture, à titre épicène.

1.1.2. Cadre d'analyse historique des systèmes socio-écologiques

Ces deux systèmes n'évoluent pas de manière cloisonnée, et leurs composantes interagissent pour former un système socio-écologique. D'un côté, les acteurs bénéficient de ce que l'on appelle des *services écosystémiques* (SE), et de l'autre, ils et elles effectuent des actions d'intervention pour gérer les écosystèmes.

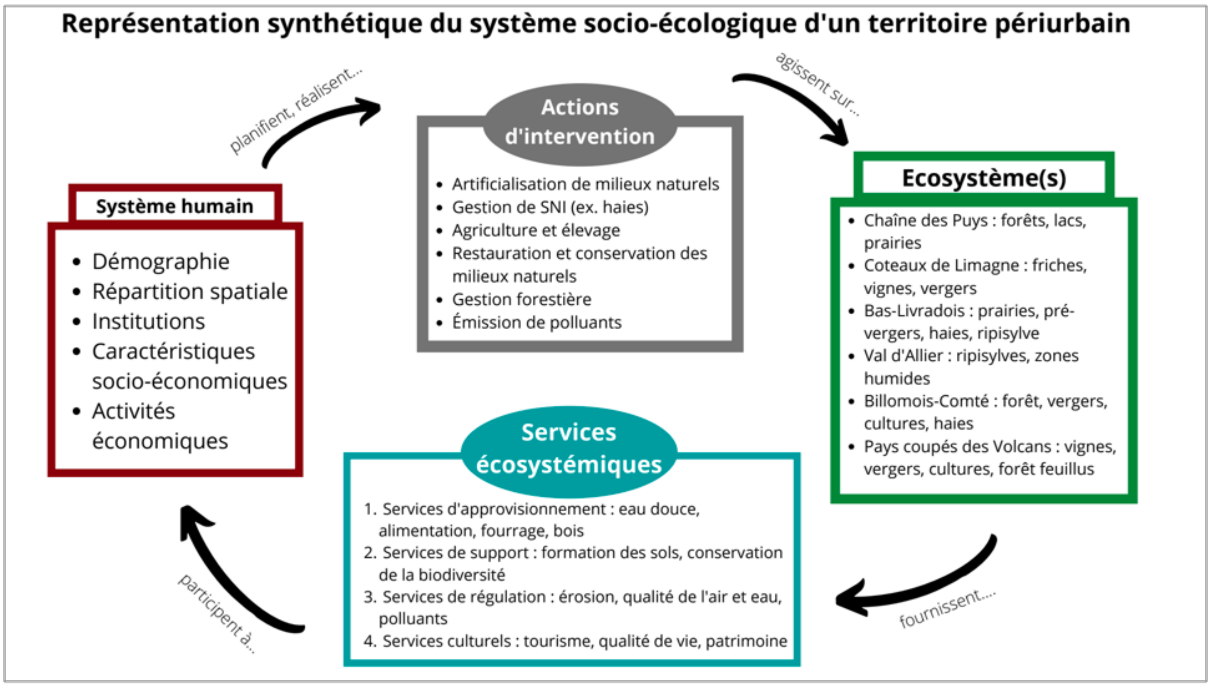

Figure 1. Représentation synthétique d'un système socio-écologique adapté au territoire de Mond'Arverne Communauté, d'après Resilience Alliance (2007).

Ce concept de SES, ainsi que sa représentation simplifiée, ont été particulièrement mis en avant avec le Millenium Ecosystem Assessment (MEA) (2005), et permet de mettre en lumière la dépendance des sociétés humaines aux écosystèmes, en cherchant à caractériser et à quantifier les bénéfices fournis par ces derniers, d'un point de vue écologique, culturel, et économique (Lamarque, 2012). Dans cette perspective, quatre familles de services écosystémiques sont identifiées : les services de support (e.g. la conservation de la biodiversité) ; les services d'approvisionnement (e.g. la production de fibres textiles ou alimentaires); les services de régulation (e.g. régulation du climat ou de l'érosion) ; et enfin les services culturels, tels que l'esthétisme ou les valeurs culturelles (MEA, 2005).

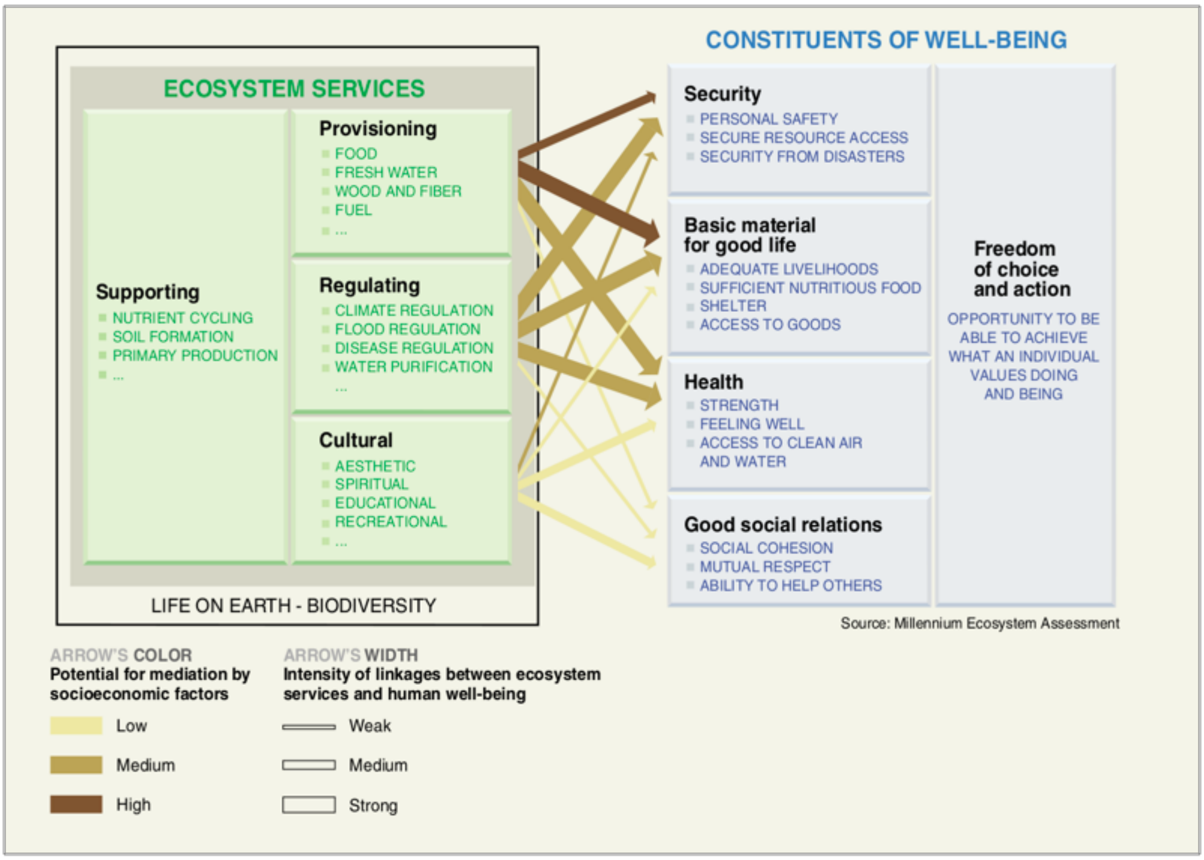

Figure 2. Les services écosystémiques en lien avec les composantes du bien-être humain, extrait du MEA 2005. Source : MEA (2005).

1.1.3. Cadre d'analyse contemporain des systèmes socio-écologiques[2]

Bien que cette représentation dichotomique soit relativement simple, elle ne permet pas d'analyser finement les raisons pour lesquelles des territoires se structurent en systèmes socio-écologiques, se maintiennent sur le long terme, ou bien perdent leur robustesse pour atteindre des états non désirés, d'un point de vue écologique et social. Par exemple, ce modèle de représentation ne nous dit rien des rôles différenciés entre utilisateurs de la ressource, instances de décision et organisations intermédiaires. Il ne dit pas non plus comment les relations entre acteurs, espèces, éléments culturels ou physiques s'organisent pour former, maintenir et transformer dans l'espace et le temps des structures essentielles au

[2] Ce point a été rédigé par Jean-Baptiste Pichancourt, chargé de recherche au Laboratoire d'Ingénierie des Systèmes Complexes (Institut National de Recherche pour l'Agriculture et l'Environnement).

maintien de l'approvisionnement en services écosystémiques. Il ne nous dit donc rien des principes d'organisation institutionnelle qui permettent la résilience et l'adaptation viable du SES. Pour pallier ces manques, des avancées récentes en écologie politique sur la gouvernance des SES ont été faites, initiées en particulier par l'école de pensée du prix Nobel d'économie Elinor Ostrom (2009). Ces avancées ont permis de proposer des représentations plus fonctionnelles qui, plutôt que de mettre l'accent sur la dichotomie entre social et écologique, s'intéressent à une analyse systémique et fonctionnelle composée d'infrastructures couplées qui forment la base de l'organisation et du fonctionnement des SES (Ostrom, 1990 ; Anderies & Ostrom, 2004 ; Anderies et *al*., 2019).

Ici, le terme d'infrastructure doit être compris en tant que réseau ou structure qui permet le fonctionnement et la circulation, par exemple de biens ou de personnes (on parle alors d'infrastructures physiques (« hard ») telles que les routes ou les chaînes de production). Mais de manière plus générale, il doit être compris comme permettant aussi le fonctionnement et la circulation du pouvoir et des instructions (infrastructure ou capital institutionnel : agences, associations…), des espèces végétales et animales (infrastructure ou capital écologique : forêts, haies, communautés végétales…), d'information, de connaissances et pratiques (infrastructure ou capital culturel), et enfin de relations sociales (infrastructure ou capital social)[3]. L'ensemble de ces infrastructures peuvent interagir et s'organiser en quatre groupes fonctionnels d'infrastructures pour former un système socio-écologique (voir figure 3). Ces quatre grandes classes d'infrastructures sont les suivantes : l'infrastructure de ressource, l'infrastructure utilisatrice de la ressource, l'infrastructure de gouvernance, et enfin l'infrastructure auxiliaire de support et de régulation de la ressource.

[3] Sur ce point, voir Anderies, Janssen & Schlager, 2016.

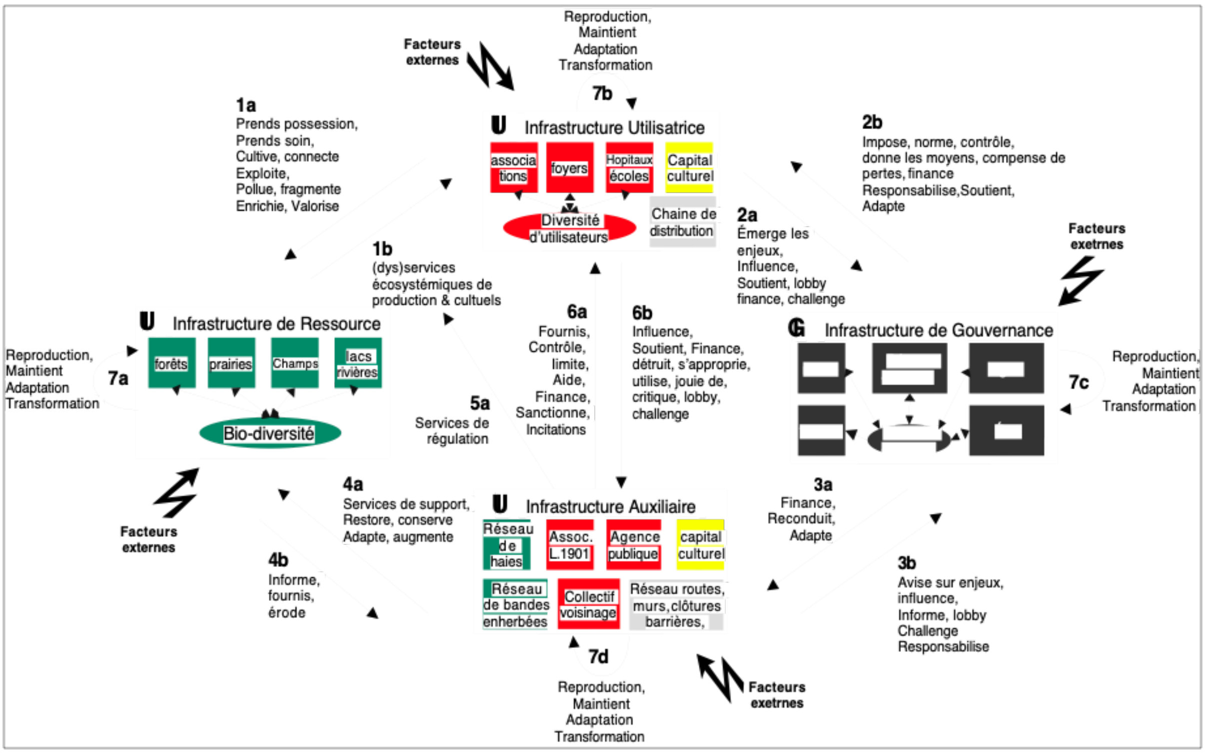

Figure 3. Représentation générique d'un système socio-écologique dans un territoire périurbain d'après Anderies & Ostrom (2002). Réalisation : Jean-Baptiste Pichancourt (2021).

L'infrastructure de ressource (boîte IR dans la figure 3) est tout d'abord composée d'unités de ressources (par exemple la biodiversité d'organismes ou d'espèces), organisées éventuellement en structures écologiques, telles que les populations et communautés d'espèces, et en écosystèmes tels que les forêts, prairies, lacs, rivières. Indépendamment de la présence humaine, ces infrastructures écologiques sont maintenues, défaites, ou s'adaptent dans le temps et l'espace par l'effet conjoint de lois écologiques (7a dans la figure 3) et des aléas environnementaux. Dans PACSEN, l'infrastructure de ressource inclut des structures écologiques comme les forêts, les prairies, les lacs, rivières et champs. Ces structures écologiques fournissent chacune des services finaux aux utilisateurs tels que les services de production (bois d'œuvre, nourriture) et culturels (esthétiques paysagères, connaissances).

L'infrastructure utilisatrice (IU dans la figure 3) est quant à elle composée d'une diversité d'utilisateurs de la ressource bénéficiant de services écosystémiques finaux. Ils peuvent s'organiser pour former des infrastructures socio-économiques d'extraction, de transformation et de distribution d'unités de ressource, pour en garantir l'approvisionnement

en services écosystémiques (par exemple des chaînes de distribution agricoles et forestières). Ils peuvent aussi s'organiser en associations, co-propriétés ou clubs.

L'infrastructure de gouvernance (IG dans la figure 3) est composée d'un ensemble d'acteurs gouvernant la gestion de la ressource et des services écosystémiques. Ces acteurs en position décisionnelle peuvent s'organiser en groupes spontanés ou en institutions plus durables. Leur rôle est de structurer les règles d'utilisation et de gestion de la ressource, de conditionner les utilisateurs de la ressource (2b), et de conditionner les acteurs de l'infrastructure auxiliaire décrite ci-dessous (2a).

L'infrastructure auxiliaire (IA dans la figure 3) est aussi composée d'acteurs, qui s'organisent éventuellement pour former des structures sociales élaborées dans l'optique de soutenir l'infrastructure de ressource (flèche 4a), d'en réguler leurs effets négatifs/positifs sur l'infrastructure utilisatrice (flèche 5a), ainsi que de conditionner les utilisateurs (flèches 6a) et les gouvernants (flèche 3b). Ces infrastructures auxiliaires se maintiennent, se reproduisent ou s'adaptent (flèche 7d) via les utilisateurs (6b), les gouvernants (3b) ou via l'impact des aléas naturels et environnementaux. Mais ces services de support et de régulation fournis par ces structures sociales peuvent en pratique se réaliser par l'intermédiaire d'autres infrastructures plus pérennes. Par exemple, les communautés locales créent des infrastructures auxiliaires de nature physique, tels que les réseaux routiers, pour améliorer l'accès à la ressource en forêt.

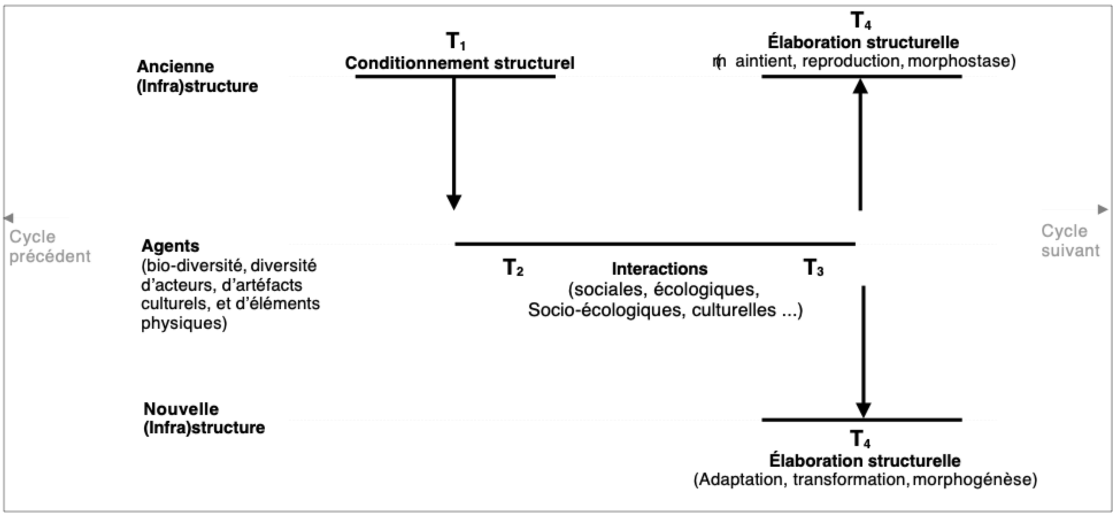

Figure 4. Cycle de maintien ou d'adaptation des infrastructures, et de leur relation avec les agents qui les composent. D'après le cadre d'analyse morphogénétique des systèmes sociaux et culturels développé par Margaret Archer (1995). Réalisation : Jean-Baptiste Pichancourt (2021).

PACSEN cherche à modéliser les différentes manières dont peuvent évoluer la fourniture de services écosystémiques en fonction de plusieurs trajectoires d'usage des terres. Les services étant fournis par des infrastructures naturelles (NI) (e.g. les forêts) et semi-naturelles (SNI) (e.g. les haies ou les prairies), leur gestion, abondance, et caractéristiques écologiques sont logiquement déterminantes au regard des bénéfices que les sociétés humaines pourront en retirer. Néanmoins, la qualité écologique des infrastructures (semi) naturelles est loin d'être le seul élément à prendre en compte lorsque l'on s'intéresse au concept de SE. En effet, les attendus des acteurs, notamment en fonction des groupes socio-professionnels auxquels ils et elles appartiennent, les normes sociales et culturelles en vigueur, ainsi que les enjeux propres au territoire concerné, sont autant de paramètres à intégrer dans notre analyse. Concernant cette dimension, Wallace (2007), précise que « *la capacité* [des écosystèmes à fournir des services] *n'est que potentielle, tant que ces services ne sont pas utilisés, consommés ou valorisés par des individus ou groupes humains* ».

1.1.4. Contraintes de modélisation sur le cadre d'analyse SES[4]

Afin d'offrir un outil d'aide à la décision proposant des solutions durables, il est nécessaire de connaître des informations quantitatives sur certains aspects de la modélisation. Les années 1990 ont vu l'émergence de la théorie de la viabilité. Celle-ci fournit un cadre mathématique pour la conservation des propriétés d'un système sur le long terme. Elle est dans son essence opposée aux approches classiques d'optimisation, dans lesquelles on cherche à minimiser ou maximiser une fonction d'intérêt sur un ensemble (par exemple : où planter tel type de haie pour maximiser un service écosystémique). Dans le cas de la viabilité, l'intérêt est focalisé sur la diversité des solutions qui s'offrent aux décideurs et aux acteurs pour conserver les propriétés d'un système (par exemple : quels choix d'action ont les acteurs pour assurer l'approvisionnement en un service écosystémique sur le long terme). Elle permet d'étudier un SES en termes de respect des contraintes de chacun, à chaque instant, plutôt que de partir à la recherche d'une solution optimale à partir d'un critère *a priori*. Cette différence de philosophie entre optimisation et viabilité explique l'engouement des scientifiques en

[4] Ce point a été rédigé par Antoine Brias, chercheur post-doctorant à l'Unité Mixte de Recherche GEOLAB (Université Clermont Auvergne et CNRS).

sciences environnementales et sociales à son égard, donnant lieu à de nombreuses applications. Afin d'utiliser cette théorie, il est nécessaire d'avoir des informations sur plusieurs variables et structures du modèle SES (voir exemple figure 5) :

- La dynamique du système : savoir comment le système va évoluer dans le temps, comment il réagit aux actions de gestion (quel impact de telle mesure sur les services écosystémiques fournis) ;
- Les actions possibles des acteurs : savoir quelle gamme d'actions les acteurs sont prêts à mettre en œuvre pour agir sur le système ;
- Les contraintes et préférences des acteurs : comprendre les attentes des acteurs vis-à-vis des services écosystémiques.

Les entretiens avec des acteurs locaux sont un premier pas pour avoir accès à ces informations. Le travail de modélisation consiste ensuite à les traduire en équations mathématiques pour utiliser les méthodes de viabilité.

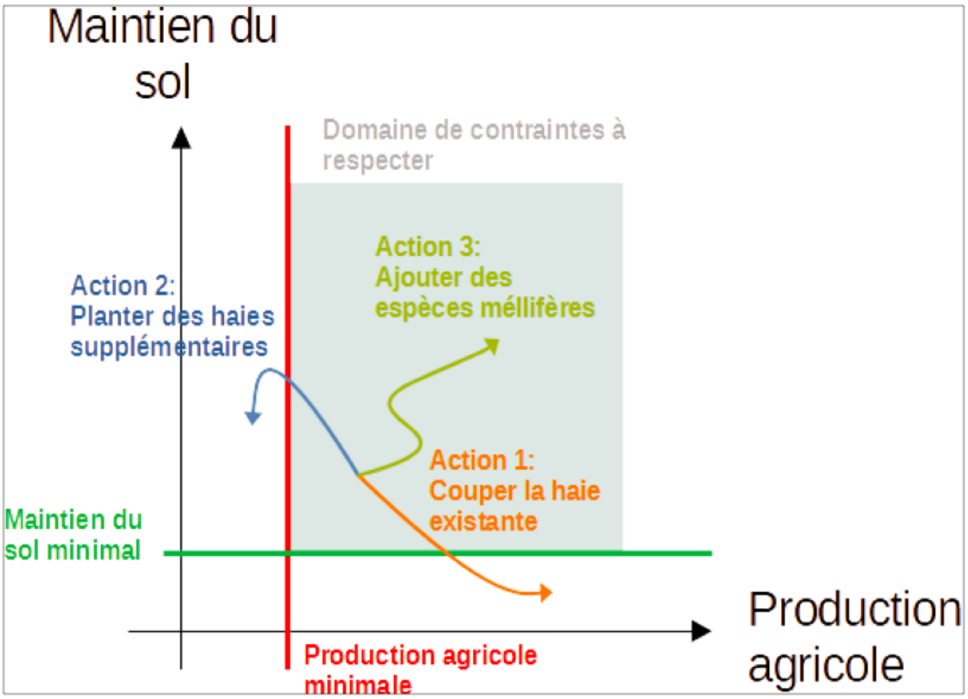

Figure 5. Exemple d'actions et de contraintes de viabilité dans le cadre de la gestion de deux services écosystémiques : la production agricole et le maintien du sol. Réalisation : Antoine Brias (2021).

Dans la figure 5, on suppose qu'un certain niveau de production agricole et de maintien du sol doit être respecté. Cela crée un domaine de contraintes dans lequel on cherche à maintenir l'état du système. Dans cet exemple, trois actions sont possibles et vont influencer la dynamique du système. Couper la haie permet de semer à la place et d'améliorer la production agricole au détriment du maintien du sol. Planter de nouvelles haies améliore le maintien du sol mais peut gêner les machines agricoles et diminuer le rendement de l'exploitation. Ajouter des espèces mellifères permet d'attirer des pollinisateurs et peut favoriser la production agricole tout en améliorant le réseau racinaire de la haie. Cette troisième action permet de maintenir les services écosystémiques dans le temps.

1.1.5. Déroulé des missions de stage

Le stage dans lequel ce travail s'inscrit comprenait à l'origine plusieurs missions, qui s'inscrivent dans les contraintes analytiques et de modélisation décrites ci-dessus. La première mission était de contribuer à la définition du système socio-écologique de notre territoire d'étude dans ses aspects sociaux. Cela impliquait donc d'établir un diagnostic des principaux enjeux, cadres législatifs et acteurs en présence, notamment par l'analyse de documents programmatiques territoriaux et extra-territoriaux. Par la suite, était surtout attendu l'échange avec plusieurs acteurs pertinents autour de services écosystémiques, de différents scénarios d'usage des terres (e.g. naturalisation, intensification de l'agriculture, urbanisation), afin d'obtenir des informations sur leurs contraintes, préférences et capacités d'adaptation vis-à-vis de ces derniers. Les modes de récolte de données envisagés étaient des entretiens individuels semi-directifs ainsi que des ateliers en petits collectifs autour de supports visuels.

Néanmoins, ces attentes initiales étaient trop précoces par rapport aux besoins, contraintes et temporalité du projet, ce qui a nécessité une révision des missions au bout de plusieurs semaines. Si le travail de diagnostic a pu être réalisé dès le début du stage, les ateliers n'étaient plus pertinents et des entretiens ont été réalisés dans un contexte différent (voir en deuxième partie). La première moitié du stage a alors été consacrée aux tâches suivantes :

- L'analyse bibliographique des différents matériaux à notre disposition[5] et que nous avons pu nous approprier[6] ;
- La co-construction de la problématique du projet avec l'équipe de recherche, notamment par la réalisation d'entretiens-test qui ont permis de définir collectivement le champ des possibles compte tenu des contraintes de la modélisation mathématique et de celles de la récolte de données par le biais d'entretiens semi-directifs ;
- L'élaboration de la méthode d'entretien, par la construction du guide et de la grille d'analyse, ainsi que de la définition des acteurs pertinents à solliciter.

La seconde moitié du stage fut dédiée à la conduite des entretiens, à leur retranscription et à leur analyse, ainsi qu'à la rédaction du mémoire de fin d'études. Les apports attendus étaient donc avant tout en matière de développement territorial (capacité d'analyse des enjeux d'un territoire, de ses acteurs et institutions) ainsi qu'en conduite et analyse d'entretiens en sciences sociales.

1.2. L'étude d'un territoire périurbain : Mond'Arverne Communauté

1.2.1. Un territoire institutionnel récent

Les travaux réalisés dans le cadre de PACSEN portent sur le territoire de Mond'Arverne Communauté, une Communauté de Communes située dans le Puy-de-Dôme, entre les agglomérations de Clermont-Ferrand et d'Issoire. Ce territoire fut créé en 2017 suite à la loi NOTRe de 2015 encourageant l'agrandissement des intercommunalités, et résulte donc de la fusion de trois anciennes Communautés de Communes (Les Cheires, Gergovie Val d'Allier et Allier Comté Communauté). Cette institution relativement nouvelle compte à présent plus de 40 400 administré·e·s et associe 27 communes.

---

[5] Les recherches effectuées par Magali Weissgerber, doctorante en géographie environnementale, portant sur le même territoire d'étude, ont été précieuses pour la définition des enjeux territoriaux.

[6] Via un échange téléphonique avec le pôle développement du territoire d'étude.

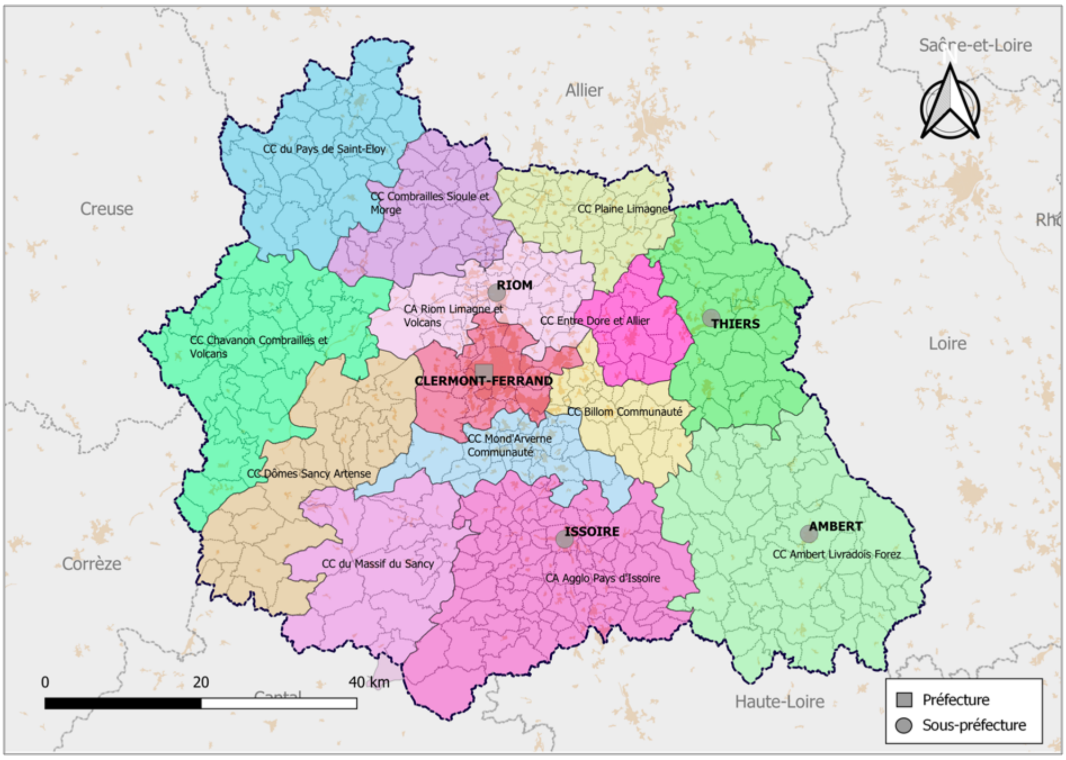

Figure 6. Les intercommunalités du Puy-de-Dôme. Source : Wikipédia.

Le choix de ce territoire a été fait pour plusieurs raisons. La première est sans doute le positionnement d'un autre projet de recherche (BIOECO) sur cet espace, qui permettait un accès facilité aux acteurs locaux et à tous renseignements. Magali Weissgerber, doctorante, et Amélie Menet, stagiaire, avaient d'ailleurs mené en 2019 une série d'entretiens et récolté des informations permettant d'appréhender les principaux enjeux territoriaux (voir annexe 1). En outre, Mond'Arverne Communauté est un territoire périurbain qui rencontre des problématiques propres à son type d'espaces, comme par exemple une difficulté à limiter l'étalement urbain et à préserver les habitats naturels et les corridors écologiques. Ainsi, les apports du projet PACSEN ont plus de chance d'être généralisables à d'autres territoires français. Ceci étant, l'intercommunalité présente de nombreuses spécificités géographiques qu'il s'agira de prendre en compte, d'autant qu'elle abrite en son sein une mosaïque de paysages très hétérogène.

1.2.2. Un territoire structuré par plusieurs entités paysagères

Plusieurs sous-ensembles sont à distinguer au sein de Mond'Arverne, avec des logiques d'occupation des sols différentes.

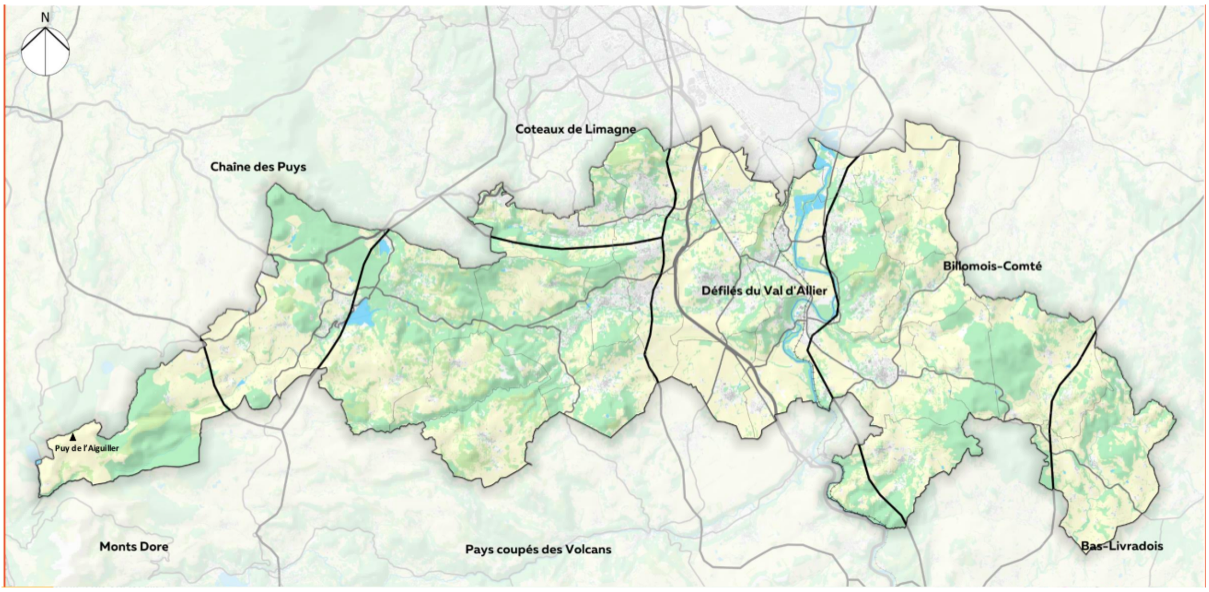

Figure 7. Les six entités paysagères de Mond'Arverne Communauté[7]. Source : Diagnostic PLUi Mond'Arverne Communauté, Even Conseil (2018).

Comme le montre la figure 7, le territoire présente au centre-ouest plusieurs zones urbanisées, très attractives par leur proximité et leur accessibilité avec la métropole clermontoise, qui correspondent à des zones de plaine et de vallons alluviaux. Sur ces parties, l'enjeu de limitation de l'étalement urbain est fort, tout comme son corollaire, celui de la préservation des espaces naturels et agricoles. De plus, règne sur ces espaces de façon majoritaire une activité agricole céréalière dite intensive, effectuée sur des parcelles de grande taille et travaillées en monoculture. En s'éloignant un peu vers l'ouest, l'on trouve des zones de coteaux, urbanisées et présentant des écosystèmes spécifiques comme des forêts thermophiles ou encore des friches s'installant sur des anciennes parcelles de vignes et vergers. Enfin, trois derniers sous-ensembles nous semblent importants à présenter, ceux de la Chaîne des Puys, du Billomois-Comté et du Bas-Livradois. A l'ouest (quasi exclusivement sur la commune d'Aydat), la forte présence de la forêt est notable, dominée par le pin et plutôt caractéristique des boisements de moyenne montagne. La présence de lacs et de pelouses sèches en font une zone très touristique, en particulier autour du lac d'Aydat. Enfin, l'est du territoire constitue les premiers reliefs du Livradois avec, un peu en amont, une zone

[7] La commune de Saulzet-le-Froid, située à l'extrême-ouest du territoire, a depuis été rattachée à une autre intercommunalité. L'entité paysagère des Monts Dore n'est donc pas abordée dans le cadre de notre étude.

de boisement particulièrement importante (la forêt de la Comté), des hameaux dispersés, des vergers, notamment de noyers, ainsi qu'une dominante de prairies.

1.2.3. Les grands enjeux territoriaux

Les enjeux territoriaux découlent donc naturellement de cette diversité paysagère, mais aussi de dynamiques exogènes comme l'attractivité des villes environnantes et des préoccupations sociétales croissantes (transition énergétique et écologique, relocalisation de l'agriculture et de l'alimentation, caractéristiques socio-économiques...). Lors de notre travail d'analyse, nous les avons structurés en plusieurs catégories : milieux naturels, agriculture, paysage, et dynamiques territoriales (voir annexe 2). Cette classification a été élaborée dans l'optique de pouvoir dégager par la suite plusieurs scénarios d'usage des terres, et de qualifier les évolutions possibles de l'utilisation du foncier.

La figure 8 recense les principaux enjeux qui ressortent de cette analyse bibliographique, axée sur les orientations, actions et choix politiques qui influent ou peuvent influer sur les (agro)écosystèmes. Plusieurs documents étaient à notre disposition pour effectuer l'analyse, qui portent sur des échelles territoriales parfois infra- ou extra-territoriale. L'annexe 1 recense ces différentes ressources. En outre, les retranscriptions de plusieurs entretiens effectués par Magali Weissgerber et Amélie Menet ont constitué une autre source de données, qui offrent le point de vue d'acteurs locaux ayant une vision plus technique de leur territoire.

Dynamiques territoriales	Milieux naturels
**Tourisme et attractivité**<br><br>- Promotion des ressources et savoirs-faire locaux<br>- Aménagement activités de pleine nature<br>- Valorisation économique du Val d'Allier	**Cours d'eau et milieux aquatiques**<br><br>- Mise en valeur du Val d'Allier<br>- Maintien de la qualité écologique des cours d'eau<br>- Gestion quantitative de la ressource en eau<br>- Prévenir l'eutrophisation
**Habitat**<br><br>- Adaptation de l'urbanisme aux enjeux climatiques<br>- Développement maîtrisé de l'habitat<br>- Densification du tissu urbain	**Continuités écologiques**<br><br>Maintien et amélioration des trames écologiques
**Filière forêt-bois**<br><br>- Exploitation durable et locale des milieux boisés<br>- Développement d'une filière forêt-bois	**Espaces naturels sensibles**<br><br>- Valoriser les ENS<br>- Préservation des espaces à fort intérêt écologique
	**Forêts**<br><br>- Préserver la qualité écologique des milieux boisés<br>- Conservation de parcelles forestières<br>- Gestion durable

Agriculture
**Foncier agricole**<br><br>- Maîtrise de l'artificialisation des terres<br>- Maintien de la vocation agricole<br>- Surveillance de la dynamique d'enfrichement
**Pratiques culturales**<br><br>- Développement du pastoralisme<br>- Evolution des pratiques culturales<br>- Transition des exploitations laitières et d'élevage
**Types de production**<br><br>- Soutien de l'activité maraîchère<br>- Soutien de l'arboriculture<br>- Développement de l'apiculture<br>- Maintien des productions existantes
**Alimentation**<br><br>- Développement et soutien des circuits courts<br>- Promotion des ressources du territoire<br>- Renforcement de l'autosuffisance alimentaire<br>- Produire, transformer et consommer localement
**Agroécologie**<br><br>- Renforcement du réseau bocager et des boisements en plaine<br>- Maintien des IAE dans les agroécosystèmes<br>- Conciliation entre agriculture et biodiversité
**Énergie**<br><br>Développement de la méthanisation

Milieux naturels (suite)
**Biodiversité faunistique et floristique**<br><br>- Conservation de la diversité des espèces et des milieux<br>- Mise en place d'une gouvernance intercommunale des ressources naturelles<br>- Maintien des noyaux écologiques (en zones d'estive)<br>- Maintien d'éléments de biodiversité sur la totalité du territoire
**Risques naturels et environnementaux**<br><br>- Prévention des risques d'inondations, de coulées de boue, d'incendies et de mouvements de terrain<br>- Limitation des risques d'érosion et de ruissellement

Paysage
**Urbanisation**<br><br>- Diminution des coupures paysagères<br>- Lutte contre l'uniformisation du paysage<br>- Limitation de l'extension urbaine
**Conflits d'usage**<br><br>- Anticipation des conflits liés à l'eau<br>- Conciliation entre développement urbain, renouvellement urbain, et préservation des terres agricoles
**Enfrichement**<br><br>Lutte contre l'enfrichement des coteaux de Limagne
**Continuités paysagères et écologiques**<br><br>- Conservation de la vue et de la silhouette des bourgs<br>- Maintien des continuités écologiques<br>- Restauration de la dynamique fluviale de l'Allier<br>- Préservation des spécificités paysagères des communes de montagne

Figure 8. Les principaux enjeux territoriaux de Mond'Arverne Communauté à travers quatre prismes : agriculture, milieux naturels, paysage, et dynamiques territoriales.

1.3. Apports théoriques complémentaires du mémoire

1.3.1. Choix analytiques préliminaires

Au sein du précédent point, nous avons détaillé les cadres d'analyse mobilisés par l'équipe de scientifiques dans le cadre du projet PACSEN, à savoir ceux des systèmes socio-écologiques, de l'analyse institutionnelle et de développement, et de la viabilité. Cela avait pour but de replacer les missions de stage dans un contexte scientifique précis, mais également de mieux appréhender les différentes finalités du projet. Toutefois, d'autres apports théoriques seront présentés et mobilisés dans ce mémoire, qui font l'objet des paragraphes suivants.

Une difficile mobilisation de l'analyse institutionnelle

La volonté d'étudier d'autres travaux découle en premier lieu de la difficulté à s'approprier l'analyse institutionnelle et de développement. Le manque de visualisation précise de livrables allant dans ce sens ne permettait pas, selon nous, d'appuyer la totalité de notre travail sur ce type d'analyse, au risque de ne pas pouvoir valoriser les résultats obtenus dans le cadre du stage de façon satisfaisante.

Si ce cadre d'analyse a été posé très clairement de façon théorique, avec une mise en avant des intérêts qu'il présente au vu de la problématique du projet de recherche, nos difficultés ont avant tout résidé dans l'articulation de cette théorie avec des données brutes, en l'occurrence des comptes-rendus d'entretiens et une analyse bibliographique de documents programmatiques. Certains travaux réalisés dans le cadre du stage ont donc permis d'alimenter une analyse institutionnelle nécessaire au projet, mais ils n'ont pas pour autant constitué le cœur de ce mémoire.

De plus, le territoire d'étude n'a probablement pas facilité l'utilisation de cette analyse, bien que le choix de ce dernier soit compréhensible. En effet, s'appuyer sur des ressources déjà existantes au sujet d'un territoire facilite l'entrée dans ses enjeux et permet un

gain de temps non négligeable. En parallèle, notons que les intercommunalités font l'objet d'un intérêt accru de la part des dernières réformes territoriales et lois de décentralisation (Loi NOTRe, 2015 ; Loi MAPTAM, 2014) : en effet, leur compétences réglementaires (et donc leur légitimité à intervenir sur différents champs de politiques publiques) se trouvent élargies, notamment en proportion de leur nombre d'habitant·e·s. Il est donc compréhensible que le milieu de la recherche se penche également sur cet échelon, en particulier dans des projets en lien avec l'usage des terres, sujet faisant écho à plusieurs compétences intercommunales (urbanisme, agriculture, forêt, environnement...). Dans une perspective de valorisation des travaux de recherche, voire d'une mise en place opérationnelle de certaines préconisations, cette proximité avec la réalité institutionnelle est plus qu'intéressante.

Néanmoins, si les intercommunalités ont gagné en compétences réglementaires, cela n'a pas nécessairement été accompagné par un gain de légitimité auprès des citoyen·ne·s[8]. Dans les faits, ces groupements de communes demeurent des territoires encore peu appropriés par les individus, voire peu identifiés, qui sont perçus comme des instances relativement éloignées des réalités quotidiennes (AdCF, 2013). Dès lors, la question de la contribution et de l'implication des acteurs à des projets intercommunaux se pose, ce qui peut se traduire concrètement par des difficultés à dialoguer sur des problématiques et des projets focalisés sur cet échelon[9]. En outre, nous l'avons vu, Mond'Arverne Communauté agglomère une diversité de sous-espaces, avec des contextes écologiques locaux qui varient d'un ensemble à un autre. Si cela constitue une richesse en termes de données récoltées, il est cependant difficile de dégager des résultats généralisables à tout le territoire, mais aussi de mobiliser sur la durée le concept de système socio-écologique qui suppose, outre l'appropriation dudit système par les acteurs, une certaine homogénéité écologique dans l'objet étudié (à titre d'exemples, les cas étudiés par Ostrom et son équipe sont une forêt communale ou encore une zone de pêche). En pratique, nous avons d'ailleurs été amenés à rediviser notre territoire d'étude en plusieurs zones pour pouvoir gagner en précision (à ce sujet, voir le point 2.1.1.).

---

[8] Dans un sondage réalisé en 2021 par l'institut CSA, 33 % des Français·e·s jugeaient « clairs » le rôle et les compétences des intercommunalités, en dernière place après ceux des communes, des départements et des régions (Sénat, 2021).

[9] Voir sur ce point les résultats des entretiens.

Des focus dans la problématisation du sujet et un approfondissement de notions clés

L'un des choix effectués dans ce mémoire a été de se focaliser sur l'adaptation à une forme de changement global, à savoir le changement climatique. Ce choix peut paraître réducteur, en comparaison avec l'ambition globale du projet qui vise à prendre en compte une plus grande diversité de changements. Mais nous pensons qu'il a pu nous permettre d'investiguer un peu plus en détail, dans les contraintes de temps que nous avions, la manière dont est prise en compte cette question à l'échelle d'un territoire bien précis. Le focus sur le changement climatique a en grande partie été déterminé par la disponibilité de la littérature en géographie et développement territorial sur le sujet. En effet, cette dernière nous a semblé abondante et pertinente à mobiliser pour l'étude d'une intercommunalité, comme nous le détaillons dans le point 1.3.2.

En parallèle du focus choisi pour la problématique du mémoire, les ressources bibliographiques complémentaires que nous avons pu étudier ont permis de développer certaines notions clés. Alors que la « *production de scénarios* » et « *l'analyse de conflits* » apparaissaient par exemple comme des objectifs allant de soi dans le cadre du projet, une analyse bibliographique plus approfondie de ces notions[10] a permis d'illustrer qu'elles reflètent en réalité des choix méthodologiques et conceptuels précis en sciences sociales, que nous avons pu mettre en exergue avec d'autres concepts dans notre revue de la littérature. L'un des apports souhaités du mémoire est donc de réussir à fournir des références bibliographiques autour de ces notions, mais aussi à les appliquer à notre cas d'étude.

1.3.2. Adapter les territoires au dérèglement climatique : un enjeu alliant gouvernance prospective et gestion des conflits d'usage

Le point suivant a vocation à présenter une brève revue de la littérature sur les concepts abordés dans ce mémoire, en complément de ce qui a été présenté en 1.1. Nous nous pencherons tout d'abord sur la notion d'adaptation, figurant au cœur de ce travail, et sur la manière dont elle est appréhendée à l'échelle des territoires. Puis, nous nous intéresserons à celles de prospective territoriale et de conflits d'usage, qui sont, pour la première, un outil

[10] Prospective territoriale et conflits d'usage.

méthodologique permettant d'investiguer notre sujet, et pour l'autre, une manière d'analyser nos résultats.

L'adaptation au dérèglement climatique : vers une institutionnalisation et une opérationnalisation progressives sur les territoires

L'une des notions clés du projet PACSEN et du présent rapport est celle d'adaptation des territoires. Pour des raisons de praticité et d'accès à la littérature scientifique, nous avons choisi de focaliser notre travail sur l'adaptation au dérèglement climatique. En effet, ce type d'adaptation appliqué à la gestion des territoires est le plus documenté et étudié, en comparaison à l'adaptation à l'érosion de la biodiversité, par exemple.

Toutefois, nous avons pleinement conscience que le dérèglement climatique est loin d'être l'unique élément à l'origine de processus d'adaptation des territoires. Dans un chapitre d'ouvrage (dir. Soussana, 2013), Bertrand et *al.* analysent les tenants et les aboutissants de l'adaptation des filières agricoles et forestières pour un territoire. Cette dernière est déterminée par plusieurs facteurs, dont « *les capacités d'adaptation intrinsèques liées notamment au milieu naturel, aux modes de production, aux ressources humaines* », « *l'état et le type de stratégies adaptatives existantes* »[11], ainsi que « *la place et le degré de patrimonialisation des filières pour un territoire* ». À titre d'exemple, l'impact de l'augmentation des sécheresses liées au dérèglement climatique sur la production de fourrage aura des effets très différents sur l'économie d'un territoire de montagne qui tire la majeure partie de ses revenus de la vente de produits de terroir, que sur une zone géographique où la production agricole est beaucoup plus diversifiée. Un système socio-écologique n'est pas une entité figée qui viendrait subitement être perturbée par le dérèglement climatique : les mutations induites par cet évènement viennent interagir avec des composantes qui sont elles-mêmes le fruit d'adaptations à des évènements ou contextes socio-politiques.

Précisons d'emblée qu'il n'existe pas encore de définition et de cadre d'analyse partagés pour l'adaptation (Bertrand et *al*., 2012 ; Richard, 2013 ; Quenault, 2013 ; Simonet, 2015). Si l'adaptation est étudiée en biologie dès le XIXe siècle, puis en psychologie et en

[11] Les auteur·e·s citent la recherche d'autonomie à l'œuvre dans plusieurs fermes, qu'elle soit fourragère, énergétique, ou encore relative au mode de commercialisation des denrées.

gestion (Van Gameren et *al.*, 2014), son arrivée en sciences sociales est en effet bien plus récente, jusqu'à constituer un objet de recherche à part entière à partir des années 2000 (Desse et *al.*, 2017). En ce qui nous concerne, nous emprunterons la définition du GIEC, qui est d'ailleurs la plus communément utilisée, à savoir « *l'ajustement des systèmes naturels ou humains en réponse à des stimuli climatiques présents ou futurs ou à leurs effets, afin d'atténuer les effets néfastes ou d'exploiter des opportunités bénéfiques* » (2014).

Le terme d'adaptation est souvent présenté en parallèle de celui d'atténuation (ou *mitigation*), c'est-à-dire « *la stabilisation des concentrations de gaz à effet de serre [...] à un niveau qui empêche toute perturbation anthropique dangereuse du système climatique* » (ADEME, 2021). Les travaux du GIEC ont participé à instaurer cette dichotomie, que l'on retrouve dans la littérature, à savoir celle de l'atténuation et de l'adaptation au dérèglement climatique. C'est notamment parce que les modalités de mise en œuvre de ces deux types de politiques sont différentes qu'elles ont longtemps été pensées séparément (Bertrand & Richard, 2015). Biesbroeck et *al.* (2010), citent plusieurs raisons à cela, comme par exemple des différences dans les indicateurs utilisés ou dans les périmètres d'application (échelle des collectivités territoriales pour les politiques d'atténuation, entités biophysiques ou bio-régionales pour les politiques d'adaptation) (Bertrand & Richard, 2012). Toutefois, penser ces deux types de politiques de manière cloisonnée s'avère de moins en moins pertinent. En effet, « *ce sont bien les politiques d'atténuation, et donc l'évitement d'un emballement climatique incontrôlé, qui rendent possibles de concevoir des stratégies d'adaptation efficaces et partagées* » (Bertrand & Richard, 2015, p. 8.). Deux grandes formes d'adaptation se sont peu à peu distinguées dans la littérature scientifique : l'adaptation *incrémentale* (ou « état »), qui vise le maintien d'un système par une logique d'ajustement à plusieurs impacts néfastes, ainsi que l'adaptation *transformationnelle* (ou « processus ») (Magnan, 2009 ; Simonet, 2017). Cette dernière vient questionner les mécanismes institutionnels, financiers et techniques en place, et en particulier leur pertinence à pouvoir diminuer les vulnérabilités (Simonet, 2015). Elle implique donc des changements beaucoup plus importants, notamment d'activités et de pratiques (Noble et *al.*, 2014).

Comment l'adaptation se traduit-elle sur les territoires ? Dans les discours, puis dans les politiques publiques ? Ce questionnement révèle l'une des principales critiques effectuées vis-à-vis de cette notion. En effet, si les travaux du GIEC, et notamment leur cinquième rapport (2014), donnent une place croissante à l'adaptation dans les politiques climatiques,

son manque d'opérationnalité est régulièrement pointé (Van Gameren et *al.*, 2014 ; Simonet, 2017). Plusieurs raisons expliquent cette déconnexion entre appropriation politique croissante et difficile traduction en actions : la présence de freins cognitifs (Gifford, 2009), d'un déficit de représentation dans le quotidien des acteurs en comparaison avec d'autres enjeux bien identifiés (Bertrand & Fouqueray, 2017), des mécanismes de dépendance au sentier[12], le manque de bénéfices perçus (Bertrand & Richard, 2015), mais aussi un manque d'indicateurs et de méthodes pour évaluer ce qui relève d'une adaptation réussie (Van Gameren et *al.*, 2014). En outre, l'idée même de s'adapter a pu aussi être perçue comme une forme d'échec sur la capacité des sociétés à endiguer le dérèglement climatique, tout comme une remise en question explicite de la soutenabilité du système économique depuis l'industrialisation (Boissier, 2020).

En France, les premières actions et politiques d'adaptation sont menées avec une entrée thématique, telles que les problématiques de la gestion quantitative de l'eau, ou encore la pérennisation de filières agroalimentaires. L'entrée spatiale est aussi présente, avec des politiques qui se structurent autour d'enjeux très locaux (inondations et fortes chaleurs en milieu urbain, adaptation de l'agriculture en milieu rural). À l'échelle nationale, le Plan National d'Adaptation pour la période 2011-2015 est la première forme d'institutionnalisation. Par la suite, la loi Grenelle 2 en 2010 instaure les Plans Climat Énergie Territoriaux (PCET) pour les collectivités de plus de 50 000 habitant·e·s, ainsi que les Schéma Régionaux Climat Air Energie (SRCAE), qui constituent les premiers cadres juridiquement contraignants en matière de politique climatique, et qui ont vocation à territorialiser l'adaptation (Desse et *al.*, 2017 ; Richard, 2013). L'approche développée est, à cette époque, centrée sur l'énergie, et ne prend pas nécessairement en compte d'autres enjeux qui peuvent accentuer la vulnérabilité des territoires face au dérèglement climatique, tels que l'étalement urbain (Desse et *al.*, 2017). *A contrario*, d'autres outils qui ne traitent pas de front la question climatique sont parfois plus susceptibles de favoriser la résilience, comme les Plans Locaux d'Urbanisme. En outre, l'adaptation apparaît de manière relativement disparate dans les documents programmatiques : pour le volet énergétique dans les Plans Climat Air Énergie Territoriaux, pour la lutte contre les îlots de chaleur dans les documents d'urbanisme, ou encore dans les documents de gestion des risques naturels pour l'érosion des littoraux. Si

[12] Le concept de dépendance au sentier, ou *path dependence*, a été formalisé en science politique dans les années 1990. Il met en avant le poids des décisions et institutions passées dans les décisions politiques présentes, expliquant la difficulté pour des acteurs publics à s'engager dans des trajectoires radicalement différentes (Palier, 2019).

cette notion est traitée de manière très variable selon les problématiques locales, elle peine globalement à être appréhendée de façon intégrée.

Usage de démarches prospectives pour l'adaptation : des outils d'aide à la décision face à l'incertitude ?

L'approche prospective, en l'occurrence par la production de scénarios, a été présente dès le début du projet PACSEN pour appréhender l'adaptation. L'un des principaux objectifs est en effet d'élaborer une série de scénarios d'usage des terres, destinés à informer les acteurs locaux des services écosystémiques relatifs à chaque scénario, mais aussi susceptibles de les appuyer dans leur prise de décision. Ce type d'exercice n'est pas nouveau, puisque de nombreuses démarches prospectives sont élaborées à l'échelle de territoires biophysiques, institutionnels, ou encore concernant l'avenir de filières économiques (Aigrain et *al.*, 2016 ; Cerema, 2019). Initié à l'échelle nationale par la délégation à l'aménagement du territoire et à l'action régionale (DATAR), les collectivités et autres territoires locaux se sont peu à peu saisis de la démarche pour développer une vision partagée des dynamiques spatiales et des actions à mettre en œuvre, en particulier dans un contexte de changements globaux (Perrin, 2016).

Néanmoins, il peut être judicieux de préciser que la prospective n'est qu'une des manières possibles de concevoir l'adaptation. Simonet et Leseur (2015), lors d'entretiens avec plusieurs acteurs d'un territoire, démontrent que trois types de réponses émergent concernant l'interprétation de la notion d'adaptation : cette dernière est en majorité perçue comme le fait de modifier des pratiques et normes sociales, pour favoriser une gestion durable des ressources et une meilleure prise en compte des systèmes écologiques (« perspective naturaliste ») ; puis comme le fait d'aménager et de protéger des infrastructures et du bâti existant (« perspective résiliente ») ; et enfin comme la volonté de réduire l'incertitude en mobilisant des connaissances et des outils prospectifs, type scénarios (« perspective prospective »).

En effet, dans un contexte d'évolutions environnementales peu prévisibles, les approches prospectives ont l'avantage de constituer des stratégies flexibles, qui laissent la possibilité de modifier un programme d'actions, et d'intégrer au fur et à mesure de nouvelles informations et connaissances (Richard, 2013). De manière générale, elles facilitent la

compréhension des dynamiques territoriales, notamment dans un contexte de changements globaux et de coopération croissante entre acteurs (Durance et *al.*, 2007).

Les scénarios, en particulier, permettent d'étudier la faisabilité de différentes options mais aussi de les hiérarchiser selon leur pertinence, notamment selon différentes projections temporelles (Church, 2018). Cependant, selon les acteurs qui élaborent les scénarios, ces derniers peuvent comporter plusieurs biais et refléter une vision partielle du territoire, d'où la nécessité de veiller à une transdisciplinarité (Van Ruijven et *al.*, 2014). Nous avons vu que l'un des freins à la mise en place de politiques d'adaptation est le manque de perception par les acteurs des bénéfices associés, ou bien à l'inverse des pertes évitées. La mise en place de stratégies, ou du moins d'actions, nécessite donc d'avoir défini collectivement les risques en lien avec le dérèglement climatique. Une étape préalable à toute action serait ainsi de « *co-produire et de diffuser des connaissances* » (Bertrand & Fouqueray, 2017), afin de créer un référentiel commun.

Perrin (2016), évoque un contexte d'*écologisation de la prospective* lié aux incertitudes environnementales, qu'il nous paraît pertinent de mettre en parallèle avec l'adaptation au dérèglement climatique. En effet, si tout ce qui relevait du non-humain était autrefois considéré comme facteur de stabilité dans les prises de décision, la nature constitue désormais l'un des plus profonds facteurs d'incertitude pour les sociétés humaines (dérèglement climatique, érosion de la biodiversité, disparition des milieux naturels...). Le concept de services écosystémiques est notamment mobilisé dans ce contexte, puisqu'il pointe les liens d'interdépendance qui unissent systèmes humains et écologiques. Mobilisé dans des exercices de prospective, il permet de prendre en compte de façon systémique les facteurs environnementaux dans les visions projetées sur le futur. Perrin précise que l'un des intérêts de l'approche par les SE est de décloisonner et dé-sectoriser des enjeux pourtant liés, en mettant en avant leurs liens d'interdépendance. De plus, la dimension spatiale des SE permet de cartographier et d'identifier des zones d'action prioritaires, notamment en vue d'orientations d'aménagement spécifiques. Toutefois, il précise aussi que les attentes des acteurs en matière de SE sont contradictoires et peuvent évoluer dans le temps, en fonction du contexte : cette approche nécessite donc une grande flexibilité, et la possibilité de pouvoir transiter rapidement d'un scénario à un autre.

L'adaptation des territoires face à des pressions environnementales : vectrice de tensions et conflits d'usage de l'espace ?

Il est maintenant établi que les changements globaux, en l'occurrence le dérèglement climatique, peuvent générer des pressions sur les écosystèmes dont les sociétés humaines dépendent, en particulier les agroécosystèmes. L'une des hypothèses du projet est que dans un contexte d'altération, voire de suppression de certains de ces écosystèmes, des conflits relatifs à l'usage de l'espace restant peuvent émerger (d'ordre social, économique ou politique). L'outil d'aide à la décision doit en effet proposer plusieurs scénarios consensuels, qui maximisent les préférences des groupes d'individus et anticipent l'apparition de conflits. Dans ce travail, nous essaierons donc d'analyser la présence de tensions et/ou conflits et de les caractériser, afin de voir si ces derniers sont intégrés dans le cadre d'une gouvernance intercommunale.

Les espaces ruraux et périurbains, comme Mond'Arverne Communauté, peuvent apparaître comme des « réceptacles » de conflits divers du fait de leur multifonctionnalité. En effet, ils possèdent simultanément des fonctions économiques et de production, résidentielles et récréatives, ainsi que de conservation (par exemple de la biodiversité) (Torre et *al.*, 2006). Au sein de ces mêmes espaces, les agroécosystèmes représentent un enjeu fort : tout d'abord parce qu'ils constituent le mode d'usage des terres majoritaire, mais aussi parce qu'ils fournissent des services écosystémiques essentiels et variés, qu'ils soient de l'ordre de la production, de la durabilité ou encore des attentes sociétales (Bretagnolle, 2013). Le mode de gestion de ces agroécosystèmes peut donc faire l'objet de rapports de force entre individus et/ou groupes possédant des intérêts antagonistes en matière de SE.

S'il n'est pas possible d'affirmer une hausse de la conflictualité liée aux usages de l'espace, ce phénomène semble néanmoins récurrent, tout comme l'on constate une hausse des recours aux tribunaux administratifs, ou encore de l'opposition vis-à-vis de projets d'aménagement et d'infrastructures (Torre et *al.*, 2016). Avant toute chose, il est essentiel de différencier une tension d'un conflit. Tandis que la première n'aboutit pas nécessairement à des actes conflictuels et peut perdurer dans le temps, le deuxième constitue un engagement qui peut prendre différentes formes : « *recours en justice, publicisation (différend porté devant des instances publiques), médiatisation, confrontation verbale, destruction de biens ou d'infrastructures, ou encore production de signes (interdiction d'accès, barrières...)* » (Torre

et *al.*, 2016, p. 13). D'autres caractéristiques relatives aux conflits d'usage de l'espace et de voisinage sont leur inscription sur un territoire, et leur matérialité. Dans ce travail, tensions et conflits seront abordés, notamment parce que les tensions peuvent constituer des conflits latents, et sont ainsi pertinentes à évoquer lorsque l'on évolue dans un contexte d'incertitude.

Dans l'analyse des conflits d'usage, Torre et *al.* (2006) pointent la nécessité de ne pas confondre usages et usagers de l'espace, afin d'éviter des oppositions trop rapides entre des catégories d'acteurs (la traditionnelle opposition « ruraux *versus* néo-ruraux », par exemple). Les auteurs mettent ainsi en lumière trois phénomènes concomitants : un même acteur peut avoir des usages de l'espace différents, peut présenter des facettes différentes au sein de groupes sociaux selon ses intérêts à un instant T, tout comme ses réactions peuvent être différentes au sein de groupes sociaux homogènes lorsque certains évènements l'affectent. Aussi, les auteurs invitent à analyser les conflits par le prisme des usagers plutôt que des usages, bien que cette approche ajoute de la complexité puisqu'elle prend en compte une multitude d'interactions entre acteurs, elles-mêmes ayant lieu au sein d'institutions (Torre et *al.*, 2006).

2. MÉTHODES D’ENQUÊTE : ANALYSE BIBLIOGRAPHIQUE ET CONDUITE D’ENTRETIENS

2.1. Récolte de données quantitatives sur les haies et plusieurs services écosystémiques associés

2.1.1. Des choix méthodologiques déterminés par les contraintes de modélisation

Un focus sur une infrastructure agroécologique : la haie

Les contraintes liées à la modélisation explicitées au point 1.1.4. ont nécessité de se focaliser sur une seule infrastructure agroécologique d’intérêt : les haies. En effet, l’étude simultanée de tous les types d’infrastructures (semi)naturelles du territoire paraissait difficilement réalisable pour l’élaboration d’une première preuve de concept. Au niveau des missions de stage, cela s’est traduit par une redéfinition de la fiche de poste, où l’objectif principal est devenu le suivant : « *étudier l’adaptation incrémentale du réseau de haies de notre territoire d’étude, pour assurer la viabilité dans le temps d’un jeu de services écosystémiques d’intérêt collectif »* (fiche de poste révisée, mai 2021).

Définir ce qu’est une haie n’est pas simple, tant les définitions sont nombreuses dans la littérature. Nous retiendrons ici celles de Fanny Le Guillou (2020) et Léo Magnin (2021), qui soulèvent déjà quelques enjeux en sciences sociales autour de cette IAE :

> « Un ensemble d’éléments ligneux, jugés utiles par certains propriétaires et gérés en conséquence, associés à d’autres linéaires hérités ayant autrefois un “rôle” mais aujourd’hui

abandonnés, et à des haies considérées comme gênantes, qui sont gérées de façon à ne plus l'être » (Le Guillou, 2020, p. 20).

« Une entité paysanne, dévaluée lors de la modernisation agricole, puis requalifiée en objet digne de curiosité en faveur de l'écologisation » (Magnin, 2021, p. 26).

Ce que l'on considère comme une haie peut ainsi être amené à évoluer dans le temps, en fonction des intérêts des acteurs en présence et des choix de gestion. Ces intérêts sont ceux de propriétaires et d'individus isolés, certes, mais pas seulement. Dans sa définition, Magnin souligne le poids de choix socio-économiques collectifs dans le regard porté par la société sur les haies : celui de la modernisation agricole et du remembrement dans un premier temps, durant lequel les haies constituent un objet contraignant suscitant peu d'intérêt, et celui de préoccupations écologiques croissantes dans un second temps, où les services écosystémiques du bocage sont (re)découverts et ont vocation à être préservés.

D'un point de vue écologique, les haies sont un objet d'étude particulièrement intéressant pour plusieurs raisons. Tout d'abord, elles sont pourvoyeuses d'une grande diversité de services écosystémiques, d'approvisionnement, de support, de régulation, ainsi que socio-culturels. Les principaux d'entre eux, à l'échelle des agroécosystèmes, sont énoncés et schématisés sur la figure 9.

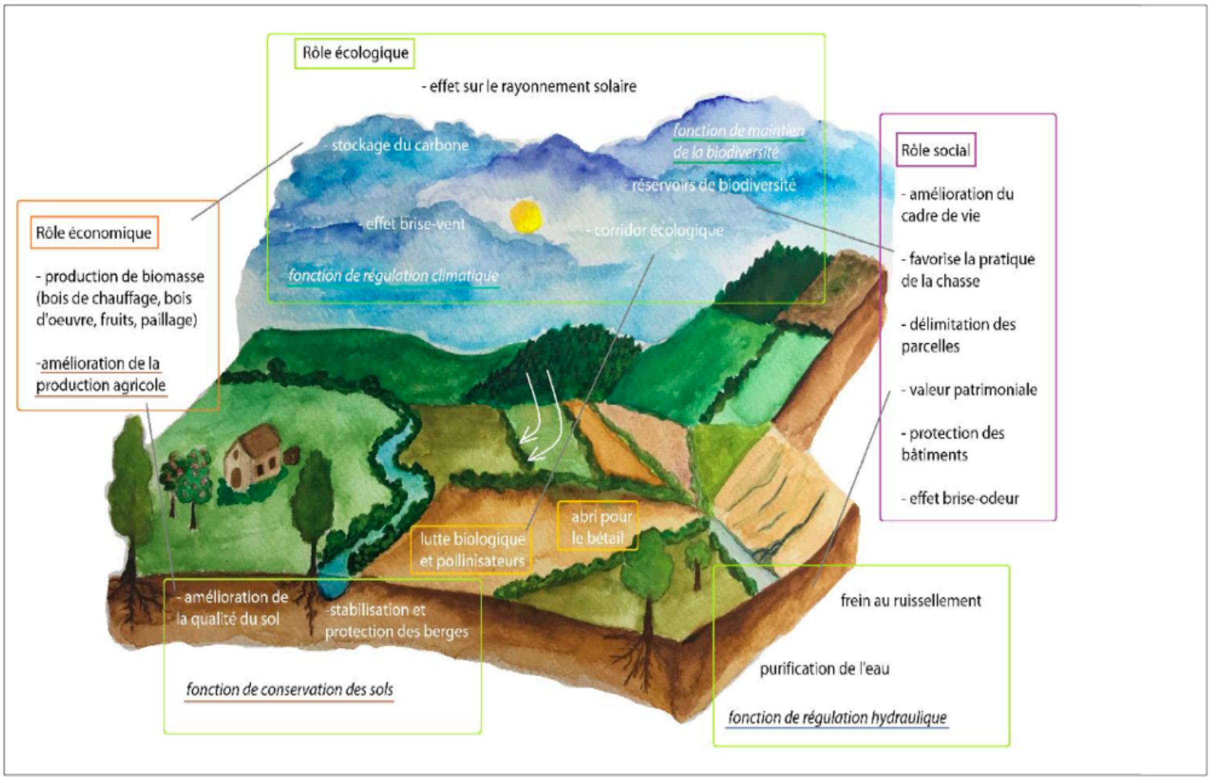

Figure 9. Schéma représentant la multi-fonctionnalité des haies sur un bassin versant. Source : Le Guillou, 2019, p. 37.

De plus, certains de ces services peuvent être considérés comme des *services d'adaptation* (Lavorel et *al.*, 2020), au sens qu'ils permettent de limiter les effets négatifs des changements globaux sur les sociétés humaines, et donc d'accompagner leur adaptation. On citera par exemple le service de lutte contre l'érosion, qui peut atténuer ou empêcher des dégâts matériels suite à de fortes précipitations, ou encore le rôle de « micro-climat » joué par le bocage.

Un focus sur six services écosystémiques

Le nombre de services écosystémiques fournis par les haies rend difficile une étude exhaustive de chacun d'eux. De plus, la question des indicateurs se pose. En effet, certains services s'avèrent plus aisés à évaluer que d'autres, et facilitent par la suite le travail de modélisation. Dans le cadre de PACSEN, il a été décidé de s'intéresser à six services écosystémiques, choisis principalement pour deux raisons. D'une part leur pertinence au vu du contexte géographique de notre territoire d'étude, ou en d'autres termes leur forte

probabilité d’être considérés comme importants par les acteurs locaux. D’autre part, ces services ont été retenus pour leur potentiel d’évaluation, qui permettent aux scientifiques d’utiliser des données quantitatives et précises pour l’élaboration du modèle.

Filtration de l’eau

Ce service correspond à la filtration qualitative de l’eau, par l’élimination des polluants, et notamment des nitrates. « *Les racines des arbres sont souvent plus profondes que celles des cultures, elles peuvent intercepter des nutriments apportés en excès ou produits par minéralisation de la matière organique des sols* » (Viaud & Thomas, 2019, p. 14). On citera à ce sujet la directive nitrates, appliquée depuis 1991 et réactualisée, pour préserver la qualité des eaux. Elle impose aux agriculteurs et agricultrices ayant des parcelles proches de cours d’eau d’installer des zones tampons entre les cultures et les berges, à savoir des « *surface en herbe, talus, arbres, haies, zone boisée, ou tout aménagement visant à limiter le ruissellement et le transfert des fertilisants vers le cours d’eau* » (Préfecture des Bouches-du-Rhône, 2017, p. 8). Le rôle des haies concernant la filtration de l’eau est donc bien identifié, et même institutionnalisé.

Maintien du sol

Le terme de maintien du sol n’est pas le plus rencontré dans la littérature, on parlera plus fréquemment de *lutte contre l’érosion du sol*. Lors des entretiens, nous avons cependant utilisé ce premier terme pour des raisons de facilité. Il s’agit ici du rôle de retenue de la terre effectué par les haies au sein des parcelles agricoles, qui évitent ou diminuent les descentes de terrain, pouvant provoquer baisse de rendements et dégâts matériels (Soltner et *al.*, 1985). En outre, le système racinaire des haies facilite l’infiltration de l’eau dans les sols et limite ainsi les risques d’érosion causés par la vitesse de ruissellement (Wolton et *al.*, 2014). Comme nous le verrons plus loin, Mond’Arverne est particulièrement concernée par ce service, avec une partie du territoire (les coteaux secs) faisant face à des problèmes d’érosion, se soldant fréquemment par des coulées de boue et des inondations.

Production de bois/biomasse

Le bois bocager constitue une source de valorisation économique possible de la haie pour le monde agricole. Cette dernière peut produire du bois de chauffage ou bois énergie, des plaquettes bocagères, du bois pour des piquets de clôture, et plus rarement du bois d'œuvre (Baudry et *al.*, 2000). Si la production de bois bûche n'est pas la plus pertinente au regard des essences et du mode de gestion des arbres de haies, celle de plaquettes à destination de chaudières ou de litières animales semble prometteuse. Des filières tentent de se structurer dans ce sens, certaines bien installées, comme sur le territoire de Lannion-Trégor Communauté (avec la SCIC Bocagenèse), et d'autres en construction comme dans le Puy-de-Dôme, avec l'appui d'acteurs techniques comme la Mission Haies Auvergne et la Chambre d'Agriculture.

Conservation de la biodiversité

Les haies constituent des réservoirs de biodiversité, au sens qu'elles sont des habitats pour plusieurs espèces floristiques et faunistiques, ainsi que des corridors écologiques, permettant la circulation de ces dernières et facilitant les connexions entre plusieurs zones d'habitat. Dans un contexte de fragmentation et de destruction des habitats naturels et semi-naturels liées aux activités humaines, le maintien de corridors linéaires apparaît comme un enjeu central pour la conservation de la biodiversité, d'où la mise en place de Trames Vertes et Bleues (TVB) (Bergès et *al.*, 2010). Ces outils visent également, outre l'amélioration de la connectivité, à « *compenser la perte d'habitats » ainsi « qu'introduire des individus [...] où les populations sont en voie d'extinction* » (Bergès et *al.*, 2010, p. 27).

Esthétisme paysager

Ce service est probablement le plus difficile à évaluer et à quantifier, tant il relève de dimensions subjectives. Il s'agit ici du bien-être procuré aux humains par la beauté d'une haie, ou d'un paysage comportant des haies. Il comporte également une dimension patrimoniale, d'attachement affectif. Si celle-ci n'est pas apparue de façon flagrante pour Mond'Arverne Communauté, elle l'est en revanche pour d'autres territoires, pour qui les haies font pleinement partie de l'identité paysagère (« le bocage Bourbonnais », « le bocage Normand »). Comme nous le soulignait la directrice de l'association Mission Haies Auvergne

en entretien, la typologie de haies est déterminante lorsque l'on s'intéresse à la question de l'esthétisme. Le type de haies apprécié par les individus est fortement corrélé aux normes socio-culturelles des territoires dans lesquels ils et elles vivent, ou aux groupes sociaux auxquels ils et elles appartiennent. C'est le cas par exemple de préférences recensées à plusieurs reprises pour des haies basses, bien taillées et très denses, qui sont identifiées comme la meilleure manière d'entretenir son terrain, donnent une image de « *travail bien fait* », et sont ainsi gage de respectabilité pour les propriétaires. À l'inverse, certains individus préféreront se trouver dans des chemins très arborés, aux essences variées, et seront plus enclins à choisir des haies hautes.

Support à la production agricole

Dans le cadre des entretiens, le service de support à la production a été détaillé pour plus de simplicité en trois sous-services : l'ombrage pour le bétail, l'habitat pour les auxiliaires de culture, et l'effet brise-vent. Ces trois éléments ont en commun d'avoir un intérêt agronomique et fonctionnel à l'échelle de la parcelle, plus ou moins bien identifié par les agriculteurs et agricultrices. Par exemple, l'ombrage pour le bétail constitue une réelle motivation pour conserver, voire planter des haies, puisque les fortes chaleurs ont un impact direct sur la production laitière, et sur le bien-être animal de manière générale (Chambre d'Agriculture de Loire-Atlantique, 2016). En outre, le service de brise-vent est jusqu'à maintenant la première raison de plantation de haies sur les parcelles. En effet, le bocage diminue les phénomènes d'assèchement ou de destruction des cultures causés par le vent et la grêle, ou, dans le cas d'une exploitation d'élevage, protège les troupeaux (Mission Haies Auvergne, 2015). Enfin, les haies constituent un habitat propice au développement d'auxiliaires de culture (reproduction, hivernage, alimentation...), qui régulent les populations de ravageurs et ont un impact positif sur la productivité agricole (Chambre d'Agriculture du Puy-de-Dôme, 2018).

Notons que les disservices écosystémiques, à savoir les effets négatifs des écosystèmes sur les sociétés humaines, ont été beaucoup moins abordés que les services dans le cadre de l'étude. Toutefois, ils sont pris en compte dans le projet PACSEN et nos échanges avec plusieurs acteurs locaux ont fait remonter deux disservices majeurs liés aux haies : le frein au passage des engins agricoles, ainsi que la baisse de rendements liée à l'ombre et à la rétention d'eau par les arbres. Ces éléments ont tout intérêt à être pris en compte dans des projets

d’aménagement et de gestion bocagère, dans la mesure où ils sont parfois plus souvent conscientisés que les services par les acteurs.

Un focus sur quatre sous-territoires

De même qu’un focus a été fait sur une infrastructure agroécologique et plusieurs services associés, le territoire d’étude a été recoupé en plusieurs zones d’étude, facilitant le travail de terrain et de cartographie pour les écologues. Si nous ne nous sommes pas exclusivement limités à ces sous-territoires dans la conduite d’entretiens, nous avons privilégié les contacts sur ces zones, à savoir les communes de Veyre-Monton, Saint-Saturnin, la Sauvetat et Manglieu.

Ces quatre communes ont été choisies pour plusieurs raisons. Tout d’abord car elles possèdent des caractéristiques écologiques et socio-économiques différentes, ainsi que des linéaires de haies variés en termes de quantité et de types de haies présentes. L’échelle de la commune nous a paru pertinente pour des questions d’accessibilité à des bases de données et aux acteurs, mais aussi parce que ce sont des entités institutionnelles dont les choix d’actions en matière de haies ne sont pas toujours similaires, pouvant ainsi donner lieu à des comparaisons entre sous-territoires.

1) Veyre-Monton

Cette commune est l’une des plus urbanisées de Mond’Arverne, avec 3 588 habitant·e·s (INSEE, 2018). Parmi les communes étudiées, elle est aussi celle dont le linéaire de haies est le moins important (18,22 m/ha) (Minaca, 2021). Caractérisée comme périurbaine, sa forte croissance démographique est liée à sa proximité avec la métropole clermontoise, et accueille ainsi plusieurs habitant·e·s effectuant des déplacements pendulaires quotidiens vers et depuis ce pôle. En termes d’occupation du sol pour l’agriculture, l’on observe une majorité de grandes cultures, mais aussi des vignes, bien qu’en diminution. La commune accueille d’ailleurs la cave Desprat-Saint-Verny, coopérative viticole d’importance pour l’appellation d’origine contrôlée Côtes d’Auvergne. Veyre-Monton se situe sur une zone de coteaux où les coulées de boue, inondations et glissements de terrain sont fréquents. Ainsi, une vigilance particulière est accordée à cette problématique, notamment dans un contexte d’intensification des précipitations. Le cours d’eau de la Veyre traverse la commune.

2) Saint-Saturnin

Saint-Saturnin, commune de 1 167 habitant·e·s (INSEE, 2018), se trouve en interface entre la chaîne des Puys et la plaine de la Limagne, et fait partie de l'aire d'attraction de Clermont-Ferrand. Cette commune relativement touristique avec plus de 10 000 touristes par an, présente des enjeux patrimoniaux forts, autant au niveau du patrimoine bâti (présence d'église romane, d'un château, et de nombreuses habitations traditionnelles) que du patrimoine naturel. Le linéaire de haies y est d'ailleurs le plus fourni de la Communauté de Communes : 41,97 m/ha (Minaca, 2021). Réputé pour son cadre de vie, la conservation et la valorisation de ce patrimoine multiformes sont mis en avant par les élu·e·s et habitant·e·s. Le village est traversé par deux cours d'eau : la Monne et la Veyre. L'activité agricole est en déclin mais reste relativement diversifiée : élevage de bovins laitiers, de volailles, d'ovins, et de caprins, ainsi que des cultures de céréales et de légumineuses. La présence de la forêt est également notable, dont une vingtaine d'hectares est la propriété de la commune. Enfin, Saint-Saturnin est membre du Parc Naturel Régional des Volcans d'Auvergne.

3) La Sauvetat

La Sauvetat est une commune peu dense, composée de 715 habitant·e·s, qui fait partie de l'aire d'attraction de Clermont-Ferrand. L'activité agricole est dominée par la céréaliculture sur des parcelles de grande taille. En conséquence, le réseau de haies est l'un des moins denses de l'intercommunalité (21,15 m/ha) (Minaca, 2021). La plupart des habitant·e·s travaillent au sein de la métropole clermontoise, et sont amené·e·s à effectuer des trajets domicile-travail quotidiens. Par conséquent, le dynamisme du centre-bourg constitue un enjeu particulier sur cette commune, pour faire évoluer l'image de « village-dortoir ». En outre, La Sauvetat est traversée par le cours d'eau du Charlet, dont le mauvais état écologique fait l'objet d'un plan de gestion à l'échelle du Syndicat Mixte des Vallées de la Veyre et de l'Auzon, et donc d'une vigilance particulière.

4) Manglieu

La commune a été choisie pour étudier un sous-territoire plus représentatif de l'extrémité est de Mond'Arverne Communauté, typique des contreforts du Livradois. Plus

rurale, et plus éloignée de la métropole clermontoise, la mosaïque paysagère y est aussi plus diversifiée (prairies, forêts, bocage, zones humides, cultures, arbres isolés…). Le linéaire de haies est globalement bien préservé (23,89 m/ha) (Minaca, 2021) et s'intègre à une activité d'élevage laitier dominante. La commune est membre du Parc Naturel Régional du Livradois-Forez. Manglieu accueille 466 habitant·e·s (INSEE, 2018) qui sont répartis entre le bourg principal et une vingtaine de hameaux dispersés.

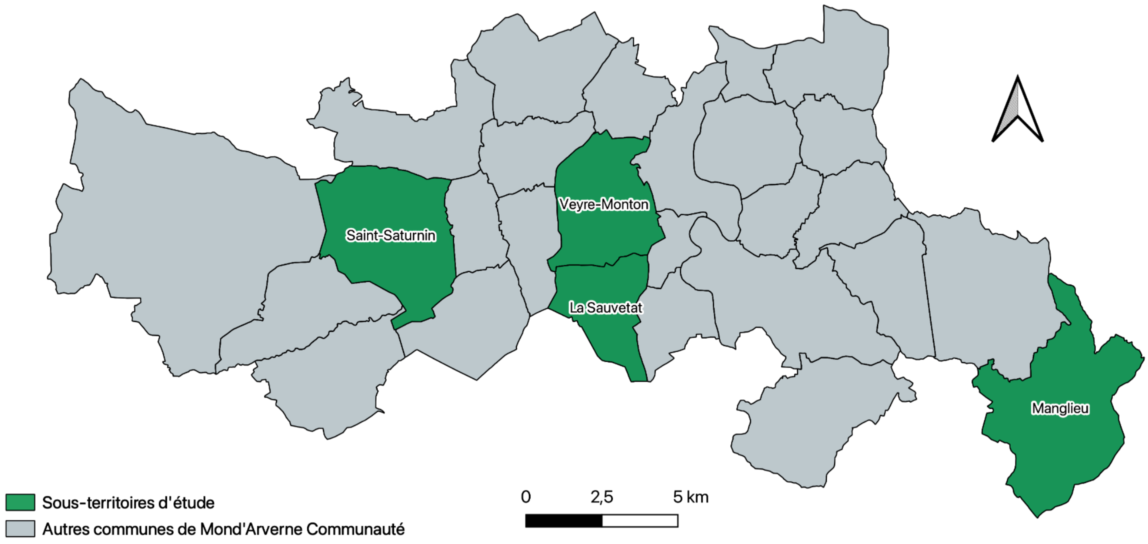

Figure 10. Localisation des quatre communes d'étude au sein de Mond'Arverne Communauté.

2.1.2. L'élaboration du guide d'entretien

La phase d'entretiens constitue l'un des modes de récolte de données parmi plusieurs autres à l'échelle du projet PACSEN. Il est utile de rappeler brièvement les travaux qui ont précédé les entretiens, et qui ont été effectués par le laboratoire d'écologie :

- Une photo-interprétation de l'évolution du linéaire de haies sur le territoire

- Une caractérisation des types de haies présents sur les quatre communes d'étude

L'élaboration du guide d'entretien a donc été réalisée en parallèle du travail des écologues, et a été construite au fur et à mesure de leurs avancées (choix des services pertinents, inclusion de la typologie de haies…).

Les objectifs des entretiens

En parallèle des travaux des écologues, les entretiens avaient pour but de fournir des données plus « sociales » aux travaux de modélisation, par le biais d'échanges assez directifs et ciblés (voir le guide complet en annexe 3), même si dans les faits le format était plutôt semi-directif. Plus précisément, les objectifs étaient les suivants :

- Connaître et quantifier les services et disservices écosystémiques liés aux haies importants pour les acteurs (à partir des six services identifiés) ;
- Décrire l'état actuel du réseau de haies sur le territoire et évaluer la viabilité de cet état pour les acteurs (évaluer leur satisfaction ou insatisfaction concernant le réseau existant) ;
- Connaître les préférences des acteurs en matière de types de haies ainsi que leur répartition spatiale (à partir d'une typologie de haies suggérée) ;
- Apprécier la capacité et les leviers d'actions des acteurs sur le réseau de haies.

Échanger autour de la notion de services écosystémiques : création de supports d'échange

Si la notion de services écosystémiques est au cœur du projet de recherche, elle n'est pas toujours très intelligible pour tous les acteurs. De plus, les haies font l'objet d'un « *déficit de représentation* » (Monier, 2021, retranscription d'entretien), ce qui ne facilite pas des échanges précis en la matière. En d'autres termes, les acteurs ne visualisent parfois pas du tout les haies présentes sur leur territoire ainsi que les services qu'elles fournissent, ce qui rend difficile de s'exprimer sur d'éventuelles préférences d'aménagement bocager. Par

conséquent, s'appuyer sur des supports visuels est apparu rapidement comme une condition nécessaire à la récolte de données et à des échanges fluides.

Deux éléments nécessitaient alors d'être matérialisés. Les *six services écosystémiques* étudiés, mais également *plusieurs types de haies*. En effet, comme énoncé dans les objectifs d'entretien, les données recherchées sont, entre autres, les préférences des acteurs concernant une typologie du bocage.

Dans un premier temps, nous avons donc créé des fiches représentant chacune un service ou sous-service (voir annexe 4), qui comportent une photo prise sur le territoire d'étude (excepté une) ainsi qu'une description synthétique du SE concerné. À l'aide d'une jauge d'importance allant de -5 à 5, les acteurs étaient amenés à classer ces SE selon l'importance donnée, dans le contexte du territoire d'étude, et au vu de leurs préoccupations personnelles et professionnelles.

Dans un deuxième temps, nous nous sommes intéressés à la typologie de haies. En effet, la fourniture de services écosystémiques est fortement corrélée à plusieurs aspects, dont le type de haies et le contexte environnemental dans lequel elles se trouvent (occupation du sol sur la parcelle et aux alentours, exposition au soleil et au vent…). Le GEOLAB a donc proposé une typologie de haies pour l'étude, et s'est attaché à déterminer le potentiel de SE fourni par chacun de ces types (grâce à un travail de caractérisation effectué sur le terrain). En parallèle de cette étude, les résultats des entretiens ont donc vocation à renseigner les préférences des acteurs sur les services écosystémiques d'importance, et les types de haies nécessaires à favoriser pour cela. La typologie de haies choisie pour PACSEN s'est fortement appuyée sur le travail effectué par la Mission Haies Auvergne. Les six types (ou stades) conservés sont les suivants :

- Pas de haie
- Haie basse
- Haie basse ponctuée
- Haie ancienne (ou « relictuelle »)
- Alignement d'arbres
- Haie haute

Pas de haie

Par cette dénomination, nous faisons référence à des paysages de monocultures avec une absence de haies sur plusieurs dizaines d'hectares, voire une absence d'arbres isolés.

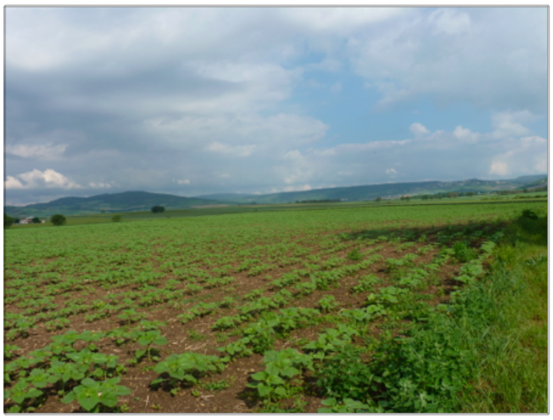

Haie basse

Une haie entre deux et quatre mètres de haut, soit récemment plantée et amenée à évoluer en haie haute, soit régulièrement entretenue et qui a vocation à rester comme telle.

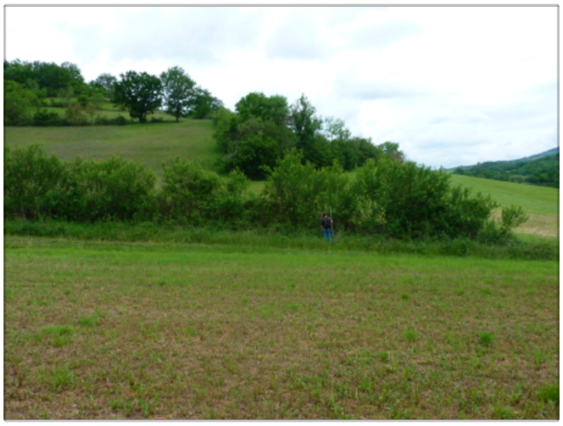

Haie basse ponctuée

Une haie basse (entre deux et quatre mètres) ponctuée de grands arbres (d'une dizaine de mètres).

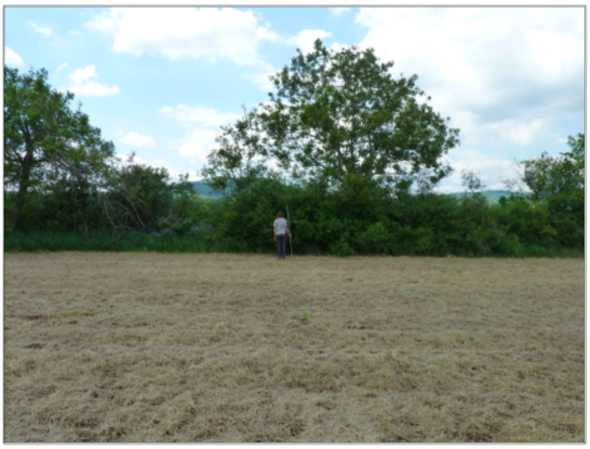

Haie ancienne (ou « relictuelle »)

Une haie qui fut auparavant plus fournie, et dont l'aspect a été modifié pour des contraintes d'entretien ou des choix de gestion (e.g. passage des engins agricoles). Elle peut être mono- ou pluri-strates.

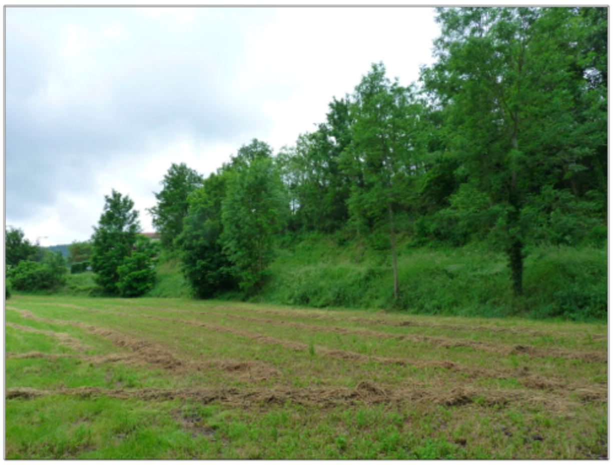

Alignement d’arbres

Des arbres de taille moyenne à grande, sans strate arbustive, et avec une strate herbacée peu fournie et diversifiée.

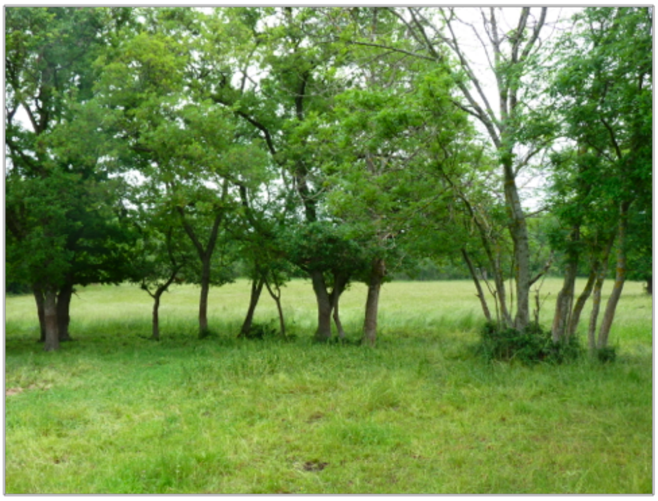

Haie haute

Une haie d’une dizaine de mètres de haut, avec des strates arborée, arbustive, et herbacée. Elles sont, entre autres, les plus rencontrées en bord de cours d’eau (ripisylve).

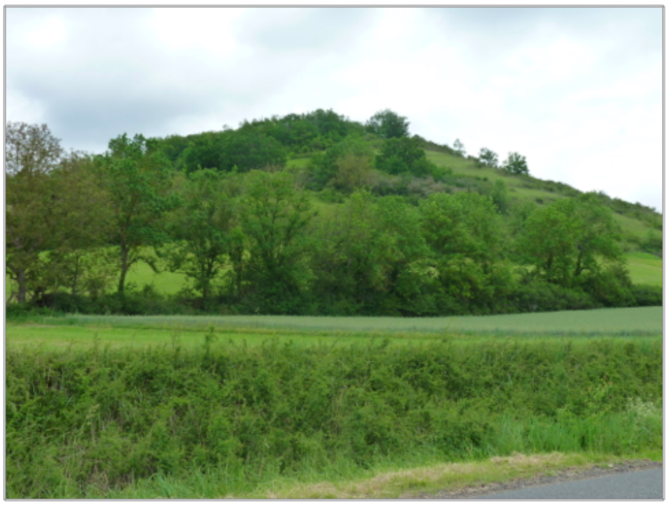

Les acteurs étaient amenés à mettre de côté les types de haies qui ne correspondaient pas à leurs préférences, et à classer les photos restantes. Enfin, était attendu un

positionnement de chaque photo sur une jauge de présence, qui correspond à la présence souhaitée de chaque type de haie sur le territoire (allant d'absence à très forte présence).

Étude et ciblage de l'écosystème d'acteurs autour des haies

En parallèle de la création de supports d'entretien et de la conception du guide, nous avons ciblé les acteurs pertinents à rencontrer lors des entretiens. Les haies étaient autrefois gérées et entretenues de manière relativement individuelle, par l'action de chaque propriétaire sur sa parcelle. Ce sont les premiers programmes de replantation des années 1980, menés par les départements et appuyés par l'Institut du développement forestier (Sanson et *al.*, 2019), qui donnent une dimension plus collective et politique à cette infrastructure agroécologique. En effet, les services environnementaux rendus par les haies sont nombreux, et ont intérêt à être préservés à titre collectif, d'où l'implication croissante de plusieurs acteurs sur ce sujet, notamment publics.

Lors de notre travail, nous avons distingué trois types d'acteurs :

- ***Les utilisateurs et utilisatrices de haies***

Par cette dénomination, nous incluons toute personne ou structure bénéficiant de services écosystémiques rendus par les haies. Il s'agit bien sûr des agriculteurs et agricultrices, qui bénéficient des SE à l'échelle des agroécosystèmes, en particulier ceux de support à la production (ombrage, auxiliaires de culture, brise-vent). Le service d'esthétisme paysager concerne en revanche un plus grand nombre d'acteurs : les habitant·e·s, notamment dans des territoires périurbains où les attentes en matière d'espaces boisés sont susceptibles d'être fortes, en sont directement bénéficiaires. Les élu·e·s et associations, en tant que représentant·e·s de la société civile, sont en mesure d'orienter les choix de gestion dans le sens des attentes paysagères exprimées. Dans une moindre mesure, nous citerons les entreprises travaillant dans la transformation et la valorisation du bois, en lien avec le SE de production de bois/biomasse. Toutefois, d'après nos lectures et retours d'entretiens, la filière n'est pas assez structurée pour que le bois bocager constitue une demande forte de la part des entreprises du secteur.

- ***Les gestionnaires de haies***

Les haies se situant en grande majorité sur des terrains privés, les agriculteurs et agricultrices en sont ainsi les premiers gestionnaires directs. Même dans le cas où le terrain est loué en fermage, la charge de l'entretien revient au preneur (Chambre d'Agriculture de l'Allier). Certaines haies appartiennent toutefois au domaine public, comme c'est le cas en bord de voies communales et départementales. La charge d'entretien incombe aux riverains, mais peut aussi être effectuée directement par les services communaux et départementaux pour des raisons de sécurité (Chambre d'Agriculture de l'Allier).

- ***Les personnes ayant un pouvoir de décision sur l'aménagement de l'espace au sens large***

Nous faisons ici référence aux acteurs de l'aménagement de manière générale. Les collectivités territoriales sont les principales concernées, avec des possibilités de préserver et/ou de gérer certaines haies par le biais d'outils réglementaires. C'est le cas des PLUi, des arrêtés de protection de biotope, ou encore des directives de protection et de mise en valeur des paysages, qui permettent de conserver des haies à travers la mise en place de zonages environnementaux, mais aussi de réglementer leur gestion (AFAC-Agroforesterie, 2015). Ainsi, les communes et intercommunalités sont concernées par ce type de mesure. De plus, d'autres acteurs institutionnels et associatifs en charge de l'aménagement du territoire peuvent agir en faveur ou en défaveur du bocage. Nous citerons ici les Plans de Gestion du Bocage, qui peuvent être menés à l'échelle d'une exploitation, ou de manière plus large à l'échelle d'un bassin versant ou d'un territoire défini. Ces plans visent à établir un diagnostic technique du linéaire concerné et de favoriser des pratiques d'entretien plus durables. Les organismes porteurs de ce type d'outils sont variés :

- Des intercommunalités (e.g. la Communauté d'Agglomération de Cholet) ;
- Des organismes privés intéressés par la valorisation du bocage (e.g. la SCIC ENR du Pays de Dinan) ;
- Des syndicats mixtes (e.g. les Syndicats Mixte du Trégor et du Parc Naturel Régional des Marais du Cotentin et du Bessin) ;
- Des Chambres d'Agriculture ;

- Des associations spécialisées dans la gestion du bocage (e.g. Mission Bocage, Mission Haies Auvergne).

Au vu de l'étude des différents acteurs en présence, les personnes et structures ciblées pour les entretiens ont été les suivantes :

- Les agriculteurs et agricultrices du territoire
- Les élu·e·s des communes et de l'intercommunalité (en ciblant en priorité les quatre sous-territoires d'étude)
- L'association des Amis de Saint-Saturnin (association d'habitant·e·s œuvrant au maintien et à la valorisation du patrimoine bâti et naturel)
- Des chargés de mission de la Communauté de Communes
- L'association Mission Haies Auvergne
- Le Conservatoire des Espaces Naturels d'Auvergne
- L'association Terre de Liens
- La Chambre d'Agriculture du Puy-de-Dôme

Certains acteurs ont été ciblés sur le tard, ou recommandés pendant des entretiens, comme la Fédération de Chasse du Puy-de-Dôme, la LPO, ou le SMVVA. La réalisation de plusieurs entretiens-test, avec des personnes non spécialistes du sujet, ont grandement contribué à déterminer le champ des possibles entre contraintes de modélisation (la nécessité d'obtenir des informations très quantitatives et ciblées), et contraintes des entretiens semi-directifs (la nécessité de dérouler un guide d'entretien cohérent pour les acteurs, avec des questions qui ne les mettent pas en difficulté). Au total, quatre entretiens-test ont été effectués avant de parvenir à la version finale.

2.2. Récolte de données qualitatives sur le positionnement d'acteurs au regard de scénarios territoriaux plus larges

Cette deuxième partie d'entretien, qui correspond à la moitié des questions posées, avait vocation à fournir des données plus qualitatives. Alors que l'adaptation du réseau de

haies est ici étudiée comme un type d'adaptation incrémentale, la seconde phase se place dans l'optique d'une adaptation plus transformationnelle. Dans ce contexte, l'adaptation d'une seule infrastructure agroécologique du système (ici les haies) n'est plus suffisante au regard de l'ampleur des perturbations envisagées (effets des changements climatiques, de l'érosion de la biodiversité...). Il est alors nécessaire d'envisager une transformation des activités (processus de destruction-création par exemple), voire de l'usage des terres. À ce titre, nous nous plaçons dans une perspective plus macroscopique, à l'échelle du territoire initial que constitue Mond'Arverne Communauté, et non plus à l'échelle des quatre sous-territoires.

Cette phase est ici considérée comme plus exploratoire, dans la mesure où elle repose sur des sources de données moins variées que l'étude des haies (bibliographie et conduite d'entretiens *versus* cartographie, bibliographie, étude de terrain, conduite d'entretiens). De plus, elle a été moins accompagnée par le regard des différents membres de PACSEN et repose quasi exclusivement sur notre travail de stage. Toutefois, c'est cette phase qui a permis d'aller plus en détail sur la notion de conflits d'usage et d'aborder plus concrètement la question des arbitrages collectifs. Elle a permis de monter en généralité et de permettre aux acteurs du territoire de s'exprimer sur les grands enjeux de leur territoire via une méthode prospective.

Les objectifs de cette deuxième partie d'entretien étaient les suivants :

1. Identifier les risques et vulnérabilités perçus sur le territoire en lien avec le dérèglement climatique ;
2. Identifier la présence ou non-présence de stratégies d'adaptation face à ces vulnérabilités ;
3. Sonder les préférences des acteurs parmi plusieurs scénarios d'usage des terres ;
4. Sonder les actions envisagées en lien avec chaque scénario ;
5. Identifier des tensions et conflits d'usage de l'espace latents et/ou existants.

Ces informations permettent également d'identifier les infrastructures agroécologiques et services écosystémiques associés qui constituent des enjeux pour le

territoire en matière d'adaptation. Dans cette partie d'entretien, il a été choisi de se focaliser de façon prioritaire sur les vulnérabilités et risques liés au dérèglement climatique, pour les raisons explicitées au point 1.3.1.

2.2.1. Élaboration de scénarios d'usage des terres et du guide d'entretien

Les deux premiers objectifs se sont traduits en entretien par de simples questions ouvertes, sur la perception des acteurs et les actions envisagées et menées face aux risques qu'ils identifient (voir annexe 3, questions 9-10-11). Les conflits et tensions n'ont pas systématiquement été abordés de manière directe, mais plutôt décelés dans une partie des réponses des acteurs, puis précisés par le biais de relances.

Le principal travail en amont des entretiens a été l'élaboration de scénarios d'usage des terres, qui ont, par la suite, été soumis aux acteurs (voir annexe 5). Ces scénarios ont été construits suite à l'analyse bibliographique des enjeux territoriaux, dont la synthèse est présentée en 1.2. La première étape a donc été une lecture des différents documents programmatiques à notre disposition, puis un résumé des enjeux qui ressortaient en matière d'agriculture, de paysage, de milieux naturels, et de dynamiques territoriales (voir annexe 2). Dans le même temps, nous avons recensé les différentes actions et politiques prévues dans ces documents, ainsi que le portage de ces actions, leur échéance, et enfin leur portée contraignante. À partir de ces informations, nous avons commencé à entrevoir des scénarios possibles pour le futur du territoire. Un scénario reflète souvent une grande thématique qui revient de manière récurrente dans les différents documents, et dont les actions en la matière sont relativement nombreuses, et parfois juridiquement contraignantes. Chaque scénario vise à répondre à un ou plusieurs enjeux identifiés comme cruciaux dans les diagnostics préalables à chaque plan d'actions.

L'étude des documents programmatiques (voir annexe 1) a fait ressortir cinq trajectoires possibles pour Mond'Arverne Communauté :

1) Transition alimentaire

Nous traitons ici des objectifs et projections du territoire en matière d'agriculture et d'alimentation, principalement énoncés dans le PAT (échelle extra-territoriale), le projet de territoire (échelle Mond'Arverne) et le PCAET (échelle Mond'Arverne). Ces derniers visent une augmentation de la production agricole et de la consommation alimentaire sur le territoire, par l'installation et le soutien d'activités agricoles ainsi que le développement des circuits de proximité. Est aussi évoquée dans les trois documents la durabilité des exploitations agricoles, notamment par la réduction des intrants de synthèse. Les défis envisagés pour ce scénario sont la conciliation entre le développement de l'activité agricole et la présence de biodiversité, le maintien de la vocation agricole sur le territoire au vu du non-renouvellement des générations, et la préservation des terres agricoles dans les zones où les pressions à l'urbanisation sont fortes (plaine céréalière). Les opportunités sont le maintien d'emplois et de filières économiques sur le territoire, ainsi qu'une consommation alimentaire locale.

2) Relocalisation économique

Par relocalisation économique, nous entendons le développement de zones d'emploi, d'activités (notamment artisanales et industrielles), et l'accueil de nouveaux et nouvelles arrivant·e·s. Dans son PLUi et son projet de territoire, l'intercommunalité prévoit l'accueil de 5 000 habitant·e·s entre 2018 et 2030, qui se matérialise par la construction de 3 000 logements neufs (Diagnostic PLUi, 2018). Au total, 245 hectares de consommation d'espace sont anticipés. Le principal défi pour ce scénario de relocalisation économique est la conciliation entre développement économique et résidentiel, et la préservation des milieux naturels et agricoles. Les opportunités sont le potentiel de diversification des activités économiques et la hausse démographique, générant de l'attractivité pour l'intercommunalité.

3) Attractivité touristique

Le tourisme est apparu dans tous les documents programmatiques, mais aussi dans les discours des acteurs, comme un enjeu majeur. Celui-ci est abordé autour de sites phares comme le lac d'Aydat, le Val d'Allier, et les sites archéologiques comme Gergovie. La valorisation de ces espaces est envisagée par le prisme de leur gestion et de leur mise en avant (via l'Office de tourisme intercommunal, notamment), mais aussi par la construction d'infrastructures périphériques d'accès, de stationnement, et de logement, jugées aujourd'hui

insuffisantes. Dans ce scénario, plusieurs sites ont donc une fonctionnalité résolument touristique, et plusieurs zones alentours sont aménagées dans ce sens. Les défis qui nous semblent ressortir sont la conciliation entre développement touristique et qualité des milieux naturels (en particulier pour des sites comme le Val d'Allier), la conciliation entre tourisme et agriculture (dans les zones de montagne, à proximité des prairies et estives), mais aussi la préservation du cadre de vie des habitant·e·s. Dans ce scénario, il est toutefois possible de développer des formes d'écotourisme et/ou d'agritourisme, compatibles avec les enjeux précédemment cités. Cette trajectoire laisse imaginer une meilleure valorisation des ressources locales, vectrices d'attractivité.

4) Naturalisation du territoire

Le terme de naturalisation amène deux idées différentes : d'une part celle de restaurer des milieux naturels qui ont perdu en qualité et/ou en diversité, et d'autre part celle de recréer de tels espaces. Cela peut se faire de manière délibérée (le souhait de rétablir des continuités écologiques et d'accroître la biodiversité, par exemple), ou bien du fait d'évolutions paysagères non contrôlées (e.g. dynamiques d'enfrichement sur les coteaux). Les perspectives de naturalisation sont envisagées à travers les différents zonages environnementaux (zones Natura 2000, ZNIEFF, réserves naturelles, TVB…), ainsi que l'accompagnement de pratiques agricoles existantes (e.g. MAEC à l'échelle d'un bassin versant). Les défis en matière d'usage des terres posés par ce scénario sont la conciliation entre hausse démographique anticipée et préservation des milieux naturels, et le maintien de continuités écologiques sur le territoire, notamment sur l'axe est-ouest, malgré la présence d'éléments fragmentants comme l'autoroute A75 ou la voie ferrée. Une restauration de la qualité écologique des milieux permettrait néanmoins de maintenir de nombreux services écosystémiques.

5) Transition énergétique

La dernière tendance relevée est la volonté du territoire de contribuer aux enjeux énergétiques contemporains, à la fois par la production d'une énergie décarbonée, et par la réduction des émissions de gaz à effet de serre. Le PCAET est le principal document directeur en la matière, mais cette préoccupation apparaît également dans d'autres textes. Dans ce scénario, les défis résident surtout dans la conciliation entre infrastructures

énergétiques et attractivité touristique, ainsi que dans la gestion de tensions liées à des zones de production d'énergie (notamment d'électricité). En outre, l'un des grands chantiers de la transition énergétique sur le territoire est l'évolution des pratiques culturales, en particulier au sein des exploitations d'élevage.

Chaque scénario consiste en une extrapolation de plusieurs données et objectifs du territoire portant sur une même thématique (alimentation, énergie, milieux naturels, tourisme, économie). Il est évident que le territoire a peu de chance de n'embrasser qu'une seule de ces trajectoires, et que la réalité correspond davantage à un mélange de plusieurs scénarios. L'objectif de cet exercice était surtout de mettre les acteurs face à d'éventuelles incompatibilités entre plusieurs de ces scénarios, puis de discuter sur la priorisation souhaitée pour les prochaines années. L'approche mise en avant était toujours celle de l'usage des terres, d'où la réflexion autour de la conciliation de plusieurs usages au sein d'un même espace.

2.2.2. Élaboration d'une grille de traitement et d'analyse des données

Si la partie d'entretien concernant les haies a fait l'objet d'une grille d'analyse co-construite avec les modélisateurs (voir annexe 6), qui extrait les principales informations quantitatives utiles au modèle, la seconde a nécessité la création d'une autre grille spécifique (voir annexe 7). Pour cette dernière, nous avons choisi de décortiquer notre analyse en quatre points :

- Les impacts du dérèglement climatique sur le territoire et les enjeux d'adaptation
- La caractérisation des actions d'adaptation
- Les préférences de scénarios d'usage des terres
- La caractérisation des conflits et tensions liés à l'usage des terres

Les impacts et enjeux d'adaptation perçus par les acteurs ont été récoltés de manière relativement descriptive. Nous nous sommes intéressés à :

- La localisation des impacts (échelle locale ou territoriale, voire extra-territoriale)
- Les types de vulnérabilités (d'ordre socio-économique ou écologique)
- Les implications de ces effets en matière d'IAE affectées et de SE fournis

Pour caractériser les actions d'adaptation, nous avons utilisé plusieurs critères d'analyse, trouvés dans la littérature. Il existe de nombreuses typologies sur les actions et stratégies d'adaptation, « *tant [ces dernières] sont élaborées dans des contextes socio-économiques, territoriaux, politiques, environnementaux, variés et imbriqués, au croisement de multiples situations complexes* » (Richard, 2013, p. 80). Nous nous sommes inspirés de la grille présentée dans l'ouvrage de Van Gameren et *al.* (2014), qui nous semble prendre en compte les informations cruciales qui entourent les actions d'adaptation : le contexte spatial, temporel, et humain. De plus, sont amenées dans cette grille deux autres dimensions, celles de la nature et de la conception des actions (voir *infra*), qui donnent des réponses sur la manière dont les enjeux climatiques sont compris par les acteurs et inclus dans les politiques publiques. Elles peuvent être mobilisées pour arbitrer entre un panel d'actions possibles, et donc dans une perspective d'aide à la décision, car elles possèdent un caractère opérationnel. Pour chaque action énoncée par les acteurs, nous avons donc cherché à connaître :

- L'échelle spatiale : l'action est-elle très localisée ou menée à l'échelle territoriale, voire extra-territoriale ?
- L'échelle temporelle : l'action est-elle menée à court terme (moins de cinq ans), moyen terme (cinq à dix ans), ou long terme ?
- La nature des actions : l'action est-elle considérée comme *verte* (basée sur les écosystèmes), *grise* (basée sur de l'ingénierie et la mise en place d'infrastructures et de technologies), ou *soft* (d'ordre institutionnel, politique, financier) ?
- Les acteurs associés : les acteurs porteurs de l'action sont-ils publics et/ou privés ? Quelles sont leurs relations ?
- La conception des actions : l'action est-elle *flexible*[13] ? Génère-t-elle des *co-bénéfices*[14] ? Est-elle *sans-regret*[15] ?

[13] Une action ou stratégie flexible peut être revue à mi-parcours, et laisse la possibilité de basculer vers une autre stratégie si celle-ci s'avère inadaptée.

[14] Les co-bénéfices désignent tous les effets positifs en plus de ceux du secteur de l'adaptation au changement climatique, par exemple des effets positifs sur la biodiversité ou sur la santé humaine.

[15] Une action sans-regret génère des effets positifs dans tous les scénarios climatiques, même en l'absence de changements climatiques (Prutsch et *al.*, 2010 ; Van Gameren et *al.*, 2014).

Sur la question des scénarios, la grille avait vocation à retranscrire les trajectoires jugées les plus prioritaires à mettre en œuvre, ainsi que leur priorisation. En ce qui concerne les actions mises en œuvre pour la réalisation des scénarios, nous avons utilisé les mêmes critères de caractérisation que ceux énoncés dans le point précédent.

Enfin, les tensions et conflits d'usage ont été appréhendés conjointement avec l'approche des services écosystémiques, et par des critères d'analyse énoncés et développés par Torre et *al.* (2016). Pour la première, nous avons cherché à savoir quelles étaient les infrastructures agroécologiques concernées par les tensions et conflits (le cas échéant) ainsi que les services écosystémiques qui y sont associés. Pour la deuxième, nous avons cherché à savoir l'objet de la tension ou du conflit (maîtrise du foncier, contestation de projets d'aménagement, externalités négatives générées par des activités…), ainsi que les acteurs en présence (acteurs publics, entreprises, particuliers…).

2.3. Points de vigilance concernant l'analyse des résultats

Il nous semble que les résultats développés dans la partie suivante doivent être appréhendés au regard de plusieurs contraintes. Ces dernières sont d'ordre pratique et méthodologique, et expliquent, d'une part, une taille limitée de l'échantillon final, et de l'autre, des réponses parfois incomplètes de la part des acteurs. Si nous pensons que les personnes rencontrées sont très pertinentes au regard de la problématique posée, et que chacun des échanges a été riche, l'analyse finale doit être fortement nuancée d'un point de vue quantitatif.

2.3.1. Des difficultés d'ordre pratique

Si les entretiens devaient initialement être menés sur une période de cinq mois, la phase de conduite n'a pu démarrer qu'en juillet, pour se concentrer durant l'été et le mois de septembre. De plus, si certains publics ont été faciles à rencontrer, comme les maires ou les acteurs techniques, d'autres ont été beaucoup plus compliqués à joindre, comme les agriculteurs et agricultrices. Cela s'explique en grande partie par la période des entretiens, qui est peu propice à la rencontre du fait de la charge de travail de ces acteurs. Par conséquent,

les résultats développés présentent une vision à la fois politique et technique des enjeux auxquels l'on s'intéresse, mais manque réellement de celle du monde agricole, un acteur pourtant majeur sur la question du bocage. Cela se voit autant dans les services écosystémiques d'importance mis en avant que sur les préférences de scénarios. En outre, d'autres aléas sont venus encore réduire l'échantillon, notamment concernant la catégorie des « habitant·e·s ». Des entretiens prévus avec deux associations et deux habitant·e·s rencontré·e·s sur le terrain ont finalement été annulés[16], bien que des échanges téléphoniques et physiques plus informels ont été menés avec ces personnes, dont nous nous inspirerons dans notre analyse.

2.3.2. Des difficultés d'ordre méthodologique

Dans le cas où les acteurs ont accepté la demande d'entretiens, des difficultés d'ordre méthodologique ont pu être observées, venant ici aussi nuancer la portée des résultats finaux.

La première est la densité du guide d'entretiens, autant sur sa longueur que sur la diversité des renseignements qui sont demandés. Le nombre d'informations recherchées (données sur les haies pour la modélisation, actions d'adaptation, préférences de scénarios d'usage des terres) était, *a posteriori*, sans doute trop ambitieux. En pratique, nous avons parfois dû sélectionner les informations à demander au vu des contraintes de temps des acteurs. Si nous avions voulu obtenir des réponses très détaillées pour la totalité du guide, il aurait fallu dans l'idéal compter sur une durée proche de deux heures ou deux heures trente, quand la majorité ne disposait en moyenne que d'une heure, voire moins. En outre, peu d'acteurs étaient à l'aise pour aborder en détail tous les sujets, donnant des réponses parfois hétérogènes.

La seconde difficulté était de concilier les attentes de la modélisation avec le format d'un entretien semi-directif, dans lequel les personnes interrogées ne sont pas complètement dirigées. Plusieurs d'entre elles n'étaient pas à l'aise avec les exercices de classement, de hiérarchisation, et de notation qui étaient demandés, et qui sont pourtant nécessaires pour produire des données quantitatives. Nous avons donc régulièrement adapté les questions afin

[16] Pour des raisons médicales d'une part, et sans justification dans le cas d'une habitante.

de retirer les informations principales, mais les réponses ne sont donc pas toutes de la même précision, et formulées selon les mêmes termes.

Finalement, notre échantillon est constitué de neuf personnes (huit entretiens), composé d'un agriculteur, de trois maires, et de cinq acteurs techniques. L'agriculteur est un arboriculteur en agriculture biologique, contacté via l'association Terre de Liens, qui est assez sensible aux questions environnementales, et notamment au sujet des haies. Les trois communes enquêtées, qui représentent bien la diversité du territoire par leurs tailles et enjeux respectifs, nous ont semblé adaptées pour pouvoir retranscrire le regard d'élus locaux. Enfin, les cinq acteurs techniques nous ont permis d'aborder la question de l'adaptation sous plusieurs angles : agricole avec la Chambre d'Agriculture et la Mission Haies, écologique avec le CEN Auvergne, ainsi que paysage et urbanisme avec la Communauté de Communes.

3. ANALYSE DES RÉSULTATS

3.1. Les haies sur le territoire de Mond'Arverne : état des lieux et perspectives pour une infrastructure agroécologique multi-services

3.1.1. Un état des lieux des haies sur le territoire de Mond'Arverne : analyse par les services écosystémiques

Les services d'importance liés aux haies d'après les entretiens

Le premier objectif de nos entretiens était de sonder les préférences des acteurs interrogés concernant une série de services écosystémiques préalablement identifiés, qui représentent autant d'enjeux liés aux haies pour ce territoire.

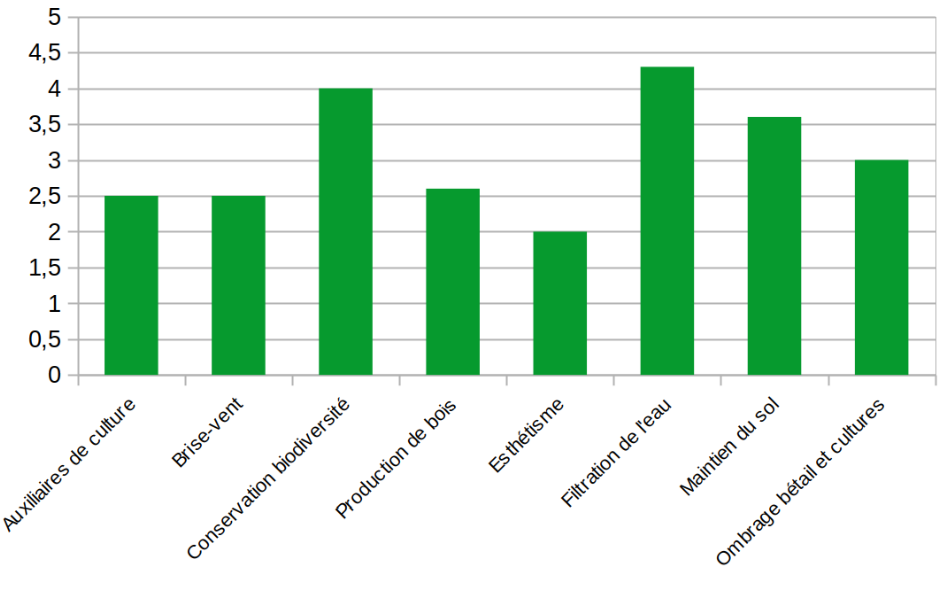

Figure 11. Importance moyenne des six services écosystémiques étudiés sur une jauge de 0 à 5.

Les services les plus mis en avant sont la conservation de la biodiversité, la filtration de l'eau, et le maintien du sol, ce qui n'est pas très étonnant au vu de la composition de l'échantillon. En effet, notre panel comporte un nombre important d'élus, qui s'expriment sur les services les plus demandés par leurs administré·e·s. La conservation de la biodiversité constituant une préoccupation croissante au sein de la société civile, il paraît logique que cela se ressente dans le discours des représentants politiques du territoire. De plus, les maires interrogés sont ceux de communes où la biodiversité fournie par les milieux naturels correspond justement à ce que les nouveaux et nouvelles arrivant·e·s viennent chercher. Dans ces communes périurbaines où la plupart des adultes en emploi travaillent sur la métropole clermontoise, la pérennisation d'une certaine qualité de l'environnement naturel, gage de qualité de vie, constitue une attente explicite, comme l'illustre le témoignage suivant : « *Oui, les habitants sont à Saint-Saturnin pour l'environnement aussi. Ils veulent préserver l'identité du village, par les maisons, mais aussi l'environnement. Le patrimoine vert, naturel. Si vous leur proposez un paysage sans haies, vous allez avoir un succès limité.* » (Maire de Saint-Saturnin, 2021).

D'autres services révèlent des caractéristiques moins sociétales, et plus spécifiques aux caractéristiques physiques du territoire. La filtration qualitative de l'eau, arrivée en tête, constitue par exemple un enjeu très bien identifié pour la Communauté de Communes, qui dispose de nombreux cours d'eau et bassins versants. Cette problématique est d'ailleurs portée par une instance spécifique, le SMVVA, qui mène des actions d'amélioration de la qualité de l'eau. Une partie du territoire étant constituée d'une zone de plaine alluviale céréalière, avec un mode de culture basé sur des apports fréquents d'intrants de synthèse, la nécessité de filtrer les polluants est exprimée par l'ensemble des acteurs. Le troisième grand service cité est celui du maintien du sol, dans un contexte de zones de coteaux particulièrement sensibles aux effets d'érosion. Sur ces espaces, l'implantation et la gestion des haies ne constituent pas uniquement des préférences mais parfois une nécessité, pour limiter les dégâts matériels sur les cultures et les habitations, causés par les coulées de boue et/ou les inondations. Les services mis en avant reflètent donc à la fois des préoccupations sociétales et des particularités très locales.

Cependant, plusieurs éléments viennent nuancer la véracité de ce classement, qui peuvent être expliqués par la taille et la composition de notre échantillon (voir le point 2.3). Le service de brise-vent apparaît comme l'un des moins bien notés chez les acteurs, alors

qu'il correspond pourtant à la première motivation de plantation de haies pour le monde agricole (Mission Haies Auvergne, retranscription d'entretien, 2021). Il en va de même pour l'esthétisme, situé en dernière position, et qui s'est rarement retrouvé au centre des échanges. Pourtant, l'esthétisme peut être l'un des principaux moteurs d'engagement au sujet des haies pour certains acteurs, en particulier pour la société civile. Un échange téléphonique avec l'association des Amis de Saint-Saturnin[17], qui a constitué le seul témoignage de ce type de public, nous a confortés sur cette piste. Cette association de préservation du patrimoine bâti et naturel organise, en coopération avec une autre association de la commune, des évènements de sensibilisation et de réflexion sur les haies, en l'occurrence par l'invitation d'un chercheur spécialisé sur le sujet, ou par la visite de la ferme d'un agriculteur ayant effectué des plantations. Dans cette perspective, les haies sont appréhendées en premier lieu comme un élément du patrimoine à conserver, voire à restaurer, bien que d'autres fonctions puissent être mises en avant.

Si les services qui ont été discutés furent sélectionnés en amont des entretiens, nous avons laissé l'opportunité aux acteurs interrogés d'évoquer d'autres services qui leur semblent importants. A souvent été évoquée la fonction d'anti-dérives des produits phytosanitaires fournie par les haies, mais aussi celle de pare-congères[18] du côté des municipalités, qui sont directement responsables de l'entretien de certaines voies de circulation.

Les services fournis par les haies : combiner une approche globale et à l'échelle de l'exploitation

Notons que deux acteurs n'ont pas souhaité noter ces services selon un ordre d'importance : la Mission Haies Auvergne et la Chambre d'Agriculture. Au sein du panel d'acteurs que nous avions initialement identifié, il s'agit des acteurs les plus techniques sur notre sujet, qui accompagnent directement le monde agricole dans la plantation et la gestion de leurs haies. Le travail qui leur était demandé se trouvait en décalage avec la vision du bocage qu'ils tentent de véhiculer auprès des propriétaires, qui est une vision résolument

[17] Un échange assez général sur les haies a été effectué par téléphone, une rencontre plus approfondie associant une autre structure ainsi qu'un agriculteur de la commune était prévue, mais n'a pas pu avoir lieu pour des raisons médicales du côté des enquêtés.

[18] Les haies peuvent éviter la formation de congères sur la chaussée et ses abords.

multi-fonctionnelle, basée sur la triple performance (Ministère de l'Agriculture et de l'Alimentation, 2015) :

> « D'abord la performance économique, donc là on est sur le système de production : c'est utile pour la culture et pour les vaches, on peut le chiffrer. Deuxième performance, la performance environnementale, on répond à des enjeux de corridors écologiques, de biodiversité etc. Dépollution de l'eau, de l'air, de captation du carbone, de production d'une ressource renouvelable. Et enfin la performance sociale, il y a une attente aujourd'hui d'un paysage plus vert, d'un système de production qui mette en place des pratiques qui puissent protéger. » (Mission Haies Auvergne, 2021).

Dans les entretiens, est bien mis en avant que la performance économique doit être l'argument prioritaire auprès du monde agricole (principal gestionnaire de haies), autrement dit les intérêts fonctionnels de la haie au sein d'un système de production. En termes de services, cela correspond aux services de support à la production agricole, qui sont les premières motivations pour planter ou conserver des haies. En parallèle, les préoccupations environnementales et sociétales constituent des bénéfices supplémentaires, mais sont rarement des raisons suffisantes en soi pour amorcer une réflexion sur le bocage à l'échelle d'une exploitation. Toutefois, les acteurs techniques nous précisent que si les services de support sont souvent les plus recherchés, ils ne sont pas mis en avant « par défaut ». Une réflexion à l'échelle de la parcelle est indispensable, afin de proposer une intégration du bocage qui répondra au mieux aux attentes de l'agriculteur ou de l'agricultrice :

> « Nous l'idée ce n'est pas de prioriser les choses, on a vocation à présenter à un agriculteur l'intérêt global de la haie, et que derrière, lui puisse l'appliquer à son exploitation. Parce que quand on voit la filtration de l'eau, ça nous paraît important pour tout ce qui est problèmes d'érosion, des anti-dérives mais aussi perte de sols, des pentes où l'on peut perdre des terrains. Donc ce sont des choses importantes. Mais nous à notre niveau, dire est-ce que ça c'est plus important que les auxiliaires, ou que la production de bois, ça va dépendre du secteur. Une exploitation en pente, on va être plutôt là-dessus, sur l'érosion des sols, et qu'est-ce qu'on peut faire pour aujourd'hui aider à la fois sur les pratiques culturales et sur la plantation de haies. » (Chambre d'Agriculture du Puy-de-Dôme, 2021).

Dans cette vision de la haie et de l'accompagnement de celles et ceux qui en ont l'usage, aucun service n'a plus d'importance qu'un autre dans l'absolu. Par ailleurs, la position de ces deux acteurs sur la notion de service est ambivalente : s'ils conçoivent l'intérêt pédagogique de cette approche pour faciliter les échanges autour des « bienfaits » de

la haie[19], et l'utilisent d'ailleurs eux-mêmes[20], la difficulté à évaluer et à quantifier les services demeure un frein à une mobilisation plus poussée de ce concept.

De manière générale, les questions sur les services d'importance se sont surtout avérées pertinentes pour des acteurs qui gèrent des haies de manière directe, et que nous avons peu rencontré du fait des contraintes explicitées au point 2.3. Toutefois, les échanges sur ce sujet ont permis de faire ressortir une variété de positionnements, et ont validé la pertinence du choix des six services d'étude.

Les haies pour s'adapter aux changements globaux ?

Outre la diversité de services fournis, la haie est aussi identifiée localement comme un levier d'adaptation aux changements globaux, au regard de problématiques spécifiques. Concernant l'érosion de la biodiversité, la fonction de corridor écologique permise par le bocage est revenue à plusieurs reprises, les acteurs étant conscients de la fragmentation présente entre les différents habitats écologiques, notamment du fait des nombreuses et larges voies de circulation anthropiques. Restaurer un linéaire de haies apparaît donc comme indispensable pour limiter l'érosion de la biodiversité : en garantissant une présence régulière du bocage sur le territoire (Trame Verte et Bleue), notamment en zone urbaine (Trame Verte Urbaine), mais aussi en sanctuarisant les haies qui fournissent les services les mieux identifiés et jugés les plus nécessaires (Diagnostic PLUi, 2018).

Les haies semblent aussi être perçues comme des leviers d'adaptation au dérèglement climatique, autant du côté du monde agricole que de celui des collectivités territoriales. Par exemple, une intensification des précipitations est anticipée, dont les effets pourraient aggraver ceux déjà présents dans les zones de coteaux (voir *supra*). Dans ces cas de figure, les pouvoirs publics locaux peuvent prendre l'initiative de travailler directement sur ce sujet, en plantant des haies et en réfléchissant à un aménagement bocager. De plus, les haies peuvent être perçues comme un élément limitant les baisses de productivité causées par les effets du dérèglement climatique. Ces dernières sont variables selon le type de productions agricoles, mais il s'agit notamment de la hausse de fréquence des canicules, de l'érosion

[19] La notion de disservice écosystémique, en revanche, n'est pas abordée.

[20] À l'aide de supports visuels un peu similaires aux nôtres.

éolienne causée par des vents plus fréquents et violents, ainsi que des gelées tardives perturbant la phénologie des cultures (voir 3.2.1.).

L'état actuel du linéaire, les préférences de types de haies et leur répartition spatiale

Par le biais des entretiens, nous avons aussi cherché à savoir quels types de haies étaient les plus observés par les acteurs sur le territoire, si ce réseau leur paraissait satisfaisant, et s'il permettait de fournir les services d'importance précédemment cités. Notons que si les enjeux et services liés aux haies sont bien identifiés, le lien avec le type de haies ne leur a pas paru pas si évident. De ce fait, les réponses que nous avons obtenues sont assez peu satisfaisantes : les acteurs ont souvent une vision approximative de l'état du réseau de haies et de sa capacité à pourvoir des SE.

Concernant l'état actuel du linéaire sur la Communauté de Communes, tous les types de haies sont revenus, avec une récurrence un peu plus forte des haies hautes, bien visualisées en bord de cours d'eau. Pour la question qui met en lien ce réseau avec les services, la plupart des acteurs n'étaient pas en mesure de répondre, excepté l'agriculteur interrogé qui possède une idée assez claire de l'usage qu'il souhaite avoir des haies, et des types nécessaires à implanter pour cela.

En revanche, les acteurs arrivent davantage à se projeter dans une vision idéale du réseau de haies, et à exprimer leurs préférences vis-à-vis des types qui le composent. De manière générale, le stade « pas de haies » a été unanimement rejeté. Le plus plébiscité est la haie haute pluri-strates, par la diversité des usages qu'elle permet (valorisation intéressante par l'agriculteur·rice, intérêts écologiques). Arrivent ensuite les haies basses ponctuées et haies anciennes, qui constituent souvent des éléments structurants du paysage dans des zones de cultures céréalières, où la tendance à la réduction du linéaire est constatée. En revanche, concernant la répartition idéale de ces différents types de haies sur le territoire, les résultats sont plus hétérogènes, si bien qu'aucune tendance particulière ne peut être soulignée, mis à part la volonté très claire de limiter fortement des paysages d'« *open-fields* ». Pour les acteurs ayant accepté de répondre à cette question (cinq personnes), les haies les plus présentes devraient être les haies hautes et les alignements d'arbres. Une présence allant de moyenne à forte est envisagée pour les haies basses et basses ponctuées.

Il nous semble que les réponses limitées et vagues obtenues pour ce point sont révélatrices de plusieurs limites d'application de la notion de services écosystémiques à notre cas d'étude :

- La difficulté pour des acteurs non scientifiques de mettre en lien la fourniture de services écosystémiques avec les caractéristiques de l'infrastructure agroécologique concernée ;

- La faible pertinence de mettre en parallèle IAE et services à des échelles trop larges (Communauté de Communes ou sous-territoires) : si l'agriculteur interrogé était en mesure d'exprimer des préférences de haies souhaitées sur sa parcelle au vu des services recherchés, il s'avère compliqué, voire impossible, d'effectuer une telle projection à l'échelle intercommunale.

Dans la perspective de réfléchir très concrètement à des aménagements bocagers fournissant une diversité de services, un focus à une échelle beaucoup plus restreinte est donc nécessaire. Pourtant, cela peut être un problème pour certains services plus « transversaux », qui pourraient nécessiter une coordination à plus large échelle, par exemple la séquestration carbone ou la fonction de corridor écologique. La question de l'action collective paraît alors primordiale pour permettre ce lien entre échelon territorial et zones ultra-locales.

3.1.2. Agir sur les haies et penser leur gestion : analyse par les acteurs en présence

Si l'approche par services a permis de relever les principaux enjeux liés aux haies sur le territoire pour les différents acteurs, qui peuvent ensuite être traduits en types de haies à favoriser, nous allons ici nous intéresser à la manière dont les acteurs du bocage agissent et se coordonnent entre eux à l'échelle de notre territoire d'étude, mais aussi comment cette IAE est prise en compte dans les politiques locales, notamment dans une perspective d'adaptation à des changements globaux.

Les haies : une IAE découpée entre plusieurs acteurs et secteurs d'action publique

Commençons par préciser que les haies ne font l'objet d'aucune structure institutionnelle ni même de réelle politique dédiée à l'échelle nationale. Après une perception négative des haies durant le remembrement et de nombreuses incitations à l'agrandissement des parcelles, les préoccupations environnementales croissantes ont ramené cette IAE dans le débat public. Cependant, cette prise en compte s'est faite de façon très sectorielle, en s'appuyant sur des organismes compétents sur certains aspects relatifs aux haies (DRAAF, DREAL, AFB…), qui incluent ces dernières dans les politiques qu'ils mènent. Les protections au titre de la loi paysage, les périmètres de protection de captage d'eau potable, les arrêtés de protection de biotope, sont autant d'exemples d'outils qui visent à préserver les haies pour certains services rendus, ici respectivement l'identité paysagère, la filtration de l'eau, et la conservation de la biodiversité (Le Guillou, 2020).

À l'échelle locale, nous retrouvons aussi cette répartition par thématiques, notamment auprès de structures chargées de mettre en œuvre des politiques nationales ou européennes. En ce qui concerne Mond'Arverne Communauté, le SMVVA participe par exemple à la mise en place du Schéma d'Aménagement et de Gestion des Eaux Allier Aval, qui décline plusieurs mesures en lien avec le maintien de la qualité de l'eau décidées à l'échelle nationale. Sur la question de la biodiversité, les gestionnaires de zones Natura 2000 (ici le CEN Auvergne et la LPO) sont garants du respect des directives européennes Oiseaux et Habitats. Dans les deux cas, les haies sont une composante de la politique et des actions mises en œuvre, mais elles n'en sont pas le sujet central.

Au bout du compte, peu d'acteurs sont réellement en mesure d'agir sur les haies de façon intégrée, comme nous le précisait l'association Mission Haies Auvergne. Cette structure, qui accompagne les propriétaires et gestionnaires de haies, fait figure de référence en Auvergne pour son rôle de sensibilisation sur les intérêts du bocage et pour son appui technique auprès des différents publics. D'après nos recherches, cet acteur non-institutionnel est le seul à développer une vision globale de la haie sur le territoire, et agit en tant que médiateur territorial au sein d'une diversité d'acteurs :

> « La haie est au Ministère de l'Écologie pour l'eau, le paysage, la captation carbone, la biodiversité. Elle est au Ministère de l'Agriculture pour la biodiversité, le changement

climatique, l'adaptation des systèmes de production, la biomasse, la forêt. Nous sommes partout et nulle part. C'est une énorme faiblesse, mais c'est aussi ce qu'on veut à tout prix garder. » (Mission Haies Auvergne, 2021).

La figure 12 ci-dessous illustre le cloisonnement des principaux enjeux qui entourent les haies, en considérant ces derniers par le prisme des services écosystémiques étudiés dans le projet.

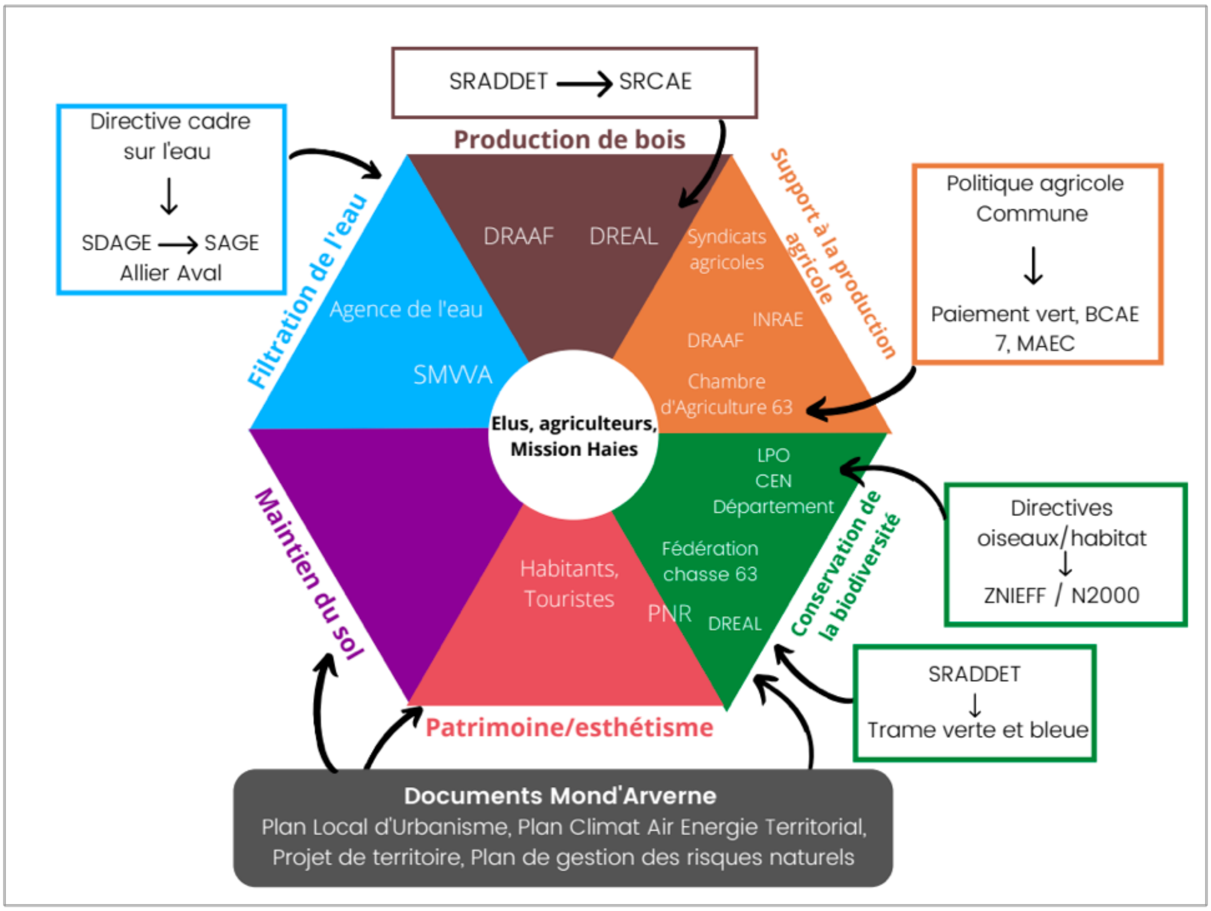

Figure 12. La haie sur le territoire de Mond'Arverne Communauté : synthèse des principaux acteurs et cadres législatifs en présence.

Ce que cette répartition des enjeux au niveau institutionnel nous amène à supposer, c'est que ce contexte ne favorise pas une approche multi-fonctionnelle de la haie, ou dans d'autres termes, une approche qui viserait à concilier différentes attentes en matière de services. Précisons toutefois qu'une vision plus systémique de la haie a tendance à s'institutionnaliser davantage, notamment grâce au travail de lobbying d'acteurs au niveau national comme l'AFAC-Agroforesterie, qui se solde par exemple par la création du Pôle

Régional Arbres hors forêt en Auvergne-Rhône-Alpes (une instance dédiée à la question du bocage à l'échelle régionale).

Les capacités d'action des acteurs sur les haies

Outre le découpage institutionnel, les haies se trouvent au croisement de plusieurs cadres juridiques et réglementaires, qui ont une influence sur la capacité d'action des différents acteurs. Ces cadres vont venir préciser la manière dont doivent se dérouler l'entretien, la gestion et la préservation des haies, et s'articulent essentiellement autour de deux aspects : les réglementations relatives aux droits de propriété, et celles relatives à des enjeux plus locaux.

Concernant la propriété, plusieurs composantes sont à distinguer (Ostrom et *al.*, 2009 ; McGinnis, 2011, pp. 177-178) : la nature du bien, le système juridique et institutionnel déterminant les droits de propriété, les ayants-droits et les usagers. Les haies constituent un bien de nature variable : elles peuvent relever du domaine public (communal, intercommunal, départemental) comme du privé (terres agricoles, particuliers). Ainsi, les droits de propriété peuvent faire référence à l'un de ces deux domaines, mais agrègent également d'autres réglementations, notamment en lien avec la vocation du foncier sur lequel se trouve l'IAE. Par exemple, dans le cas du foncier agricole, un·e agriculteur·rice louant ses terres en fermage est chargé·e de l'entretien et de la valorisation des haies, mais doit obtenir l'aval du propriétaire pour des opérations plus définitives, comme des arrachages.

En outre, les haies peuvent se situer dans des zones ayant des enjeux paysagers, démographiques et fonciers très variables, nécessitant des modes de gestion et de protection adaptés. En conséquence, toutes les haies ne sont pas concernées par les mêmes textes juridiques et par les mêmes réglementations, comme l'illustre la figure 13.

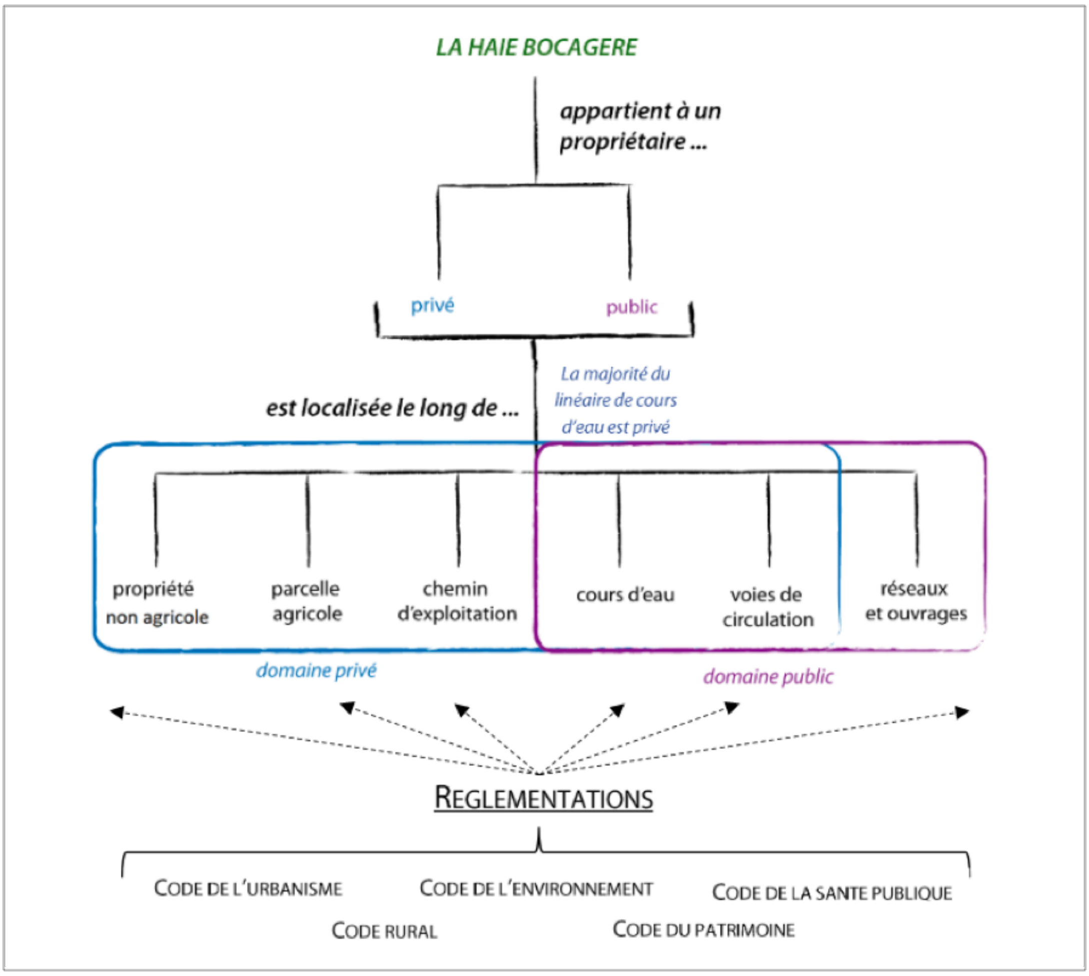

Figure 13. Schéma conceptuel représentant la complexité de l'aspect réglementaire de la haie. Source : Le Guillou, 2019, p. 91.

Néanmoins, les capacités d'actions des acteurs ne sont pas uniquement déterminées par le contexte juridique et réglementaire en vigueur, même si ce dernier est un élément facilitant ou limitant selon les cas. La nature des relations entretenues avec les autres acteurs du bocage, l'accès à certaines informations, ainsi que les ressources économiques, sont autant d'éléments qui dessinent la marge de manœuvre allouée à chaque acteur.

La figure 14 ci-dessous recense les principaux leviers d'actions qui ont été exprimés par les personnes interrogées. Pour les trois types d'acteurs que nous distinguons (techniques, politiques, et monde agricole), les plus grands leviers sont « directs » : il s'agit ici de la possibilité de planter ou de gérer soi-même des haies, ou d'accompagner d'autres individus ou structures dans cette démarche. En parallèle, on observe un autre type de levier d'action plus « indirect », qui consiste à avoir un rôle d'influence sur la question du bocage, que ce

soit par des activités de sensibilisation ou de lobbying. Enfin, d'autres manières d'agir sur le réseau de haies sont plus spécifiques à la catégorie d'acteurs à laquelle on s'intéresse : des choix de gestion personnels pour le monde agricole, ainsi que la mobilisation d'outils réglementaires, pour les collectivités dotées de compétences sur certaines thématiques (e.g. l'urbanisme).

LEVIERS D'ACTION		
**Acteurs politiques**	**Acteurs techniques**	**Monde agricole**
Plantation de haies sur domaine public (communal/intercommunal)	Accompagnement et formation à des pratiques de gestion durable	Plantation de haies sur les parcelles : choix des essences, du type de haies, de son implantation…
Sensibilisation du grand public aux rôles des haies	Activité de lobbying auprès des pouvoirs publics (échelle locale, régionale et nationale)	Sollicitation de structures pour de l'appui technique à la gestion
Protection de haies grâce aux outils réglementaires	Recherche et développement sur les possibilités de valorisation du bocage	Choix du mode de valorisation de la haie
Formation des agents à des pratiques de gestion durables	/	/

Figure 14. Les leviers d'action sur les haies ressortis lors des entretiens.

Si certaines des personnes interrogées se sentent en capacité d'agir sur le réseau de haies (la majorité de l'échantillon), d'autres n'expriment pas la même opinion, ou bien fournissent une réponse double. Le facteur qui limite le plus l'action semble ici être de l'ordre des relations sociales entre acteurs, et de la défiance qui peut entourer les discussions autour du bocage (voir figure 15). Une défiance de la part du monde agricole, d'une part, pour qui les haies sont encore trop souvent associées à des préoccupations éloignées de celles de l'exploitant·e (e.g. conservation de la biodiversité). D'autre part, les acteurs politiques se sentent parfois mal à l'aise, voire illégitimes, pour dialoguer avec des acteurs privés sur la manière de gérer leurs biens et leur activité. En outre, des problèmes d'accès à l'information et aux connaissances sont pointés, aussi bien du côté des acteurs privés que publics (connaissances sylvicoles, juridiques...). Enfin, des aspects plus pratiques peuvent décourager

les gestionnaires à agir sur les haies, comme des capacités financières et d'ingénierie insuffisantes, ou un matériel d'entretien inadéquat.

FACTEURS D'INACTION		
**Acteurs politiques**	**Acteurs techniques**	**Monde agricole**
Coût financier d'entretien à long terme	Sentiment de défiance sur la question du bocage de la part du monde agricole	Manque de matériel d'entretien adapté
Dialogue difficile avec les propriétaires privés	Décalage entre les zones d'intervention de la structure et celles des haies (gestion de sites déterminés *versus* linéaires qui les séparent)	Potentiel agronomique des haies mal identifié
Manque de matériel d'entretien adapté, de mutualisation des équipements	/	Incertitude liée à l'intégration de la haie dans le système de production
Manque de connaissances juridiques et botaniques concernant les haies dans le domaine public	/	Manque de connaissances en sylviculture (peu transmises dans les formations agricoles)

Figure 15. Les freins à l'action sur les haies relevés par les acteurs en entretien.

Ce point avait donc vocation à replacer le contexte des acteurs en présence autour du bocage pour le territoire de Mond'Arverne, ainsi que leurs éventuels leviers d'actions. À l'échelle de l'intercommunalité, les acteurs sont globalement répartis par champs de compétences et d'expertise, comme c'est le cas à l'échelle nationale, bien que la présence d'un acteur clé sur le territoire permette d'intégrer une vision plus systémique de l'IAE dans les plans de gestion. En ce qui concerne leurs capacités d'action, nous avons vu que ces dernières étaient très variables, autant sur la marge de manœuvre allouée que sur le type d'actions effectuées. Elles sont déterminées par les normes juridiques et relatives aux enjeux locaux en vigueur, la nature des relations entretenues entre acteurs du bocage, l'accès à une diversité d'informations, ainsi que les ressources économiques disponibles.

3.1.3. La place des haies dans les stratégies d'adaptation de Mond'Arverne

Après avoir dressé un tableau des enjeux écologiques et sociaux qui entourent le bocage sur notre territoire d'étude, nous avons souhaité regarder de plus près la manière dont cette IAE était intégrée, ou non, dans les stratégies d'adaptation[21]. Les éléments que nous avons choisi d'étudier pour appréhender ces stratégies sont les documents programmatiques, qui illustrent les choix et directions pris à l'échelle du territoire intercommunal pour les prochaines années, et les dires des acteurs rencontrés, qui reflètent davantage des stratégies individuelles ou collectives, à l'échelle de sous-territoires ou entités (communes, collectifs non-institutionnels).

La place des haies dans les documents de planification territoriale

Lors de l'analyse bibliographique portant sur différents documents programmatiques (voir annexe 1), nous avons relevé la présence des haies à quatre reprises.

1) Dans le *PCAET*, les haies apparaissent explicitement dans la fiche action (« *Renforcer les corridors écologiques* »), au sein de la thématique « *Agriculture et consommation* ». La volonté exprimée est de mailler les parcelles agricoles de haies avec un double objectif : améliorer la connectivité d'un point de vue écologique et la qualité des sols pour les exploitations. Toujours au sein de la même thématique, les haies sont aussi évoquées de manière plus indirecte via « *la création de zones tampon* » entre les parcelles et les habitations, avec une visée d'anti-dérives des produits phytosanitaires.

2) Le *PLUi* de la Communauté de Communes est encore en cours d'élaboration, mais plusieurs documents, notamment de diagnostic, sont consultables. Dans le diagnostic des enjeux territoriaux, les haies sont reliées à deux éléments principaux. Le premier est la dimension paysagère du bocage qui concerne uniquement l'est du territoire, via sa fonction traditionnelle de séparation des parcelles (voir sur ce point la description des entités paysagères, en partie 1.2.). Le deuxième élément cité est le rôle de corridor écologique des

[21] Par le terme de « stratégie », nous entendons ici au sens large tout plan d'actions, politique, ou trajectoire relevé dans l'analyse.

haies, décrit par la Trame Verte et Bleue dans le SRADDET (élaboré à l'échelle régionale, mais avec des déclinaisons dans les PLUi locaux).

3) Dans le *SCoT* du Grand Clermont (auquel Mond'Arverne est adhérente), les haies sont, de la même manière, abordées à travers leur rôle de corridor écologique. Toutefois, elles sont évoquées de façon plus notable en tant qu'entités paysagères ayant un impact positif sur l'attractivité du territoire, et dont la place dans les aménagements touristiques doit être prise en compte et intégrée.

4) Le SMVVA est porteur de deux *contrats territoriaux* concernant le périmètre géographique de Mond'Arverne. Dans le Contrat Territorial des 5 Rivières, les haies sont évoquées dans le premier volet portant sur la préservation de la qualité de l'eau. Une fiche action spécifique leur est d'ailleurs consacrée (« *Soutenir la plantation et la gestion durable des haies* »), qui « *vise à accompagner le public agricole mais également les collectivités et les propriétaires fonciers dans l'objectif de préserver et d'améliorer la trame bocagère* » (Contrat Territorial des 5 Rivières, 2020, p. 7). Elles sont aussi présentes au sein d'une deuxième fiche figurant dans un autre volet du contrat, autour de la fonctionnalité des milieux aquatiques. Le service écosystémique recherché est celui de stabilisation des berges, fourni par les ripisylves (« *Restauration de la végétation rivulaire* »). Enfin, dans le Contrat Territorial du Charlet, un réseau bocager prévoit d'être créé en guise de zone tampon, pour diminuer le transfert des polluants.

La place des haies dans les stratégies individuelles et collectives des acteurs rencontrés

Les témoignages d'acteurs donnent un panorama assez large de la manière dont cette IAE peut être prise en compte dans une perspective d'adaptation. Nous avons choisi d'en restituer trois exemples, selon les types d'acteurs.

- **Pour des acteurs techniques : exemples de la Mission Haies et du CEN Auvergne**

Lors de notre échange avec l'association Mission Haies, cette dernière a fortement mis en avant le potentiel des haies dans l'adaptation au dérèglement climatique, en particulier pour le monde agricole qui en subit directement les effets. Auprès de ce dernier, la structure met notamment en avant les caractéristiques suivantes :

> « L'arbre a des rôles absolument essentiels : c'est un tampon. À l'ombre d'un arbre, il fait jusqu'à 10 degrés de moins l'été en plein jour, et il fait 3 degrés de plus la nuit. Il fait moins froid et moins chaud. [...] Des experts du GIEC ont adapté leurs modèles au Massif Central, et ils disent des canicules de plus en plus fortes, oui, de l'irrégularité de l'eau de plus en plus forte, oui, des hivers plus doux, oui mais, potentiellement toujours aussi froid. […] L'arbre est donc un tampon avec l'effet brise-vent, par rapport à l'eau, l'optimisation de l'irrigation aussi. […] Donc de nouveau la haie va nous permettre de ralentir le vent, d'irriguer le bon jour, et limiter le dessèchement. […] Enfin, vis-à-vis des écosystèmes, la haie permet une circulation dans un contexte de changements pour les écosystèmes, afin qu'ils s'adaptent, qu'ils puissent ajuster leurs habitats.» (Mission Haies Auvergne, 2021).

La haie apparaît donc comme un élément du système de production à intégrer permettant de le rendre plus résilient vis-à-vis des variations climatiques, mais aussi d'accompagner d'autres écosystèmes dans leur propre adaptation.

Le CEN, de son côté, agit un peu en amont de la dynamique de plantation et de gestion bocagères, mais dans une même perspective. En travaillant sur la variabilité inter- et intra-spécifique des essences bocagères, notamment avec le label Végétal Local[22], la structure cherche à rendre les nouveaux plants plus adaptés et adaptables aux variations du climat, afin qu'ils soient en mesure de fournir les services d'adaptation précédemment cités.

- **Pour des acteurs privés : l'exemple d'un arboriculteur**

L'arboriculteur que nous avons rencontré souhaite planter des haies autour de toutes ses parcelles de vergers, dans un but bien identifié d'adaptation au dérèglement climatique. Les différents épisodes de gelées tardives et de canicules l'ont poussé à intégrer dans son système de nouveaux éléments, dont des haies, pour tenter de lisser ces différents effets. En

[22] Travail de sélection génétique effectué en partenariat avec des pépiniéristes, dans le but de développer des variétés végétales plus robustes aux aléas climatiques.

fonction des vergers (répartis sur trois sites distincts), la visée des plantations ne sera pas exactement la même, tout comme le type de linéaire à implanter. Toutefois, la recherche du rôle de tampon thermique fourni par les haies constitue la motivation principale, qui peut être combinée à d'autres avantages (anti-dérives, production de bois énergie, production de petits fruits et de plantes comestibles).

- **Pour des acteurs publics : l'exemple de la commune de Saint-Maurice-ès-Allier**

Saint-Maurice-ès-Allier est une commune en zone de coteaux soumise à des risques d'érosion, une situation qui a nécessité l'action de la municipalité pour tenter de limiter les dégâts causés par des inondations. Ainsi, la commune s'est lancée, entre autres, dans un programme de plantation des haies, suite à une étude hydraulique préalable. En perspective d'une intensification des épisodes orageux, ont été mis en terre de plus de 500 plants à vocation anti-hydraulique, sur un bassin versant concernant une vingtaine de propriétaires (La Montagne, 2018 ; 2020).

On observe un contraste assez important entre la place des haies dans les documents de planification et dans les stratégies des acteurs. Premièrement, elles ne sont évoquées avec précision que dans la Trame Verte et Bleue et les contrats territoriaux portés par le SMVVA, les autres documents ne décrivant pas de façon précise la manière de gérer le bocage. D'autre part, d'après nos lectures, les haies ne sont **jamais** envisagées comme un élément susceptible de favoriser l'adaptation au dérèglement climatique, ni d'ailleurs l'atténuation de ce dernier (pas de mention de la séquestration carbone, par exemple). Pourtant, force est de constater que cette IAE est déjà appréhendée comme telle, avec des services d'adaptation rendus aux exploitations agricoles tout comme aux populations. Ce constat concerne néanmoins un cercle d'acteurs assez restreint, mais dont la capacité à influencer le débat est réelle (en particulier pour les acteurs techniques qui accompagnent les élu·e·s dans le cadre de plusieurs projets ou dans la gestion de sites bien définis).

La gouvernance d'IAE privées fournissant des bénéfices collectifs, notamment pour l'adaptation : quelle mise en pratique ?

Les infrastructures agroécologiques, ici en l'occurrence les haies, fournissent des services écosystémiques, et notamment d'adaptation, dont les bénéfices profitent à une diversité d'acteurs (d'où l'implication des pouvoirs publics pour agir sur le sujet). Toutefois, les recherches et entretiens effectués ont pointé la difficulté d'agir sur ces IAE pour garantir les services qu'elles rendent, car elles sont majoritairement implantés sur du domaine privé :

> « Deux réalités se côtoient avec d'un côté le bocage en tant que patrimoine commun assurant des services écosystémiques à bénéfices collectifs dans les territoires (pour l'eau, la biodiversité, les paysages, le stockage de carbone…) et de l'autre, le bocage en tant que bien privé et outil de production contribuant à l'économie de l'exploitation par une valorisation économique directe ou indirecte (services écosystémiques agri-environnementaux). Dès lors, la vocation de l'action publique n'est pas de choisir ou privilégier l'une de ces deux réalités, mais de les articuler. » (Sanson, Baudry & Narcy, 2019, p. 12).

Les mêmes auteurs indiquent plusieurs voies possibles pour favoriser cette gouvernance :

- « *La structuration de filières économiques pour assurer la gestion durable des haies et leur renouvellement, en articulant responsabilités individuelles et co-responsabilités ;*
- *La mise en réseau des acteurs et le renforcement de la coopération entre ces acteurs pour l'animation de projets de territoires, notamment autour de la plantation de haies ;*
- *La construction d'outils contractuels en soutien aux agriculteurs et d'outils d'accompagnement et de connaissance pour les gestionnaires de haies et les agriculteurs (plan de gestion durable des haies, pôle métier bocage…).* » (Sanson, Baudry & Narcy, 2019, p. 13) .

Si l'on soumet ces différentes pistes au contexte de Mond'Arverne Communauté, plusieurs démarches nous montrent qu'une diversité d'acteurs du territoire s'engage déjà dans ce sens.

Concernant le premier point, nous évoquerons le travail effectué par la Mission Haies Auvergne et la Chambre d'Agriculture du Puy-de-Dôme pour initier une filière de bois bocager, qui commence, avant toute chose, par convaincre les gestionnaires des intérêts de ce type de ressource[23]. Pour ce qui relève de la mise en réseau des acteurs autour de la question bocagère, celle-ci pourrait théoriquement s'effectuer autour des documents programmatiques intercommunaux structurants. Cependant, d'après notre étude, elle semble pour l'instant se focaliser autour de zones géographiques bien déterminées, comme sur la commune de Saint-Maurice-ès-Allier ou sur des bassins versants, là où les enjeux (qualité de l'eau, érosion) sont globalement acceptés par tous les acteurs en présence. Sur ces zones, le travail en partenariat a bien lieu, facilité par des structures aux fonctions d'animation et d'appui technique. Enfin, nous constatons effectivement la présence d'outils contractuels, souvent sur des échéances de cinq à dix ans. Si, contrairement à d'autres territoires comme la Normandie ou la Bretagne, les outils ciblés sur la gestion bocagère ne sont pas très développés, d'autres dispositifs comme les PAEC permettent d'aborder ce sujet avec le monde agricole, et de les appuyer dans la gestion de leurs haies.

3.2. Quelles stratégies d'adaptation pour Mond'Arverne ?

Le point 3.1. s'est attaché à présenter une infrastructure agroécologique particulière, à analyser les services écosystémiques d'importance pour ce territoire, le contexte social dans lequel elle s'inscrit, mais aussi la manière dont elle peut être mobilisée dans une perspective d'adaptation. Nous allons maintenant nous projeter à une échelle plus large, intégrant une plus grande diversité d'IAE, et mobiliser pour cela des cadres d'analyse plus spécifiques au mémoire, qui sont développés en deuxième partie.

Nous commencerons par présenter les vulnérabilités liées au dérèglement climatique qui sont identifiées par les acteurs rencontrés, ainsi que les actions d'adaptation envisagées ou en cours. Puis, nous analyserons les préférences d'usage des terres qui ont été exprimées, ainsi que les arbitrages et tensions que ces choix génèrent – ou peuvent générer.

[23] L'INRAE de Theix, qui travaille avec ces deux acteurs, mène des recherches depuis plusieurs années sur les potentiels de la litière plaquette, concernant différents types d'élevage (ovins, bovins).

3.2.1. Les vulnérabilités liées au dérèglement climatique identifiées

Des vulnérabilités d'ordre socio-économique et écologique

Excepté un acteur, la totalité des personnes avec qui nous avons échangé ont la sensation que le territoire est vulnérable face au dérèglement climatique, à l'heure actuelle comme dans les années à venir. La plupart des vulnérabilités citées sont d'ordre socio-économique : des dégâts matériels ainsi que des impacts sanitaires sur les populations.

La question des dégâts matériels concerne le territoire dans sa globalité, mais paraît plus flagrante sur certaines zones. Sur celles de coteaux, les coulées de boue sont un impact à la fois déjà constaté et attendu, du fait de l'intensification des précipitations. De façon plus globale pour le territoire, une hausse des inondations et des vents violents est constatée et anticipée, de part la situation hydrographique (présence de nombreux cours d'eau) et d'exposition du territoire (manque d'éléments brise-vent). Sur le volet sanitaire, les canicules plus fréquentes constituent le risque principal, avec des conséquences directes sur les populations les plus fragiles, dont la proportion devrait augmenter dans les années à venir (vieillissement de la population sur le périmètre intercommunal). Précisons que ces vulnérabilités ont surtout été détaillées par les élus rencontrés, dont les responsabilités les placent en première ligne lors de la gestion de ces évènements.

Par ailleurs, sont aussi relevées des vulnérabilités sur le système écologique, notamment sur les milieux boisés et agricoles. Pour les premiers, l'enjeu principal est l'adaptation des essences locales, dont les risques directs sont le dépérissement d'arbres, ainsi que des échecs récurrents dans les programmes de plantation. Concernant les agroécosystèmes, l'augmentation de l'érosion éolienne, les sécheresses, les canicules et les gelées tardives récurrentes, ont toutes pour effet de diminuer la productivité végétale, même si chaque culture réagit à ces effets de manière différenciée.

Les infrastructures agroécologiques et services écosystémiques affectés

Ces vulnérabilités donnent des indications sur plusieurs services écosystémiques d'importance pour le territoire, qui ont tout intérêt à être pérennisés. La figure 16 ci-dessous

recense ces deux types d'éléments : en bas, les différentes vulnérabilités citées par les acteurs (en plus ou moins gros caractères selon leur récurrence), et en haut les services écosystémiques qui y sont associés.

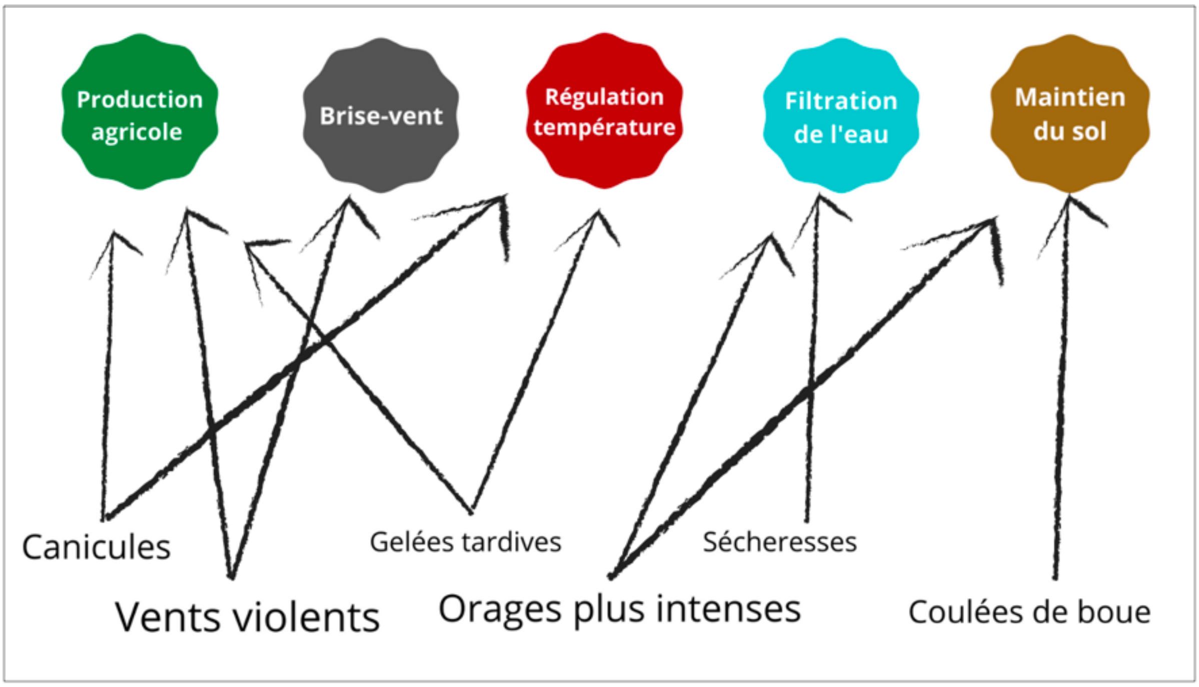

Figure 16. Les services écosystémiques les plus impactés par le dérèglement climatique sur le territoire de Mond'Arverne, d'après une série d'entretiens.

Nous remarquons que la plupart de ces services peuvent être fournis par les haies. Toutefois, d'autres infrastructures agroécologiques sont aussi concernées et ont été citées lors des entretiens, tout comme d'autres éléments du système écologique au sens large. Dans les deux cas, ces infrastructures sont citées pour leur rôle de « tampon » vis-à-vis des effets du dérèglement climatique : bandes enherbées, bosquets, zones humides, forêts. Les prairies et vergers ont également été évoqués à plusieurs reprises pour la production fourragère et alimentaire qu'ils fournissent, et qui doivent être pérennisées. Dans cette perspective, les actions d'adaptation doivent avoir pour but de garantir certains de ces services, voire à garantir simultanément le maintien de plusieurs services.

3.2.2. Analyse des actions d'adaptation

Typologie des actions engagées et en réflexion

La carte ci-dessous recense les différentes actions d'adaptation qui sont réfléchies ou ont été mises en place à l'échelle communale ou infra-communale au sein de la Communauté de Communes. D'autres actions ont une portée plus large : c'est le cas du système de veille à destination des personnes fragiles en période de canicule, ou bien du travail de sélection de variétés végétales locales mené par le CEN, en partenariat avec des pépiniéristes.

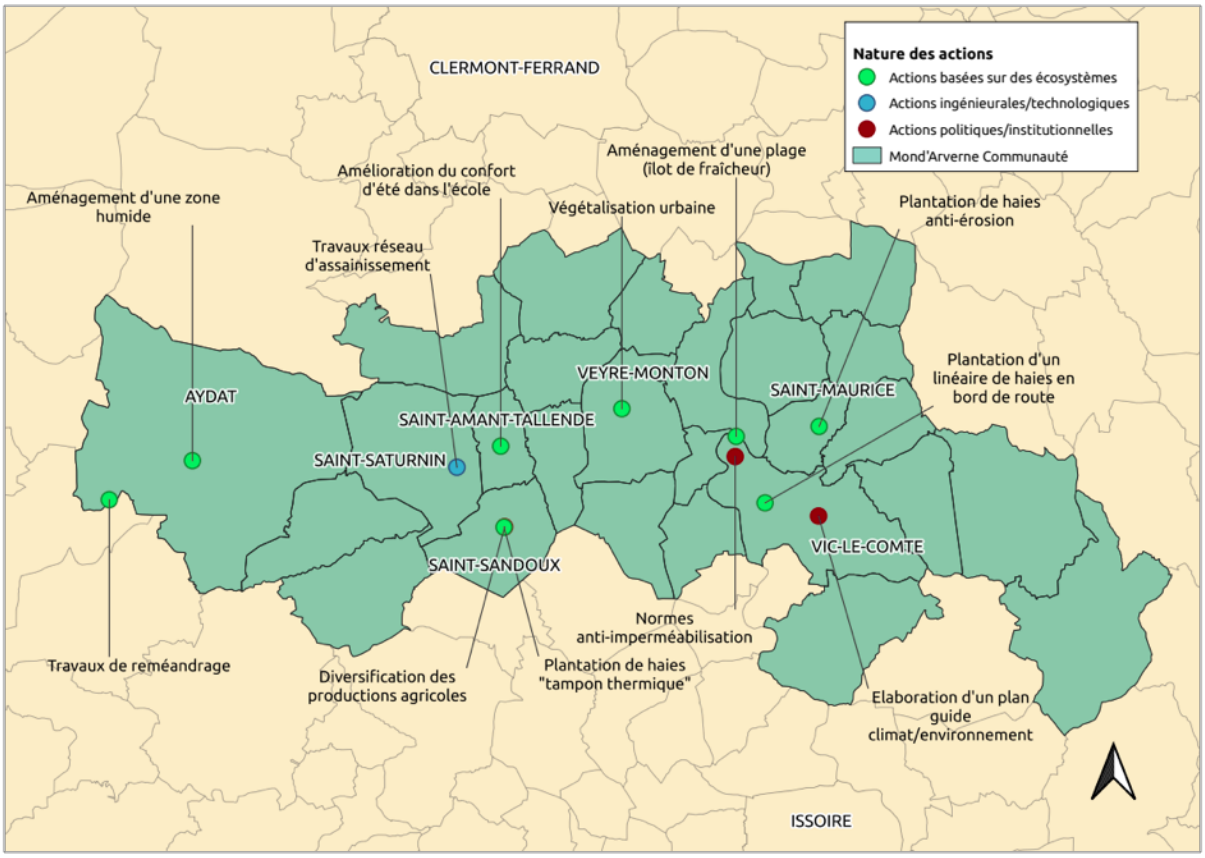

Figure 17. Cartographie des d'adaptation recensées sur le territoire de Mond'Arverne.

L'adaptation : un concept peu approprié par l'échelon intercommunal

La première remarque qui peut être faite est que la plupart des actions citées sont mises en œuvre à une échelle très locale, et répondent davantage à un besoin urgent d'agir qu'à une réelle stratégie planifiée en amont. De fait, assez peu d'actions d'adaptation sont

portées à l'échelle intercommunale, et elles ne font d'ailleurs pas explicitement référence à une nécessité de *s'adapter*. Il s'agit des normes d'urbanisme favorisant l'infiltration de l'eau dans les zones urbaines, en particulier pour les nouveaux lotissements, afin d'éviter les inondations. C'est donc à travers l'outil du PLUi, dont l'élaboration est du ressort de l'intercommunalité du fait de sa compétence urbanisme, que des normes favorisant l'adaptation peuvent être observées en pratique. Notons que le PCAET, document programmatique pourtant axé sur le dérèglement climatique, ne comprend pas de réel volet adaptation déclinant un plan d'actions opérationnel, à la manière du volet atténuation que nous avons pu citer à plusieurs reprises.

Pour le moment, les actions menées se déroulent donc surtout à l'échelle locale, particulièrement au sein des communes selon les aléas spécifiques qu'elles rencontrent. La plupart des initiatives font directement écho à des compétences réglementaires dont elles ont la charge : l'action sociale pour la mise en place d'un système de veille à destination des personnes vulnérables en temps de canicule, ou encore l'entretien de la voirie pour la gestion d'écoulements. De plus, certaines actions ne sont pas menées à l'échelle d'un périmètre institutionnel, mais plutôt d'une ou de plusieurs entités écologiques. C'est le cas de travaux de reméandrage menés par le SMVVA sur la Veyre en amont des habitations, ou bien des recherches sur la variabilité des essences locales auxquelles participe le CEN Auvergne.

À l'échelle communale : gestion de crise plutôt qu'adaptation

Du fait de notre échantillon, nous avons beaucoup plus de retours sur les actions menées par des acteurs publics (échelle communale) que privés. Concernant l'implication des différents acteurs dans ces actions, un schéma assez récurrent se dégage : l'équipe municipale conçoit et pilote l'action ou le plan d'actions, en étant quasi systématiquement appuyée par des partenaires techniques publics ou privés. La réalisation des actions est ensuite réalisée par des prestataires privés selon le code des marchés publics, sauf quand la mise en œuvre possède un caractère pédagogique (e.g. la plantation de haies effectuée par des habitant·e·s). Ce schéma varie quand l'action a lieu sur une partie importante de foncier privé, et qu'elle nécessite donc l'aval, voire l'implication des propriétaires. Cependant, très peu d'actions allant dans ce sens ont été citées.

Bien que les actions relevées permettent de s'adapter aux effets du dérèglement climatique, elles sont rarement énoncées explicitement comme telles. Elles s'apparentent en effet davantage à une forme de gestion de crise, face à laquelle la commune se retrouve en situation de responsabilité directe et se doit de réagir (e.g. dégâts matériels causés par des coulées de boue, pressions de commerces pour diminuer les inondations). Ainsi, la plupart des actions menées ont une échéance plutôt courte, qui laisse juste le temps aux responsables politiques de mettre en œuvre la ou les solution(s) répondant au problème identifié. Par ailleurs, la majorité des actions citées génèrent des co-bénéfices, c'est-à-dire qu'elles ne répondent pas exclusivement à l'enjeu d'adaptation au dérèglement climatique, mais qu'elles fournissent d'autres avantages aux populations. C'est le cas par exemple de l'aménagement d'une aire de baignade en bord d'Allier, qui constitue un accès à la fraîcheur en période de canicule, mais offre aussi un espace de loisirs pour les habitant·e·s ainsi qu'un attrait touristique. Cette démarche de co-bénéfices est compréhensible pour des structures ayant des moyens financiers assez limités, et qui ont tout intérêt à effectuer en priorité des actions pluri-thématiques.

Des acteurs non-institutionnels en demande d'actions d'adaptation

Parallèlement à cela, des communes peuvent aussi mettre en place plusieurs types d'action pour répondre à une problématique précise. C'est le cas de l'amélioration du confort d'été dans les écoles, qui est traitée à Saint-Amant-Tallende conjointement par le prisme de la rénovation thermique des bâtiments, de la sensibilisation des jeunes, et de la végétalisation des cours d'école. Pour autant, cette démarche plus globale est encore au stade de réflexion, et n'a pas été citée dans d'autres communes, bien que ces dernières s'emparent progressivement du sujet depuis quelques années. Notons que dans ce cas, d'autres acteurs sont à l'origine de la réflexion en parallèle des élu·e·s. En effet, les associations de parents d'élèves et l'Adhume sont des parties prenantes importantes pour initier ces discussions.

Des engagements variables selon la taille de la commune

La prise en compte des enjeux d'adaptation paraît aussi extrêmement liée à la taille de la structure à laquelle on s'intéresse, celle-ci déterminant les ressources financières et humaines pour agir sur ce sujet. Les communes de plus grande taille vont par exemple jusqu'à mener des réflexions stratégiques sur leur propre périmètre, en se projetant sur des

plans pluriannuels, tandis que les plus petites sont davantage dans une démarche de gestion de l'urgence (e.g. travaux sur la voirie).

Le monde agricole et les acteurs techniques : d'ores et déjà dans une dynamique d'adaptation

Les seuls témoignages que nous avons obtenu concernant des actions d'adaptation dans le secteur privé concernent le monde agricole. Dans ces situations (diversification des cultures, introduction d'IAE dans le système de production), l'action est menée par l'exploitant·e de sa conception à sa réalisation, avec parfois l'appui technique d'acteurs comme la Chambre d'Agriculture via les services spécialisés. La nécessité de s'adapter est explicite puisque la viabilité économique de l'activité en dépend.

Concernant les acteurs techniques, ceux que nous avons pu rencontrer sont naturellement bien au fait des enjeux d'adaptation - qu'ils soient en contact des milieux agricoles ou naturels. De par leur expertise sur le sujet, ils peuvent être un élément déclencheur d'une mise en action du pouvoir politique : soit en interpellant directement les collectivités et en proposant leur appui, soit en orientant de manière implicite la mise en œuvre des actions dans une perspective d'adaptation.

Pour l'instant, les actions d'adaptation en cours ne laissent ainsi pas entrevoir de tendance générale concernant une typologie, une logique d'acteurs, ou bien une temporalité particulière. Elles sont menées au gré des enjeux locaux, souvent par les communes via leur champ de compétences réglementaires, et ne font pas encore l'objet d'une politique concertée à l'échelle intercommunale. Cependant, il est à noter que la plupart des initiatives menées génèrent des co-bénéfices, pour des raisons économiques mais aussi organisationnelles, ce qui peut justifier pour ce territoire la pertinence d'actions basées sur les écosystèmes, ou actions « vertes ».

Les actions d'adaptation basées sur les écosystèmes

En effet, les adaptations basées sur les écosystèmes sont décrites « *comme des options ayant des co-bénéfices pour l'adaptation des sociétés au changement climatique et l'atténuation de ses effets, le développement socio-économique durable des communautés*

locales, la protection de l'environnement et la conservation de la biodiversité » (Bruley, 2021, p. 15 ; Munang et *al*. 2013). Dans le cas des actions que nous avons recensées, les principaux co-bénéfices sont la hausse de la qualité de vie par l'intégration d'infrastructures semi-naturelles près des habitations et autres lieux de vie, ainsi qu'un impact positif sur la biodiversité. Dans certains cas, ces solutions d'adaptation peuvent même faire partie d'une stratégie d'attractivité du territoire par sa mise en valeur, et donc générer des bénéfices d'ordre économique.

Bien que les co-bénéfices générés par ce type d'adaptation soient souvent assez bien identifiés, force est de constater que leur mise en place n'est pas toujours évidente. En effet, ces actions nécessitent un engagement à long terme, et des ressources humaines pour assumer l'entretien des IAE implantées ou restaurées. En comparaison avec la mise en place d'une infrastructure physique, les IAE peuvent nécessiter un suivi plus régulier. Or, sur certaines communes, l'entretien des haies communales est parfois assumé par des agriculteurs, car la municipalité n'a ni les agents, ni le matériel, pour effectuer ces tâches. L'implantation d'IAE supplémentaire apparaît alors dans ce cas peu réaliste. Ainsi, dans les situations où les actions ne sont pas encore mises en place, la problématique de l'entretien demeure le principal frein.

Notons que ce type d'adaptation commence à prendre une portée opérationnelle, sur des territoires urbains comme ruraux. Le projet LIFE ARTISAN, financé par l'Union Européenne et piloté par l'Office Français pour la Biodiversité, a par exemple vocation à mettre en lumière les solutions d'adaptation basées sur la nature, et à accompagner les collectivités dans une meilleure intégration de ces dernières dans leur projet de territoire. Nous avons suivi un webinaire rendant compte de l'avancée de ce projet, qui proposait un focus sur les stratégies d'adaptation autour de la gestion quantitative de l'eau. Les exemples présentés ont fait écho avec ce que nous avons pu observer à l'échelle de notre territoire d'étude, même si, dans notre cas, les démarches étaient plus embryonnaires. On observe un mélange de plusieurs actions : certaines portées par le monde agricole sur l'intégration d'IAE dans leur système de production, des réflexions menées par les élu·e·s autour de l'urbanisme permettant d'intégrer par exemples des « zones à inonder », des aménagements portés par des syndicats de gestion de l'eau en amont des bassins versants, ainsi que diverses initiatives autour de la végétalisation en milieu urbain. L'intérêt de la mise en valeur de ces démarches est de montrer la plus-value de ces différentes options par rapport à d'autres actions plus

“traditionnelles”, basées sur des infrastructures physiques et technologiques, ainsi que de proposer des solutions très concrètes aux freins qui peuvent être identifiés.

Nous faisons ici l’hypothèse que les effets du dérèglement climatique demeurant encore relativement « maîtrisables » au sein de territoires comme Mond’Arverne, les actions basées sur les écosystèmes peuvent constituer des options attractives, notamment pour viser un traitement intégré des enjeux. Dans notre cas, les effets de l’érosion de la biodiversité sont actuellement aussi, voire plus importants, que ceux du dérèglement climatique, et ont donc tout intérêt à être traités de concert. En revanche, cela n’est peut-être pas si évident pour d’autres territoires, ou des solutions plus technologiques peuvent sembler plus efficaces lorsque l’on se trouve « au pied du mur » (e.g. construction de digues pour des territoires littoraux).

3.2.3. Quelles trajectoires d’usage des terres pour favoriser l’adaptation ?

Après avoir étudié les différentes actions d’adaptation mises en œuvre ou en réflexion, nous avons souhaité sonder les préférences des acteurs concernant plusieurs scénarios d’usage des terres. Dans chacun d’eux, les infrastructures agroécologiques et naturelles au sens large ont des places variables, et certains types d’actions d’adaptation plutôt que d’autres vont être favorisés. Dans cette perspective, la prise en compte des tensions et conflits liés à l’usage des terres est importante pour deux raisons : avoir une idée des rapports de force qui sous-tendent chaque trajectoire, et permettre au sein de ces dernières une utilisation des IAE qui soit en mesure de satisfaire les différentes attentes en matière de SE.

Les scénarios d’usage des terres plébiscités par les acteurs

Le scénario le plus mis en avant est celui de l’attractivité touristique (voir en détail le scénario au point 2.2.1.), notamment par la valorisation de sites phares du territoire : lac d’Aydat, sites archéologiques, Val d’Allier, Comté d’Auvergne. La préférence pour ce scénario s’explique pour diverses raisons. La première est que cette trajectoire figure explicitement dans les différents documents programmatiques (en particulier du projet de territoire) comme une orientation majeure de l’intercommunalité dans les prochaines années. Pour les élu·e·s, cette direction constitue une opportunité de créer de la valeur économique en

valorisant les ressources locales, et donc d'obtenir des ressources financières pour investir dans d'autres trajectoires parallèles. D'autres acteurs voient ce scénario en appui d'un autre, comme la Chambre d'Agriculture qui perçoit une opportunité de soutenir l'activité agricole locale via l'agritourisme. La relocalisation économique a d'ailleurs été citée par la plupart des élus comme une condition nécessaire à la mise en œuvre de projets sur le territoire, dans un contexte de dotations aux collectivités actuellement insuffisantes. En revanche, des points de vigilance ont régulièrement été émis de la part d'autres acteurs, qui voient dans la relocalisation économique des risques d'artificialisation des sols.

Les deux autres scénarios les plus mis en avant sont ceux de la transition alimentaire et de la transition énergétique. Le premier s'explique sans doute de par le type d'acteurs rencontrés, naturellement proches du monde agricole. De plus, Mond'Arverne Communauté est engagée dans un Projet Alimentaire Territorial, et a participé à plusieurs phases de diagnostic et de réflexions sur le système alimentaire, et aux manières de le relocaliser. Ainsi, nous pouvons dire que ce sujet infuse depuis plusieurs années sur le territoire, bien que l'intercommunalité ne s'en soit pas encore réellement saisi, du fait d'autres démarches engagées en parallèle (phases d'élaboration du PCAET, puis du PLUi). Cela n'est pas le cas pour la transition énergétique. En effet, l'un des axes principaux du PCAET de Mond'Arverne concerne la production d'énergie renouvelable. Des projets sont d'ores et déjà en cours, en particulier concernant l'implantation de panneaux photovoltaïques sur des toitures privées et publiques, et constituent une mise en place concrète du plan d'actions. À l'image du tourisme, ce scénario est donc déjà en cours de réalisation, d'où sa mise en avant.

Enfin, la naturalisation du territoire constitue l'une des deux trajectoires les moins souhaitées, et quand elle l'est, c'est que l'acteur n'a pas exprimé de préférences vis-à-vis des cinq scénarios proposés. Au vu des enjeux du territoire tels qu'ils sont appréhendés par les acteurs, la naturalisation ne paraît pas prioritaire, ou bien sur des zones relativement délimitées, et avec un but bien identifié (e.g. vocation touristique).

La difficulté à arbitrer au sein d'un territoire multifonctionnel

La priorisation de ces scénarios n'a pas été évidente à effectuer pour les personnes interrogées. Nous avons conscience que cet exercice n'est pas facile, tant il va de soi que certaines actions, ou politiques, peuvent être transversales et répondre simultanément à

plusieurs scénarios. Néanmoins, il a aussi permis de mettre en avant la difficulté à effectuer des arbitrages au sein d'un territoire résolument multifonctionnel, et qui souhaite continuer à s'assumer comme tel. Cette problématique nous a d'ailleurs été confirmée par un chargé de mission de l'intercommunalité, en référence à l'élaboration de l'un des documents programmatiques :

> « Dans l'absolu je suis d'accord, il faut tout faire. Mais le problème avec le fait de tout faire, c'est quels sont les moyens qu'on peut mettre derrière ça ? Parce qu'il faut des moyens. Alors il faut de l'ambition, faut de l'envie, très bien, mais ça ne suffit pas. Il faut du temps humain, de l'argent, de l'ingénierie, des capacités à travailler ensemble [...]. Est-ce qu'il y a pas un intérêt à mettre le paquet sur une, deux ou trois priorités ? Et c'est là où à chaque fois c'est compliqué. On voit le projet de territoire : on couvre tout. Mais on est dans l'incapacité de tout faire […]. Parce que la réalité financière fait qu'une collectivité aujourd'hui elle a de moins en moins de ressources, elle doit gérer ce qu'elle a fait jusqu'à présent, et elle a peu de marge de manœuvre pour de nouvelles choses. » (Agent de Mond'Arverne Communauté, 2021).

Vis-à-vis de ces difficultés d'arbitrage, l'un des partis pris que nous avons pu relever est de se concentrer en premier lieu sur les scénarios de relocalisation économique et d'attractivité touristique, afin que les ressources qu'ils génèrent constituent un tremplin à la réalisation des autres scénarios. Mais ce n'est pas la seule manière de considérer le problème. D'autres acteurs semblent exprimer que l'action face aux changements globaux (qui peut être représentée par les scénarios 1, 4 et 5) est prioritaire, et qu'elle peut constituer, de surcroît, des opportunités économiques intéressantes pour le territoire.

Des actions qui dessinent des usages des terres souhaités

Les actions prioritaires à mettre en œuvre pour réaliser les scénarios donnent des éléments sur la manière jugée pertinente d'utiliser les terres. Bien que des initiatives très diversifiées aient été relevées, il nous semble que quatre tendances se dégagent, dont deux font directement écho aux scénarios les plus plébiscités.

La première est l'aménagement de zones touristiques, comme en bord d'Allier ou autour du lac d'Aydat (amélioration de l'offre d'hébergement et de stationnement, proposition d'activités). Dans un deuxième temps, nous relevons plusieurs projets en lien avec le scénario de transition énergétique : l'implantation de sources de production d'énergie renouvelable sur le domaine public, ou encore le montage d'une coopérative citoyenne de production

d'électricité. Ensuite, un autre type d'actions consiste à sanctuariser des zones agricoles pour garantir le maintien de cette activité sur le périmètre intercommunal, notamment au regard de plusieurs enjeux : ne pas artificialiser des sols ayant un potentiel agronomique avéré (via les documents d'urbanisme), aménager des espaces-test pour les porteurs et porteuses de projet agricole, et maintenir les activités existantes. Enfin, plusieurs acteurs jugent qu'agir sur la mobilité (réduction des déplacements, multimodalité, modes doux…) permet d'avoir une influence de façon transversale sur plusieurs sujets. En termes d'aménagements, cela peut se traduire par la création de voies cyclables et de pôles multimodaux pour desservir de façon homogène le territoire, sachant que les principales voies de transport nécessaires à la circulation des transports en commun sont déjà existantes (voie ferrée, routes départementales, autoroute).

Il est intéressant de constater que la typologie de ces actions est assez différente de celle qui a été observée en matière d'adaptation. Tout d'abord, il s'agit ici quasi exclusivement d'actions dites « grises » ou « soft ». Les premières sont des aménagements de voirie, des agrandissements de zones d'activités, l'implantation de technologies, tandis que les secondes visent en règle générale à modifier des normes existantes afin d'y intégrer de nouveaux enjeux (e.g. concilier production énergétique et enjeux paysagers dans le PLUi, mieux protéger le foncier agricole). Encore au sein de ces actions dites « soft », on observe des phénomènes de réorganisation des acteurs, toujours à l'aune de ces nouveaux enjeux (e.g. fusion de l'office de tourisme intercommunal avec celui de la métropole de Clermont Auvergne, afin de mener une politique commune de plus grande envergure). Si les actions d'adaptation étaient en majorité réalisées à l'échelle communale ou d'une entité écologique, les actions en lien avec l'usage des terres sont ici conçues et réalisées par l'intercommunalité, bien que la mise en œuvre puisse associer d'autres acteurs et d'autres échelons de gouvernance.

3.2.4. Appréhender les tensions et conflits liés à l'usage des terres : une prise en compte nécessaire

Typologie des tensions et conflits

Les entretiens effectués nous ont permis de mettre en lumière plusieurs conflits et tensions existant sur le territoire au sujet de l'usage de l'espace.

Tout d'abord, commençons par dire que peu de conflits ont été énoncés : nous n'en avons noté que deux. Le premier est un phénomène de dénonciation par courrier d'agriculteurs ayant arraché des haies sur leurs parcelles. Ces dénonciations sont faites par des habitant·e·s, qui contactent la mairie pour leur faire part de leur désaccord, bien que souvent, la commune possède une marge de manœuvre limitée sur le sujet. Les conflits autour de cette infrastructure agroécologique ne sont pas rares. Torre et *al*. (2006) soulignent d'ailleurs leur prédominance au sein des espaces périurbains :

> « [Le réseau de haies est devenu] une gêne plus qu'un auxiliaire agricole, en particulier dans les zones cultivées en céréales et en maïs. Privées d'intérêt technique, elles sont en revanche fortement chargées de dimension affective de la part des résidents non agriculteurs et d'une partie des exploitants attachés à cet élément symbolique du territoire. Le programme Agenda 21, en cours, renforce ce processus de "patrimonialisation", et révèle des conflits quant à la préservation ou à la restauration du réseau mis en place dans le passé par les exploitants agricoles. » (Torre et *al*., 2006, pp. 421-422).

Le deuxième conflit recensé est la contestation de l'implantation d'un supermarché sur des terres agricoles, une prise de position portée par des habitant·e·s ainsi que des associations. Ce faible nombre de conflits relevés est bien sûr loin d'être représentatif de la réalité : il est expliqué par la faible taille de notre échantillon, et par le caractère sensible de ce sujet pour certains acteurs, qui n'ont parfois pas intérêt à s'y étendre. Cependant, des recherches bibliographiques portant sur l'intercommunalité n'ont pas abouti à une découverte massive de conflits supplémentaires, ce qui laisse présager que l'étude des tensions n'est pas inintéressante pour déceler des conflits latents.

La figure 18 présente les différentes tensions liées à l'usage des terres qui ont été rapportées. Elle y résume les principales composantes sociales (objet, acteurs) et écologiques (SNI, SE) qui entourent chacune d'entre elles.

**Descriptif**	**Objet de la tension**	**Acteurs en présence**	**SNI concernées**	**SE principaux**
Implantation d'éoliennes	Maîtrise du foncier	Élu·e·s, particuliers, entreprises de prospection	IAE des agroécosystèmes (prairies)	Esthétisme, production agricole
Usage des cônes de vue du territoire	Maîtrise du foncier	Élu·e·s, habitant·e·s, bureaux d'étude paysage	Forêts	Esthétisme, stockage carbone, production de bois
Poids politique des petites communes dans les instances de gouvernance relatives à l'usage des terres (PLUi)	Maîtrise du foncier	Élu·e·s	/	/
Implantation de photovoltaïque au sol *vs* enjeux environnementaux et alimentaires	Maîtrise du foncier	Élu·e·s, entreprises de prospection, habitant·e·s, Architectes des Bâtiments de France	IAE des agroécosystèmes	Production agricole, conservation de la biodiversité, esthétisme
Normes de densification du bâti *vs* problèmes d'écoulement des eaux	Maîtrise du foncier	Élu·e·s, habitant·e·s, Direction Départementale des Territoires	Jardins, bandes enherbées, haies	Filtration de l'eau
Insertion de végétation ligneuse sur les	Maîtrise du foncier	Agriculteur·rice·s, élu·e·s	IAE des agroécosystèmes	Maintien du sol

zones agricoles sensibles aux écoulements				
Tensions inter-agriculteurs sur la dispersion de produits phytosanitaires	Externalités négatives	Agriculteur·rice·s	SNI des agroécosystèmes	Production agricole, filtration de l'air, filtration de l'eau
Aménagement des chemins ruraux : concilier passage des engins agricoles et activités récréatives	Activités de loisirs	Agriculteur·rice·s, élu·e·s, habitant·e·s	Haies	Esthétisme, conservation de la biodiversité
Conciliation entre activités agricoles et récréatives	Activités de loisirs, externalités négatives	Agriculteur·rice·s, élu·e·s, habitant·e·s	IAE des agroécosystèmes (haies)	Esthétisme, production agricole, anti-dérives des produits phytosanitaires
Arbitrages défavorables à l'activité agricole : manque de foncier constructible pour des bâtiments agricoles	Maîtrise du foncier	Agriculteur·rice·s, élu·e·s		Production alimentaire

Figure 18. Les principales tensions liées à l'usage des terres à l'échelle d'une intercommunalité, d'après une série d'entretiens.

La maîtrise du foncier : principal objet de tensions

L'objet de tension le plus répandu est de loin la maîtrise du foncier, c'est-à-dire des clivages entre plusieurs possibilités d'utiliser des terres. Cela s'explique, selon nous, pour deux raisons. La première est le caractère multifonctionnel du territoire, qui accueille des attentes très différentes en matière d'utilisation de l'espace, et qui se retrouvent en particulier au sein d'espaces interstitiels tels que le périurbain. La deuxième est peut-être davantage liée à la gouvernance de la maîtrise du foncier, au sein de laquelle les acteurs et institutions représentantes ont des positions très variables. Notons par exemple le sentiment relevé par les petites communes de peser assez peu dans la balance des décisions à l'échelle intercommunale, et donc d'avoir une capacité d'influence limitée sur les outils déployés à cette échelle. Si ce constat a lieu au sein même de la sphère politique, de tels sentiments peuvent aussi être observés entre les élu·e·s et la société civile. Par exemple, ont été contestées par des agriculteurs plusieurs décisions d'urbanisme à l'échelle communale, qui, selon eux, sont défavorables à l'usage agricole (en l'occurrence le manque de terrains agricoles ou autorisant la construction de bâtiments agricoles).

Si le territoire fait l'objet d'attentes diversifiées en matière de maîtrise foncière, cela s'explique en premier lieu par ses caractéristiques socio-économiques. En effet, la faible présence d'activités industrielles sur le périmètre intercommunal exclut de fait les conflits liés à des externalités négatives qui peuvent y être associés. En revanche, des tensions de ce type ont été relevées vis-à-vis du monde agricole concernant l'utilisation de produits phytosanitaires. Si ces plaintes de la part des riverains peuvent être récurrentes, elles sont aussi relativement circonscrites à des zones très périurbaines, où se côtoient activité céréalière conventionnelle et urbanisation croissante.

L'énergie : une question encore sensible

Plusieurs tensions sont relatives à l'implantation d'infrastructures de production d'énergie. La principale critique émise est la perte de services écosystémiques rendus par les infrastructures agroécologiques, principalement de l'ordre de la production alimentaire ou de la conservation de la biodiversité, au profit de la production énergétique locale. Si cela peut paraître paradoxal au regard de l'attrait pour ce scénario, précisons toutefois que ces tensions s'articulent autour d'installations énergétiques particulières (éolien, photovoltaïque au sol,

méthanisation). À l'image d'autres territoires (Torre et *al.*, 2016, p. 46), ce sont surtout au sujet d'infrastructures qui modifient de façon conséquente l'usage des sols que la résistance est la plus forte, ce qui laisse peut-être entrevoir un déploiement important d'autres modes de production peu consommatrices de foncier.

Activités récréatives et agricoles : vers des tentatives de conciliation

L'esthétisme paysager semble s'imposer ici comme une réelle exigence, à l'image de la démarche de valorisation touristique qui est en cours, et l'attrait pour le scénario associé. Cela peut être illustré par les tensions liées à l'utilisation de certains espaces comme les chemins ruraux, pourtant initialement alloués à l'activité agricole. Ces espaces semblent évoluer aujourd'hui vers un usage hybride, même si cela n'est pas nécessairement officialisé. La gestion des infrastructures agroécologiques se fait alors en conséquence, comme cela peut être observé pour les haies. Ces dernières doivent être assez hautes pour fournir leur rôle d'esthétisme auprès des promeneurs et promeneuses, mais suffisamment taillées pour permettre le passage des différents engins agricoles. Toutefois, concilier agriculture et usages récréatifs ne constitue pas l'unique élément déterminant derrière ce type de tension. En particulier parce qu'au sein même du scénario de transition alimentaire, plusieurs trajectoires semblent entrer en concurrence. Le rejet de certaines pratiques agricoles actuelles, mais aussi le manque d'espace alloué pour cette activité, impose un tri dans les projets présents. À quel point le monde agricole est-il associé à ces objectifs d'autonomie alimentaire ? Cette dernière n'en est-elle pas encore à un stade embryonnaire ?

L'adaptation : nouveau vecteur de tensions ?

Deux situations nous semblent être en lien avec notre problématique d'adaptation. La première est la difficulté rencontrée par une commune de convaincre des agriculteurs de faire évoluer leurs pratiques culturales sur des versants sensibles, afin de limiter les risques de coulées de boue. La deuxième concerne les normes d'urbanisme favorisant la densité du bâti, jugées peu appropriées par certaines communes. Si l'objectif de densifier les milieux urbains paraît souhaitable pour limiter l'artificialisation des sols, il peut parfois constituer un frein à la filtration de l'eau en cas de fortes précipitations, qui était autrefois facilitée par la végétation présente.

Cela nous amène déjà deux constats préliminaires. La nécessité de favoriser une intégration très poussée du milieu agricole, et au sens large du secteur privé, dans les démarches favorisant l'adaptation, qui ne l'est vraisemblablement pas aujourd'hui. En outre, se pose la question de la capacité à faire évoluer un certain nombre de normes à l'aune d'enjeux nouveaux, notamment en fonction des particularités locales.

Conclusion

La conservation de plusieurs infrastructures agroécologiques (IAE) fournissant des services écosystémiques d'intérêt collectif pose question, notamment au regard des changements globaux et des attentes diversifiées en matière d'usage des sols. Notre travail avait vocation à livrer des éléments de réponse à la question suivante : *Quelle place peuvent prendre les infrastructures agro-écologiques, en particulier les haies, dans les stratégies d'adaptation des territoires périurbains, notamment au dérèglement climatique ?*

Nous avons commencé par présenter le projet de recherche dans lequel s'inscrit ce travail, ainsi que les cadres d'analyse qui y sont mobilisés : système socio-écologique, analyse institutionnelle et de développement, et théorie de la viabilité. À cela s'ajoutent d'autres apports théoriques, comme plusieurs travaux sur l'adaptation au dérèglement climatique, la prospective territoriale, et les conflits d'usage de l'espace. Par la suite, nous avons présenté les méthodes d'enquête qui ont été utilisées lors du stage et de la rédaction du mémoire, et qui s'appliquent à deux échelles de réflexion : celle d'une infrastructure agroécologique, la haie, et celle d'un territoire institutionnel et de ses choix d'usage des terres. Il nous semble que ces deux points de vue nous ont permis de fournir des éléments de réponse complémentaires pour répondre à la problématique posée.

Les infrastructures agroécologiques pour l'adaptation : des usages existants mais qui peinent à s'institutionnaliser

Nous avons pu constater qu'au sein d'un territoire périurbain comme Mond'Arverne Communauté, la haie est en mesure de fournir plusieurs services d'adaptation au dérèglement climatique (maintien du sol, régulation de la température…), qui sont plutôt bien conscientisés par les acteurs en présence. Que ce soit au sein de municipalités ou dans le cadre de stratégies individuelles d'agents économiques, la mobilisation de cette IAE dans une perspective d'adaptation a été relevée. De façon plus large, les actions d'adaptation menées sont d'ailleurs en majorité des actions dites "vertes", basées sur les écosystèmes, même si

l'adaptation est rarement la seule raison qui motive leur mise en place. En effet, ce type d'actions génère souvent des co-bénéfices, d'ordre écologique ou socio-économique, qui constituent un argument pertinent pour des structures aux moyens humains et financiers limités, ayant intérêt à prioriser des actions pluri-thématiques. Cependant, un décalage important est à noter entre la place de ces actions dans les stratégies des acteurs rencontrés et celle qu'elles occupent dans les documents programmatiques du territoire. Dans ces derniers, les IAE, notamment les haies, ne sont jamais abordées dans une perspective d'adaptation. Nous ferons ici la supposition que le contexte social qui entoure cette IAE participe à ce décalage : même si cela tend à évoluer, les différents services écosystémiques rendus par les haies (filtration de l'eau, support à la production agricole, biodiversité…) sont traités par des acteurs ou groupes d'acteurs spécifiques, à l'échelle locale comme nationale. Le manque de vision plus systémique de la haie constitue peut-être un frein à des aménagements bocagers qui fourniraient des services collectifs d'adaptation.

Une approche par l'usage des terres a apporté des pistes de réflexion supplémentaires sur l'intégration de ces IAE à l'échelle du territoire. Dans les projections des acteurs concernant des scénarios d'usage des terres souhaités, sont revenues en priorité des trajectoires relatives au développement touristique par la valorisation de sites emblématiques, ainsi que la transition énergétique par la production d'une énergie locale bas-carbone. Les infrastructures agroécologiques semblaient alors avoir une place relativement limitée, au profit d'actions plus technologiques. Cette priorisation doit être nuancée, puisque dans les orientations des documents programmatiques, toutes ces trajectoires ont vocation à être menées de concert. Néanmoins, les témoignages que nous avons récolté allaient souvent dans le sens d'un ou de plusieurs services écosystémiques d'intérêt (production agricole, esthétisme), reflétant des positions plutôt individuelles concernant des scénarios supposés garantir des services collectifs.

Vers de nouvelles instances autour de la gouvernance des IAE ?

Il nous semble qu'il existe, à l'heure actuelle, peu d'espaces où les attentes d'acteurs territoriaux vis-à-vis des services écosystémiques à pérenniser peuvent être exprimées. En conséquence, la gestion des infrastructures agroécologiques a tendance à se faire selon les préférences d'acteurs privés, au détriment de services plus collectifs, dont l'importance est amenée à croître dans un contexte d'adaptation. Cela ne veut pas dire qu'il n'existe aucune

situation où les choix de gestion sont faits de manière consensuelle et acceptée. Toutefois, ces situations sont davantage le fait de contextes ultra-locaux, autour d'entités écologiques sur lesquelles une action s'avère indispensable (e.g. pollution aux nitrates de cours d'eau). De plus, le nombre d'acteurs impliqués est aussi plus limité qu'à l'échelle intercommunale. Pourtant, il nous semble que cet échelon décisionnel pourrait, dans une certaine mesure, être pertinent pour réfléchir à la maîtrise du foncier, au vu des compétences croissantes dont sont dotées les intercommunalités, mais aussi dans le cas d'un territoire multifonctionnel où la question de l'usage des terres se pose constamment. Cependant, dans l'optique d'intégrer un maximum de préférences, la gouvernance mise en place se doit d'inclure certains acteurs qui sont aujourd'hui absents ou peu présents, que ce soit au sein ou à l'extérieur de la sphère politique (petites communes, monde agricole).

Plus que des résultats quantitatifs sur les services écosystémiques à privilégier sur ce territoire et à la manière de les fournir, il nous semble que ce travail a permis de tester une méthode de recueil des données, mais aussi d'obtenir une vision précise des enjeux de notre IAE d'étude par la rencontre d'acteurs spécialisés du sujet. Si plusieurs informations ont pu être récoltées par le biais des entretiens semi-directifs, ces derniers nous semblent être insuffisants pour réfléchir à comment concilier différents intérêts en matière de SE concernant l'usage des terres, notamment au vu des contraintes méthodologiques que nous avons pu expliciter. La conduite d'ateliers ou d'entretiens collectifs, initialement prévue, nous paraît nécessaire, bien que leur organisation amène de nouveaux questionnements (mobilisation des acteurs pertinents, équilibre entre acteurs spécialistes et non-spécialistes…). Ce type de moments pourrait aussi être l'occasion d'imaginer des usages hybrides des infrastructures agroécologiques, voire plus généralement des infrastructures semi-naturelles : quelle gestion des haies pour concilier attrait touristique et filtration de l'eau, quel usage des prairies pour garantir une productivité fourragère face au dérèglement climatique ainsi qu'un certain niveau de biodiversité, etc.

Bibliographie

ADEME (2021). *L'atténuation et l'adaptation*. Contenu en ligne. URL : https://www.ademe.fr/expertises/changement-climatique/quoi-parle-t/lattenuation-ladaptation.

AFAC AGROFORESTERIE (2015). *Les haies*. 14p.

AGARD, J. & SCHIPPER, L.E (2014). *Glossaire AR5*. 24p.

AIGRAIN, P., DUCHENE, E., DE CORTAZAR ATAURI, I.G., GAUTIER, J.,GIRAUD-HERAUD, E., HANNIN, H. & TOUZARD, J.M. (2016). « Travaux de prospective sur l'adaptation de la viticulture au changement climatique : quelles séries d'évènements pourraient favoriser différentes stratégies d'adaptation ? », *BIO Web of Conferences*, 7, 10p.

ANDERIES, J.M., BARRETEAU, O. & BRADY, U. (2019). « Refining the Robustness of Social-ecological Systems Framework for comparative analysis of coastal systems adaptation to global change », *Regional Environmental Change*, 19 (7), pp. 1891-1908.

ANDERIES, J.M., JANSSEN, M.A. & OSTROM, E. (2004). « A framework to analyze the robustness of social-ecological systems from an institutional perspective », *Ecology and society*, 9 (1), 18p.

ASSEMBLEE DES COMMUNAUTES DE FRANCE (AdCF) (2013). *Représenter l'intercommunalité. Enjeux et pratiques de la communication des communautés*. 87p.

BAUDRY, J. BUNCE, R.G.H. & BUREL, F. (2000). « Hedgerows: An international perspective on their origin, function and management », *Journal of Environmental Management*, 60, pp. 7-22.

BERGES, L., ROCHE, P. & AVON, C. (2010). « Corridors écologiques et conservation de la biodiversité, intérêts et limites pour la mise en place de la Trame verte et bleue », *Sciences Eaux Territoires*, 3, pp. 34-39.

BERTRAND, F., BREDIF, H., DUCHENE, E. JOSIEN, E. & TABEAUD, M. (2013). « Activités agricoles et territoires », in SOUSSANA, J.F., *S'adapter au changement climatique : agriculture, écosystèmes et territoires,*Quæ, 293p.

BERTRAND, F. & FOUQUERAY, T. (2017). « Un Parc Naturel Régional en apprentissage : enseignements d'une démarche d'adaptation aux changements climatiques des actions en faveur de la biodiversité », *Norois*, 245, pp. 47-61.

BERTRAND, F. & RICHARD, E. (2012). « Les initiatives d'adaptation aux changements climatiques. Entre maintien des logiques de développement et renforcement des coopérations entre territoires », *Territoire en mouvement*, 14-15, pp. 138-153.

BERTRAND, F. & RICHARD, E. (2015). « La délicate existence locale de l'adaptation aux changements climatiques : avec, sans, ou à côté de l'atténuation », *Développement durable et territoires*, 6 (3).

BERTRAND, F. & SIMONET, G. (2012). « Les trames vertes urbaines et l'adaptation au changement climatique : perspectives pour l'aménagement du territoire », *VertigO*, Hors-série (12).

BIESBROECK G. R., SWART R. J., & CARTER T. R. (2010). « Europe adapts to climate change. Comparing national adaptation strategies », *Global Environmental Change*, 20, pp. 440-450.

BOISSIER, F. (2020). « Les territoires face à l'adaptation au changement climatique », *Etudes*, pp. 45-56.

BRETAGNOLLE, V. (2013. « Conflits autour des ressources naturelles en territoire rural », *Le développement durable à couvert*, Editions CNRS, 363p.

BRULEY, E. (2021). *Les populations travaillent avec la nature pour co-produire l'adaptation aux changements globaux dans les Alpes françaises*. Thèse de doctorat, Université Grenoble Alpes, 226p.

CARON, A. & TORRE, A. (2005). « Conflits d'usage et de voisinage dans les espaces ruraux », *Proximités et changements socio-économiques dans les mondes ruraux*, Paris, INRA Editions.

CEREMA (2019). *La prospective au service des territoires au regard du changement climatique. Retour d'expérience sur la Communauté d'agglomération « Territoires Vendômois »*. 54p.

CHAMBRE D'AGRICULTURE ALLIER. *Entretenir les haies, qui s'en charge ?* Article en ligne. URL : https://extranet-allier.chambres-agriculture.fr/enregistrements-locaux/juridique-articles/fermage/entretenir-ses-haies-qui-sen-charge/.

CHAMBRE D'AGRICULTURE DES BOUCHES-DU-RHÔNE (2017). *Directive nitrates 2017. Pour une agriculture respectueuse de l'eau*. 24p.

CHAMBRE D'AGRICULTURE DE LOIRE-ATLANTIQUE (2016). *L'agroforesterie au service de performances techniques et environnementales des élevages*. Rapport d'étude, 18p.

CHAMBRE D'AGRICULTURE DU PUY-DE-DÔME (2018). *Les auxiliaires des cultures : les connaître et les favoriser*. 8p.

CHURCH, J.M. (2018). « Trajectoires d'adaptation face au changement climatique : analyse et transformation du système de gouvernance du massif ardennais », *Revue de géographie alpine*, 106 (3).

DESSE, R.P., FRANÇOIS, A., HOLVOET, M. & SAWTSCHUK, J. (2017). « Introduction. Adapter les territoires aux changements climatiques: transition urbanistique et aménagement de l'espace », *Norois*, 4, pp. 7-13.

DURANCE, P., GODET, M., MIRENOWICZ, P. & PACINI, V. (2007). « La prospective territoriale », *Cahier du LIPS*, 7, 134p.

INSEE (2018). Dossier complet. Commune : Manglieu (63205). 9p.

INSEE (2018). Dossier complet. Commune : La Sauvetat (63413). 9p.

INSEE (2018). Dossier complet. Commune : Saint-Saturnin (63396). 9p.

INSEE (2018). Dossier complet. Commune : Veyre-Monton (63455). 9p.

LA MONTAGNE (2018). « Saint-Maurice. Des haies contre les inondations et l'érosion ». URL : https://www.lamontagne.fr/saint-maurice-63270/actualites/des-haies-contre-les-inondations-et-lerosion_13087918/.

LA MONTAGNE (2020). « Saint-Maurice. Solidarité pour la plantation de haies ». URL : https://www.lamontagne.fr/saint-maurice-63270/actualites/solidarite-pour-la-plantation-des-haies_13761277/.

LAMARQUE, P. (2012). *Une approche socio-écologique des services écosystémiques. Cas d'étude des prairies subalpines du Lautaret.* Thèse de doctorat, Université Grenoble Alpes, 283p.

LAVOREL, S., COLLOFF, M., LOCATELLI, B., GORDDARD, R., PROBER, S., GABILLET, M., DEVAUX, C., LAFORGUE, D. & PEYRACHE-GADEAU,V. (2019). « Mustering the power of ecosystems for adaptation to climate change », *Environmental Science & Policy*, 92, pp. 87-97.

LE GUILLOU, F. (2020). *Approche systémique de la haie, pour passer de sa multifonctionnalité à la conception de plans de gestion*. Thèse de doctorat, Université de Normandie, 691p.

LIU, J., DIETZ, T., CARPENTER, S.R. & ALBERTI, M. (2007). « Complexity of coupled human and natural systems », *Science*, 317, 5844, pp. 1513-1516.

LOI N° 2014-58 du 27 janvier 2014 de modernisation de l'action publique territoriale et d'affirmation des métropoles. (J.O. 28 janvier 2014).

LOI N°2015-991 du 7 août 2015 portant nouvelle organisation territoriale de la République. (J.O. 8 août 2015).

MAGNAN, A. (2009). « Proposition d'une trame de recherche pour appréhender la capacité d'adaptation au changement climatique », *VertigO*, 9 (3).

MAGNIN, L. (2021). *La haie requalifiée. Enquête sur un dispositif d'écologisation de la politique agricole commune (2014-2019)*. Thèse de doctorat, Université Paris-Est, 509p.

McGINNIS, M.D. (2011). « An introduction to IAD and the language of the Ostrom workshop: a simple guide to a complex framework », *Policy Studies Journal*, 39 (1), pp. 169-183.

MILLENIUM ECOSYSTEM ASSESSMENT (2005). *Ecosystems and human well-being: Synthesis*. Island Press, Washington DC.

MINACA, L. (2021). *Caractérisation et évaluation qualitative des services écosystémiques au sein de la communauté de communes Mond'Arverne*. Mémoire de Master 2, Université Toulouse Paul Sabatier, 73p.

MINISTERE DE L'AGRICULTURE ET DE L'ALIMENTATION (2015). *Agroéquipements et triple performance : freins et leviers pour la transition agroécologique*. Rapport en ligne. URL : https://agriculture.gouv.fr/agroequipements-et-triple-performance-freins-et-leviers-pour-la-transition-agroecologique.

MISSION HAIES AUVERGNE (2015). *Guide technique pour la conception de haies champêtres utiles en agriculture dans le Cantal*. 33p.

MOND'ARVERNE COMMUNAUTE (2018). *Diagnostic du Plan Local d'Urbanisme Intercommunal*.

MUNANG, R., THIAW, I., ALVERSON, K., MUMBA, M., LIU, J. & RIVINGTON, M. (2013). « Climate change and Ecosystem-based Adaptation: a new pragmatic approach to buffering climate change impacts », *Current Opinion in Environmental Sustainability*, 5 (1), pp. 67-71.

NOBLE I. R., HUQ S., ANOKHIN Y. A., CARMIN J., GOUDOU D., LANSIGAN F. P., OSMAN-ELASHA B., VILLAMIZAR A., (2014). « Adaptation needs and options » in FIELD C. B., BARROS V. R., DOKKEN D. J., MACH K. J., MASTRANDREA M. D., BILIR T. E., CHATTERJEE M., EBI K. L., ESTRADA Y. O., GENOVA R. C., GIRMA B., KISSEL E.S., LEVY A.N., McCRAKEN S., MASTRANDREA P.R., WHITE L.L. (eds.), *Climate Change 2014: Impacts, Adaptation, and Vulnerability. Part A: Global and Sectoral Aspects, Contribution of Working Group II to the Fifth Assessment Report of the Intergovernmental Panel on Climate Change*, Cambridge, Cambridge University Press, United Kingdom and New York, NY, USA, p. 833-868.

OSTROM, E. (1990). *Governing the commons: The Evolution of Institutions for Collective Action*. Cambridge University Press, 298p.

OSTROM, E. (2009). « A general framework for analyzing sustainability of social-ecological systems », *Science*, 325, 5939, pp. 419-422.

PALIER, B. (2019). « Path dependence », in BOUSSAGUET, L. (2019). *Dictionnaire des politiques publiques*. 5e édition entièrement revue et corrigée. Paris, Presses de Sciences Po, pp. 446-453.

PERRIN, M. (2016). « Ecologisation de la prospective des territoires au prisme des services écosystémiques : éléments et questionnements à partir de l'enjeu eau », *Sciences Eaux Territoires*, 4, pp. 38-43.

QUENAULT, B. (2013). « Retour critique sur la mobilisation du concept de résilience en lien avec l'adaptation des systèmes urbains au changement climatique », *EchoGéo*, 24.

RICHARD, E. (2013). *L'action publique territoriale à l'épreuve de l'adaptation aux changements climatiques : un nouveau référentiel pour penser l'aménagement du territoire ?* Thèse de doctorat, Université de Tours, 520p.

SANSON, B., BAUDRY, J., NARCY, J.B. (2019). « Ressources en eau, ressources bocagères. Avant-propos », *Sciences, Eaux et Territoires*, 30, pp. 3-7.

SARTHOU, J.P. (2016). « Infrastructure agroécologique : Définition », *Dictionnaire d'Agroécologie*.

SIMONET, G. (2009). « Le concept d'adaptation : polysémie interdisciplinaire et implication pour les changements climatiques », *Natures, Sciences, Sociétés*, 17 (4), pp. 392-401.

SIMONET, G. & DUCHEMIN, E. (2010). « The concept of adaptation: interdisciplinary scope and involvement in climate change », *Sapiens*, 3 (1), pp.

SIMONET, G. (2014). « L'adaptation, champ de recherche à part entière ? Avant-propos », *Sud-Ouest européen. Revue géographique des Pyrénées et du Sud-Ouest*, 37, pp. 13-14.

SIMONET, G. (2015). « Une brève histoire de l'adaptation : l'évolution conceptuelle au fil des rapports du GIEC (1990-2014) », *Natures, Sciences, Sociétés*, 23, pp. 52-64.

SIMONET, G. (2017). « Note de recherche. L'adaptation, un concept systémique pour mieux panser les changements climatiques », *Norois*, 245, pp. 113-125.

SIMONET, G. (2020). « De l'ajustement à la transformation : vers un essor de l'adaptation ? », *Développement durable et territoires*, 11 (2).

SOLTNER, D. (1985). *L'arbre et la haie pour la production agricole, pour l'équilibre écologique et le cadre de vie rurale*. Sciences et techniques agricoles, 199p.

SWIM, J., CLAYTON, S., DOHERTY, T., GIFFORD, R., HOWARD, G., RESER, J., & WEBER, E. (2009). *Psychology and global climate change: Addressing a multi-faceted phenomenon and set of challenges*. A report by the American Psychological Association's task force on the interface between psychology and global climate change. American Psychological Association, Washington.

TORRE, A., AZNAR, O., BONIN, M., CARON, A., CHIA, E., GALMAN, M., LEFRANC, C., MELOT, R., GUERIN, M., JEANNEAUX, P., PAOLI, J.C., SALAZAR, M.I., THINON, P. & KIRAT, T. (2006). « Conflits et tensions autour des usages de l'espace dans les territoires ruraux et périurbains. Le cas de six zones géographiques françaises », *Revue d'Economie Régionale Urbaine*, 3, pp. 415-453.

TORRE, A., KIRAT, T., MELOT, R. & VU PHAM, H. (2016). « Les conflits d'usage et de voisinage de l'espace. Bilan d'un programme de recherche pluridisciplinaire », *L'information géographique*, 80 (4), pp. 8-29.

TROMMETER, M. & WEBER, J. (2004). « Développement durable et changements globaux : le développement durable l'est-il encore pour longtemps ? », *Biodiversité et changements globaux : enjeux de société et défis pour la recherche, Ministère des Affaires Étrangères*, pp. 136-152.

VAN GAMEREN, V., WEIKMANS, R. & ZACCAI, E. (2014). *L'adaptation au changement climatique*. La Découverte, Coll. Repères, 128p.

VAN RUIJVEN, B. J., LEVY, M. A., AGRAWAL, A., BIERMANN, F., BIRKMANN, J., & CARTER, T. R. (2014). « Enhancing the relevance of shared socioeconomic pathways for climate change impacts, adaptation and vulnerability research », *Climatic Change,* 122 (3), pp. 481-494.

VIAUD, V. & THOMAS, Z. (2019). « Une réflexion sur l'état des connaissances des fonctions du bocage pour l'eau dans une perspective de mobilisation pour l'action », *Sciences Eaux Territoires*, 4, pp. 32-37.

WALLACE, K.J. (2007). « Classification of ecosystem services: problems and solutions », *Biological Conservation*, 139, pp. 235-246.

WOLTON, R., POLLARD, K., GOODWIN, A. & NORTON, L. (2014). « Regulatory services delivered by hedges: The evidence base », *Defra and Natural England report LM0106,* 100p.

Table des annexes

Annexe 1 : Ressources bibliographiques 110

Annexe 2 : Extraits du tableau de l’analyse bibliographique 116

Annexe 3 : Guide d’entretien complet 118

Annexe 4 : Supports d’entretien - Fiches services écosystémiques 123

Annexe 5 : Supports d’entretien - Scénarios d’usage des terres (recto-verso) 124

Annexe 6 : Grille d’analyse « modélisation » - Exemple du traitement d'un entretien 125

Annexe 7 : Grille d’analyse « adaptation » - Exemple du traitement d'un entretien 126

Annexe 8 : Engagement de non-plagiat 127

Annexe 1 : Ressources bibliographiques

- **Récapitulatif des documents/sites internet mobilisés**

**Document**	**Source**	**Structure.s porteuse.s**	**Territoire ou portion de territoire concerné.e**	**Thématique.s**	**Caractère juridiquement contraignant**
Plan Local d'Urbanisme Intercommunal (PLUi) : diagnostic, PADD	Services de MAC	Mond'Arverne Communauté	MAC	Urbanisme, aménagement	Oui
Diagnostic territorial 2016-2017	M. Weissgerber	Mond'Arverne Communauté	MAC	Economie, environnement, aménagement, tourisme, social	Non
Projet de territoire	Services de MAC	Mond'Arverne Communauté	MAC	Economie, environnement, aménagement, tourisme, social	Non
Schéma de Cohérence Territoriale (ScoT) du Grand Clermont (PADD + DOG)	Site internet	SM Grand Clermont	MAC	Urbanisme, aménagement, mobilité	Oui
Plan Climat Air Energie Territorial	Services de MAC	Mond'Arverne Communauté	MAC	Energie, climat	Non

Projet Alimentaire Territorial (PAT)	Site internet	SM Grand Clermont et PNR Livradois-Forez	MAC	Agriculture, alimentation	Non
Schéma Régional de Cohérence Ecologique (SRCE) Auvergne	Site internet	Ex région Auvergne	MAC	Trame verte et bleue, réservoirs de biodiversité, corridors écologiques	Non
SRADDET	Site internet	Région AURA	MAC	Trame verte et bleue, réservoirs de biodiversité, corridors écologiques	Oui
Plan Local de l'Habitat Intercommunal (PLH) (délibérations intercommunales associées de 2018 et 2019)	Services de MAC	Mond'Arverne Communauté	MAC (zonages communaux)	Habitat, urbanisme	Oui
Plan Agro-Environnemental et Climatique (PAEC) Veyre Auzon Charlet	Site internet	Syndicat Mixte de la Vallée de la Veyre et de l'Auzon	Communes en bords de cours d'eau	Agriculture, gestion de l'eau, agroécologie	Oui
Charte du PNR Livradois-Forez	Site internet	PNR LF	Manglieu, Sallèdes, Pignols	Urbanisme, aménagement, paysage, gestion des milieux	Non

				naturels, agriculture	
Charte du PNR des Volcans d'Auvergne	Site internet	PNR VA	Aydat, Chanonat, Cournols, Olloix, Saint-Saturnin	Urbanisme, aménagement, paysage, gestion des milieux naturels, agriculture	Non
Loi Montagne	Site internet	État	Pignols, Sallèdes, Manglieu, Chanonat, Saint-Saturnin, Saint-Sandoux, Olloix, Cournols, Aydat	Urbanisme, paysage, agriculture	Non
Schéma d'Aménagement et de Gestion des Eaux (SAGE) Allier aval	Site internet	Commission Locale de l'Eau	MAC	Gestion de l'eau, milieux aquatiques	Oui (directive cadre sur l'eau)
Contrat Territorial des 5 Rivières	Site internet	SMVVA	Bassins versants Assats, Auzon, Charlet, Pignols, Veyre	Gestion de l'eau, milieux aquatiques	Oui
Contrat Territorial du Charlet	Site internet	SMVVA	Bassin versant du Charlet	Gestion de l'eau, milieux aquatiques	Non

Trame Verte et Bleue	Site internet	Grand Clermont (SCoT)	Réservoirs de biodiversité et corridors écologiques	Gestion des milieux naturels, préservation, biodiversité	Non
Zones Natura 2000	Sites internet + PLUi	LPO, CEN	Monts Dore, Chaîne des Puys, Pays des Couzes et Val d'Allier	Gestion des milieux naturels, préservation, biodiversité	Oui
Espaces Naturels Sensibles	Site internet	Département 63	Forêt de la Comté (commune de Vic-le-Comte)	Gestion des milieux naturels, préservation, biodiversité	Oui
Réserve Naturelle Régionale	Site internet	CEN	Puy de Marmant (commune de Veyre-Monton)	Gestion des milieux naturels, préservation, biodiversité	Oui
Plan de gestion des risques naturels	Site internet	Mond'Arverne	MAC	Risques naturels	Oui

- **Retranscriptions d'entretiens par Amélie Menet et Magali Weissgerber : liste des structures interrogées**

	**Structure / institution**

1	ONF
2	DDT
3	IADT
4	Mairie de Cournols
5	Mairie du Crest
6	CEN
7	ONCFS
8	AFB
9	Mond'Arverne Communauté
10	Mairie de Veyre-Monton
11	Mairie de Corent
12	Mairie de Saint Saturnin
13	CRPF
14	SMVVA
15	Fédération départementale PPMA
16	Association Terre de lien
17	DREAL
18	Mairie de Saint-Amant-Tallende
19	LPO
20	Mairie La Roche Blanche
21	Mairie Orcet
22	Chambre d'agriculture
23	Mairie des Martres-de-Veyre
24	Verger de l'Etoile
25	Vergers de Saint-Amant-Tallende en conventionnel
26	Verger (visite)
27	Association les croqueurs de pomme

28	AMAP de Saint-Amant-Tallende
29	Verger (téléphone)
30	CEN vergers
31	Vergers test

Source : Magali Weissgerber (2021).

Annexe 2 : Extraits du tableau de l'analyse bibliographique

Thèmes et sous-thèmes	Enjeux	Références<br>Document/Source	Objectifs envisagés<br>Si disponibles, évolutions envisagées dans le document	Actions potentielles<br>Si disponibles, actions envisagées dans le document	Acteurs concernés (non exhaustif)<br>Permettra de diriger les entretiens/questionnaires	Echelle territoriale	Habitat/Paysage
**Nature**							
Cours d'eau et milieux aquatiques							
	Mise en valeur du val d'Allier	Diagnostic territorial 2016-2017	Aménagement de la voie verte	Diagnostic des thématiques de valorisation, parcours ludique, points de vue, circuits de visite de bourgs, parcours archéologiques	Elus locaux, Grand Clermont	Val d'Allier	Le long de la rivière Allier
		Chambre d'agriculture - journal d'activité Limagne Sud	Conciliation des usages autour de la voie verte (accès aux cultures, motorisation,...)		Agriculteurs	Val d'Allier	Le long de la rivière Allier
**Paysage**							
Urbanisation							
	Eviter coupures paysagères	Diagnostic territorial 2016-2017			Elus locaux, interco, agriculteurs	Mond'Arverne	
	Lutte contre l'uniformisation du paysage (remembrement et étalement urbain standardisé)	Diagnostic PLUi			Interco, élus locaux, CA 63	Mond'Arverne	Entités paysagères : chaîne des Puys, coteaux, défilé du Val d'Allier, faille de Limagne
	Limite l'extension urbaine par rapport à la trame verte et bleue (TVB)	Diagnostic territorial 2016-2017			Elus communes, interco, métropole, Grand Clermont	Mond'Arverne	Cours d'eau, zones humides, réservoirs de biodiversité, corridors écologiques

Agriculture							
éservation des terres agricoles							
	Préservation des milieux agricoles	SAFER	Renouvellement des générations, viabilisation des exploitations et filières, maintien des surfaces agricoles productives	Prioriser l'installation, soutien des filières, politique de reconquête agricole	Agriculteurs, élus locaux, porteurs de projet agricole, interco	Région	Espaces périurbains
	Maîtrise de l'artificialisation des terres agricoles	PAT	Diminution des surfaces fouragères (15000ha en moins) et augmentation de l'artificialisation des sols (8000 ha en plus)		Grand Clermont, SAFER, élus locaux, interco, agriculteurs	Grand Clermont - PNR Livradois-Forez	

Dynamique territoriale							
Tourisme et attractivité							
	Promotion des ressources du territoire et des savoirs faire locaux	PADD	Avoir une démarche d'écologie industrielle et territoriale	Développement de circuits courts, points de vente directe, soutien des entreprises agricoles et artisanales locales	Interco, agriculteurs,		
	Développement des territoires	SAFER	Transmission des biens ruraux, valorisation ressource forestière, réalisation d'infrastructures	Veiller aux conflits d'usage dans les secteurs à habitat dispersé, revalorisation du bâti ancien, accueil de nouveaux arrivants	Collectivités territoriales, élus locaux, agriculteurs, interco	Région	Territoires en déprise économique et démographique

Annexe 3 : Guide d'entretien complet

I. Objectifs du guide

**Objectifs du guide d'entretien**	**Questions associées**
**Pour les modélisateurs**	
Connaître et quantifier les services et disservices écosystémiques (SE/DisSE) d'importance pour l'acteur	2. a) b)<br>3.
Décrire l'état actuel en matière de types de haies (variables utilisées pour la modélisation) et la viabilité de cet état pour l'acteur	4. a) b)
Connaître les préférences de l'acteur en matière de type de haies + leur répartition spatiale	5. 6. 7.
Apprécier la capacité et les leviers d'actions de l'acteur sur le réseau de haies	8.
Sonder les préférences des acteurs parmi plusieurs scénarios d'usage des terres	12. a) b)
Sonder les actions envisagées en lien avec chaque scénario	12. c) d)
**Pour le mémoire**	
Identifier les vulnérabilités et risques perçus en lien avec le dérèglement climatique sur le territoire	9. 10.

Identifier la présence ou la non-présence de stratégies d'adaptation face à ces vulnérabilités	11.

II. Public cible

Agriculteur.rice.s
Elu.e.s des communes (en priorité La Sauvetat, Veyre-Monton, Saint-Saturnin et Manglieu) et de Mond'Arverne
Habitant.e.s et associations (via les rencontres sur le terrain et les contacts)
Chargé.e.s de mission de la Communauté de Communes
Association Mission Haie Auvergne
Conservatoire des Espaces Naturels d'Auvergne
Association Terre de Liens
Chambre d'Agriculture du Puy-de-Dôme

III. Matériaux utilisés

- Fiches illustrées descriptives des services écosystémiques
- Série de photos des haies du territoire, correspondant aux six variables de la modélisation (pas de haie, haie basse, haie basse ponctuée, haie ancienne, alignement d'arbres, haie haute)
- Cartes des sous-territoires choisis (facultatif)
- Jauge d'importance des services et disservices écosystémiques
- Jauge de présence des types de haies
- Fiches illustrées descriptives des cinq scénarios

IV. Série de questions

Introduction : Brève présentation du cadre de l'entretien et du projet.

- Projet de recherche pour développer un outil d'aide à la décision territoriale (sous forme de modèle informatique) en lien avec l'adaptation des écosystèmes aux changements globaux

- Le territoire d'étude est la Communauté de Communes Mond'Arverne, avec quatre communes ciblées
- Dans un premier temps, test de l'outil modélisation sur les haies et occasion d'une première prise de contact avec les acteurs locaux, notamment pour aborder d'autres thématiques en lien avec le projet (scénarios)

A. Les services écosystémiques d'intérêt liés aux haies et leur viabilité

1. [Pas pour les élu.e.s] Voici les quatre communes que nous étudions dans le cadre de notre projet [*montrer cartes communes*].

a) Pouvez-vous me dire si vous habitez, travaillez, ou vous rendez régulièrement sur chacun de ces espaces ? Si oui, pour quelles raisons ?

[Identification du lien au(x) territoire(s) d'étude]

[…]

b) [*Si plusieurs réponses*] Sur quel usage et quelle commune êtes-vous le.la plus à l'aise pour discuter de la gestion des haies ?

[…]

Les questions qui vont suivre porteront donc exclusivement sur la commune que vous venez d'évoquer, en votre qualité de […].

2. Je vais vous présenter plusieurs fiches qui correspondent chacune à une fonction remplie par les haies [*montrer fiches SE*]. Je vous laisse en prendre connaissance.

a) Pouvez-vous placer ces fonctions selon l'importance que vous leur attribuez ? [*montrer la jauge d'importance*].

[Identification des SE d'importance et quantification]

b) Quelles fonctions jugez-vous particulièrement importantes à préserver ?

3. Pensez-vous à d'autres fonctions fournies par les haies qui ne figurent pas parmi ces fiches ?

[Recherche d'une vision exhaustive des SE et DisSE d'intérêt pour l'acteur]

4. Je vais maintenant vous montrer une série de photos avec six types de haies différents [*montrer les photos et expliquer les six stades*].

a) Parmi ces photos, lesquelles se rapprochent le plus du réseau de haies que vous pouvez observer sur la commune de […] ou Mond'Arverne ? Est-ce que ce type de haies vous semble satisfaisant ?

[...]

b) Permet-il, selon vous, d'assurer les fonctions liées aux haies qui vous semblent les plus importantes ? *[les énoncer de nouveau, maxi 3 SE].*

[Sondage de la viabilité de l'état actuel]

[…]

5. A présent, pouvez-vous écarter les photos qui ne reflètent pas du tout vos préférences en matière de haies, et laisser celles qui vous semblent satisfaisantes ?

[Sondage des états non-acceptables]

[…]

6. Pour les photos qui restent, pouvez-vous les classer dans l'ordre de vos préférences ?

[Bornes min et max des variables de la modélisation]

7. Quelle serait la répartition idéale des différents types de haies sur la commune, hors zone urbanisée ? [*Classer à l'aide de la jauge de présence]*

[Bornes min et max des variables de la modélisation]

8. Avez-vous la sensation d'être en capacité d'agir pour garantir les fonctions liées aux haies que nous avons évoqué ? Si oui, comment ? Si non, pourquoi ?

[Espace des contrôles]

B. Vulnérabilités et stratégies d'adaptation au changement climatique :

9. Pensez-vous que votre territoire est vulnérable face au dérèglement climatique ? Si oui, quels évènements ou observations vous permettent d'affirmer cela ?

[Perception des risques actuels]

10. Quels risques futurs liés au changement climatique anticipez-vous à l'échelle du territoire ?

[Perception des risques futurs]

11. Face à ces risques, mettriez-vous des actions particulières en place à titre personnel ? Si oui, quoi ? Que souhaiteriez-vous voir être mis en place à l'échelle collective ?
[Stratégies d'adaptation]

C. Scénarios d'usage des terres

12. Voici plusieurs scénarios d'usage des terres, basés sur plusieurs documents institutionnels de la Communauté de Communes Mond'Arverne [*montrer les fiches scénarios et les expliquer une à une, avec la provenance des données*].

a) Parmi eux, y a t-il des scénarios que vous ne souhaiteriez-pas voir se réaliser sur le territoire ?

b) Lequel, ou lesquels, vous semblent les plus prioritaires à mettre en œuvre ? Pourquoi ?

c) Quelles actions vous semblent prioritaires à mettre en œuvre pour réaliser ce.s scénario.s ?

d) Quelles actions vous semblent pouvoir répondre à plusieurs scénarios en même temps ?

Annexe 4 : Supports d'entretien - Fiches services écosystémiques

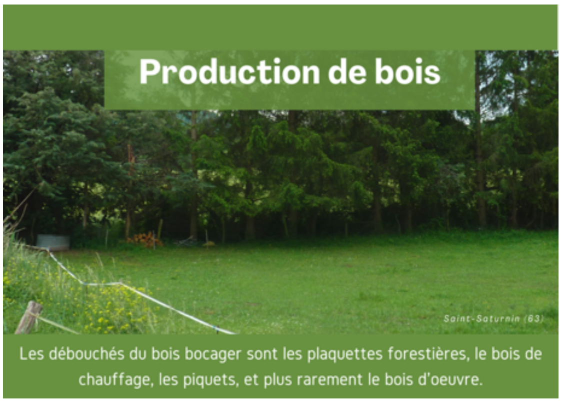

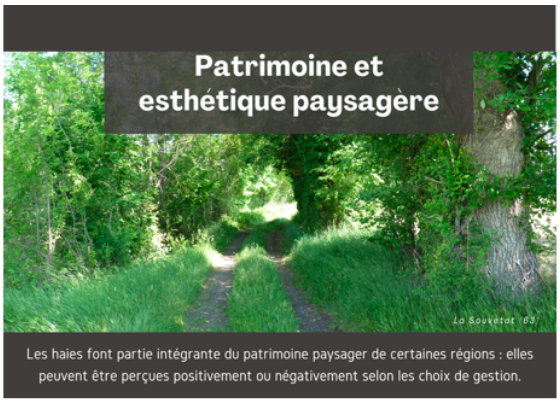

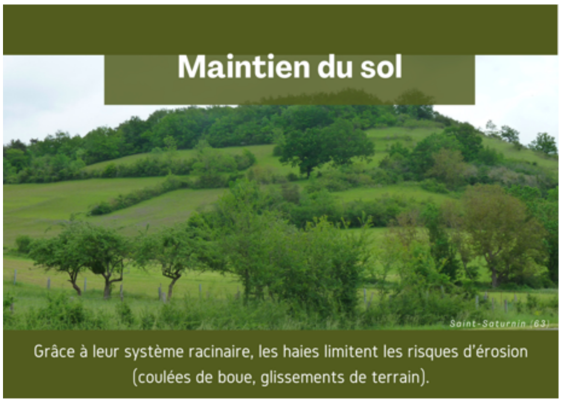

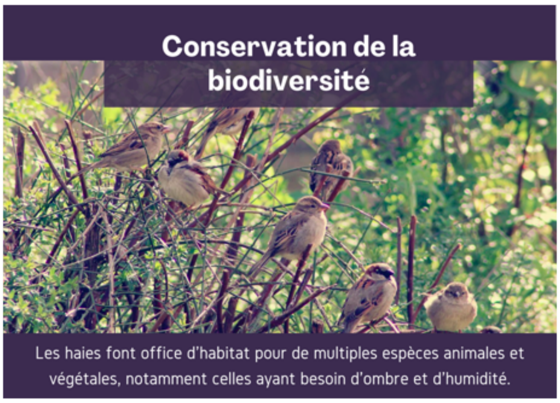

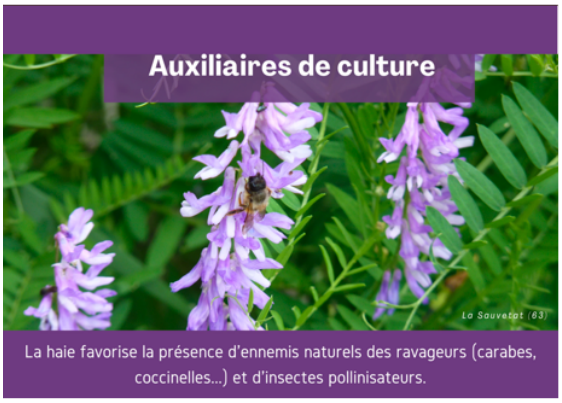

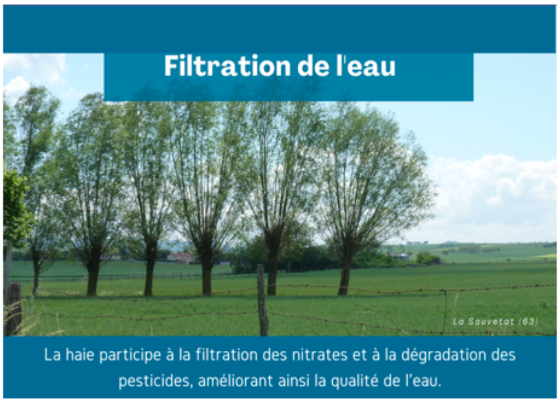

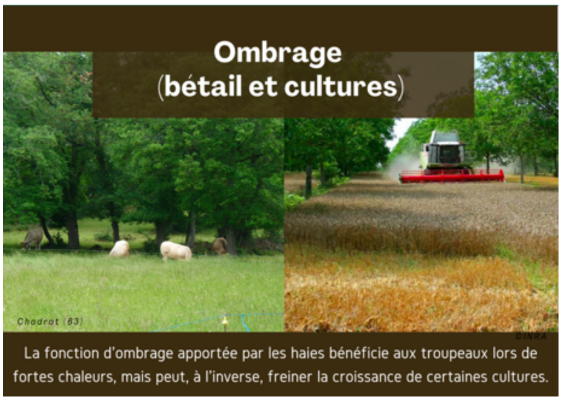

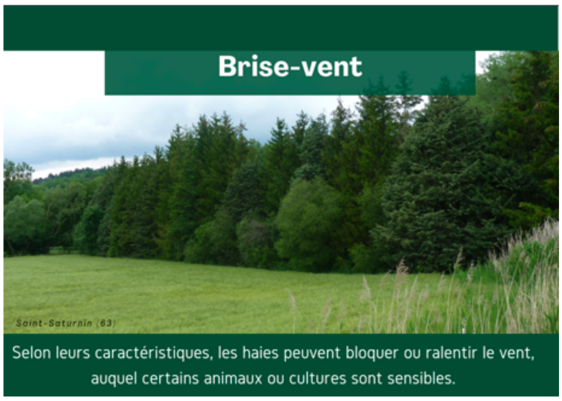

Annexe 5 : Supports d’entretien - Scénarios d’usage des terres (recto-verso)

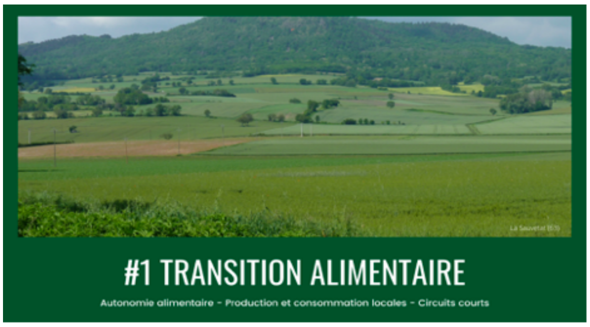

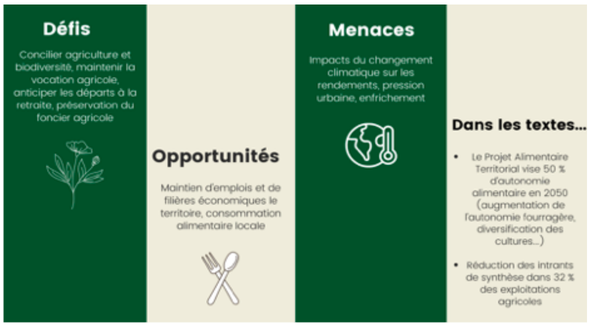

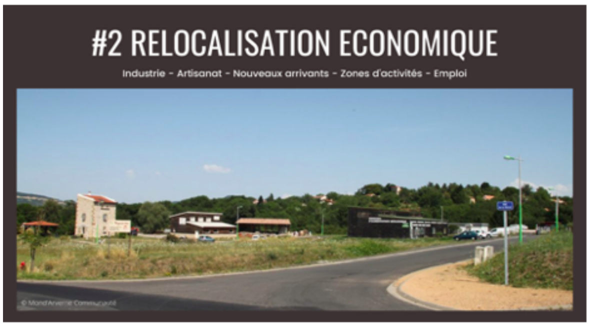

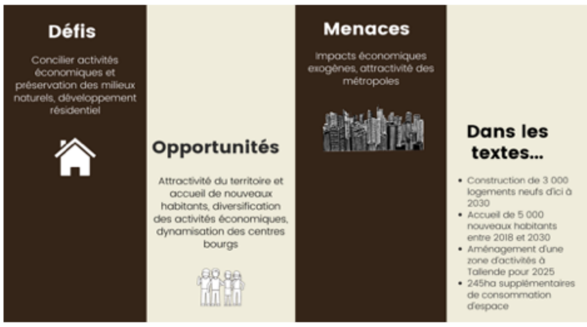

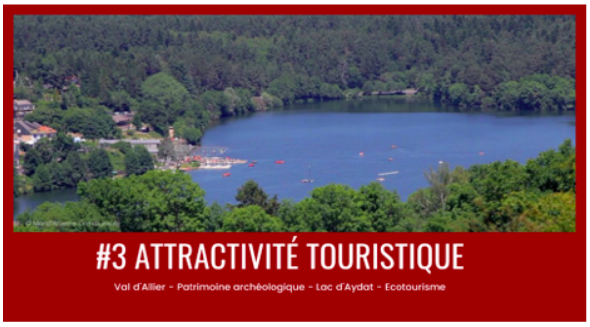

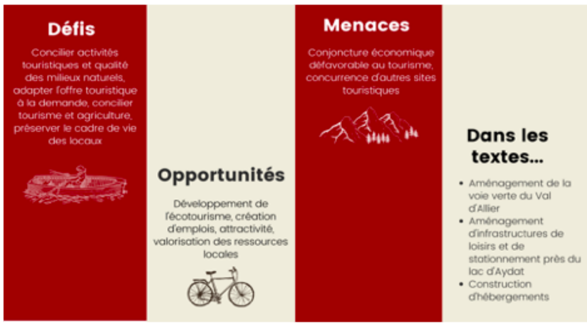

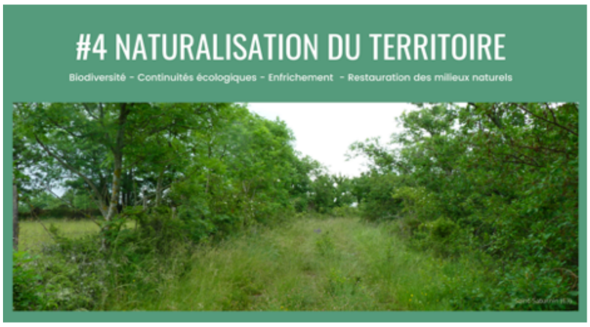

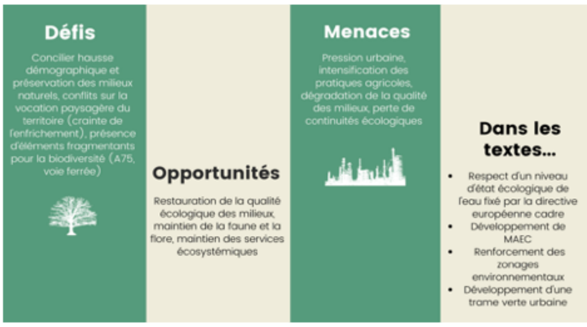

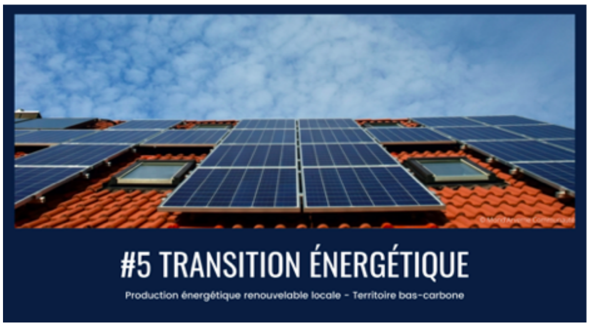

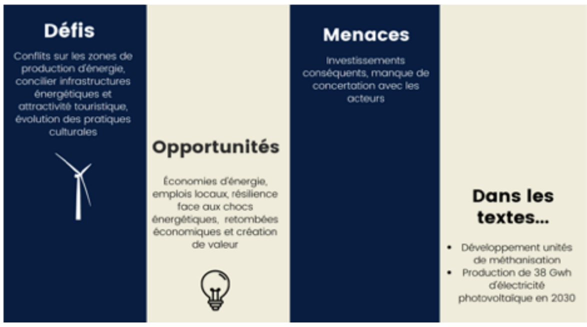

Annexe 6 : Grille d’analyse « modélisation » - Exemple du traitement d'un entretien

Identité				
ID	Nom	Date	Fonction	1. Territoire d'étude
1	x	15/07/21	x	Veyre-Monton

A. Haies et services écosystémiques								
2.a Importance des services (note pour chaque service)	2.b Services importants à préserver (pour chaque service, 1 = oui, 0 = non, 2=NSP)	3. Autres fonctions	4.a Réseau de haies (si possible, proportion)	4.b Satisfaction du réseau actuel (1 = Oui, 0 = Non, 2 = NSP)	4.c Effectivité des services (1 = Oui, 0 = Non, 2 = NSP)	4.d Commentaires	5. Préférences catégories de haies (1 = S, 0 = NS 2=NSP)	6. Ordre de préférences (du moins vers le plus, entre parenthèses si égalité)
43444454	11111111	Service lutte contre la formation des congères	Pas trop de haies anciennes	1	0	Perte de haies depuis plusieurs années. Terrains privés donc impossibilité d'intervenir	.011111	31(245)

A. Haies et services écosystémiques						
4.d Commentaires	5. Préférences catégories de haies (1 = S, 0 = NS 2=NSP)	6. Ordre de préférences (du moins vers le plus, entre parenthèses si égalité)	7. Répartition des catégories de haies	8. Capacité d'action + commentaires	9. Gestion des haies comme enjeu? (1 = Oui, 0 = Non, 2 = NSP)	Commentaires sur les enjeux
Perte de haies depuis plusieurs années. Terrains privés donc impossibilité d'intervenir	.011111	31(245)	NSP	Oui (Ajout haies en bord de route pour la sécurité)et Non (terrains privés). Peut-être dans les chemins communaux; mais l'entretien des haies est coûteux	1	• Coulées de boue et écoulements divers, Congères et verglas sur les routes car fortes expositions au vent du fait du manque de couvert végétal, Filtration de l’eau (au niveau quantitatif), Trame bleue gérée par le SMVVA au bord de la Veyre, gestion des ripisylves, Trame verte et passage de la flore et faune, abordée dans le PLUi, gestion des continuités écologiques, Réduction des risques routiers

Annexe 7 : Grille d’analyse « adaptation » - Exemple du traitement d'un entretien

3. Scénarios d’usage des terres			
3.1. N° sélectionné(s)	2-3		
3.2. Priorisation des scénarios dans le temps	2-3, 1-4-5		
3.3. Caractérisation des actions prioritaires	**1.i. (S3)**	**1.ii. (S2)**	
3.3.1. Description	Mise en valeur sites touristiques	Aménagement zones d’activités avec normes environnementales	
3.3.2. Échelle spatiale	Circonscrite (Val d’Allier, Gergovie	Circonscrite (Pra de Serre, Tallende, Meules)	
3.3.3. Échelle temporelle	Moyen terme	Moyen terme	
3.3.4. Nature des actions	Grise, soft (communication)	Grise, soft (politique d’attractivité)	
3.3.5. Acteurs associés	Public (CC, communes), privé (acteurs touristiques)	Public (CC, communes, EPF), privé (entreprises)	
3.4. Caractérisation des actions synergiques			
3.4.1. Description	Agir sur les mobilités		
3.4.2. Échelle spatiale	Globale (CC)		
3.4.3. Échelle temporelle	Moyen terme		
3.4.4. Nature des actions	Grise (travaux voirie), soft (sensibilisation)		
3.4.5. Acteurs associés	Public (CC, SMTC, Région), associations de mobilité		
3.5. Conflits d’usage des terres présents et/ou latents	**1.a.**	**1.b.**	**1.c.**
3.5.1. Description	Problème de dialogue pour la végétalisation de zones agricoles sensibles aux évènements climatiques extrêmes	Densification du bâti en zone urbaine qui pose des problèmes d’écoulement de l’eau	Panneaux photovoltaïques au sol vs enjeux biodiversité
3.5.2. Localisation	Versant de Soulasse	Veyre-Monton	X
3.5.3. Acteurs	Agriculteurs, commune	DDT, communes	X
3.5.4. SNI concernées	Bandes enherbées, haies, terres agricoles	Jardins	X
3.5.5. Services écosystémiques associés	Maintien du sol	Filtration de l’eau	X

	A	B	C	D
1		**1**		
2	**1. Impacts du dérèglement climatique et enjeux d’adaptation**			
3	1.1. Perception de vulnérabilités	Oui		
4	1.2. Description	Episodes orageux plus intenses et fréquents, coulées de boue, forte exposition au vent		
5	1.3. Localisation des vulnérabilités (sous-territoire, commune…)	Commune et ST (versant de Soulasse, route du Crest, route du Pra de Serre)		
6	1.4. Type de vulnérabilités (socio-économique, écologique)	Socio-économique (dégâts matériels habitations)		
7	1.5. Infrastructures semi-naturelles (SNI) affectées	X		
8	1.6. Services écosystémiques affectés	X		
9	1.7. Impacts anticipés	Hausse fréquence des sécheresses, risques de crues		
10				
11	**2. Caractérisation des actions d’adaptation**	**1.1.**	**1.2.**	**1.3**
12	2.1. Description	Travaux de reméandrage Veyre	Aménagement zone humide	Végétalisation urbaine
13	2.2. Échelle spatiale (globale, circonscrite)	Circonscrite (Veyre, LD Les Sagnes)	Circonscrite (amont lac d’Aydat)	Circonscrite (commune de VM)
14	2.3. Échelle temporelle (ponctuelle, long terme)	Ponctuelle	Ponctuelle	Moyen terme
15	2.4. Nature des actions (« grise », « verte », « soft »)	Verte	Verte	Verte
16	2.5. Acteurs associés (public, privé)	Public (SMVVA, communes), privés (agriculteurs, propriétaires fonciers)	Public (SMVVA, commune d’Aydat, Région, Dpt 63, DREAL, ARS, PNRVA…)	Public (commune) et associatif (MH)
17	2.6. Conception des actions (sans regret, co-bénéfices, flexibles…)	Sans regret, co-bénéfices	Sans regret, co-bénéfices	Co-bénéfices
18				

Annexe 8 : Engagement de non-plagiat

Je soussignée Elise KRIEF, étudiante en Master Gestion des Territoires et Développement Local, déclare être pleinement consciente que le plagiat de documents ou d'une partie de documents publiés sur toutes formes de support, y compris internet, constitue une violation des droits d'auteur ainsi qu'une fraude caractérisée. En conséquence, je m'engage à citer toutes les sources que j'ai utilisées pour écrire ce mémoire.

Le 29 octobre 2021, à Issoire

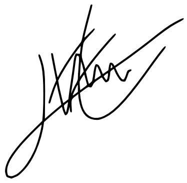

Supplement: Supplemental Information 1 — This compressed file contains: (1) A spreadsheet Presenting the details of the 4th tier SESF analysis, (2) A pdf file of the semi-structured interview guide that was used to obtain some of the SES variables. The unstructured part of the interviews contained identifiable personal information and thus was removed. It can be shared on demand only for actors who sign a sharing-consent agreement. (3) A pdf file in French language of the original M.S. report presenting elements of social-ecological analysis of the study site that was conducted during 2021. It includes the analysis of local and regional gray literature and anonymized interviews with some local stakeholders. This data represent one of the source of information for performing the socio-ecological system (SES) framework analysis across the two sites under investigation. The analysis of stakeholders encompassed both qualitative and quantitative data, facilitating an examination of their capacity to identify various types of hedgerows, their relationships with these features, preferences regarding ecosystem services associated with hedgerows, and their inclinations toward different governance arrangements and developmental trajectories. Details regarding the survey methodology and utilized documents are provided within the document. The Author of the report is Elise Krief, M.S. [file peerj-13-18938-s001.zip › Original.Report.social-ecological analysis of the study site (French).pdf]
