# Supplementary material for: Integrating adaptation pathways and Ostrom’s framework for sustainable governance of social-ecological systems in a changing world
Source: PeerJ. 2025 Feb 24;13:e18938. doi: 10.7717/peerj.18938 (PMC11867035; doi:10.7717/peerj.18938)
Supplement: Supplemental Information 2 — Details of the methodological implementation for the SES case studies in the French Auvergne Region. It also includes Tables S1 and S2. [file peerj-13-18938-s002.pdf]

Supplementary S2: Methodological implementation to the case study and simplifying assumptions

Step 1. Using the SESF for defining the set of actions based on adaptation targets

Check supplementary S1 for the detailed 4th tier SESF analysis of the study site.

Step 2. Using the CISF for a systemic organization of actions between roles

Step 2.2. Nine scenarios used for the study site

Step 2.2.1. four scenarios of constitutional-choice arrangements

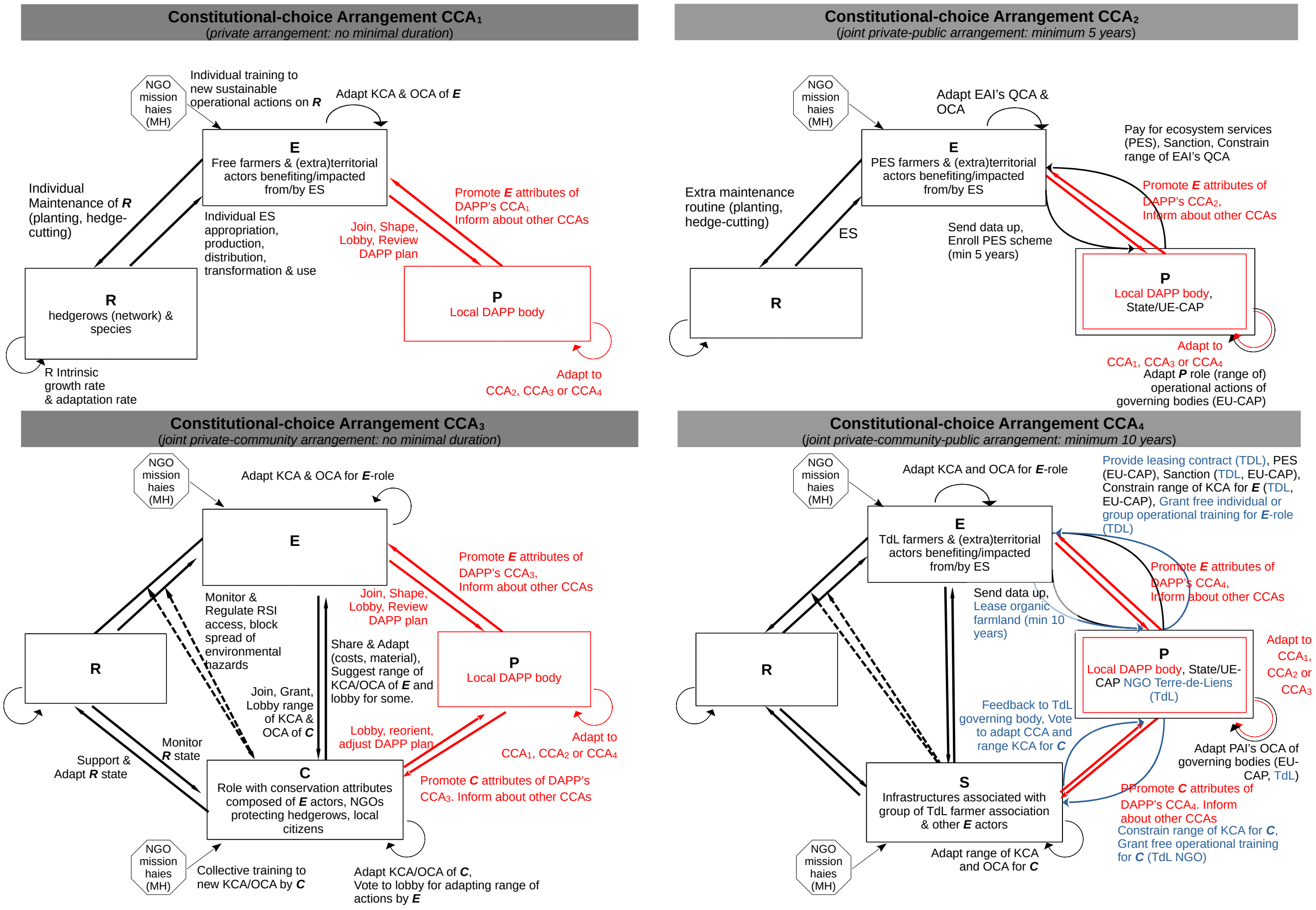

Figure S1. CISF illustration of the four scenarios of constitutional-choice arrangements (CCA) used as a basis for defining possible adaptations of the distribution of authority. The four scenarios were defined from interviews of local actors and documents associated with the study sites. CCA_1 was the dominant scenario recorded/present in the studied SES, while the other three were sporadically detected but frequently suggested for implementation based on interviews with local actors, or on strategic territorial planning documents. Each CCA has its own structural and time constraints, and permit different forms of collective-choice (KCA) and operational choice (OCA) arrangements (see table 2). RSI means resource species and infrastructures. Role compartment ***E***, ***C*** and ***P*** respectively refer to exploiting, conservation and policy-making actors and infrastructures. The red-colored compartments and arrows indicate the ***P***-role involved in the DAPP process; while the black or blue-colored ones refer to those involved in other conservation actions (CCA_3 and CCA_4) or arbitration (CCA_4).

Step 2.2.1. Nine scenarios collective-choice arrangements and associated operational-choice arrangements

Table S1: Set of constitutional, collective, and operational choice arrangements (respectively referred to as CCA, KCA and OCA in the table) for the study site located in the Auvergne region of France. The four CCA refer to the one presented in figure 2 and figure S1. For every CCA, several KCA are associated with different objectives, constrains, network/chains of actions, costs and benefits, and one fixed OCA. The codes $\boldsymbol{U_{1a}}$, $\boldsymbol{U_{0a}}$, and others in the chains of actions refer to the CIS encoding presented in figure 1.

Constitutional-Choice arrangements (CCA)			Associated Collective-Choice Arrangements (KCA)			Consequences on the operational choice arrangement (OCA) for the *E*-role: proportion of every type of action U_{1a}			Effect of the OCA on the resource infrastructure (U_{0a})	Relative cost reduction provided by the KCA to compensate the disservice cost of management (hedgerows).
Name	Description	Minimal duration	#	objectives	Method (chain of collective actions) to achieve the objective	No maintenance	Hedge-cutting (tractor mounted)	Integrative soft management		
(1) Private arrangement	**Individualistic social organization (current dominant practice):no** incentives or collective actions to support the hedgerow network or regulate actions. Most common practice is trimming. We assume that this action is included in the SES that was observed during the 1989-2019 period.	no	KCA_1	Collective-choice arrangement for baseline monitoring and range of operational actions	#NA	0.2	0.6	0.2	Business-as-usual dynamics (#NA)	0%
(2) Joint Private-Public arrangement	**Social organization and infrastructures around state-controlled scheme for the payments for ecosystem services (PES):** The state pay farmers who enter a PES scheme, under the condition that they maintain hedgerows and associated ES	5 years	KCA_2	Compulsory planting of species-rich hedgerows to increase public & common-good ES	$U_{2b} \rightarrow U_{1a} \rightarrow U_{0a} \rightarrow U_{1b}$	0.2	0.6	0.2	More planting of species-rich hedgerows: Tall +5%, Short +5%	-10%
			KCA_3	Compulsory planting of species-rich hedgerows under integrated soft management (constrain the on tractor hedge-cutting use) to protect public & common-good ES	$U_{2b} \rightarrow U_{1a} \rightarrow U_{0a} \rightarrow U_{1b}$	0.2	0.4	0.4	#NA	0%
(3) Joint Private-Community arrangement	**Social organization and infrastructures around the support and regulation of the hedgerow network, ecosystem services and exploiting actors:** Neighboring farmers enter joint private-community arrangement by forming an auxiliary association (AIA) to set auxiliary practices regarding monitoring, the sharing of material/costs/knowledge, and set operational constrains on PIA appropriation practices (e.g. on planting new hedgerows, tractor hedge-cutting, integrated soft management).	no	KCA_4	Incentives to share material and reduce costs in order to reduce constrains on tractor hedge-cutting	$U_{6a} \rightarrow U_{1a} + U_{1b}$	0.1	0.8	0.1	#NA	-10%
			KCA_5	Regulate the planting to increase poor-species hedgerows in order to increase biomass production	$U_{5a} \rightarrow U_{1a} \rightarrow U_{0a} \rightarrow U_{1b}$	0.2	0.6	0.2	More planting of productive species in species-poor hedgerows: Tall: +5%, Short+5%	0%
			KCA_6	Regulate the use of tractor hedge-cutting + Incentives on the planting of species-rich hedgerows + integrated soft management practices on these hedgerows	$U_{5a} \rightarrow U_{1a} \rightarrow U_{0a} \rightarrow U_{1b}$	0.2	0.4	0.4	More planting of species-rich hedgerows: Tall +5%, Short +5%	0%
			KCA_7	Strongly regulate maintenance activities for reducing costs and increase nature conservation	$U_{5a} \rightarrow U_{1a} \rightarrow U_{0a} \rightarrow U_{1b}$	0.4	0.4	0.2	#NA	0%
(4) Joint-Private-Community-Public arrangement	**Social organisation and infrastructure for governing, arbitrating between exploiting and supporting actors and infrastructures:** Farmers join organic farming NGO Terre-de-liens (TdL) and the EU-CAP sponsored PES scheme. Farmers lease land to TdL with extra individual regulations and benefits for the management of their own hedgerows. Farmers can join the TdL SAI group that receive funds from TdL to collectively protect the hedgerow network	10 years	KCA_8	Regulate the use of tractor hedge-cutting, set incentives to plant more species-rich hedgerows that require integrated soft management practices	$U_{2b} \rightarrow U_{5a} \rightarrow U_{1a} \rightarrow U_{0a} \rightarrow U_{1b}$	0.2	0.4	0.4	More planting of species-rich hedgerows: Tall +5%, Short +5%	-20%
			KCA_9	Strongly regulate maintenance activities & set cost sharing incentives to increase nature conservation	$U_{3b} \rightarrow U_{5a} + U_{6b} \rightarrow U_{1a} \rightarrow U_{0a} \rightarrow U_{1b}$	0.4	0.4	0.2	#NA	-20%

Table S2. Model parameters for seven ecosystem (dis)services produced by four types of hedgerows, along with the costs of operational maintenance actions and the differing needs in rural and peri-urban areas. Ecosystem (dis)services were evaluated using simple, semi-quantitative metrics from the literature. See details in Supplementary S1.

Ecosystem (dis)services (ES) (i.e. link 1b in the CIS model from figure 1)	Weighted limits of satisfaction for every ES deduced from the actors surveyed in the two SES [0-1]		Relative production of ES by type of hedgerow, on a [0-1] scale, with 1 corresponding to the observed maximal production or effect of the ES					Impact of maintenance operational action on ES, on a [0-1] scale, with 0 corresponding to a total degradation of the ES, whereas 1 meaning a perfect conservation of the ES.			Quantitative indicator of ecosystem services (and origin of indicator and measurement)
	Rural SES (La Sauvetat)	Peri-urban SES (Veyre-Monton)	No hedgerow	Short hedgerow (SH)		Tall hedgerow (TH)		No maintenance	Hedge-cutting (tractor mounted)	Integrative soft maintenance	
				Species-poor (PH)	Species-rich (RH)	Species-poor (PH)	Species-rich (RH)				
Fruit production	0.9	0.95	0	0.553	1	0.3169	0.9859	0.5	0.25	0.75	Mean number of edible fruits species (based on ecological survey)
Pollination	0.9	0.95	0	0.5204	0.9959	0.2353	1	0.5	0.25	0.75	Mean number of species attracting pollinators (based on ecological survey)
Biomass Production	0.95	0.5	0	0.25	0.25	1	0.75	1	0.5	0.75	Aerial carbon (Open data from Carbocage
Sunlight Protection	0.95	0.5	0	0.3231	0.3508	0.938	1	0.75	0.25	0.5	Mean height (based on ecological survey)
Landscape aesthetics	0.5	0.95	Aesthetic opinion of actors changes with the diversity of hedgerow present in the landscape.					0.5	0.25	0.75	Shannon index (calculated from the relative proportion of the four hedgerow types present in the landscape, and confronted to opinions of actors based on social survey)
Maintenance cost (social-economic cost)	0.1	0.1	0	0.25	0.25	0.75	1	0	0.5	0.75	Mean annual maintenance cost
Environmental hazards: fire, lateral encumbrance, snag fall, etc …	0.15	0.2	0	0.25	0.25	0.75	1	1	0.5	0.5	Based on expert knowledge

S2.1. Model parametrization and simplifications to match our case study

In the study area, situated in the Auvergne region (central France), we focused on the impact of the hedgerow dynamics of various ecosystem (dis)services. The implementation of the renewed CIS resulted in further parametrizations and simplifications.

S2.1.1. RSI Model of hedgerows network dynamics

The RSI represented the hedgerow network. It was decomposed into four hedgerow types (and a fifth extra empty state), each characterized by their height (short, tall) and biodiversity states (rich, poor plant species richness) (see Supplementary S1). The height and extent of hedgerows were defined through a GIS analysis of aerial photographs of representative parts of the studied areas in 1958, 1989, 2009 and 2019, together with field observations for plant species richness (cf. Supplementary S2). We deduced the annual average transition and stasis rates between the five hedgerow states (Supplementary S1), and then the transition matrix $\boldsymbol{M}$ that was used as a baseline model for projection of our scenarios of hedgerow network dynamics, associated with CCA_1 (figure S1). Given the discrete nature of this model, the RSI dynamics in equation 1 (main text) was also discretized.

S2.1.2. Model of ecosystem services, costs & benefits of adaptation actions

Each hedgerow state was characterized by the production of different levels of seven ecosystem (dis)services, postulated as shown in table S2. We made the assumption that the entire ES produced by hedgerows equals the ES outflow effectively used by ***E***-role, such that $\boldsymbol{U_{1b}} = 1$ in equation 1 (main text). Based on interviews with local stakeholders and regional strategic documents (Supplementary S1), we characterized archetypal needs and expectations of stakeholders in each SES. We found that two rural and peri-urban areas had somehow different needs and expectations regarding ecosystem (dis)services (table S2).

S2.1.3 Specific parametrization for the different governance arrangements and actions

In our case study, and for reducing complexity, every KCA resulted into one and only one OCA. Every combination of KCA/OCA involved a unique set of constraints on the permissible range and intensity of exploitation and management actions on hedgerows (cf. action link $\boldsymbol{U_{1a}}$ and $\boldsymbol{U_{1b}}$ in figure 1 in main text and in table S1), of supporting actions between C-role and ***R*** or ***E***-role (cf. $\boldsymbol{U_4}$ to $\boldsymbol{U_6}$), and of policy-making actions between PAI and other social compartments (cf. $\boldsymbol{U_2}$ and $\boldsymbol{U_3}$, and table S1).

At an operational-choice (OCA) level, the actions changed with the different types of hedgerows, affecting the level of production of the seven studied ecosystem (dis)service (Supplementary S1, table S2).

At a collective-choice level (KCA), not all the information could be obtained from interviews for the chains of actions presented in table S1. The same applied for the CCA level (table S2). We thus made further simplifying assumptions to eq. 1 (main text) as followed:

At a constitutional-choice (CCA) level, we assumed perfect community grouping for the $\boldsymbol{CCA_3}$ and $\boldsymbol{CCA_4}$ ($\boldsymbol{U_{6a}} = 1$ and $\boldsymbol{U_{6b}} = 0$ in equation 1 of main text). For $\boldsymbol{CCA_2}$ and $\boldsymbol{CCA_4}$, we also assumed perfect feedback from farmers to the European union (EU-CAP) and/or the NGO *Terre-de-Lien* TdL ($\boldsymbol{U_{2a}} = 1$). For $\boldsymbol{CCA_3}$ and $\boldsymbol{CCA_4}$, we assumed a perfect monitoring of the resource state ($\boldsymbol{U_{4a}} = 1$), and a perfect monitoring ($\boldsymbol{U_{5b}} = 1$) of the access rate $\boldsymbol{U_{1b}}$ of ***E***-role (farmers, local population) to get some of the ES,

or to manage hedgerows to produce ES ($\boldsymbol{U_{5a'}} = 1$ on $\boldsymbol{U_{1a}}$). Finally, for $\boldsymbol{CCA_4}$, we assumed a perfect feedback from $\boldsymbol{C} \rightarrow \boldsymbol{P}$ ($\boldsymbol{U_{3b}} = 1$). In our case, this simplification applied to TdL and DAPP board committees. We finally assumed a perfect human or economic support to the ***C***-role by ***P***-role ($\boldsymbol{U_{3a}} = 1$).

We also considered qualitative rather than quantitative change in ***E***, ***C*** and ***P*** role attributes in eq. 1. We accordingly simplified eq. 1 (main text), by considering constant the ***E***, ***C*** and ***P*** state (e.g. population number or finances), such that $dE/dt = 0$, $\boldsymbol{E} = 1$, $\boldsymbol{U_{0b}} = 0$; $dP/dt = 0$, $\boldsymbol{P} = 1$, $\boldsymbol{U_{0c}} = 0$, $dP/dt = 0$).

S2.1.4 Resulting context-specific model after simplifications

These assumptions led to the simplified model (equation S1) that we used for our case study, as per eq. 2 below:

$$
\begin{cases}
\dfrac{dR}{dt} = \overbrace{U_{0a}.R}^{\text{Natural ES Growth}} - \overbrace{U_{7b}.R}^{\text{Climate Stress}} + \overbrace{U_{4a}.R.C}^{\text{C Support}} \\
\quad - \overbrace{R.E.C}^{\text{Perfect ES Access, Flow \& Regulation}} + \overbrace{U_{1a}.E.R}^{\text{Access \& Management}} . \overbrace{U_{5a}.C}^{\text{Regulated}} \\
\dfrac{dE}{dt} = - \overbrace{U_{0b}}^{\text{Natural E Decay}} + \overbrace{R.C}^{\text{Perfect ES Access, Flow \& Regulation}} \\
\quad + \overbrace{U_{6b'}.C}^{\text{E Support}} + \overbrace{U_{2b}.P}^{\text{P Support}} = 0 \\
\dfrac{dC}{dt} = - \overbrace{U_{0d}}^{\text{Natural C Decay}} + \overbrace{U_{1b}.R.E}^{\text{Perfect Access \& ES Flow Monitoring}} + \overbrace{P}^{\text{Perfect P Support}} \\
\quad + \overbrace{U_{1a}.R.E}^{\text{Perfect Access \& Management Monitoring}} + \overbrace{E}^{\text{Perfect E Participation}} = 0 \\
\dfrac{dP}{dt} = - \overbrace{U_{0c}}^{\text{Natural P Decay}} + \overbrace{E}^{\text{Perfect E Feedback}} + \overbrace{C}^{\text{Perfect C Feedback}} = 0
\end{cases}
$$

S2.2. Model of climate stress impact on the hedgerow network

We assumed the drought stress to evenly affect the four types of hedgerows from the RSI, through variable $\boldsymbol{U_{7b}}$ in eq. 1 (or see figure 1 in main manuscript). We assumed an increased mortality rate of the hedgerow woody plant species with increasing drought stress (Barros, 2017). Level 0 represented no additional climate stress ($\boldsymbol{U_{7b}} = 0$), level 1 a moderate drought stress causing 1.2% annual mortality rate ($\boldsymbol{U_{7b}}$ = - 1.2%.0a), and level 2 a high drought stress causing 2.4% annual mortality rate ($\boldsymbol{U_{7b}}$ = - 2.4%.$\boldsymbol{U_{0a}}$).

S2.4.2. Simplifications associated with the definition of the set of robust trajectories that respect *K*

Without changing the implications, and as specified in step #3, we simplified eq. 5 (main text) for our case study to only consider the viability estimation involving adaptive controls between two levels

(CCA and KCA). For every KCA, one OCA was fixed (composed of three operational actions, see table S1). Every OCA was composed of three operational actions on the hedgerow network, whose intensity was fixed. It means that triggering an action that changed a KCA automatically led to a unique change in the OCA (cf. table S1 and S2).

S2.5. Deducing the DAPP map within the subset of viable solution

S2.5.3. Deduction of specific DAPP maps that emphasize different priorities

In our case study, once the existence of viable pathways and viable DAPP map are proved, three different types of DAPP maps can be produced, each emphasizing different priorities: (i) Certainty DAPP maps (figure 4, main text) emphasize the probability of finding long-term 30 years viable pathways of adaptation, passing by all the possible 5 years sequence between two successive decision nodes; (ii) Most secured DAPP maps (figure 5, main text) result in this work from the selection of the subset of the 10 most secured options of viable adaptation pathways;(iii) Optimal DAPP maps (figure 6 in main text) as the subset comprised of the seven pathways that maximize only one of the five ES^+ and minimized only one of the two ES^-. We supplemented this analysis by decomposing the effect of optimizing every ES on the other ES (figure 7 in main text).

S2.6. Estimating the sensitivity of the DAPP maps to changes in operational actions on hedgerow network

We then retrospectively analyzed the sensitivity of specific ES outcomes along the DAPP map. The most common approach is to represent and compare $\boldsymbol{Viab_K}$ calculated in the ES state-space and then deduce DAPP maps accordingly. However, the estimation of the former was not possible for computational reasons, and only $\boldsymbol{Viab_K}$ in the state space of the hedgerow types could be calculated (cf. Supplementary S3). To get information regarding ES, we thus had to separate the analysis in to two sub-steps. Frstly, analyzing $\boldsymbol{Viab_K}$ into the hedgerow space, and secondly perform a retrospective sensitivity analysis of the impact of changing the hedgerow types on the provisioning of every ecosystem services. The method to achieve this is described below.

S2.6.1. Evaluating the viability impact of changing hedgerow types on pathway viability

For the two SES types (rural, peri-urban) and the three climate stress levels (0, 1, 2), we first analyzed the size and shape of $\boldsymbol{Viab_K}$ (eq. 5 in main text) within the state-space associated with the four hedgerow types: tall hedgerows (TH), short hedgerows (SH), species rich (RH) and species poor (PH), via eq. 5. The state-space's size and shape are good indicators of the number and types of adaptation pathways. We thereafter produced bi-dimensional slices of $\boldsymbol{Viab_K}$ within the four-dimensional state-space, to represent $\boldsymbol{Viab_K}$ for RH vs PH (figures 9 in main text) and TH vs SH (figures S9 in supplementary S5). They describe the combinations of hedgerow types, expressed as percentages, necessary to ensure the viability of adaptation pathways

We further pinpointed the initial state's position at $\boldsymbol{t_0}$ (i.e. time when we did the SES analysis of the study sites in 2020) within $\boldsymbol{Viab_K}$, as an indication of whether the current SES state is inside, outside or near the frontier of $\boldsymbol{Viab_K}$, and therefore requires governance adaptation. For example, a small volume, asymmetrically shape $\boldsymbol{Viab_K}$(CCA|KCA|OCA) and an ES state very close to $\boldsymbol{K}$ within, both suggest that

the SES is not secured, as stakeholders need to adapt CCA, KCA, OCA to stay viable, but with more limited options of new hedgerow type proportions at hand.

S2.6.2. Evaluating the ES security gains when changing hedgerow types

We retrospectively analyzed for all the adaptive governance pathways ***u(.)*** *(eq 6* in main text*)*, how much security gains (i.e. increase in the distance from the constraint ***K*** for every $ES^+_{i,min}$ or $ES^-_{j,max}$) were gained by switching operational actions from one hedgerow type to another (TH, SH, RH, PH).

For instance for the pathways that assume a set of operational actions on tall hedgerows (RH) (i.e. $u^{OCA}_{RH}(.)$), we first estimated the distance *ΔES* between the value of ES^+_i under action pathways on RH, and the threshold $ES^+_{i,min}$, such that:

$$\Delta ES_{i,RH} = ES_i\left(u^{OCA}_{RH}(.)\right) - ES_{i,min} \qquad \text{equation S2}$$

The more positive this distance was from zero, the more secure the ES was for a given adaptation pathway, i.e. allowing to reduce the risks to cross $ES^+_{i,min}$ and not be viable. A greater and negative distance signifies dissatisfaction, i.e. non-viability.

We compared these distances pair-by-pair to analyze the relative effect of acting on i) tall hedgerows (TH) vs short hedgerows (SH) and ii) species-rich hedgerows (RH) versus species-poor (PH). For instance, based on the (i) comparison, the relative effect on acting on (i) was estimated this way:

$$\Delta ES_{RH-PH} = \Delta ES_{RH} - \Delta ES_{PH} = \left[ES\left(u^{OCA}_{RH}(.)\right) - ES_{i,min}\right] - \left[ES\left(u^{OCA}_{PH}(.)\right) - ES_{i,min}\right] \qquad \text{equation S3}$$

Assuming the resulting metric is the result of the same unit of action on every hedgerow type (i.e. $\Delta a = 1$), then eq. S3 becomes strictly equivalent to:

$$\left(\frac{\Delta ES_{RH-PH}}{\Delta u^{OCA}_{RH-PH}(.)} = \Delta ES_{RH-PH}\right) \qquad \text{equation S4}$$

Under this assumption, eq. S4 (and thus eq.S3) represents a retrospective sensitivity analysis, where the numerator is the cause and the denominator the effect. It is a way to study causality and understand retrospectively every adaptation pathway and the entire DAPP maps. It particularly measures how much ES security can be gained for any given adaptation pathway ***u(.)*** (from the satisfactory baseline defined in eq. S2, S3), that operationally consisted in increasing RH over PH. So when $\Delta ES_{RH-PH} > 0$, it means that an adaptive governance pathway ***u(.)***, consisting in increasing RH over PH, has contributed to secure more ES_i viability*;* and that the greater this value, the greater its security. Conversely, when $\Delta ES_{RH-PH} < 0$, then it tells that ***u(.)*** consisting in increasing PH over RH actually contributed to secure more ES_i viability.

Eq. S4 was estimated to compare the effect between RH and PH (figure 10) and between TH and SH (figure S10). These comparisons were summarized by i) a global statistic estimated for the entire set of pathways (viable and non-viable) and ii) for the viable set of pathways only.

References

- Barros, V.R., Dokken, D.J., Mach, K.J., Mastrandrea, M.D., Bilir, T.E., Chatterjee, M., Ebi, K.L., Estrada, Y.O., Genova, R.C., Girma, B. and Kissel, E.S., 2017. Climate Change 2014: Impacts, Adaptation, and Vulnerability. Part A: Global and Sectoral Aspects. Contribution of Working Group II to the Fifth Assessment Report of the Intergovernmental Panel on Climate Change, CB Field and others (eds.), Cambridge, Cambridge University Press.
