# Supplementary material for: Integrating adaptation pathways and Ostrom’s framework for sustainable governance of social-ecological systems in a changing world
Source: PeerJ. 2025 Feb 24;13:e18938. doi: 10.7717/peerj.18938 (PMC11867035; doi:10.7717/peerj.18938)
Supplement: Supplemental Information 3 — S3.a. The orthophotographies with the distribution of hedgerows (orange color) for the two sites (rural and peri-urban) and four years (1946, 1989, 2009, 2019). QGIS was used to generate these pictures. S3.b Spreadsheet Summary of GIS Information for Hedgerows Across the peri-urban study site (city of Veyre Monton). The spreadsheet provides a comprehensive summary for each hedgerow located within the study site of (1) Geographic Information System (GIS) attributes, (2) the physical attributes,and (3) the ecological attributes from the survey conducted in summer 2021. The GIS attributes are GPS coordinates, identification (ID), and hedgerow typology for the years 1989, 2000, and 2019. The physical attributes of every hedgerow encompass various features including size, height, length, adjacent habitats, presence of embankments, stone walls, ditches, and number of plant layers. Ecological attributes encompass a range of factors such as the number of plant species, plant pollinating species, fruit species, living wood biomass, snag number, and deadwood biomass. These metrics offer crucial information about the biodiversity and some ecosystem services characterizing the hedgerows. The data compiled in this spreadsheet serves as a valuable resource for analyzing the ecological dynamics and conservation status of hedgerows in the study area, facilitating informed decision-making and management strategies for their preservation and enhancement. This information also served as the foundation for analyzing the annual probability of transition between various combinations of hedgerow types, which include hedgerows that were tall species-rich, short species-rich, tall species-poor, short species-poor, and none. It is noteworthy that the hedgerow data from the 1946 aerial photography was exclusively utilized to estimate hedgerow density. Due to the limited visual quality, determining the exact hedgerow type was not feasible. However, this data significantly contributes to unders [file peerj-13-18938-s003.zip › Supplementary.S3.Pictures.GIS.Land.Use.Hedgerows.Rural.and.PeriUrban.1946.1989.2000.2019.pdf]

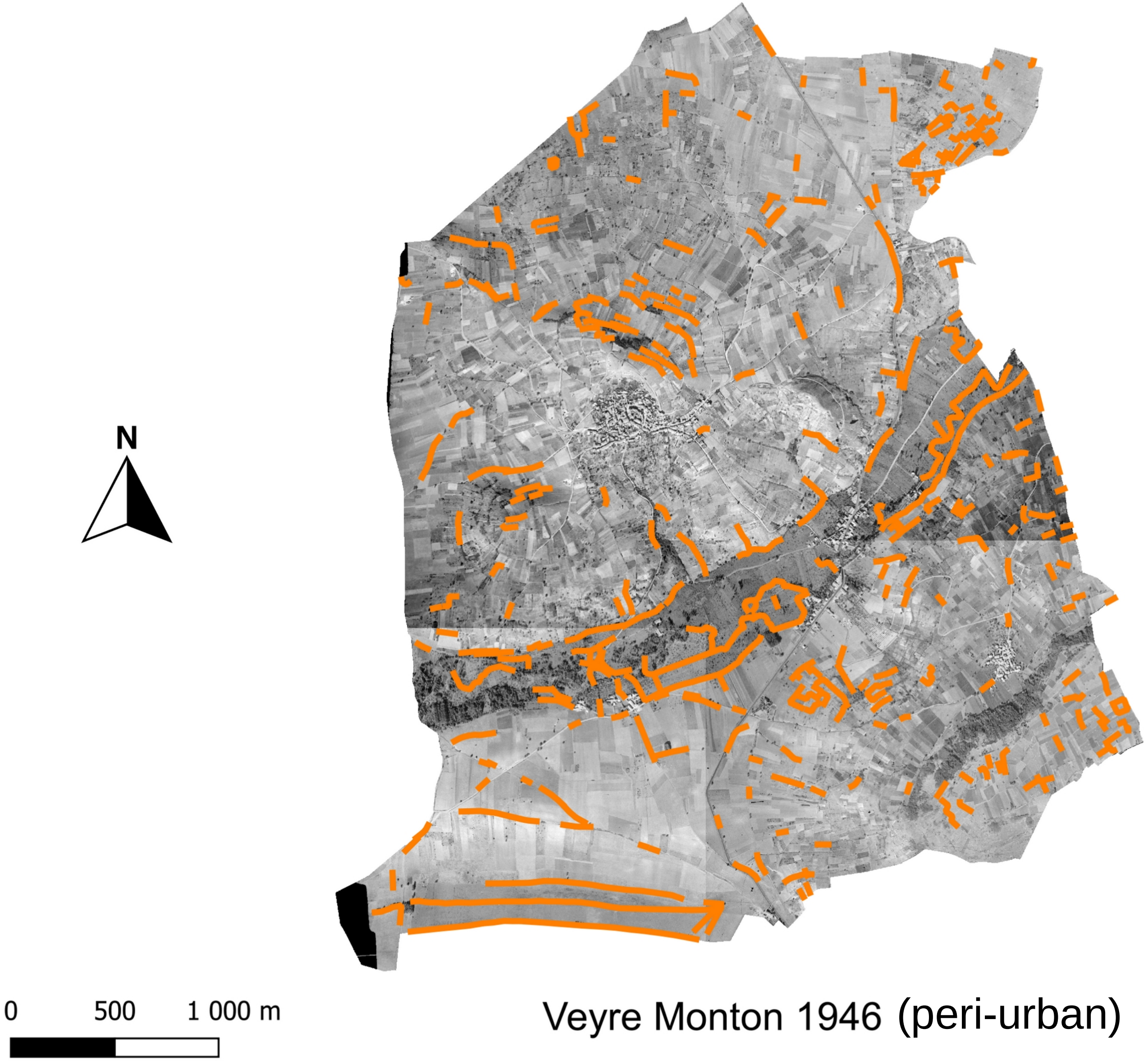

Veyre Monton 1946 (peri-urban)

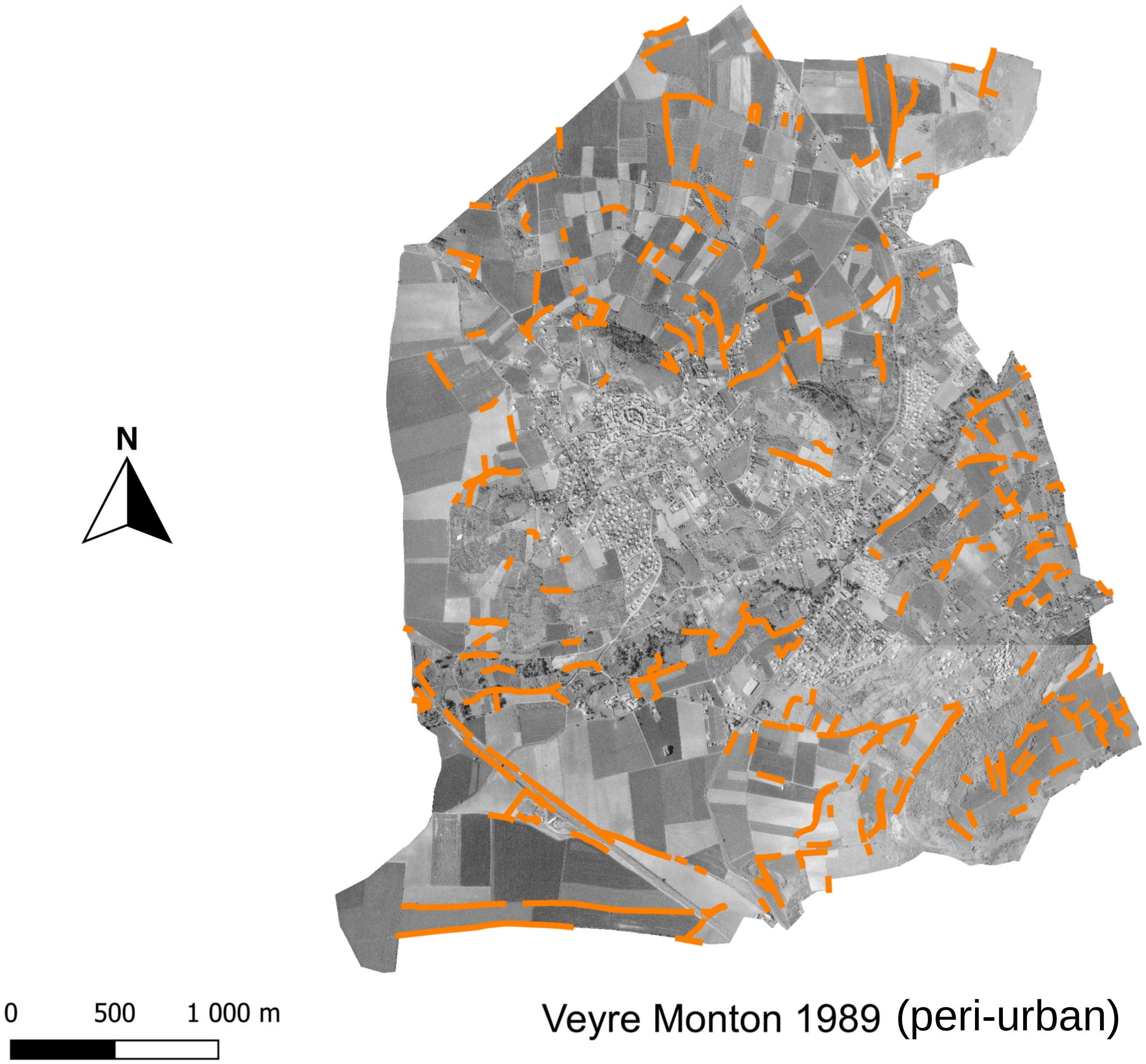

Veyre Monton 1989 (peri-urban)

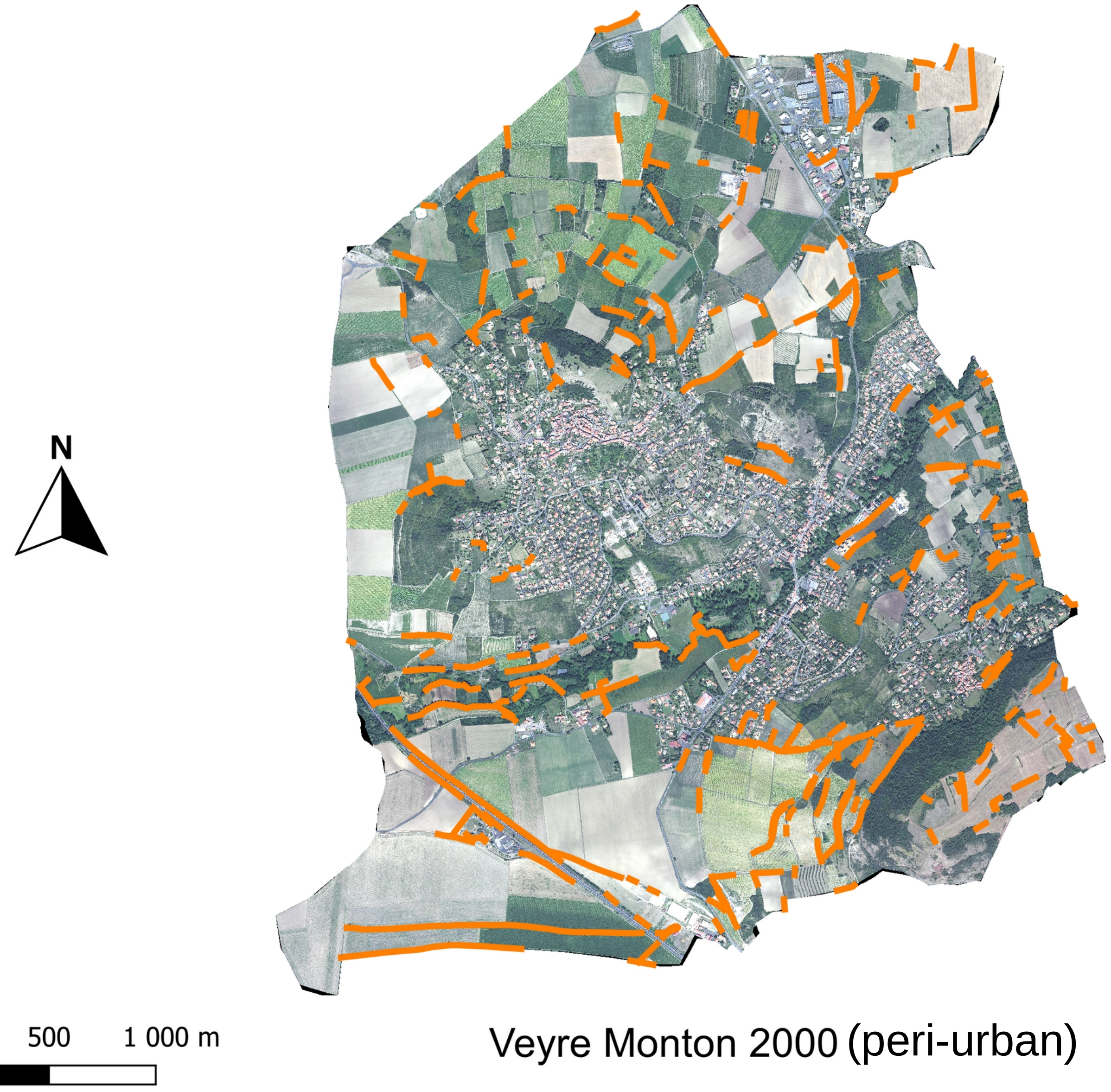

Veyre Monton 2000 (peri-urban)

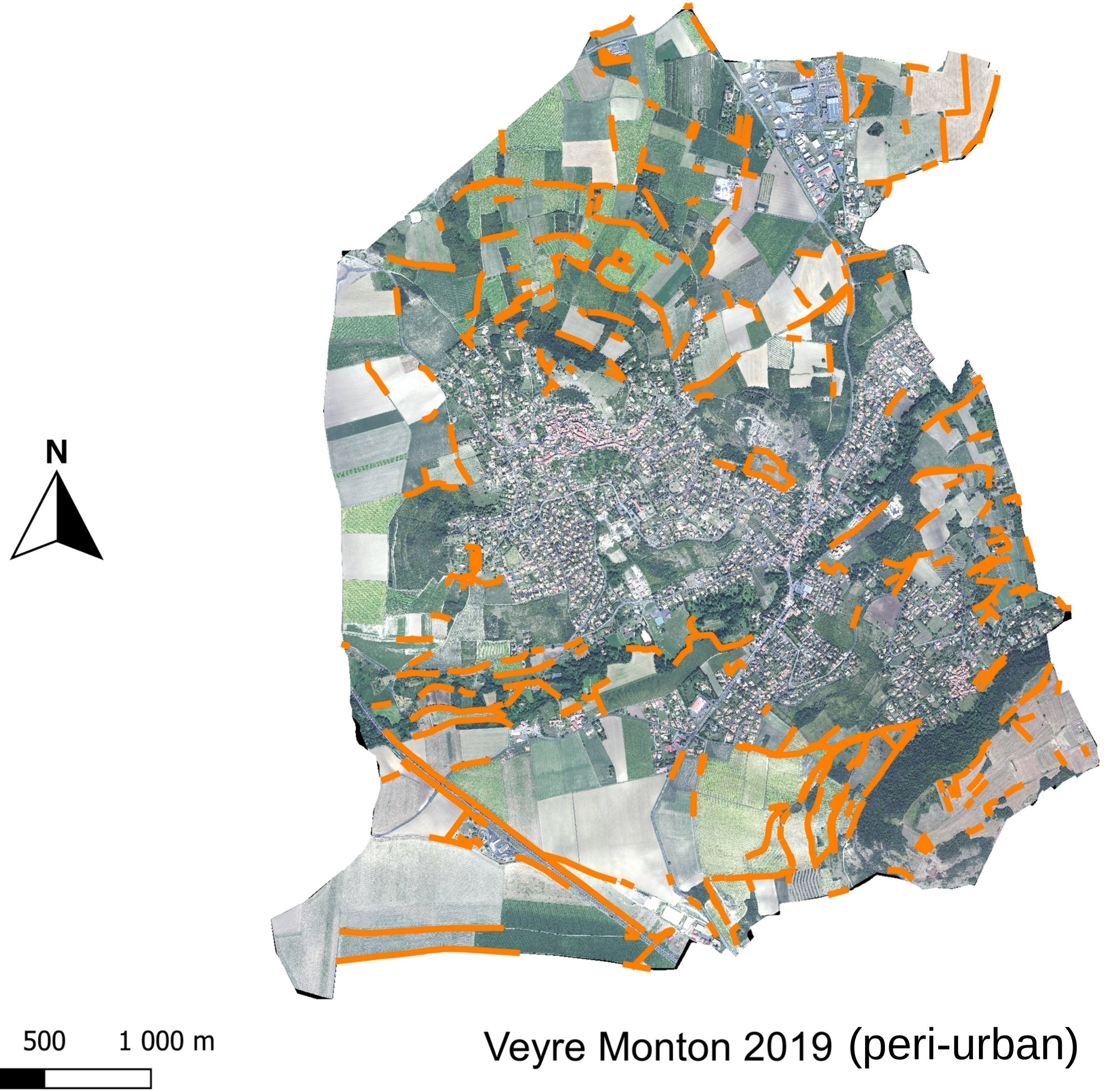

Veyre Monton 2019 (peri-urban)

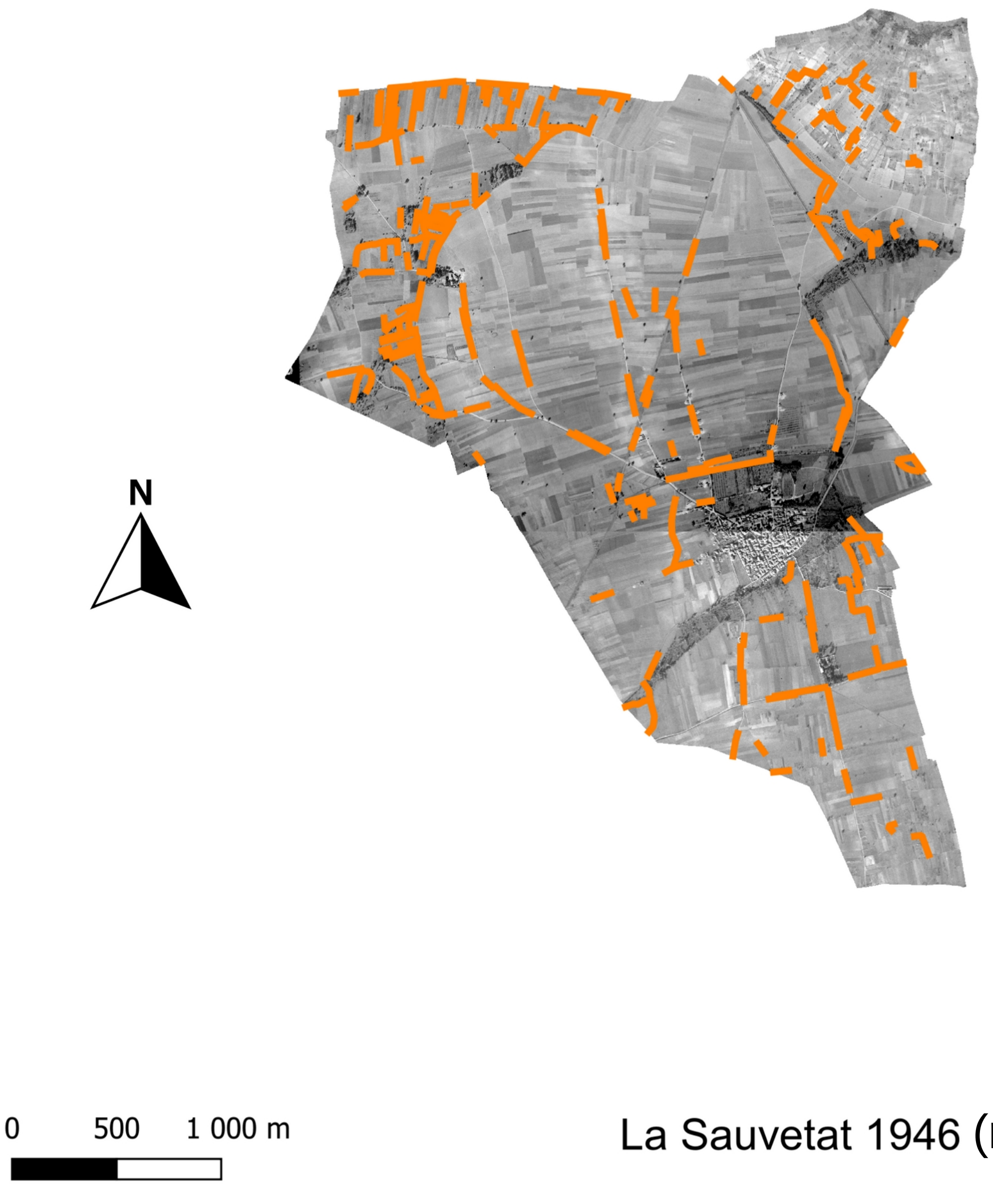

La Sauvetat 1946 (rural)

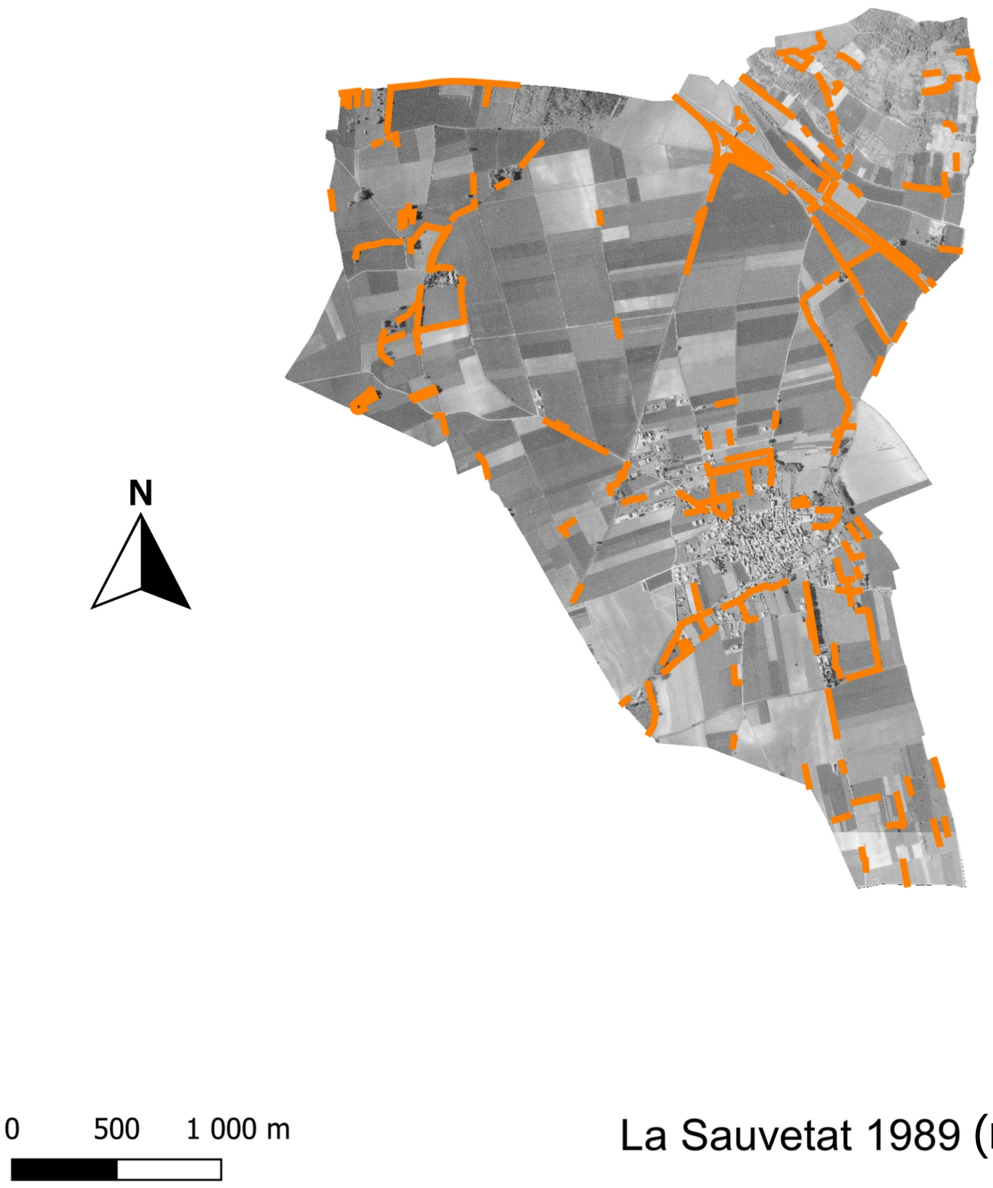

La Sauvetat 1989 (rural)

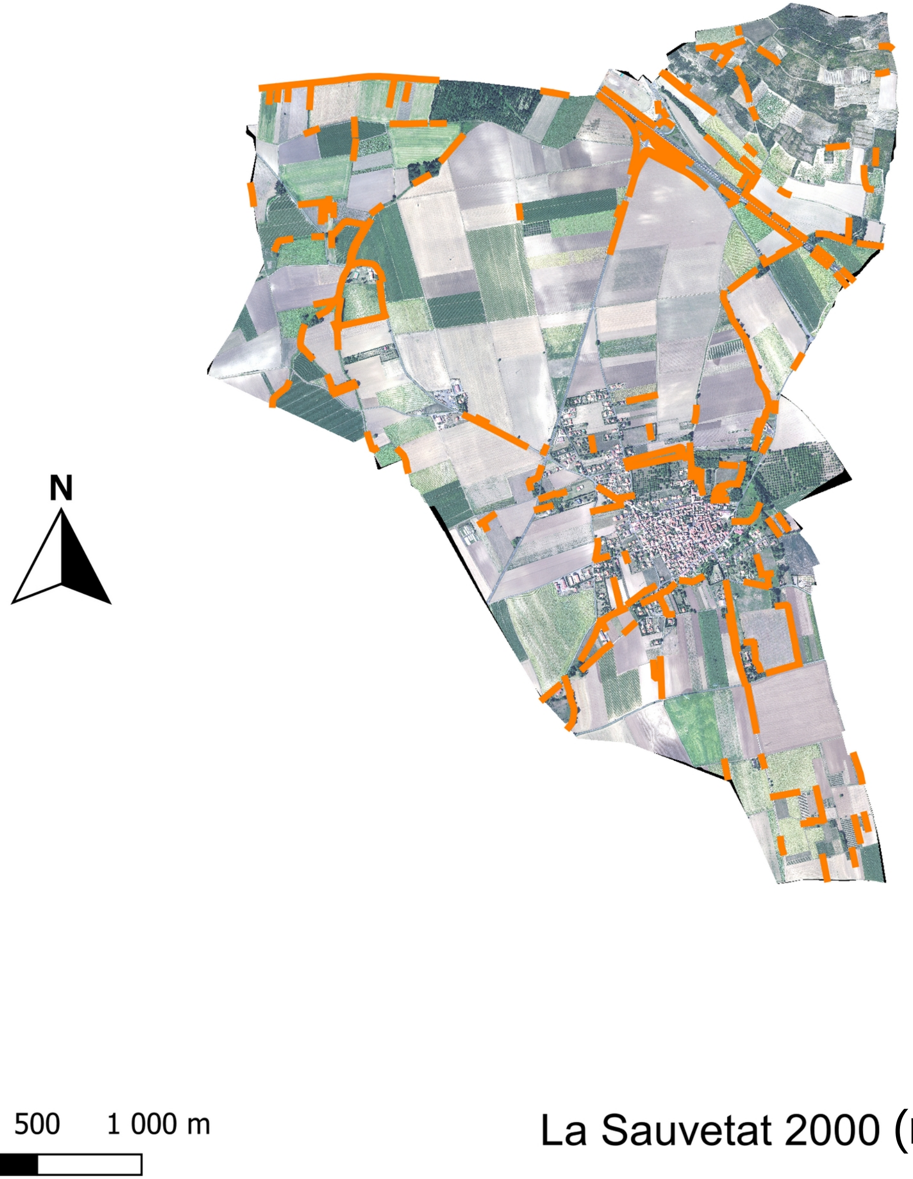

La Sauvetat 2000 (rural)

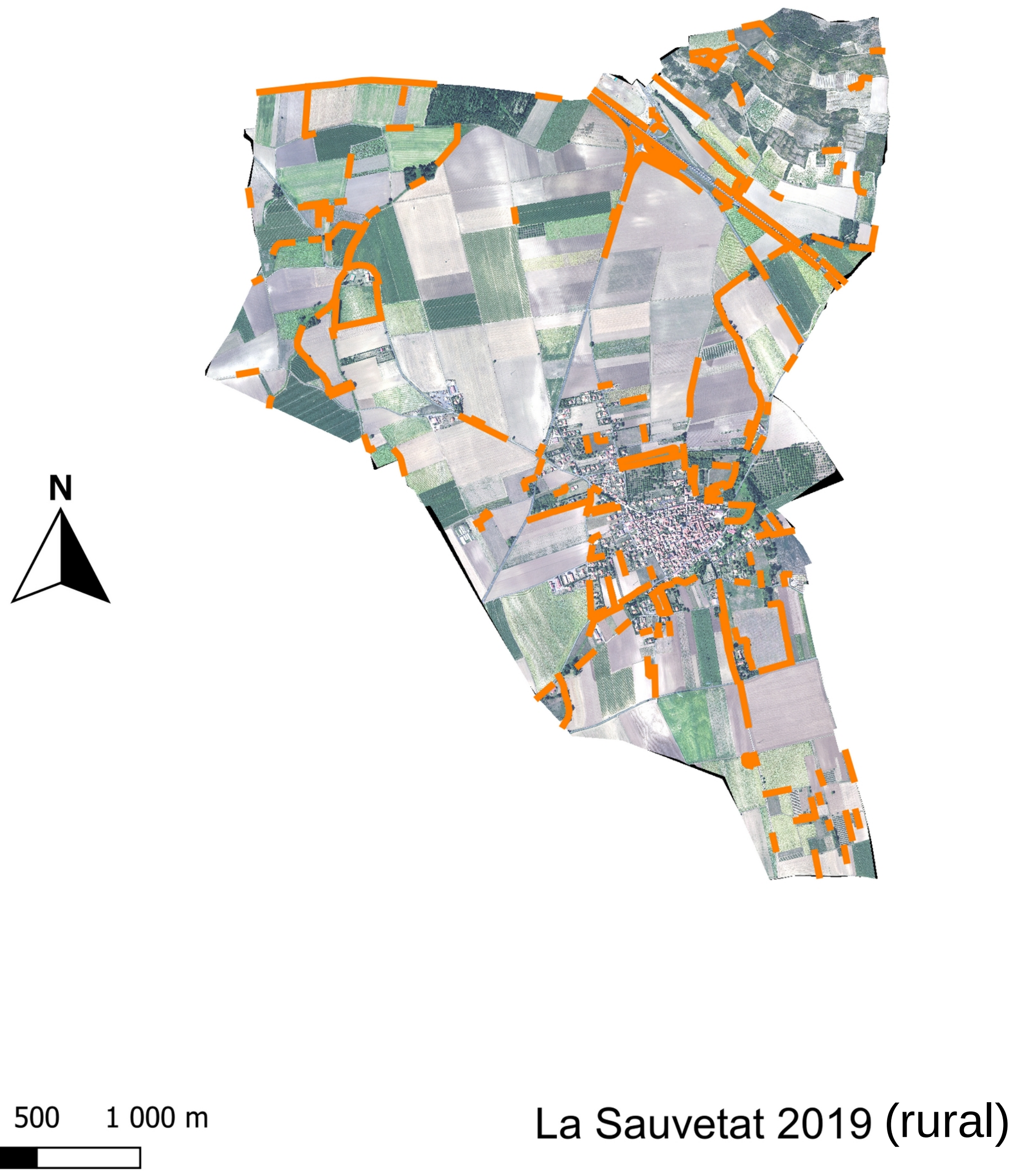
N
0
500
1 000 m
La Sauvetat 2019 (rural)
