# Supplementary material for: Integrating adaptation pathways and Ostrom’s framework for sustainable governance of social-ecological systems in a changing world
Source: PeerJ. 2025 Feb 24;13:e18938. doi: 10.7717/peerj.18938 (PMC11867035; doi:10.7717/peerj.18938)
Supplement: Supplemental Information 4 [file peerj-13-18938-s004.zip › PACSEN-main/figures/figures_annexe/dyn_hedges_full_scen_3.pdf]

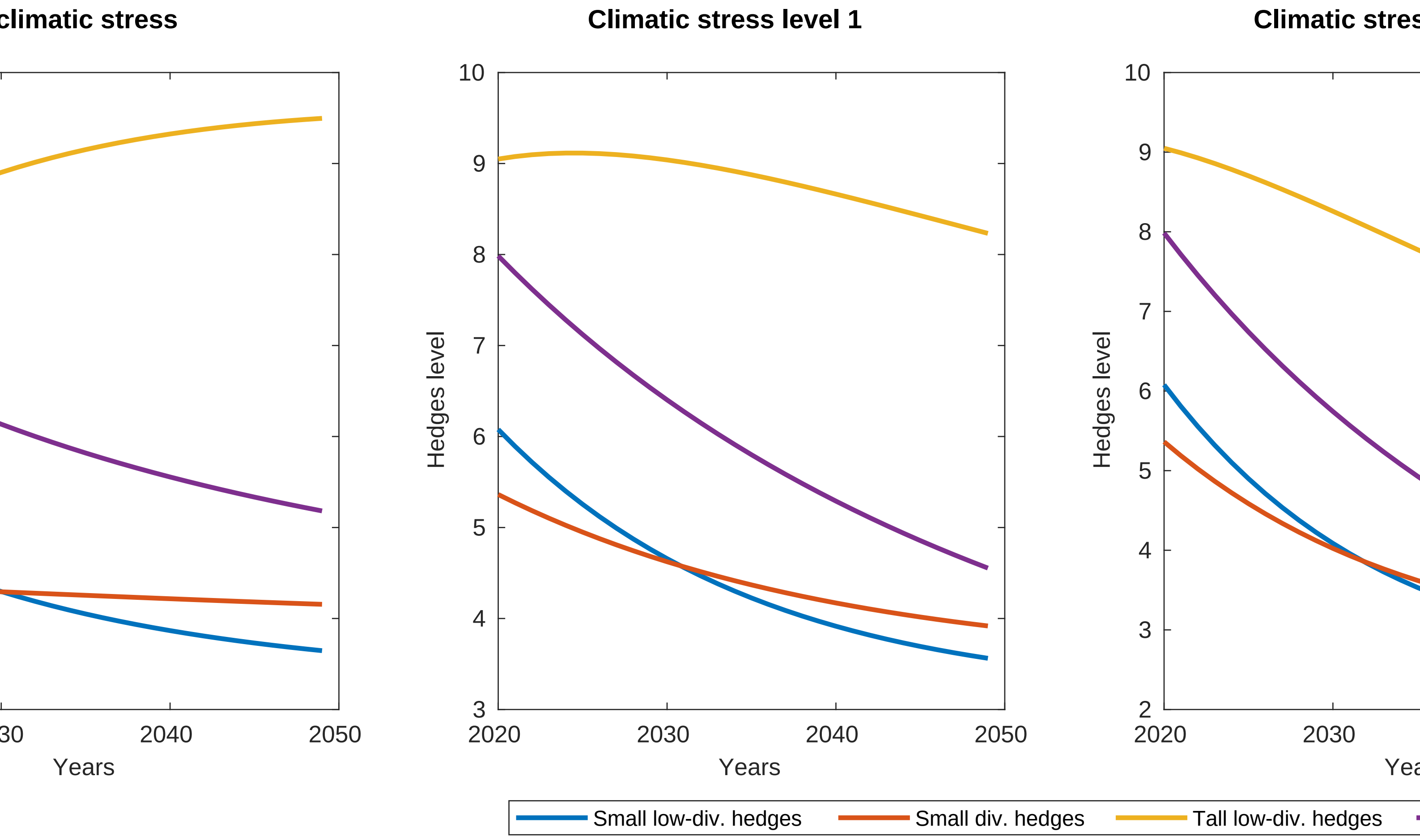
climatic stress
Climatic stress level 1
Climatic stres
Hedges level
Years
Yea
10
9
8
7
6
5
4
3
2
30
2020
2030
2040
2050
Small low-div. hedges
Small div. hedges
Tall low-div. hedges
