# Supplementary figures and images for: Integrating adaptation pathways and Ostrom’s framework for sustainable governance of social-ecological systems in a changing world
Source: PeerJ. 2025 Feb 24;13:e18938. doi: 10.7717/peerj.18938 (PMC11867035; doi:10.7717/peerj.18938)

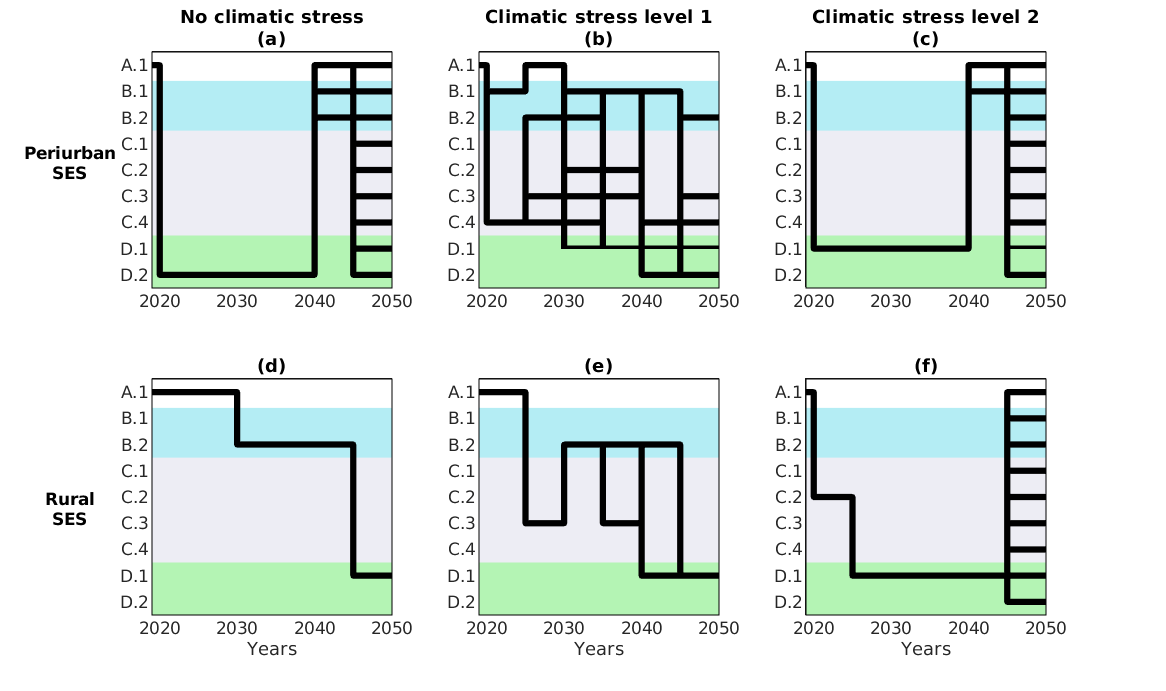

Supplement: Supplemental Information 4 [file peerj-13-18938-s004.zip › PACSEN-main/figures/best_sum_apm_action.png]

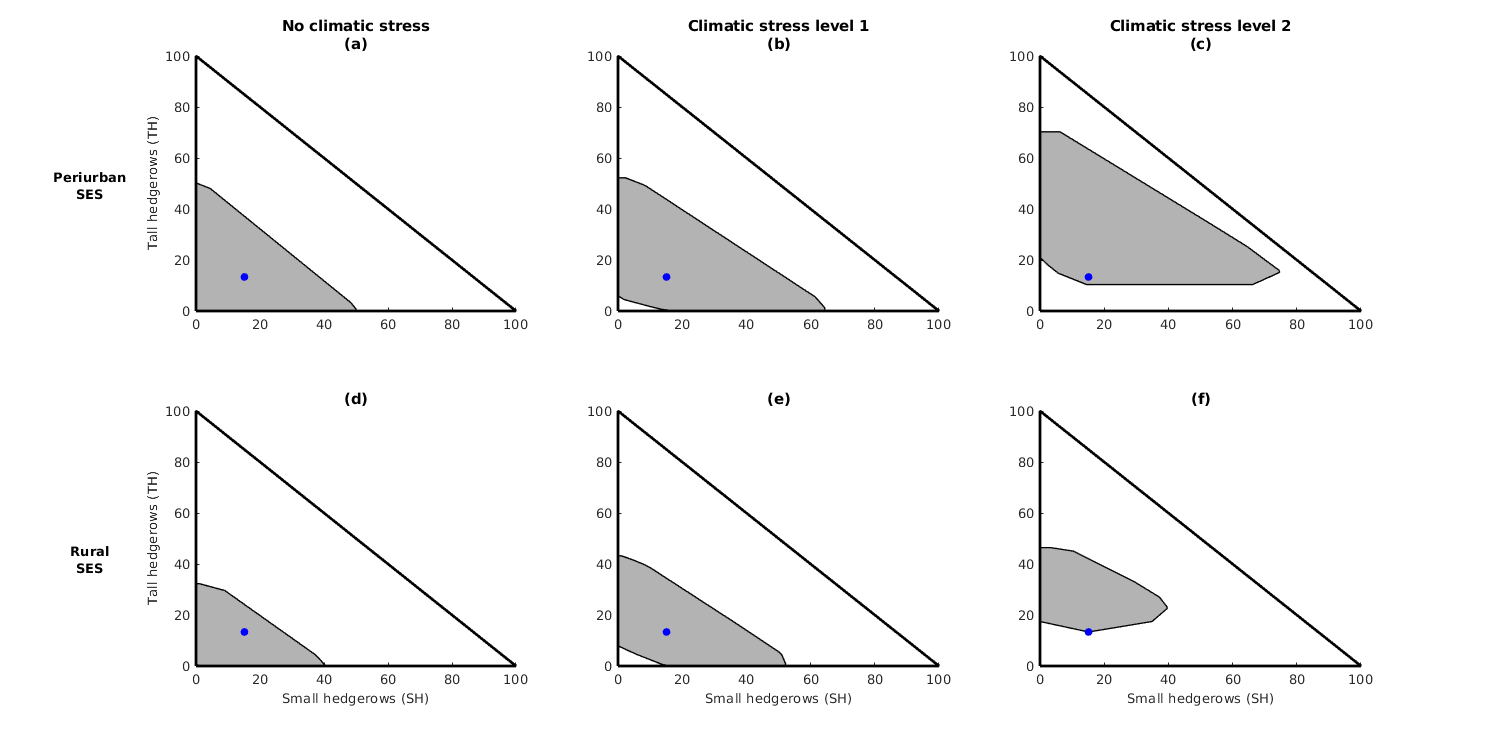

Supplement: Supplemental Information 4 [file peerj-13-18938-s004.zip › PACSEN-main/figures/fig9.png]

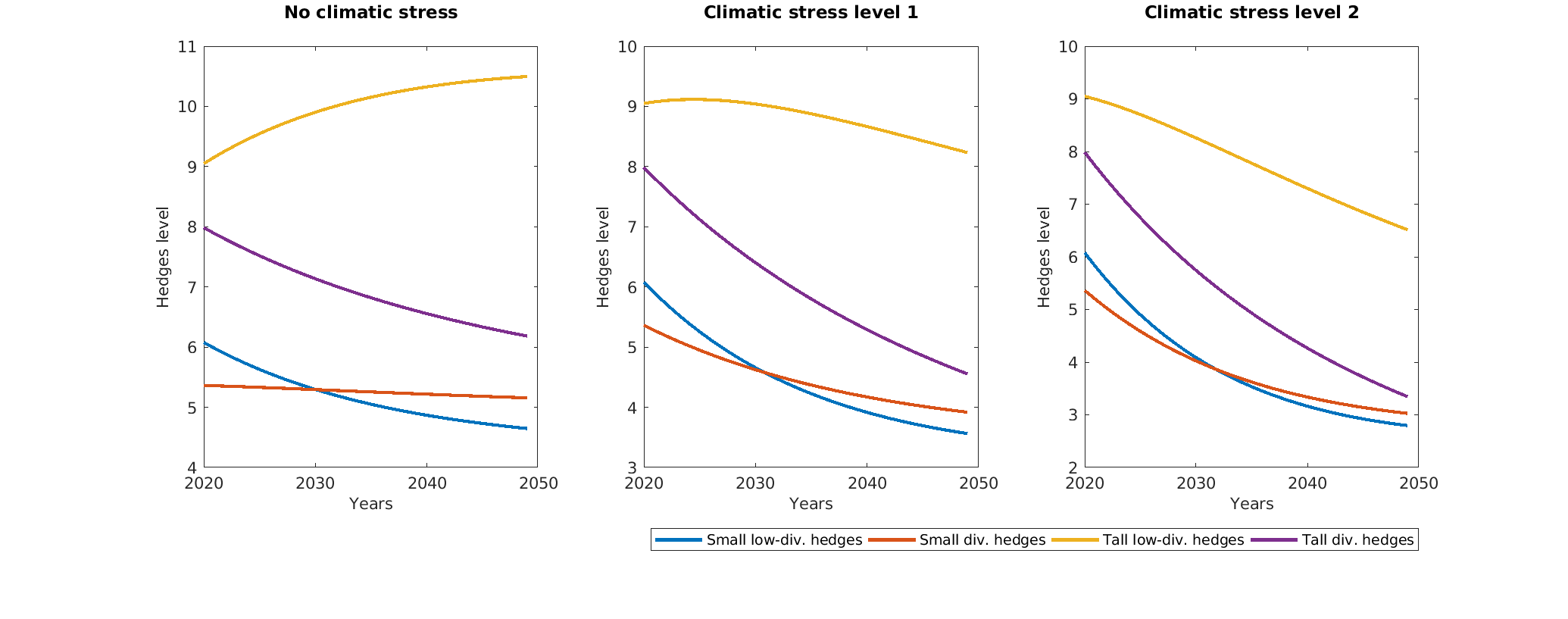

Supplement: Supplemental Information 4 [file peerj-13-18938-s004.zip › PACSEN-main/figures/figures_annexe/dyn_hedges_full_scen_1.png]

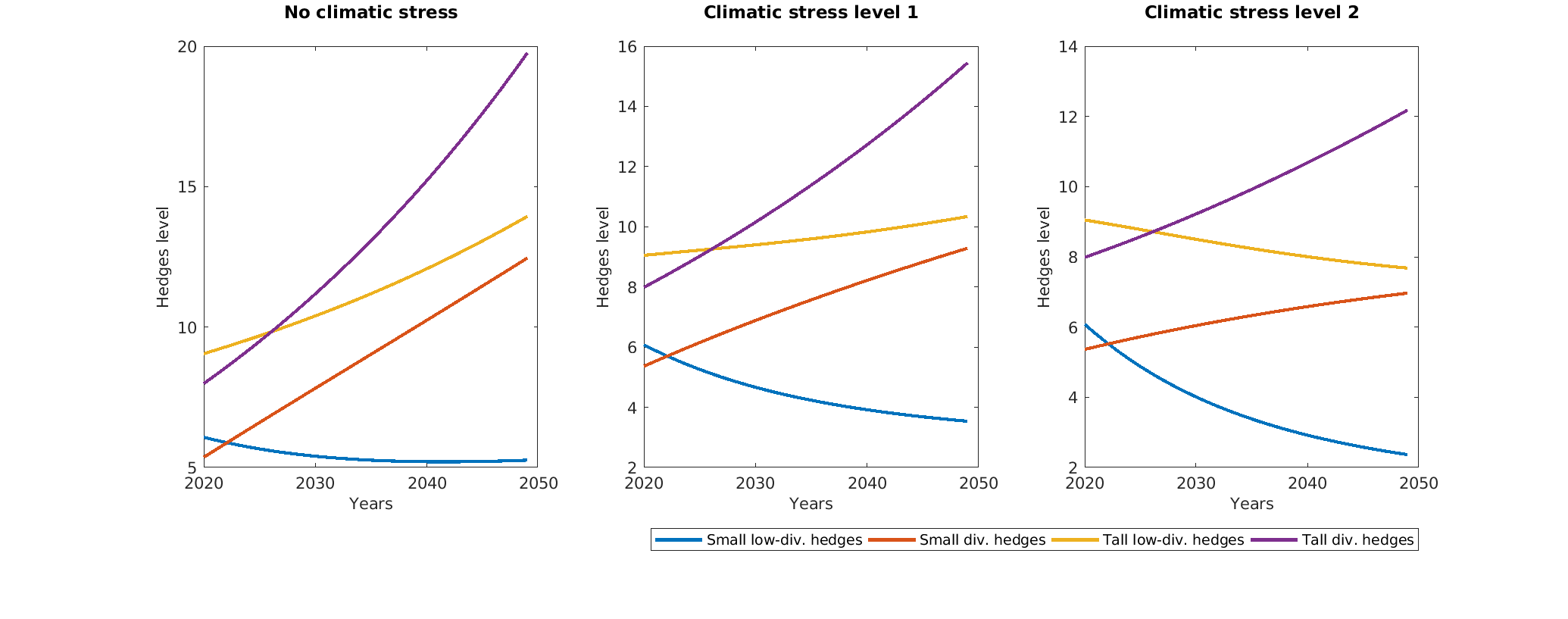

Supplement: Supplemental Information 4 [file peerj-13-18938-s004.zip › PACSEN-main/figures/figures_annexe/dyn_hedges_full_scen_2.png]

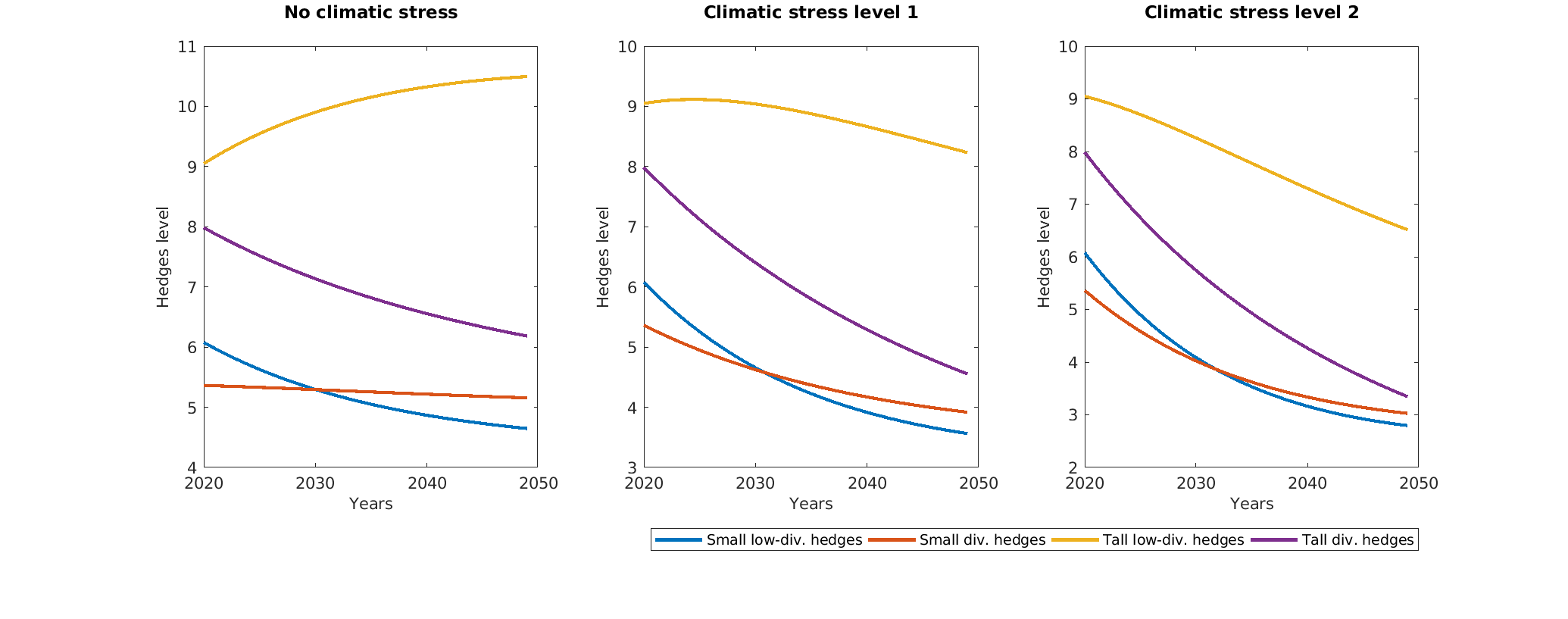

Supplement: Supplemental Information 4 [file peerj-13-18938-s004.zip › PACSEN-main/figures/figures_annexe/dyn_hedges_full_scen_3.png]

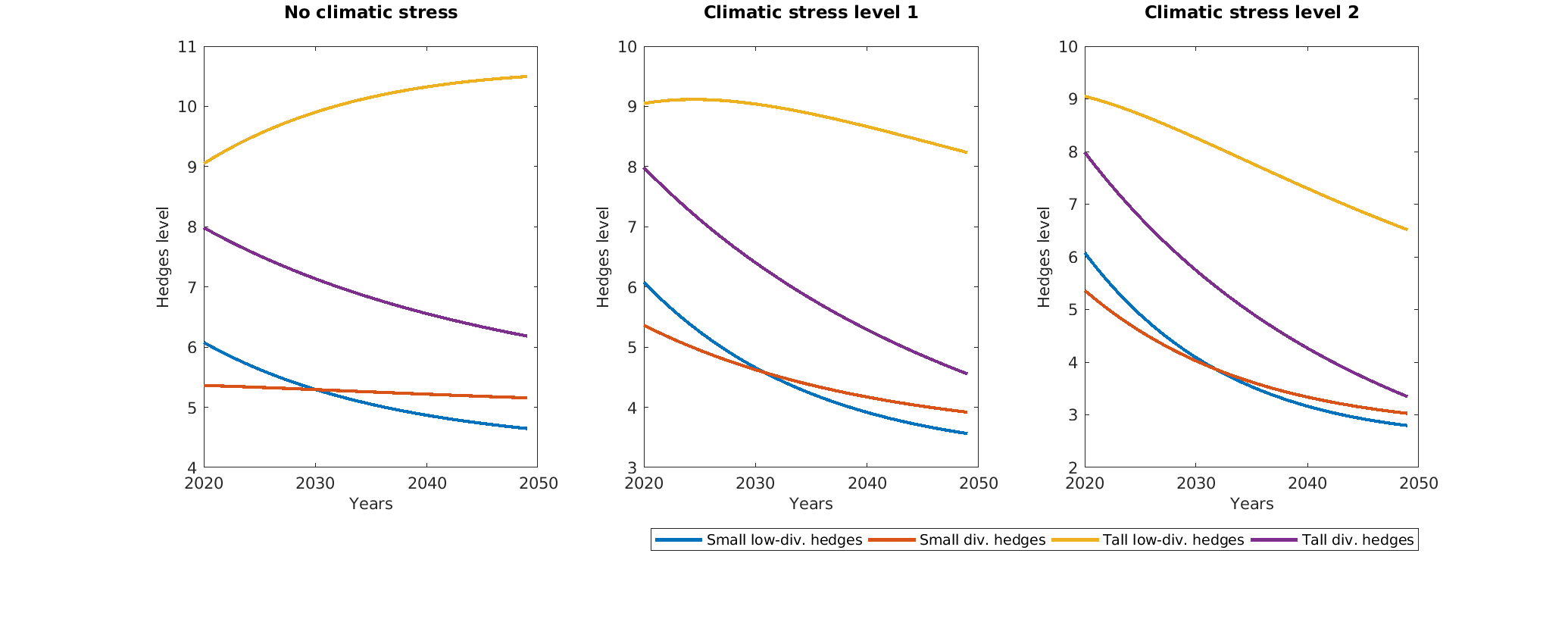

Supplement: Supplemental Information 4 [file peerj-13-18938-s004.zip › PACSEN-main/figures/figures_annexe/dyn_hedges_full_scen_4.png]

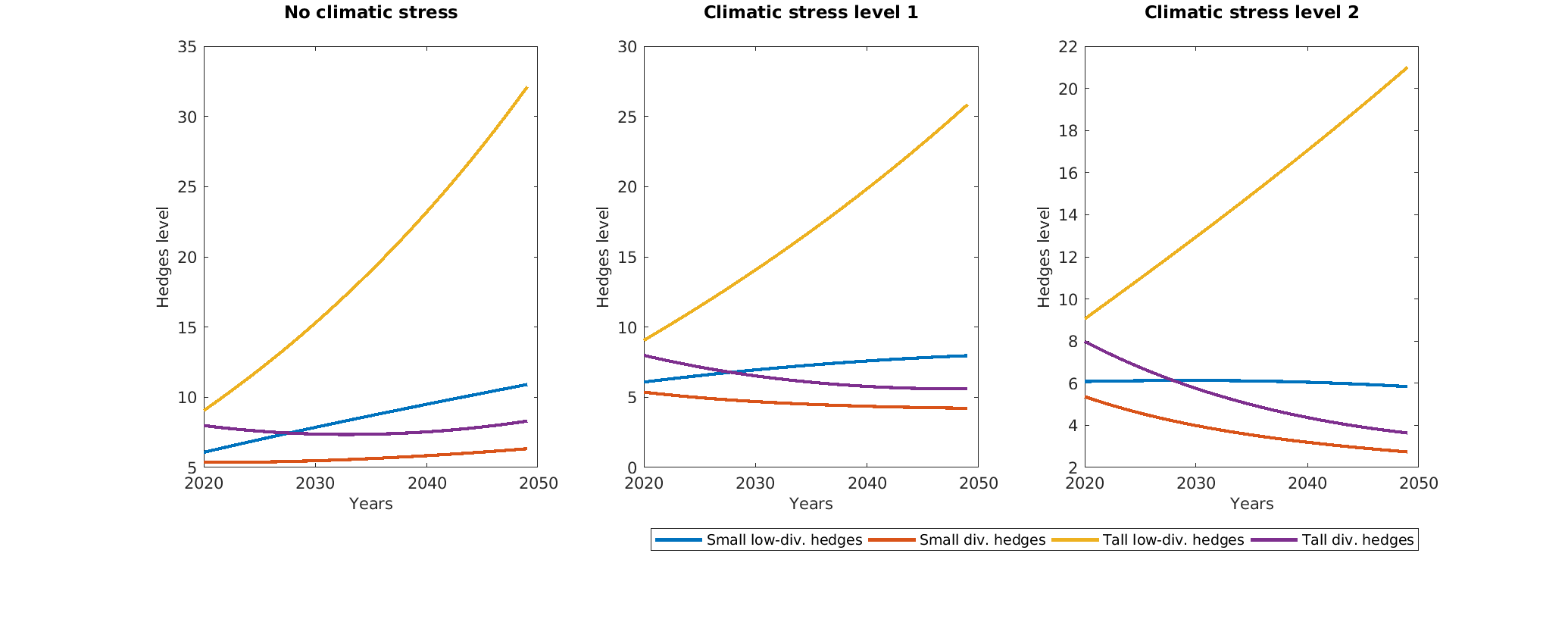

Supplement: Supplemental Information 4 [file peerj-13-18938-s004.zip › PACSEN-main/figures/figures_annexe/dyn_hedges_full_scen_5.png]

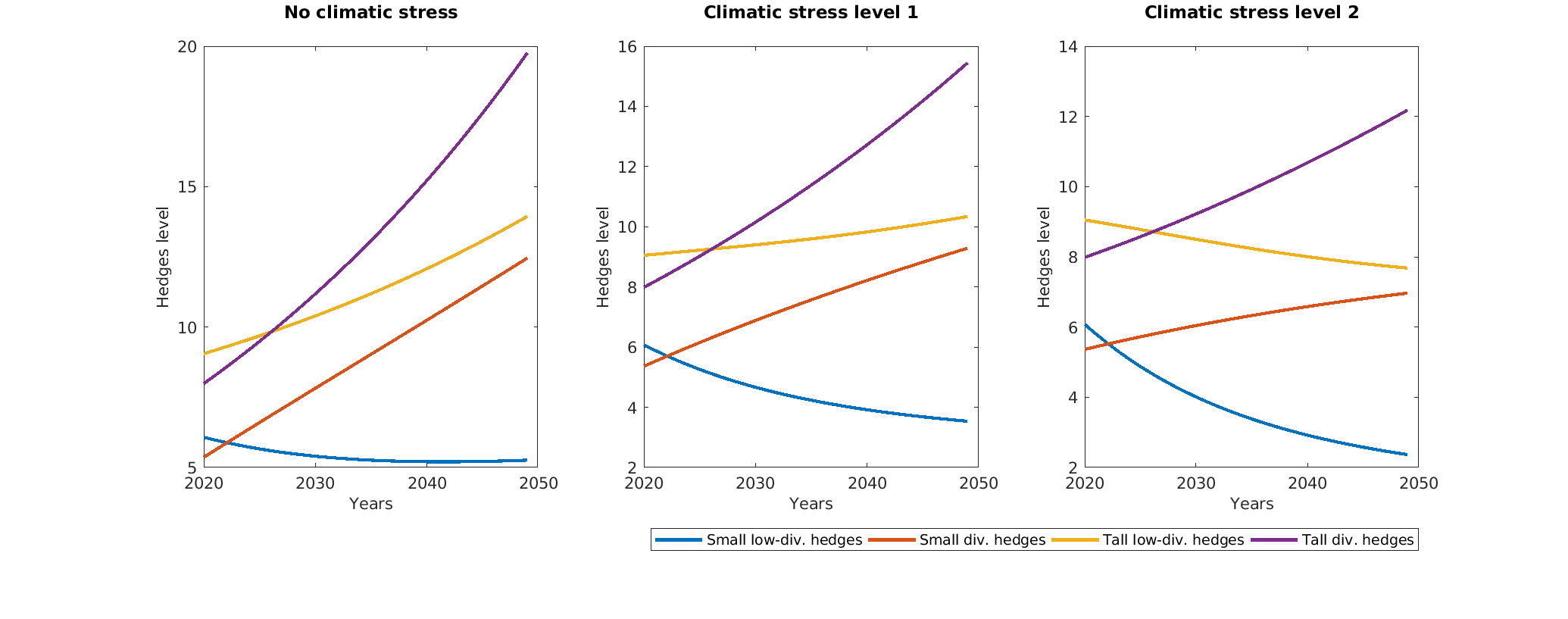

Supplement: Supplemental Information 4 [file peerj-13-18938-s004.zip › PACSEN-main/figures/figures_annexe/dyn_hedges_full_scen_6.png]

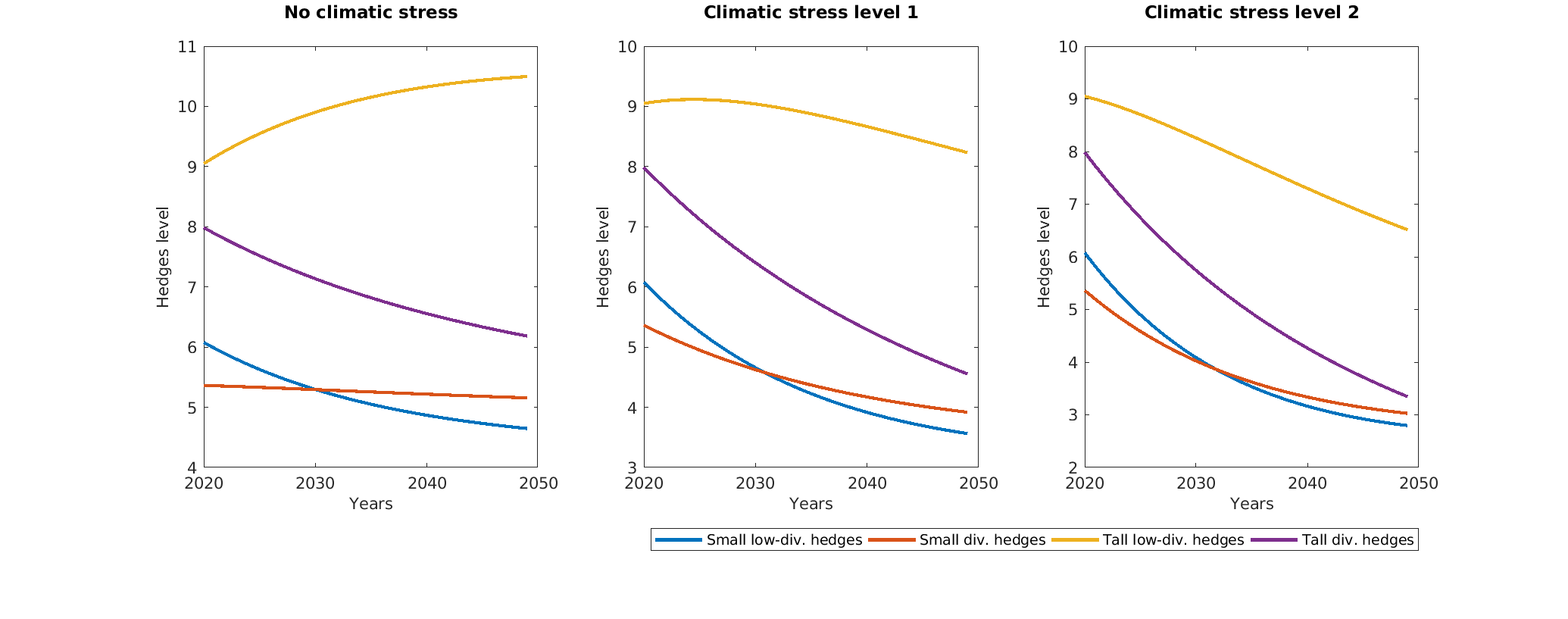

Supplement: Supplemental Information 4 [file peerj-13-18938-s004.zip › PACSEN-main/figures/figures_annexe/dyn_hedges_full_scen_7.png]

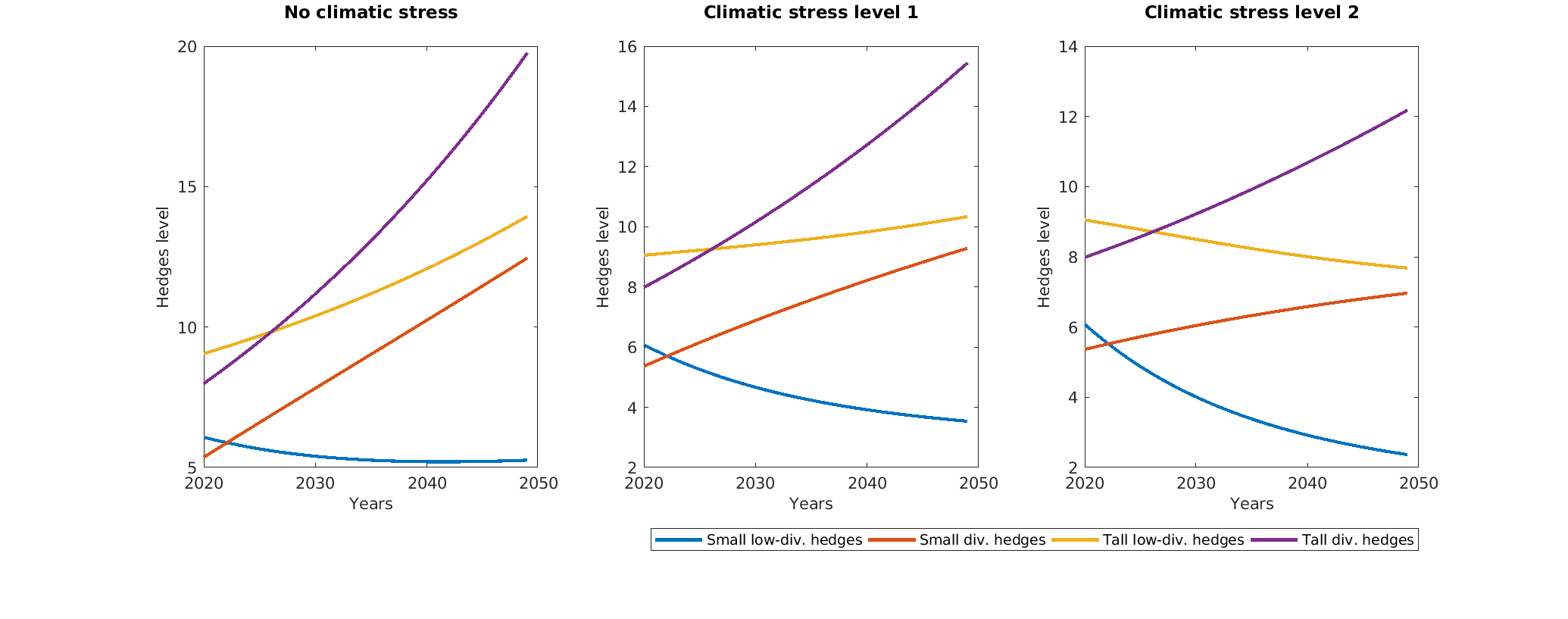

Supplement: Supplemental Information 4 [file peerj-13-18938-s004.zip › PACSEN-main/figures/figures_annexe/dyn_hedges_full_scen_8.png]

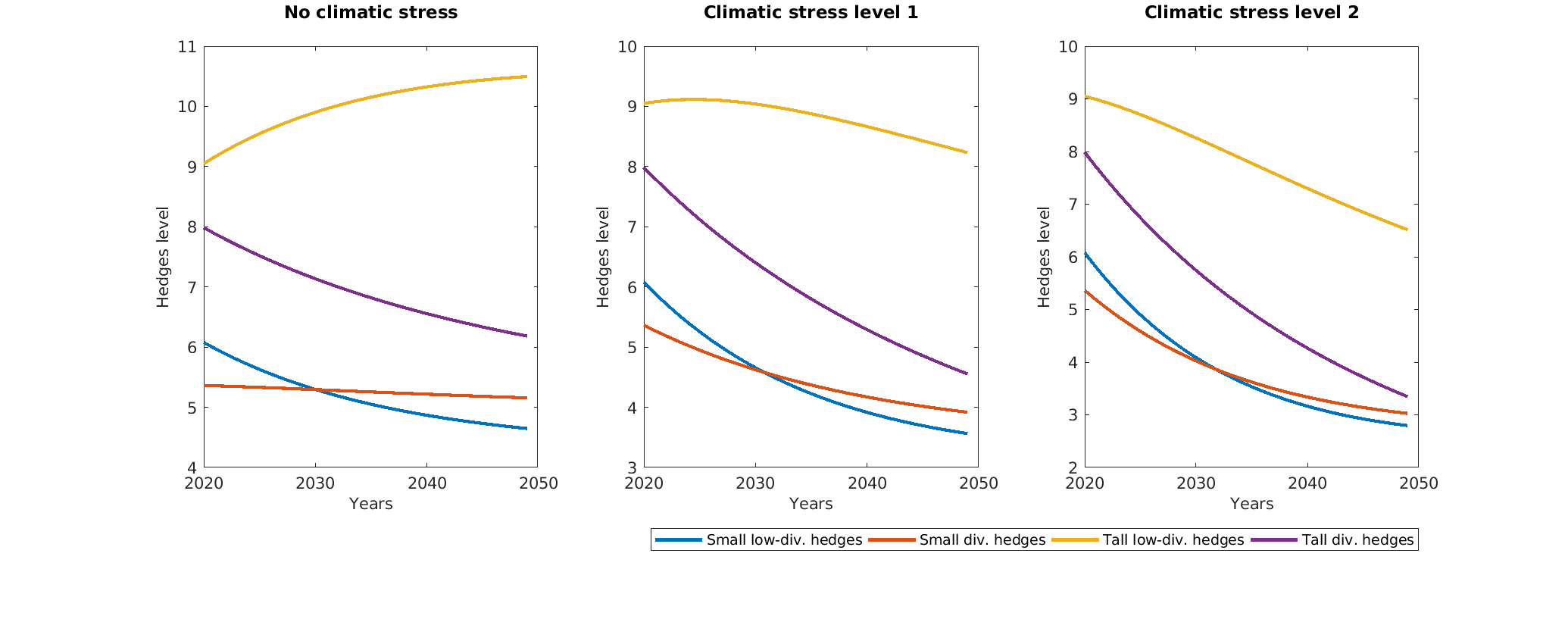

Supplement: Supplemental Information 4 [file peerj-13-18938-s004.zip › PACSEN-main/figures/figures_annexe/dyn_hedges_full_scen_9.png]

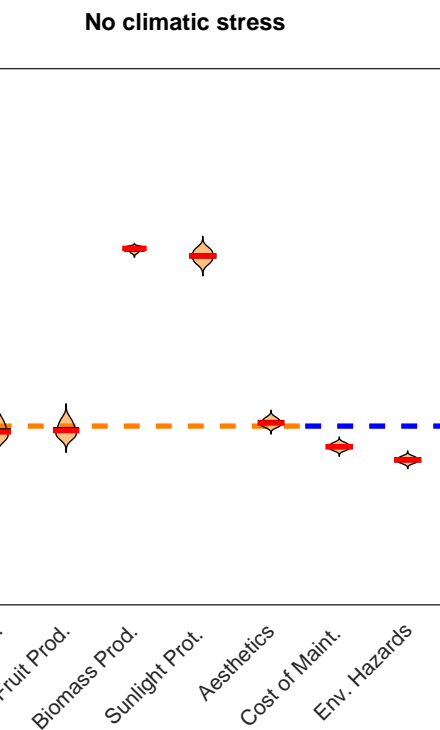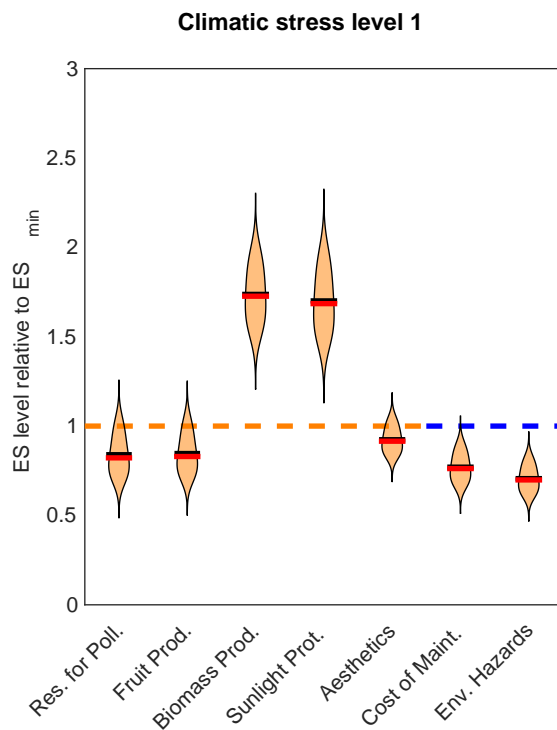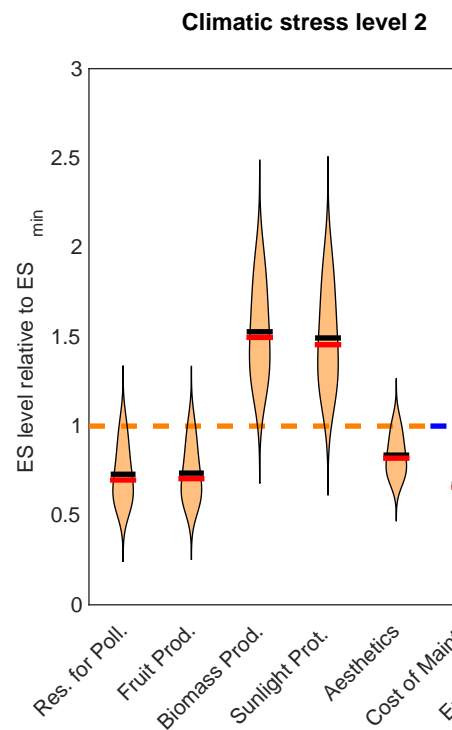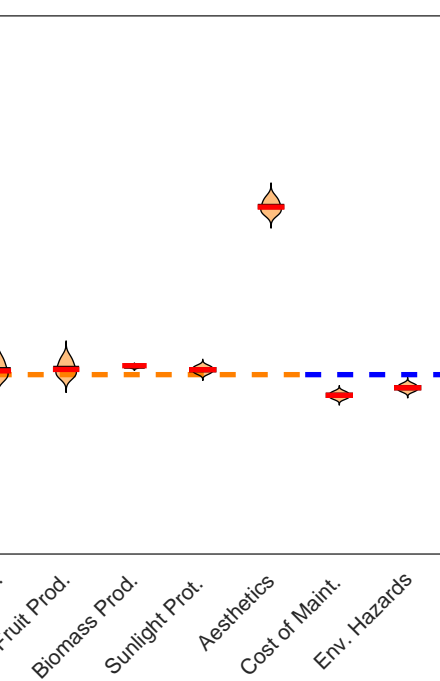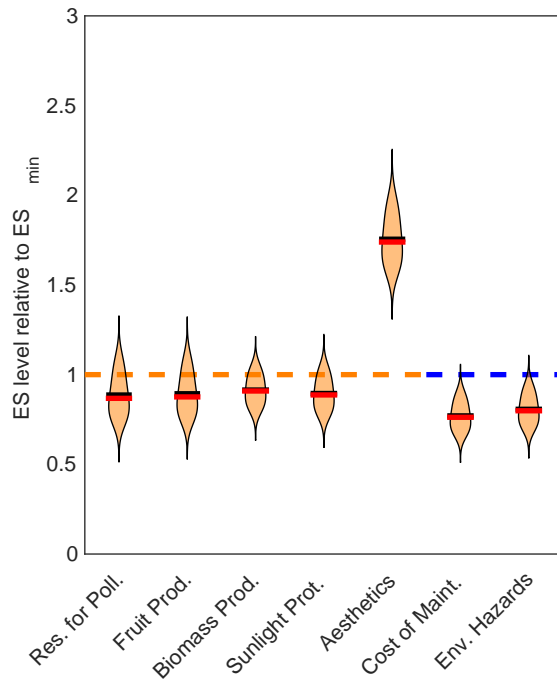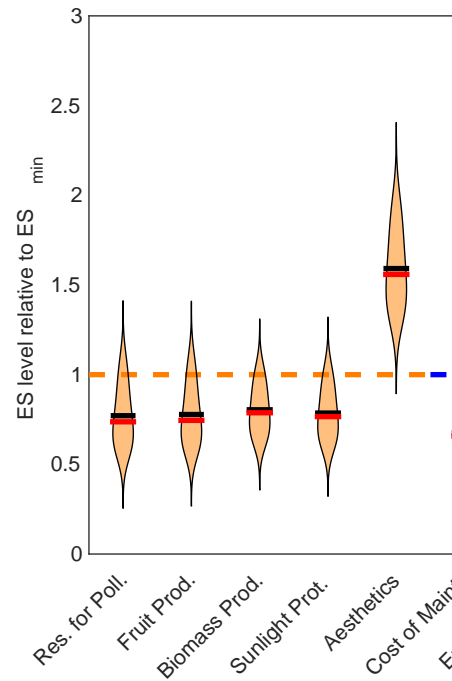

Supplement: Supplemental Information 4 [file peerj-13-18938-s004.zip › PACSEN-main/figures/figures_annexe/violin_full_scen_1.pdf]

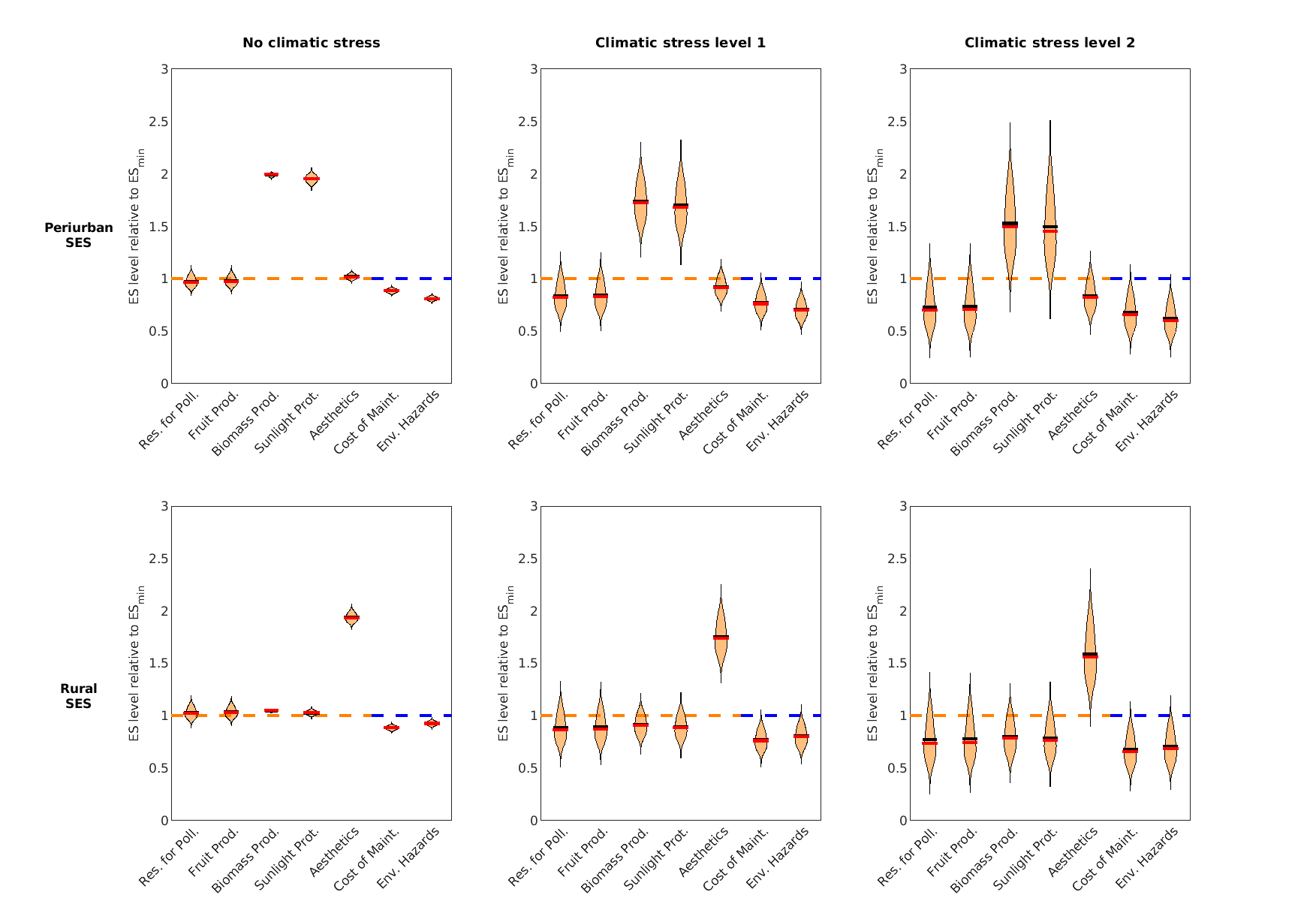

Supplement: Supplemental Information 4 [file peerj-13-18938-s004.zip › PACSEN-main/figures/figures_annexe/violin_full_scen_1.png]

No climatic stress

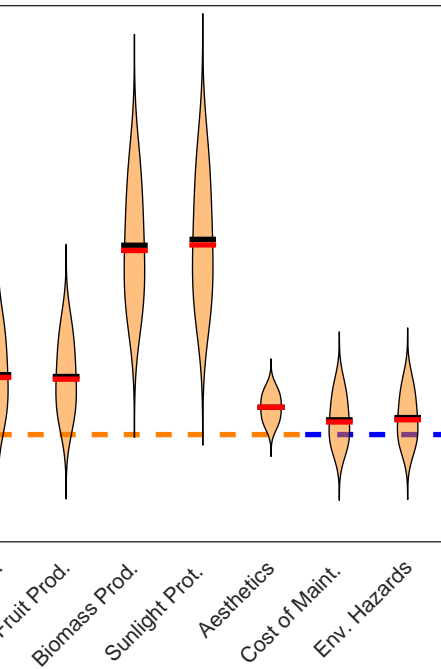

Climatic stress level 1

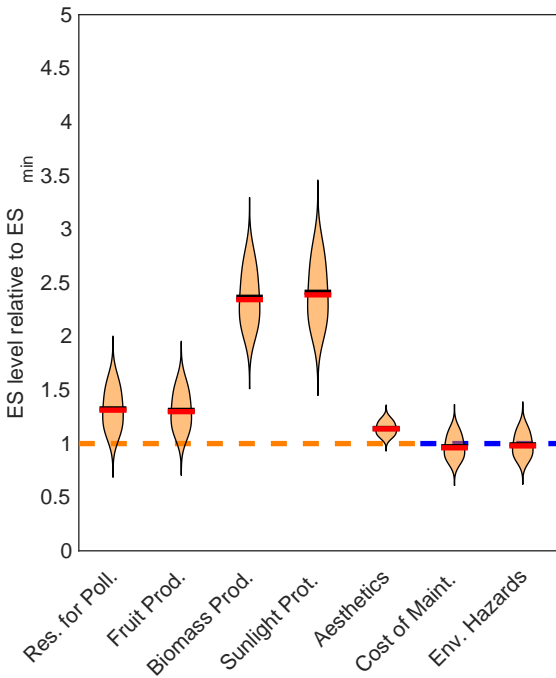

Climatic stress level 2

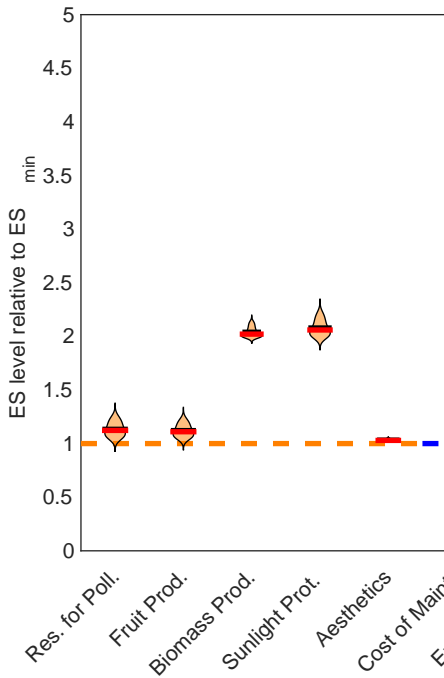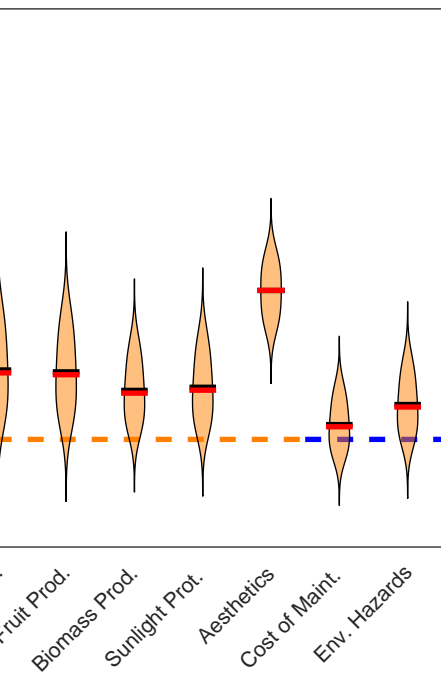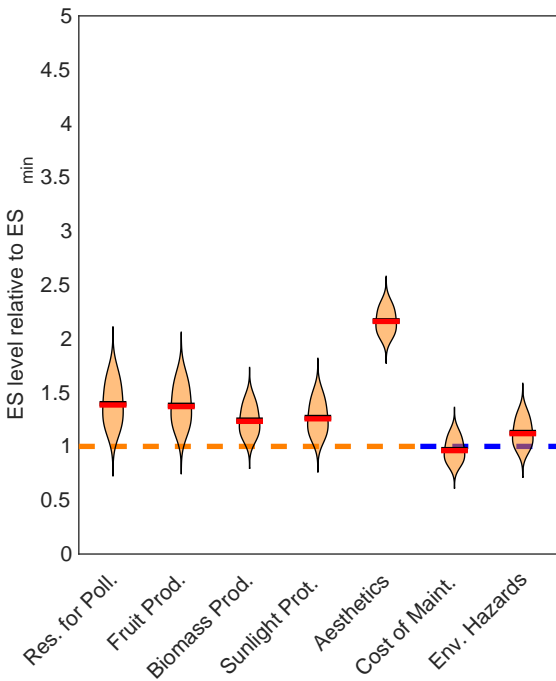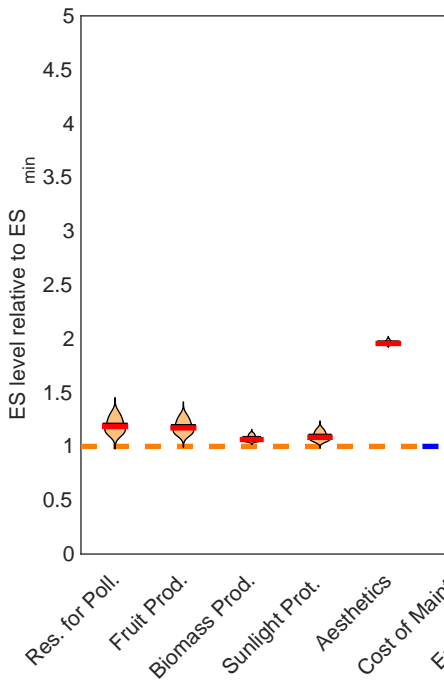

Supplement: Supplemental Information 4 [file peerj-13-18938-s004.zip › PACSEN-main/figures/figures_annexe/violin_full_scen_2.pdf]

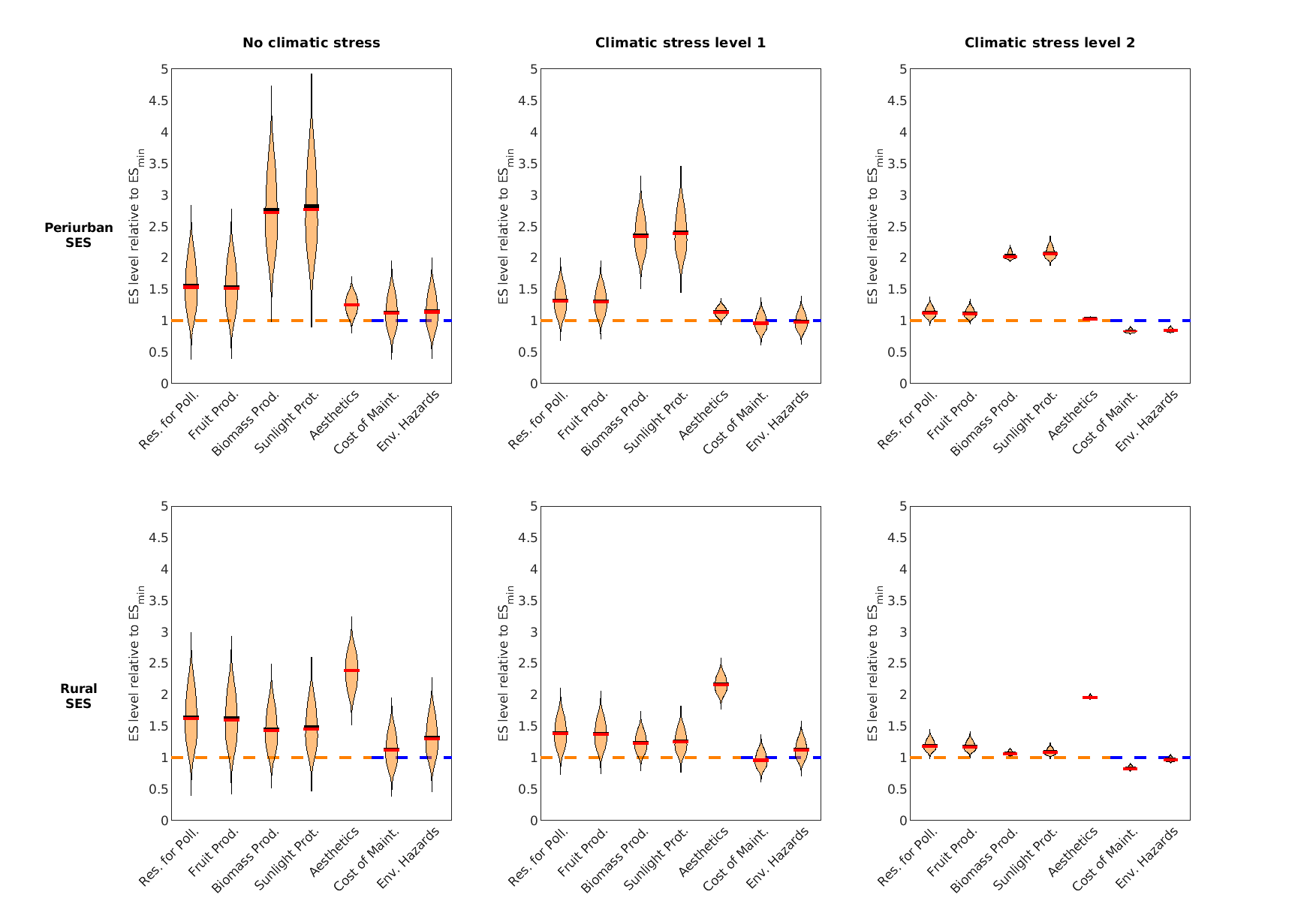

Supplement: Supplemental Information 4 [file peerj-13-18938-s004.zip › PACSEN-main/figures/figures_annexe/violin_full_scen_2.png]

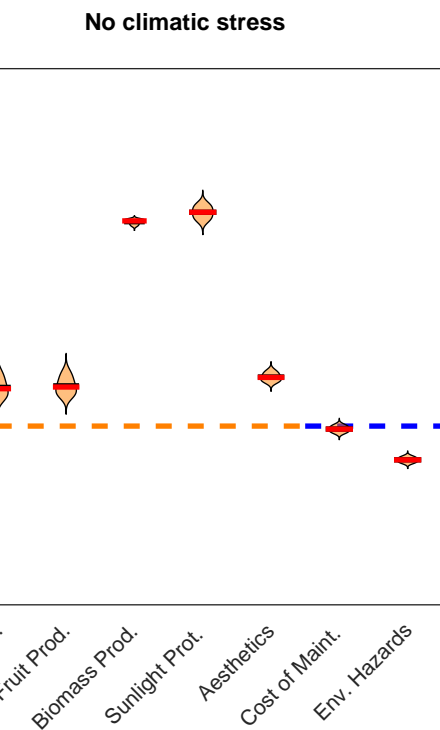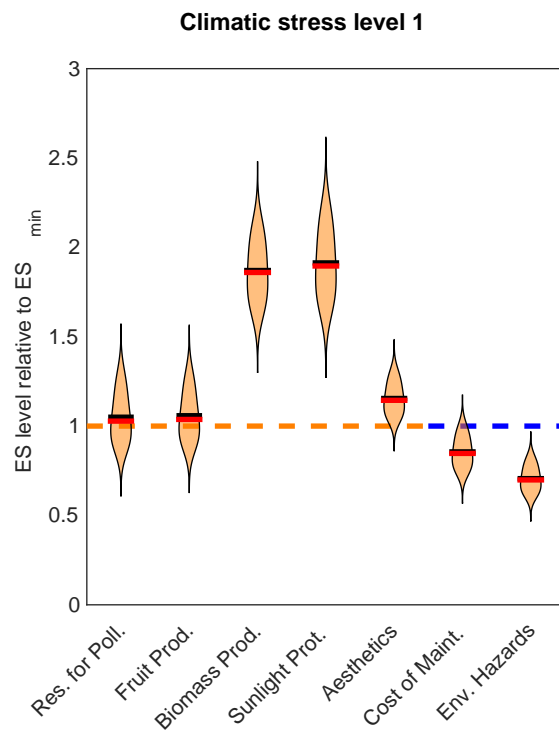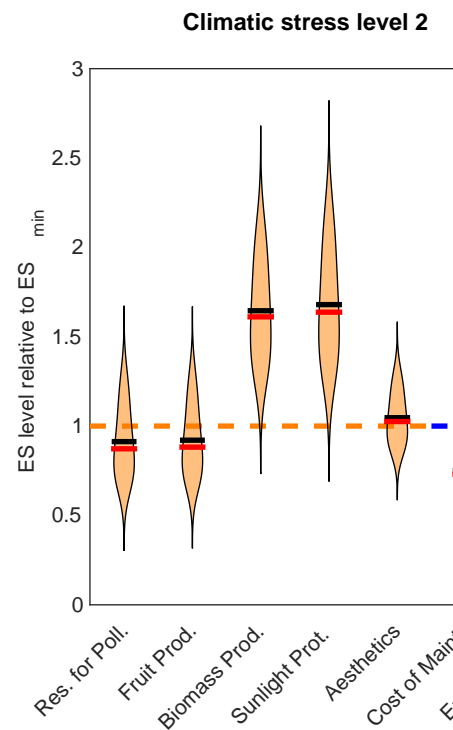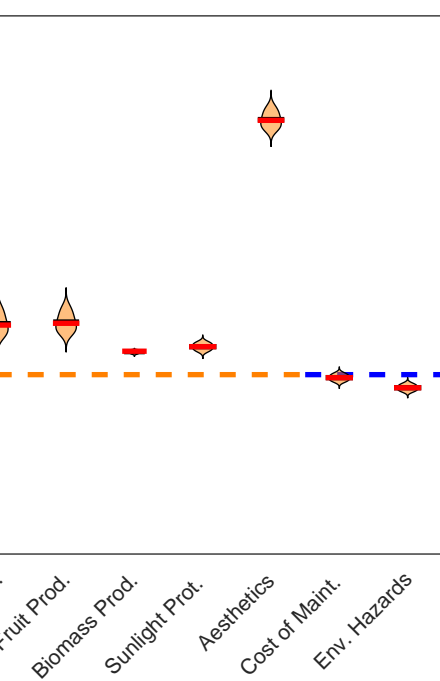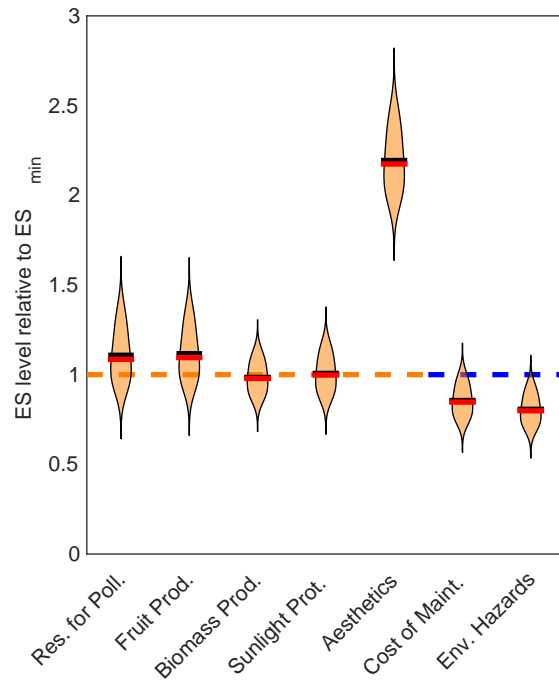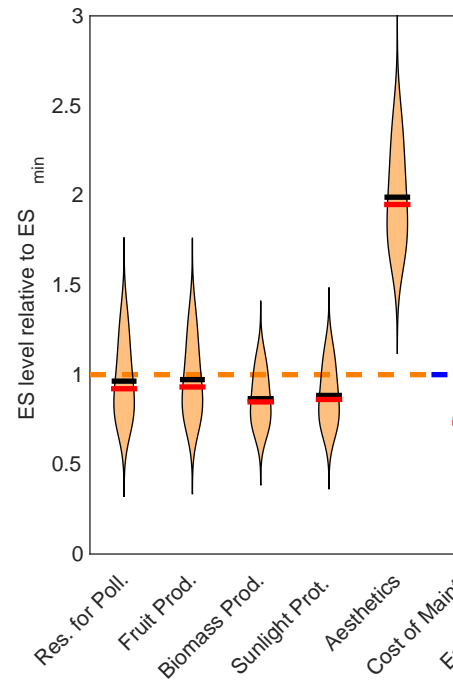

Supplement: Supplemental Information 4 [file peerj-13-18938-s004.zip › PACSEN-main/figures/figures_annexe/violin_full_scen_3.pdf]

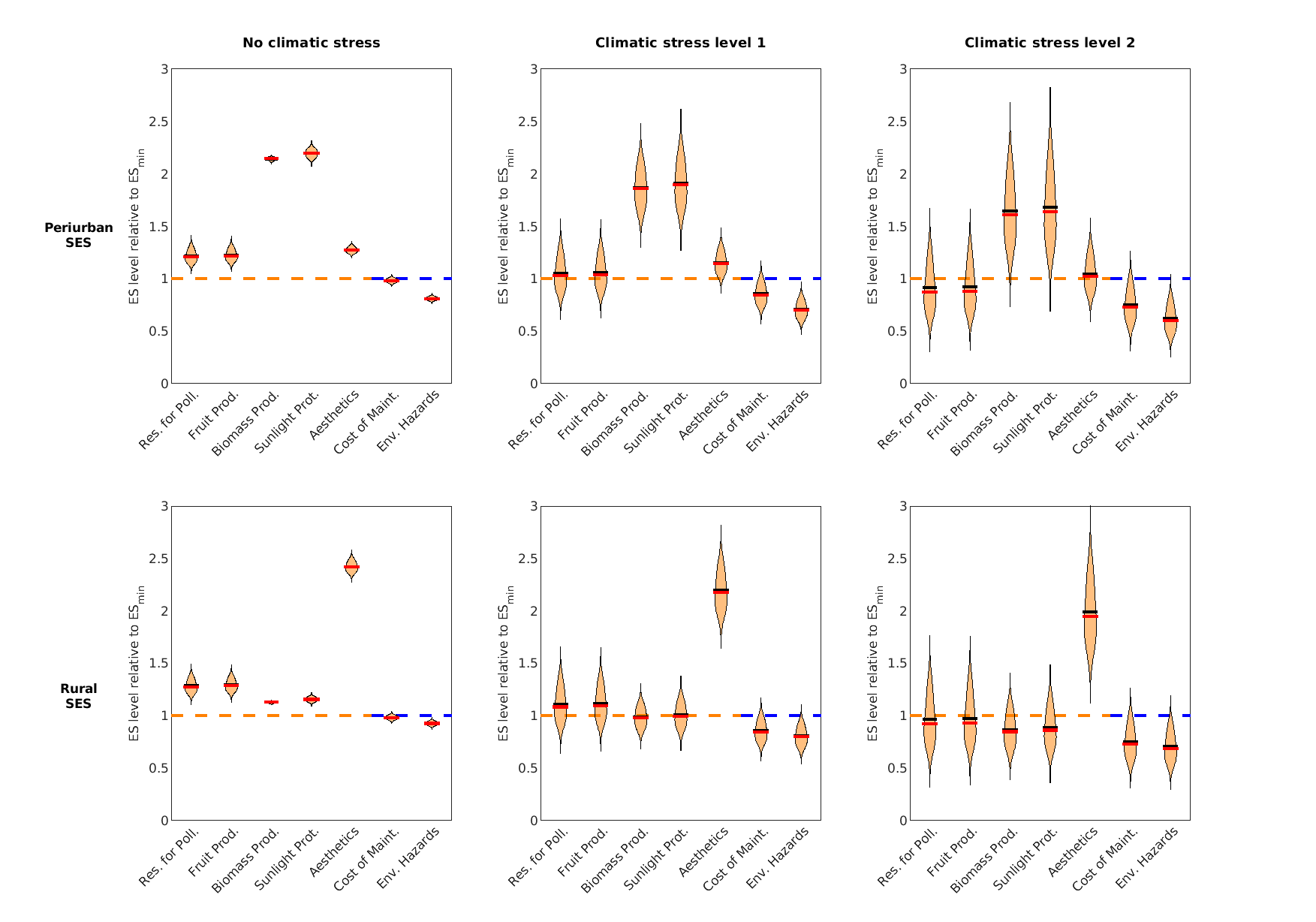

Supplement: Supplemental Information 4 [file peerj-13-18938-s004.zip › PACSEN-main/figures/figures_annexe/violin_full_scen_3.png]

No climatic stress

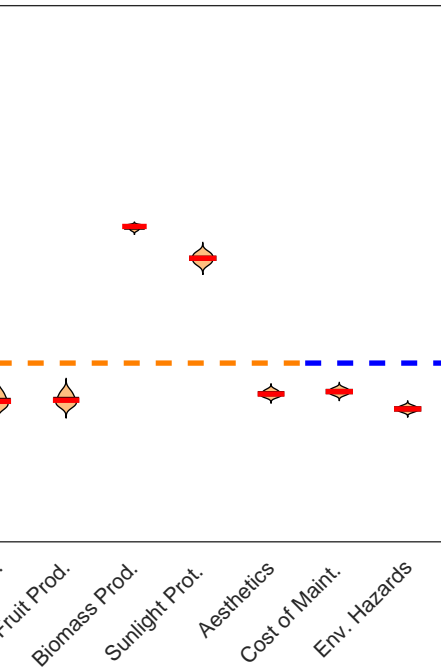

Climatic stress level 1

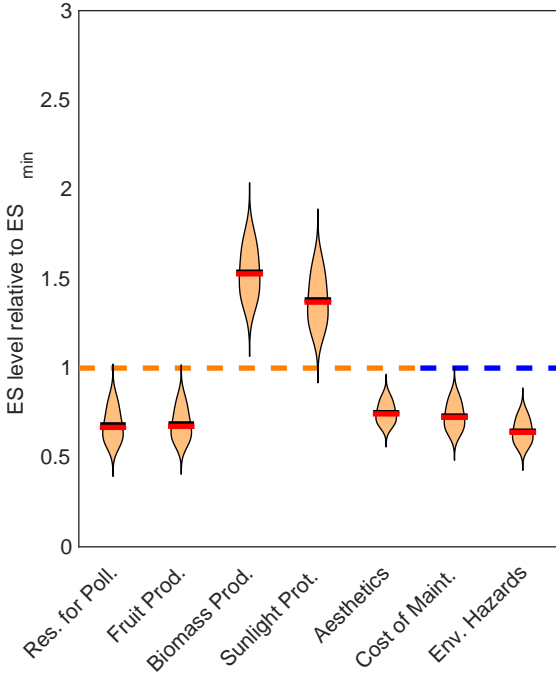

Climatic stress level 2

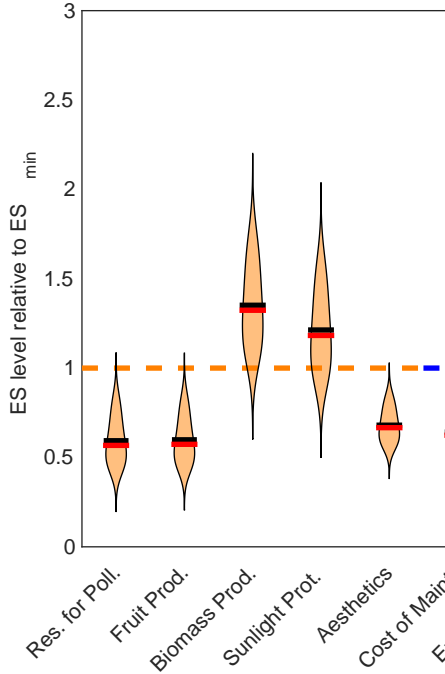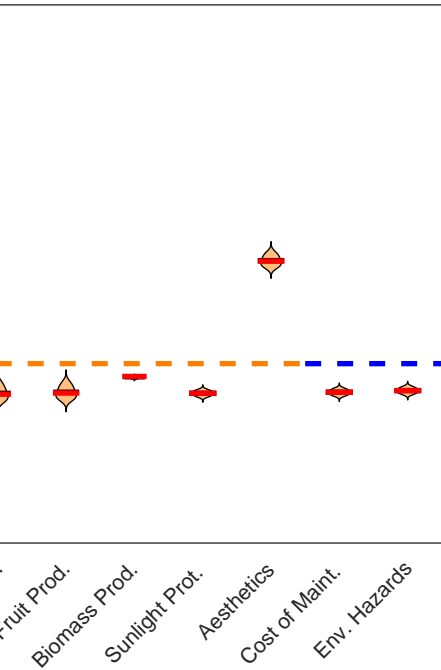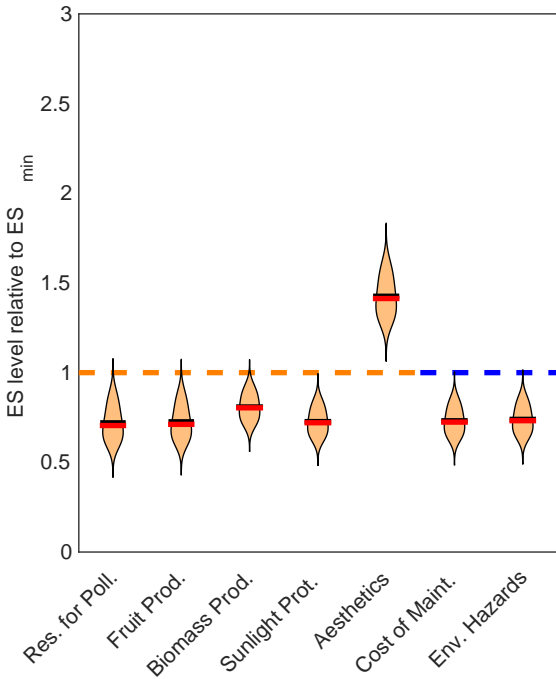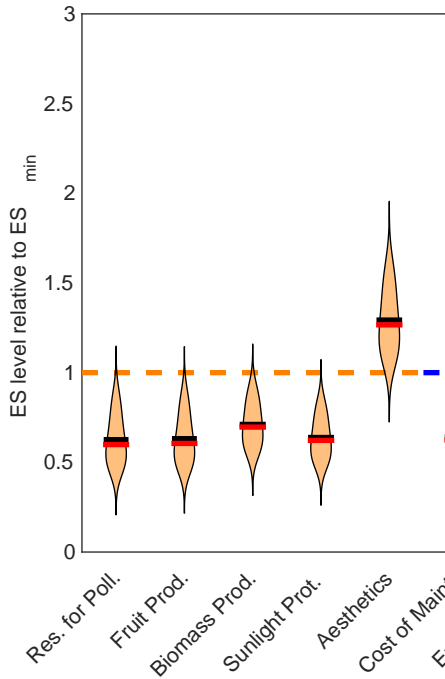

Supplement: Supplemental Information 4 [file peerj-13-18938-s004.zip › PACSEN-main/figures/figures_annexe/violin_full_scen_4.pdf]

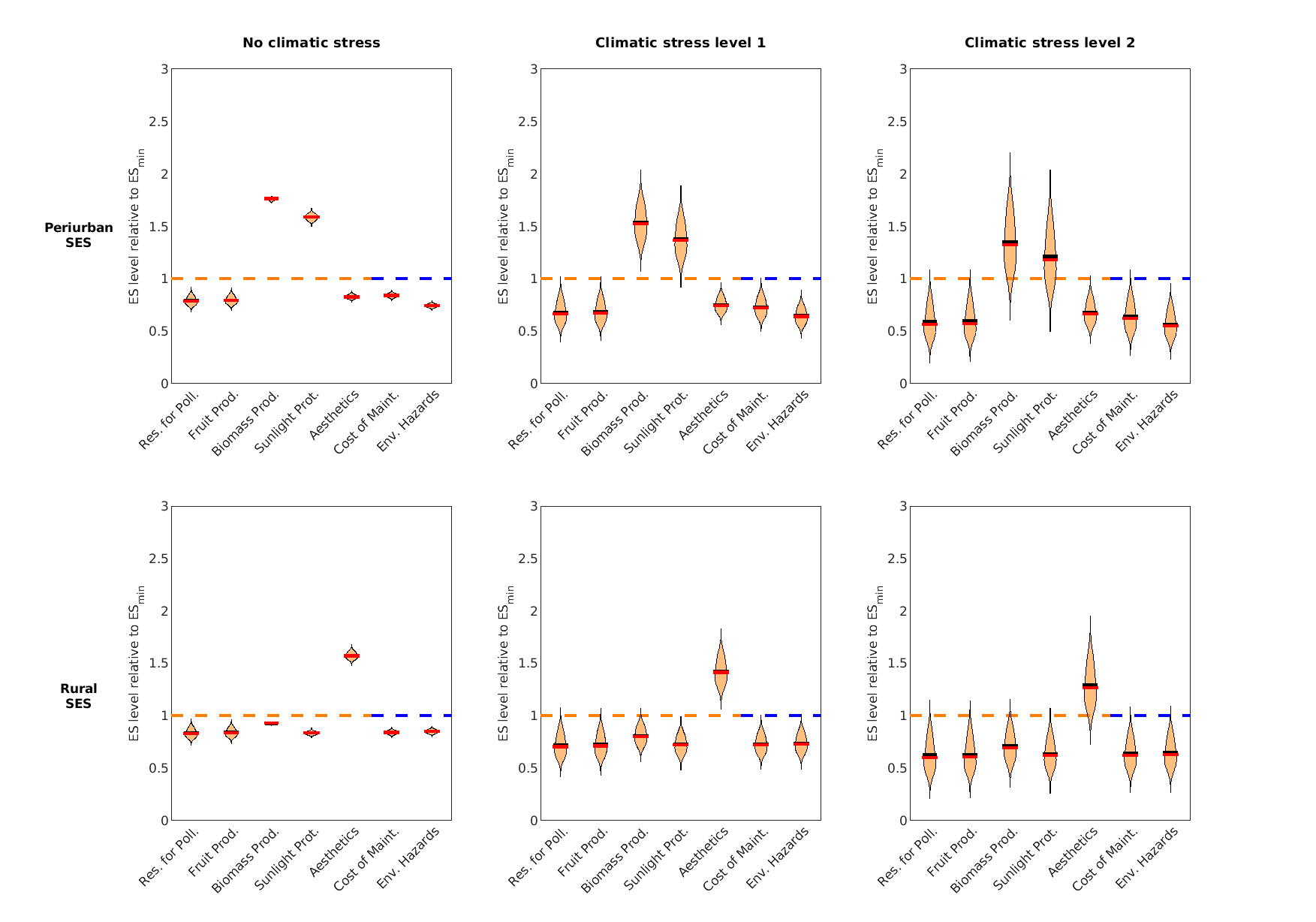

Supplement: Supplemental Information 4 [file peerj-13-18938-s004.zip › PACSEN-main/figures/figures_annexe/violin_full_scen_4.png]

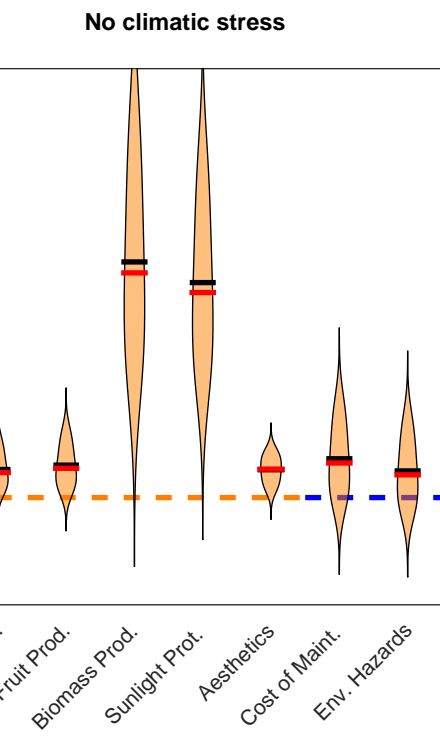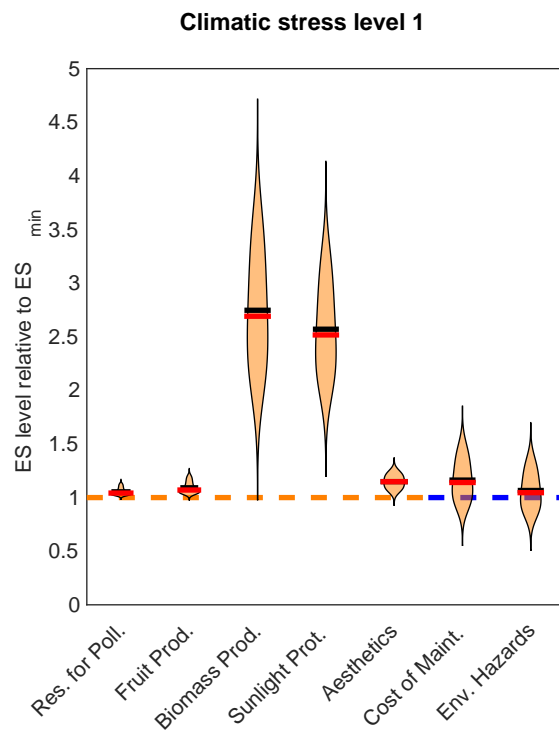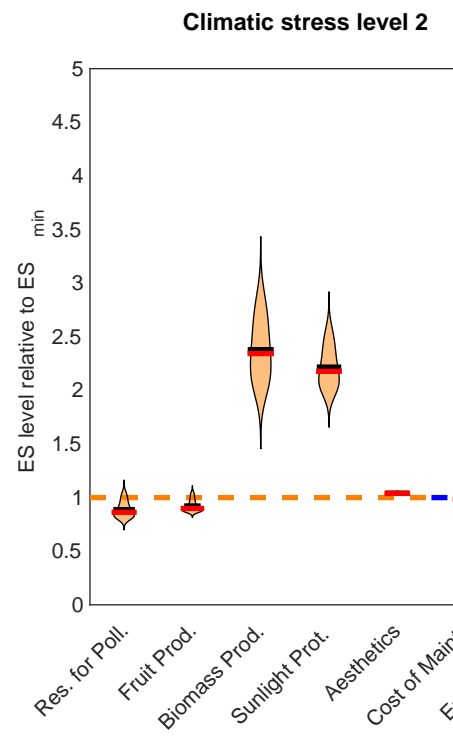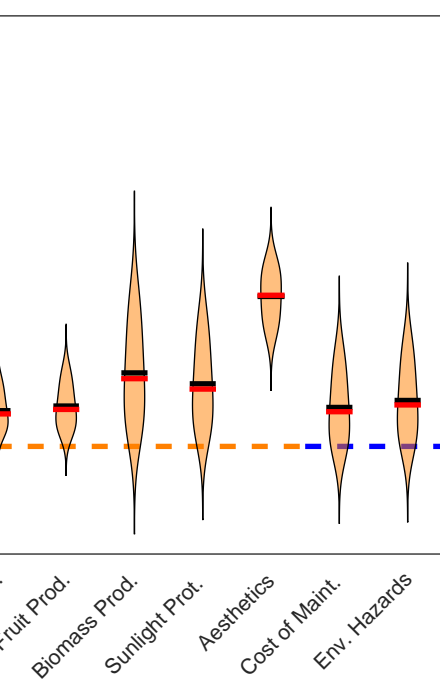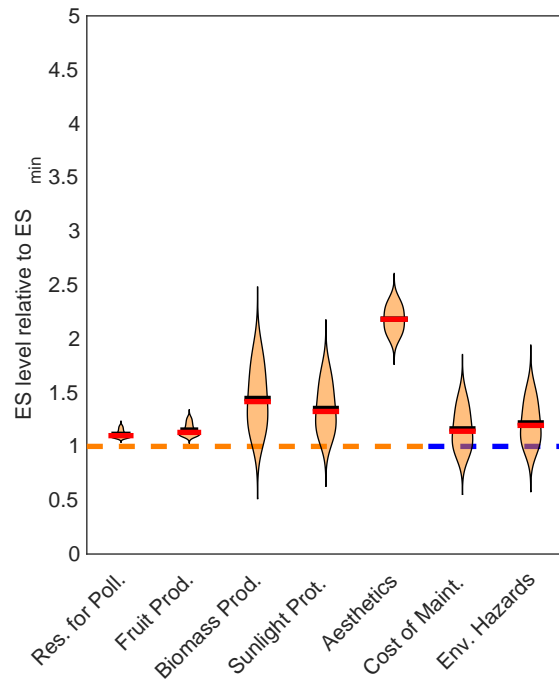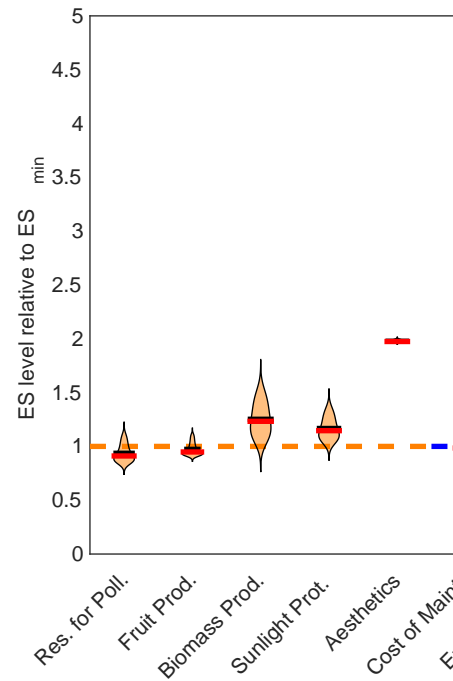

Supplement: Supplemental Information 4 [file peerj-13-18938-s004.zip › PACSEN-main/figures/figures_annexe/violin_full_scen_5.pdf]

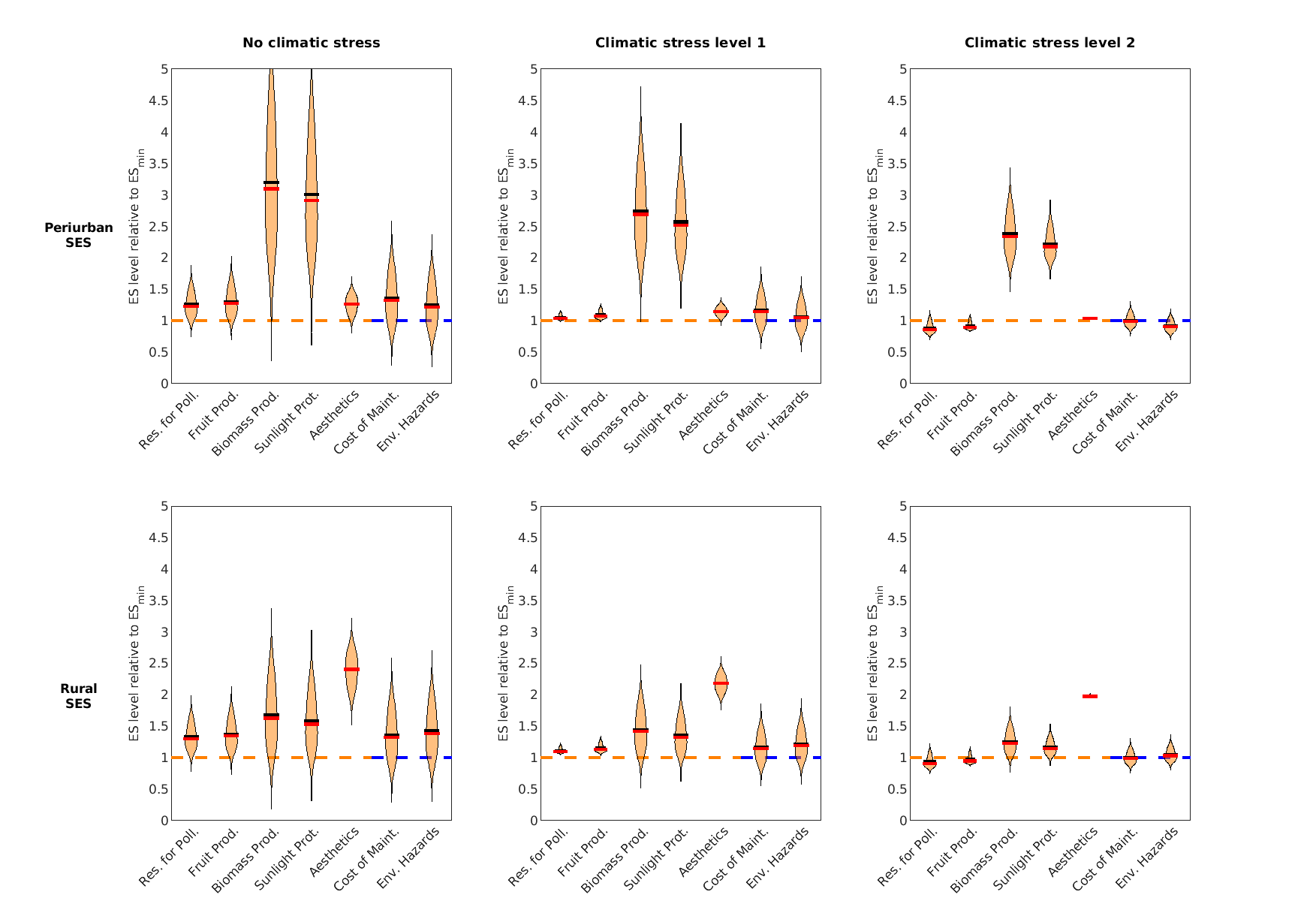

Supplement: Supplemental Information 4 [file peerj-13-18938-s004.zip › PACSEN-main/figures/figures_annexe/violin_full_scen_5.png]

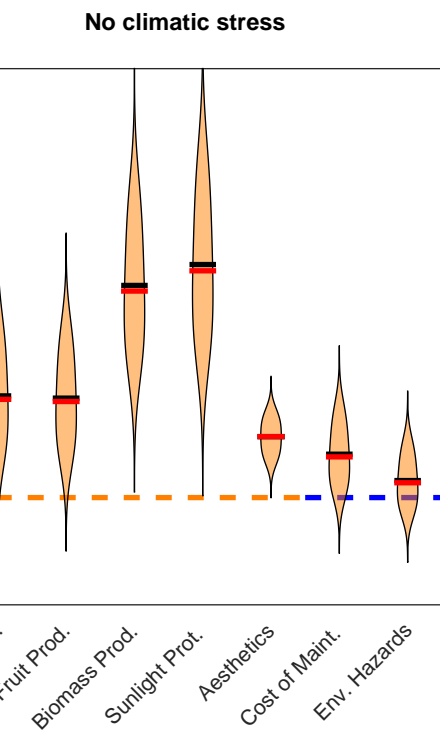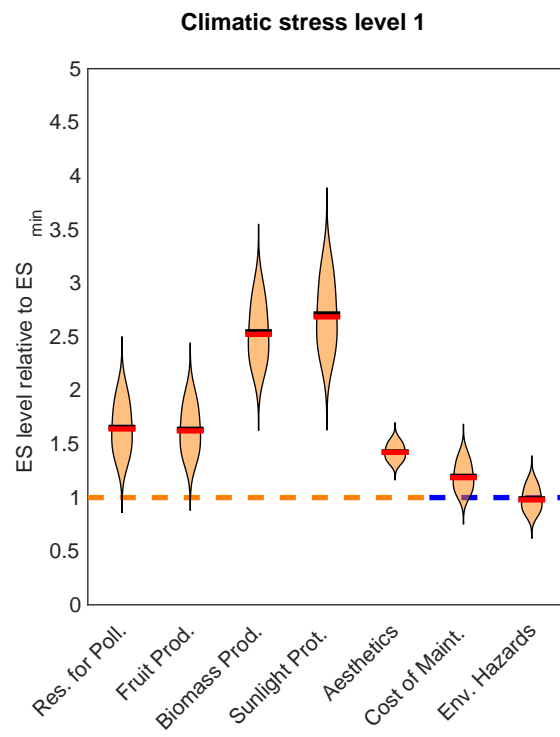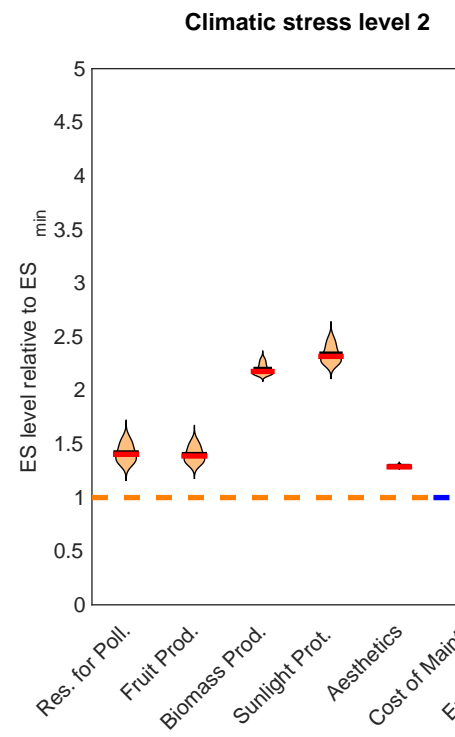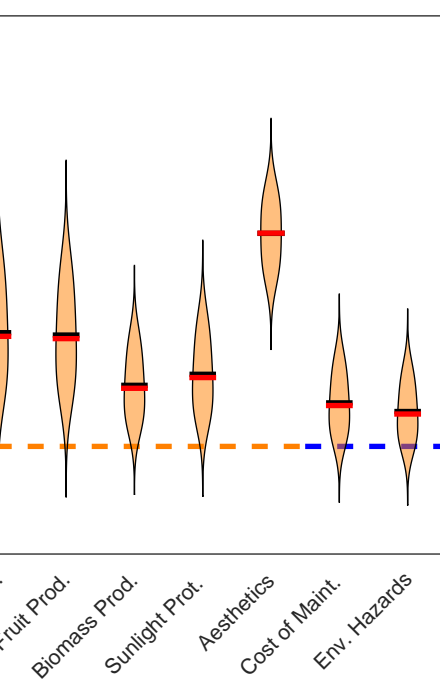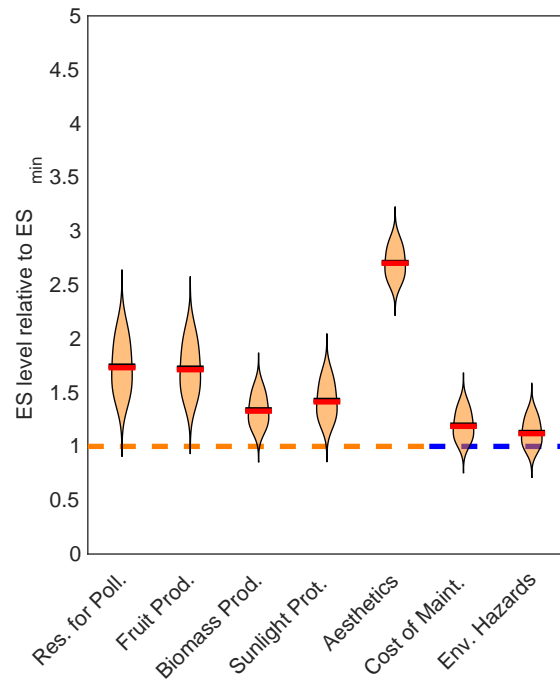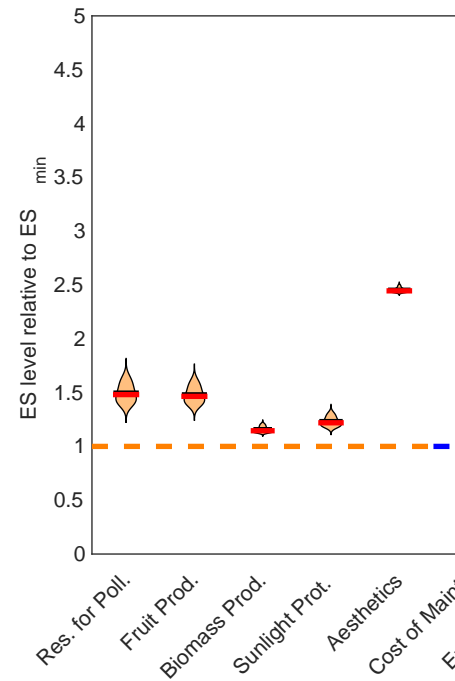

Supplement: Supplemental Information 4 [file peerj-13-18938-s004.zip › PACSEN-main/figures/figures_annexe/violin_full_scen_6.pdf]

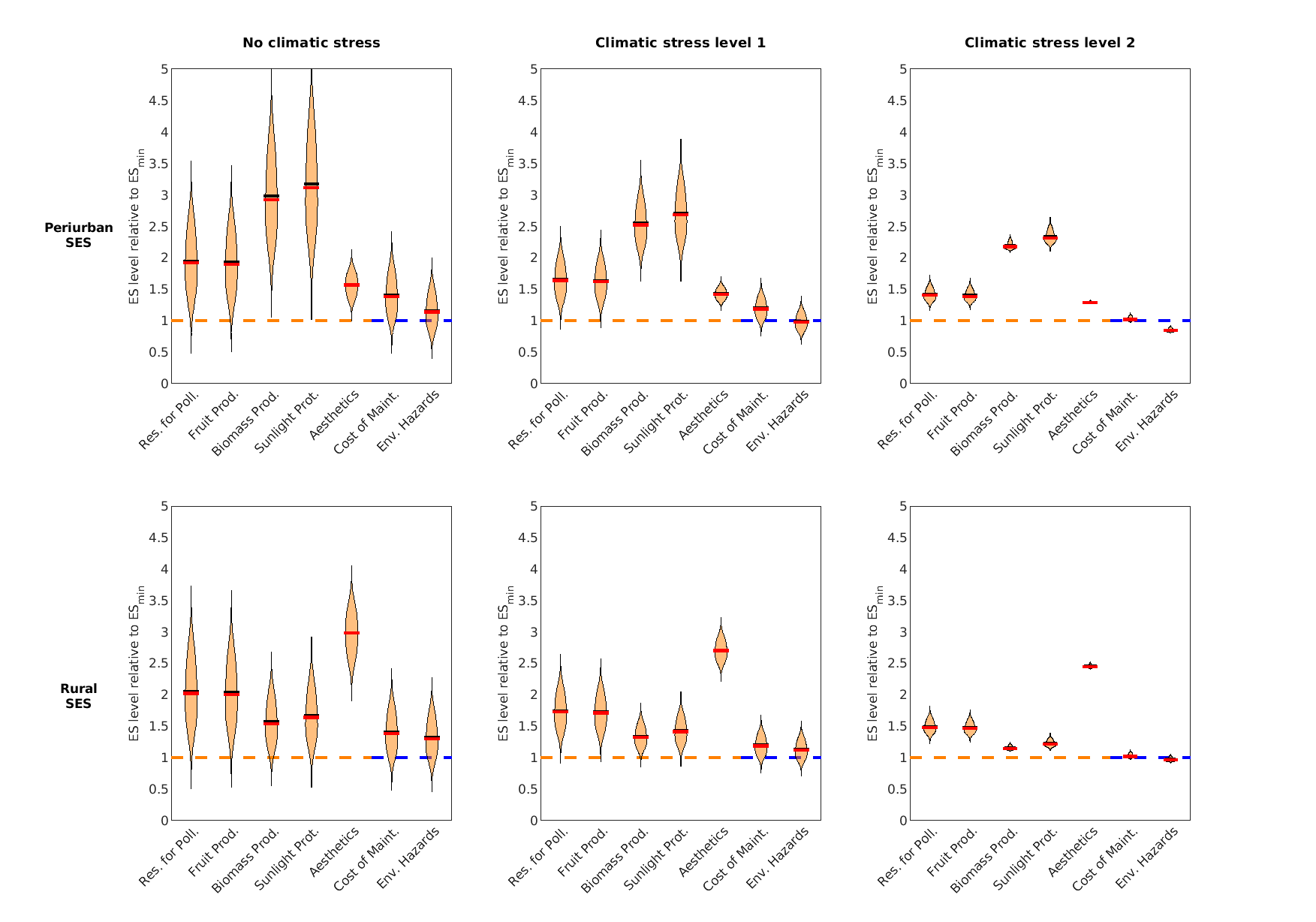

Supplement: Supplemental Information 4 [file peerj-13-18938-s004.zip › PACSEN-main/figures/figures_annexe/violin_full_scen_6.png]

No climatic stress

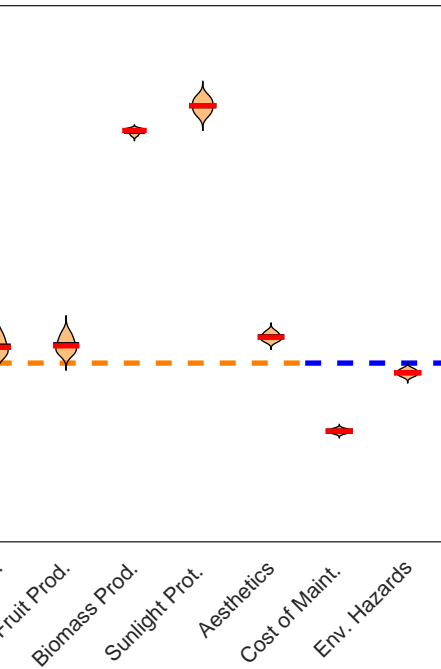

Climatic stress level 1

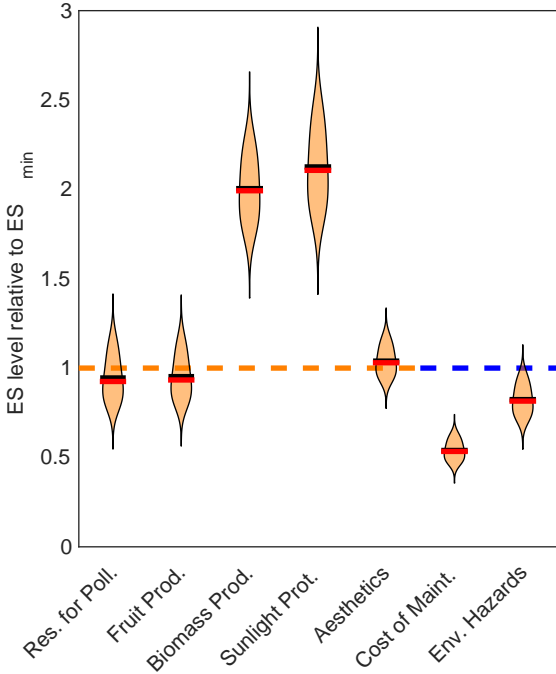

Climatic stress level 2

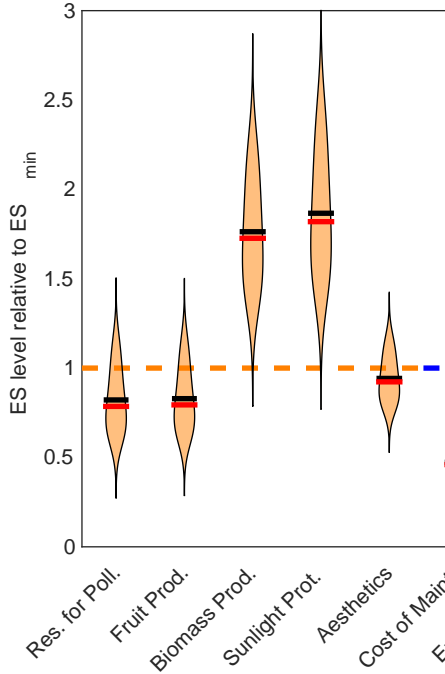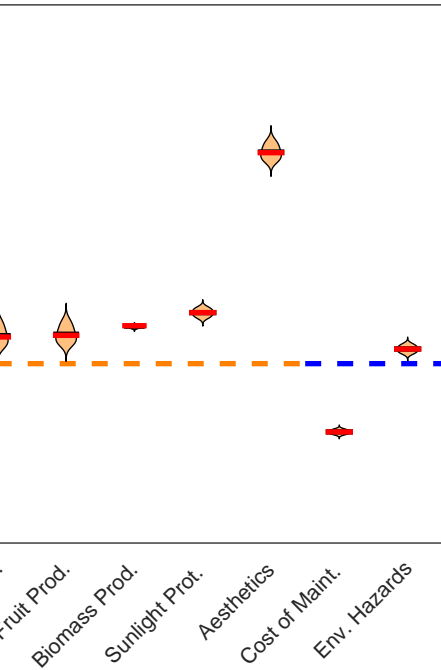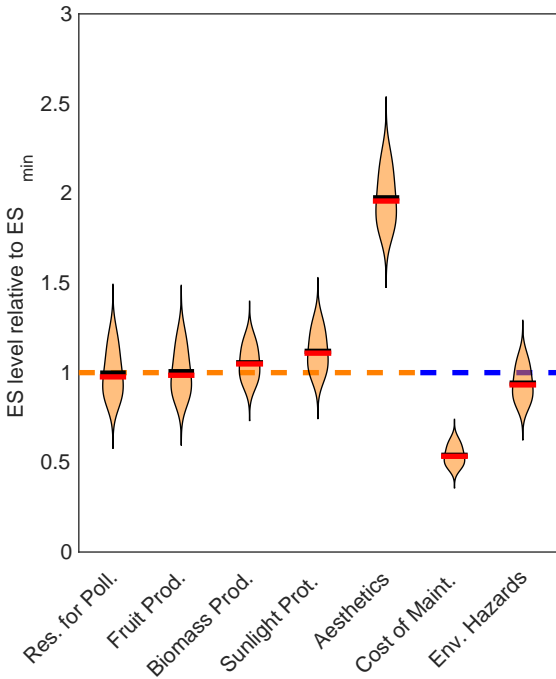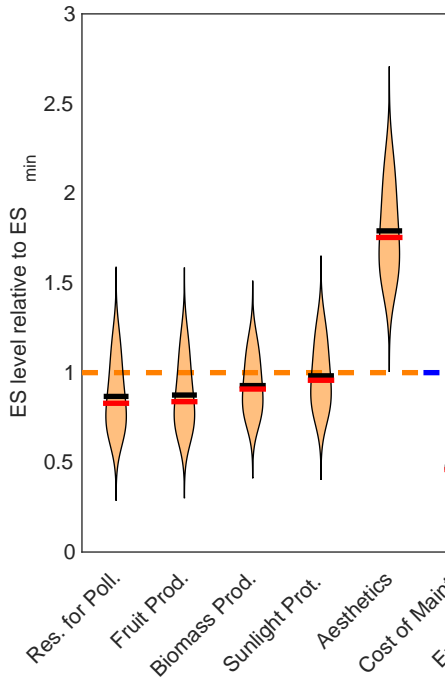

Supplement: Supplemental Information 4 [file peerj-13-18938-s004.zip › PACSEN-main/figures/figures_annexe/violin_full_scen_7.pdf]

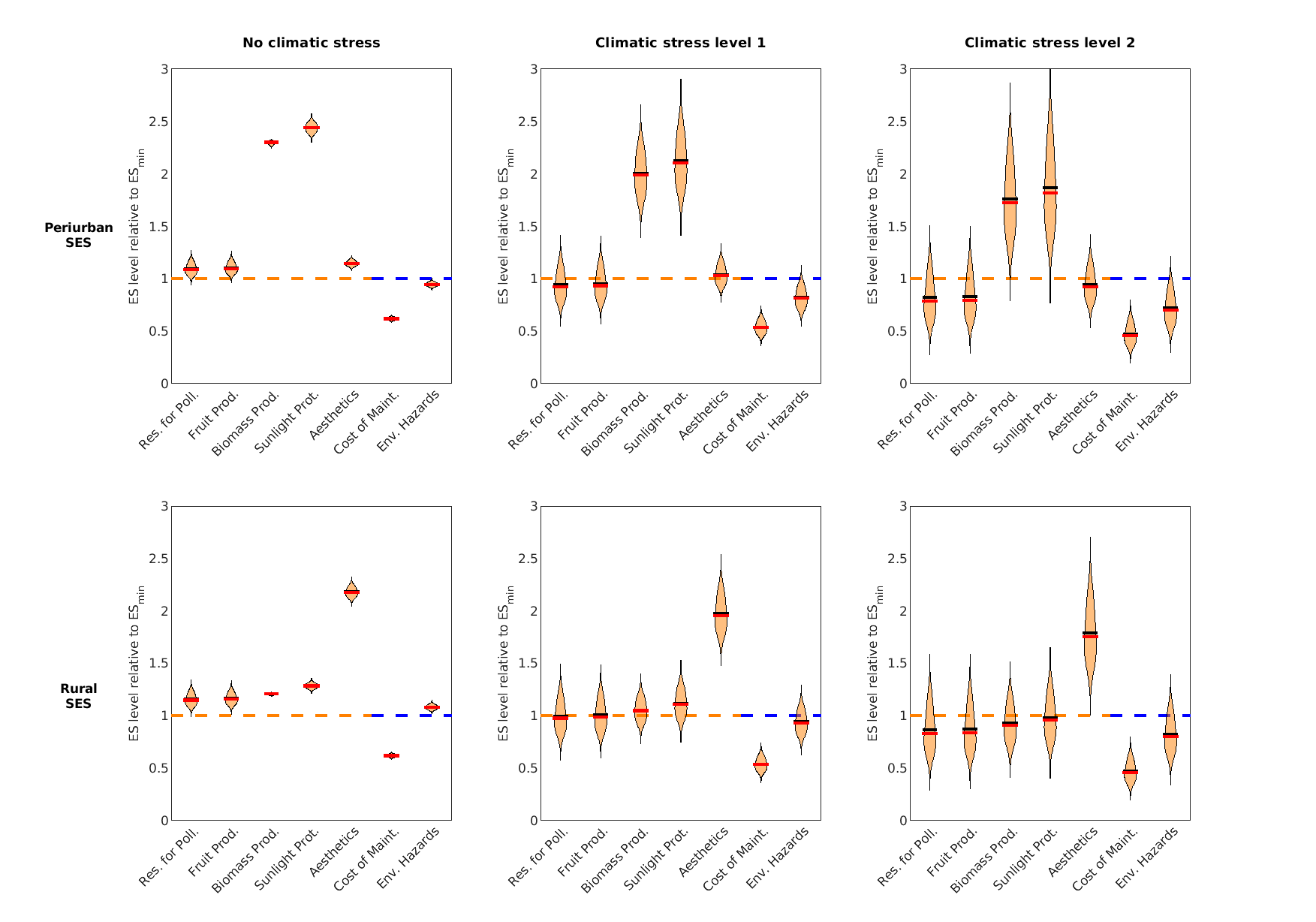

Supplement: Supplemental Information 4 [file peerj-13-18938-s004.zip › PACSEN-main/figures/figures_annexe/violin_full_scen_7.png]

No climatic stress

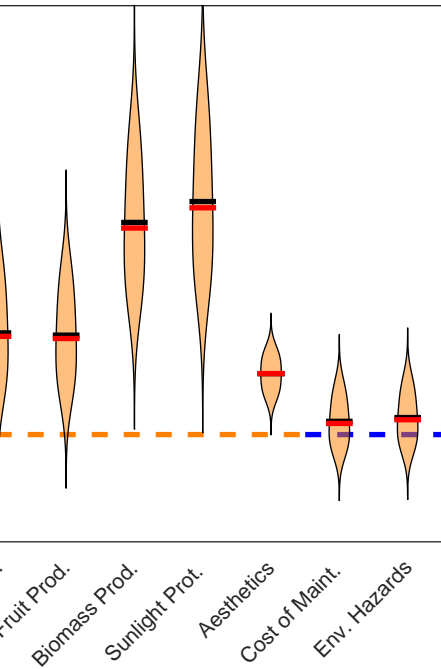

Climatic stress level 1

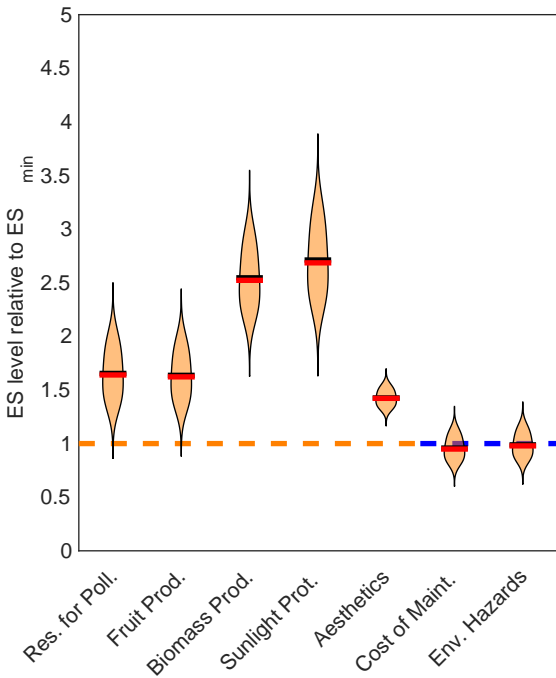

Climatic stress level 2

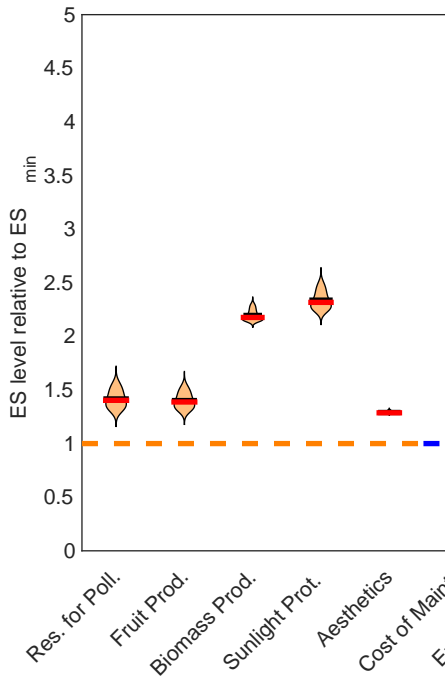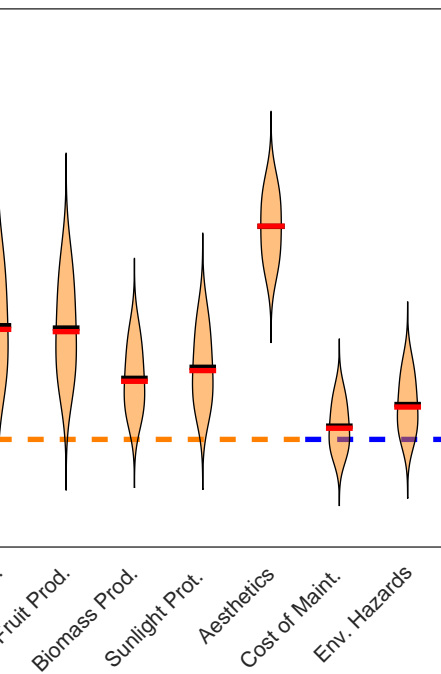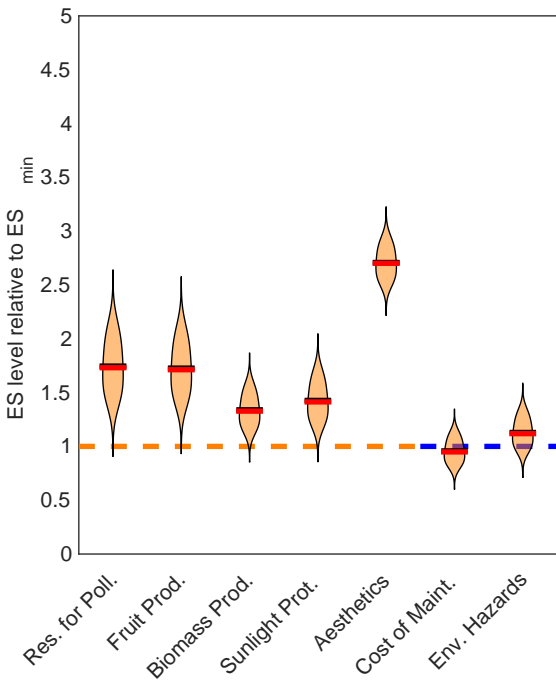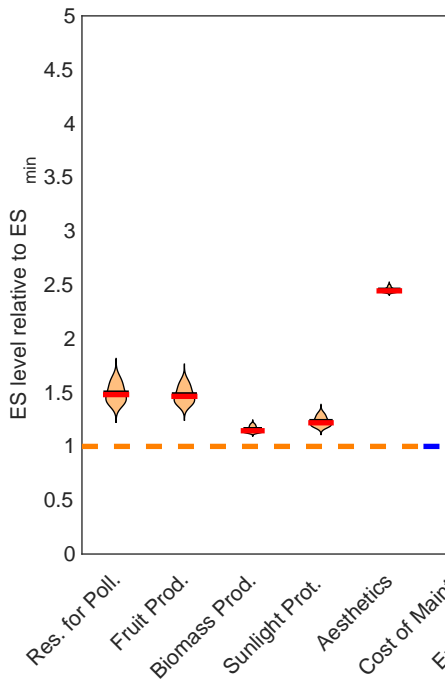

Supplement: Supplemental Information 4 [file peerj-13-18938-s004.zip › PACSEN-main/figures/figures_annexe/violin_full_scen_8.pdf]

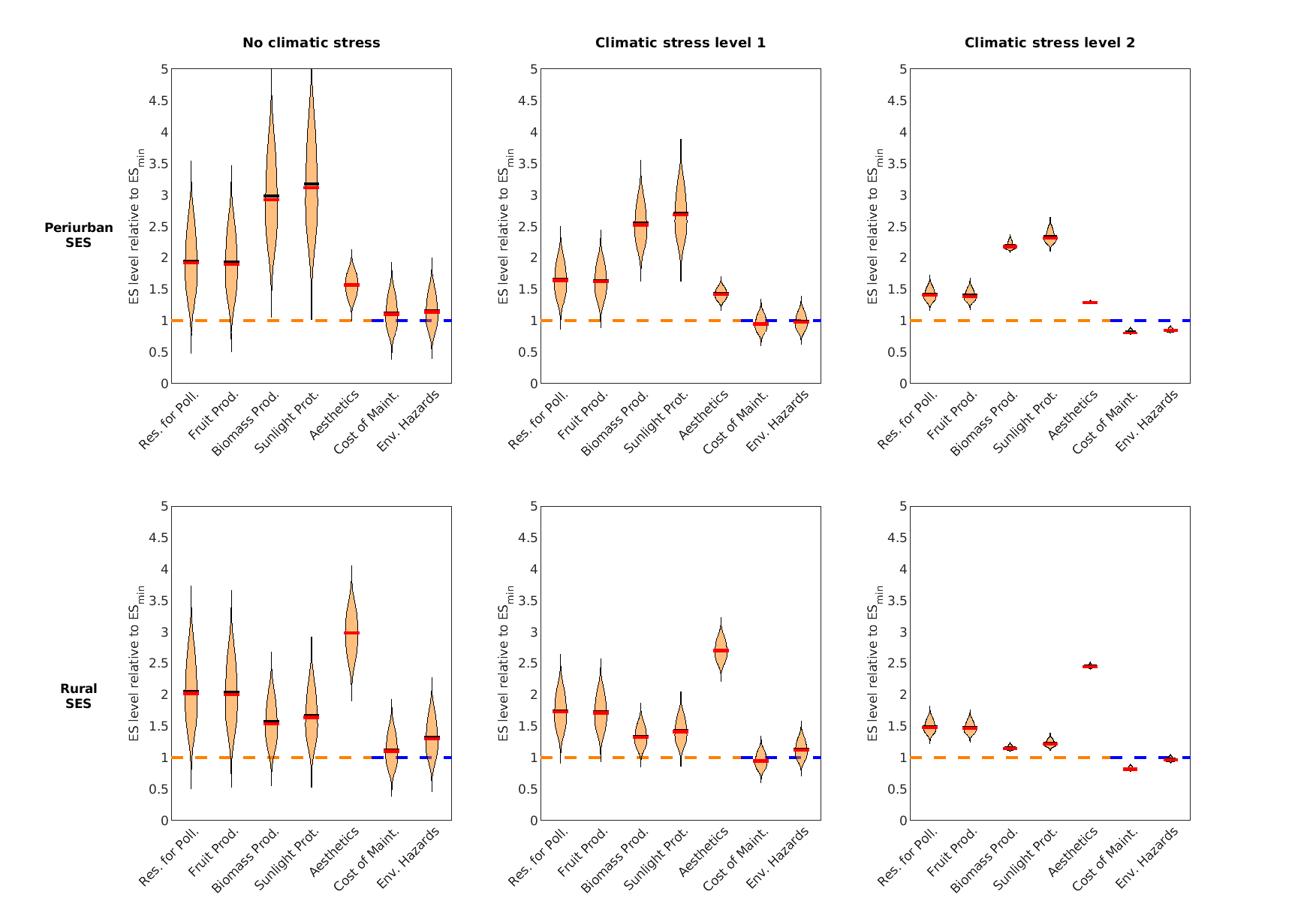

Supplement: Supplemental Information 4 [file peerj-13-18938-s004.zip › PACSEN-main/figures/figures_annexe/violin_full_scen_8.png]

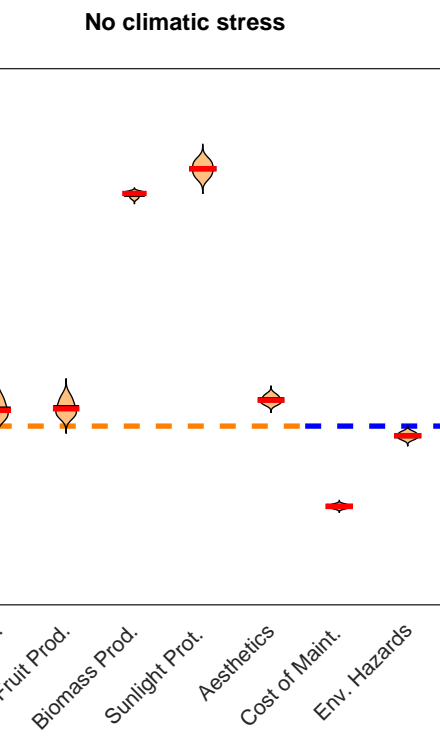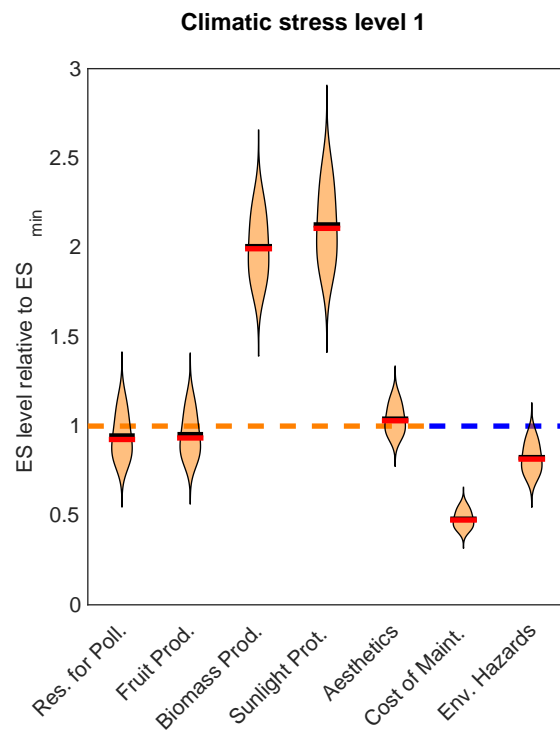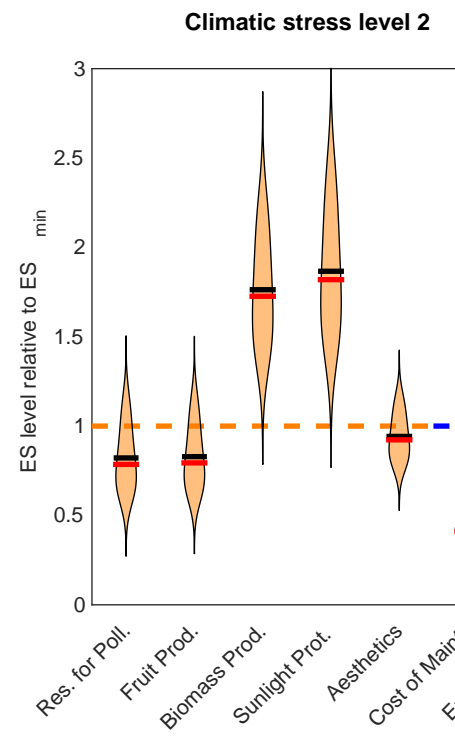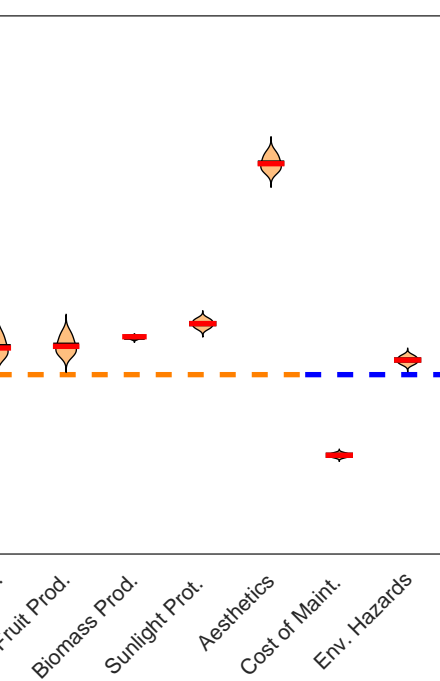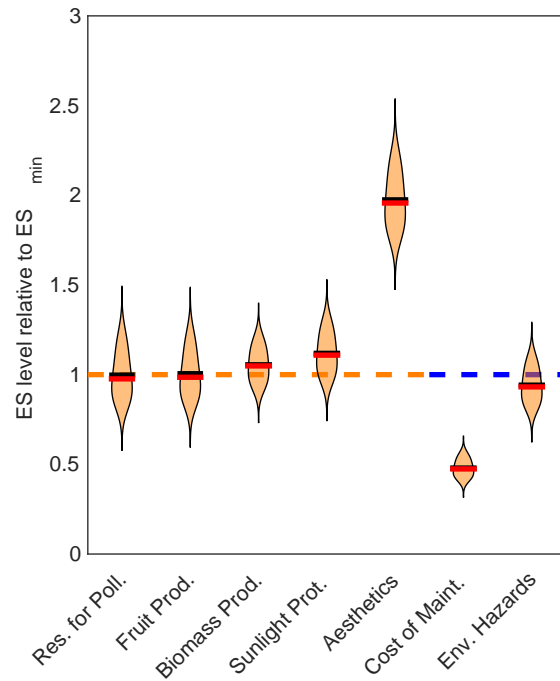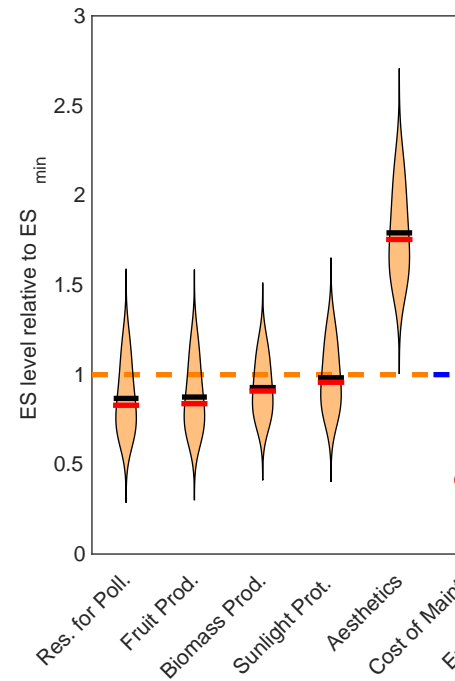

Supplement: Supplemental Information 4 [file peerj-13-18938-s004.zip › PACSEN-main/figures/figures_annexe/violin_full_scen_9.pdf]

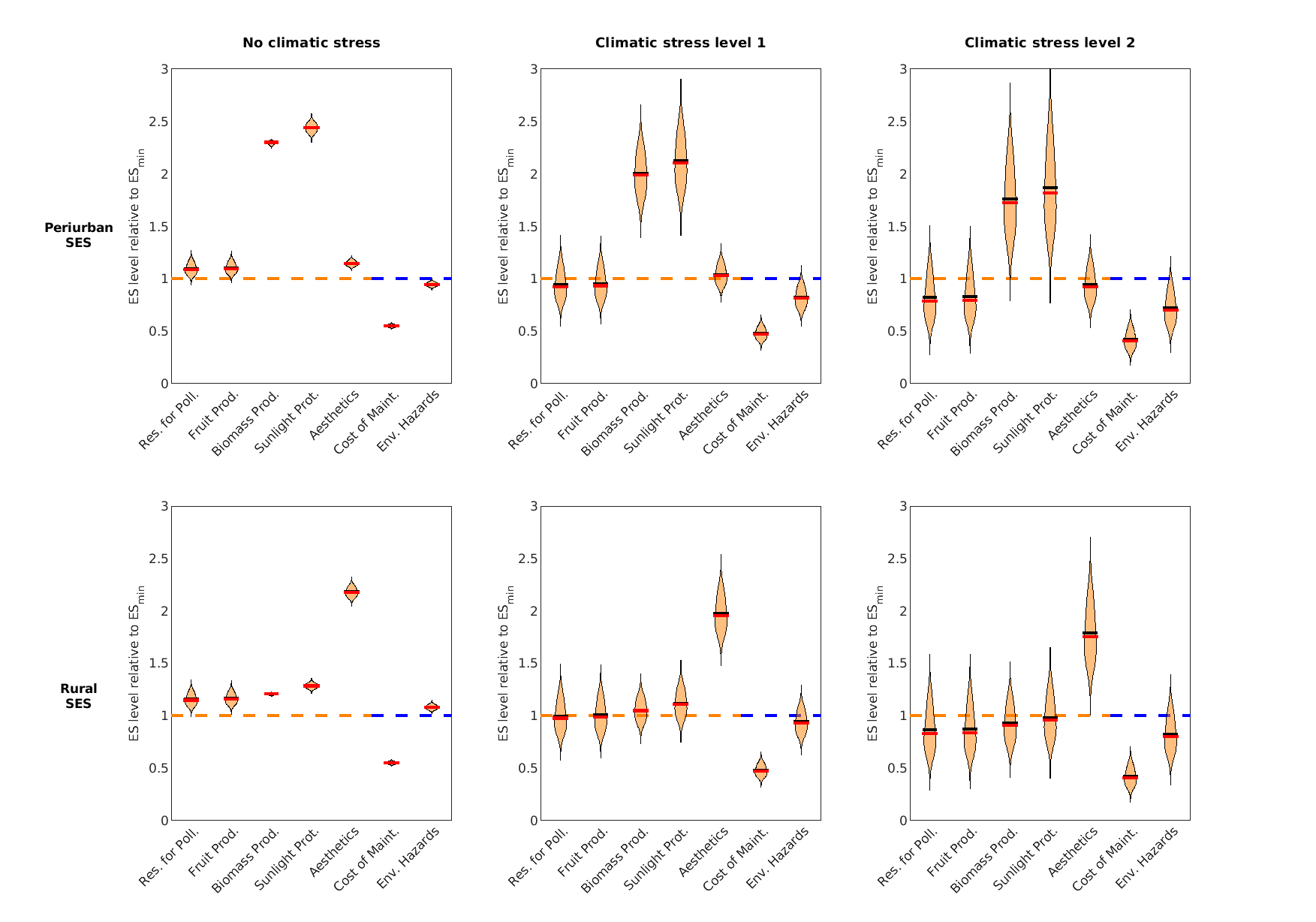

Supplement: Supplemental Information 4 [file peerj-13-18938-s004.zip › PACSEN-main/figures/figures_annexe/violin_full_scen_9.png]

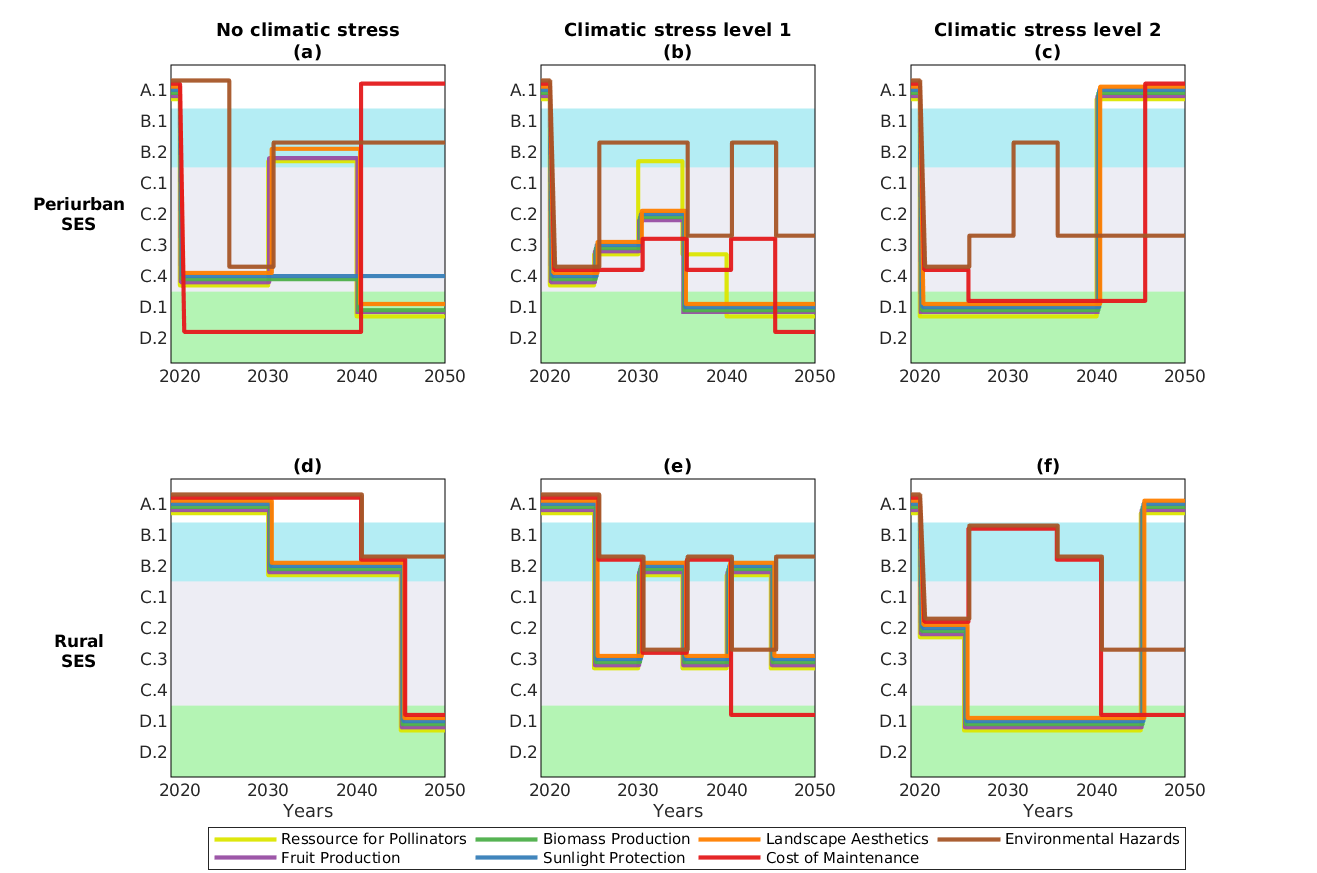

Supplement: Supplemental Information 4 [file peerj-13-18938-s004.zip › PACSEN-main/figures/max_indiv_apm_action.png]

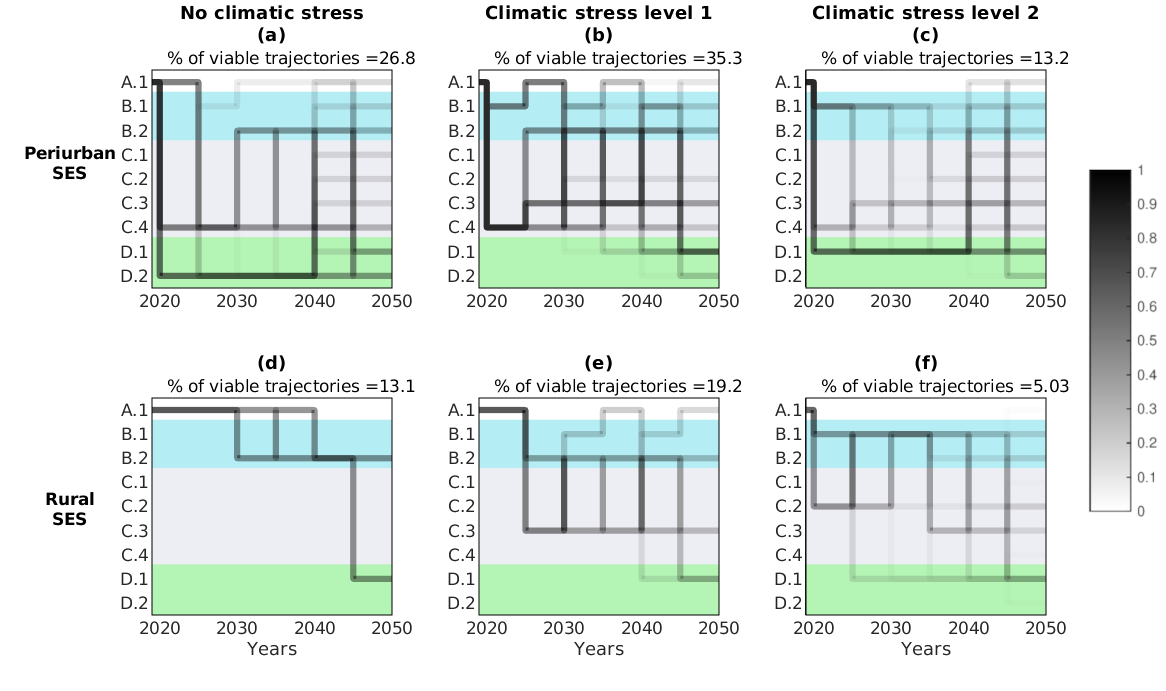

Supplement: Supplemental Information 4 [file peerj-13-18938-s004.zip › PACSEN-main/figures/viable_density_output.png]

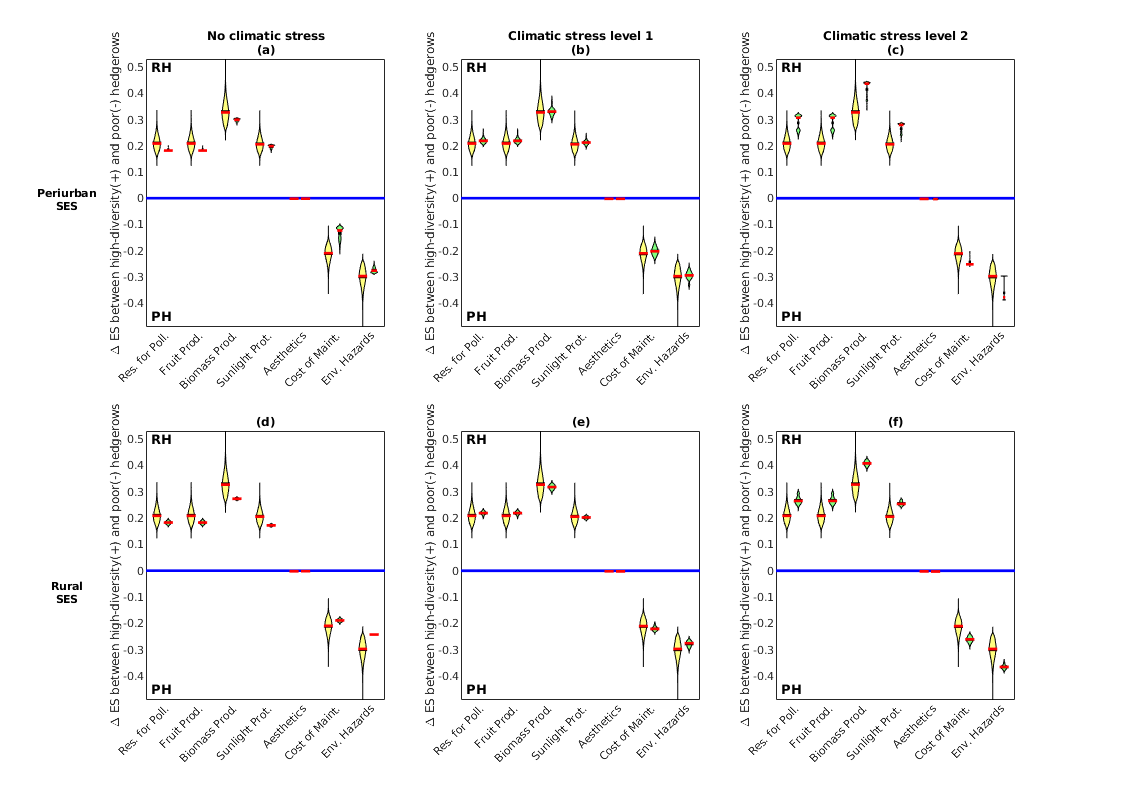

Supplement: Supplemental Information 4 [file peerj-13-18938-s004.zip › PACSEN-main/figures/violin_delta_diversity.png]

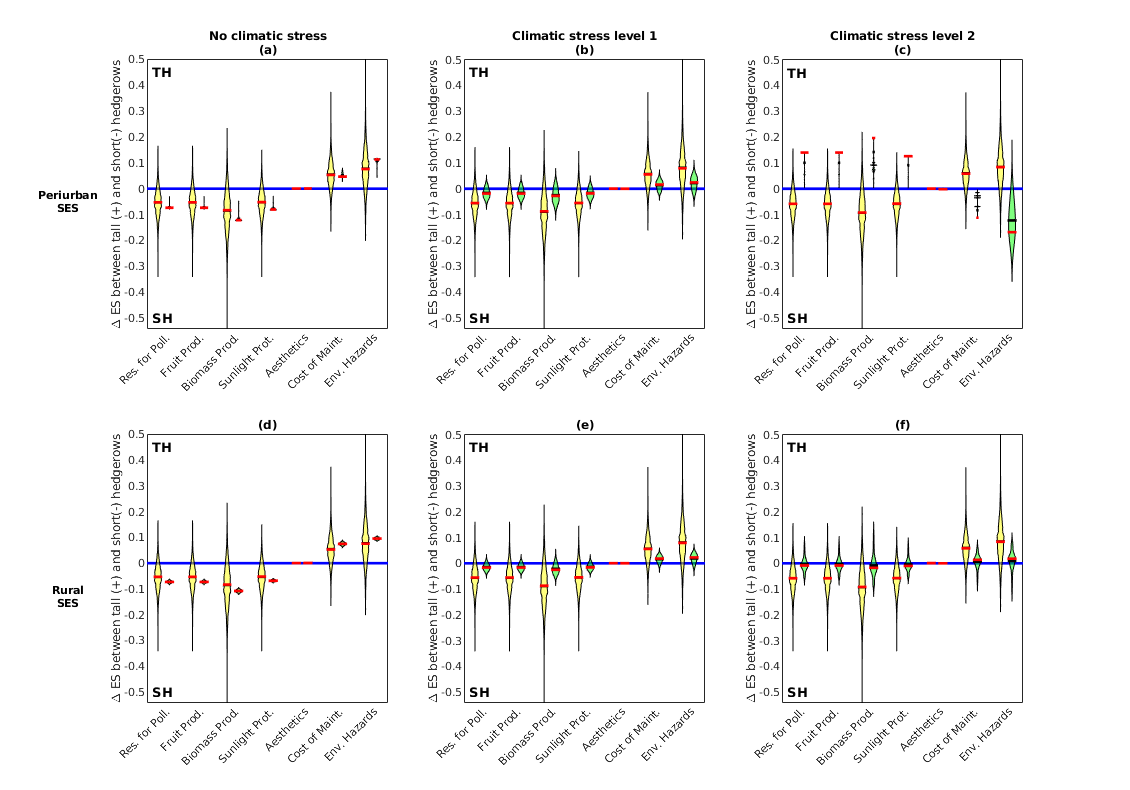

Supplement: Supplemental Information 4 [file peerj-13-18938-s004.zip › PACSEN-main/figures/violin_delta_height.png]

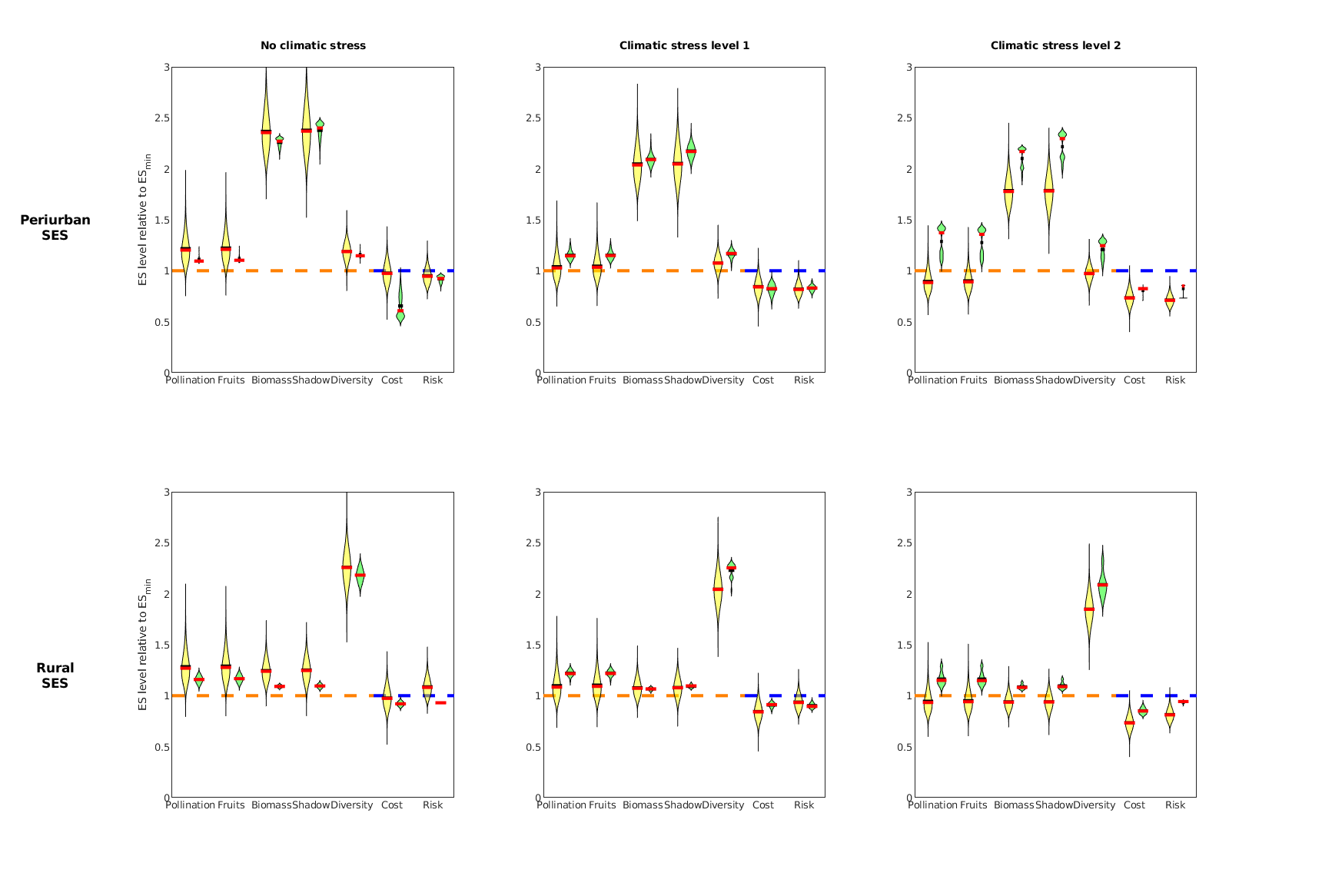

Supplement: Supplemental Information 4 [file peerj-13-18938-s004.zip › PACSEN-main/figures/violin_es_june.png]

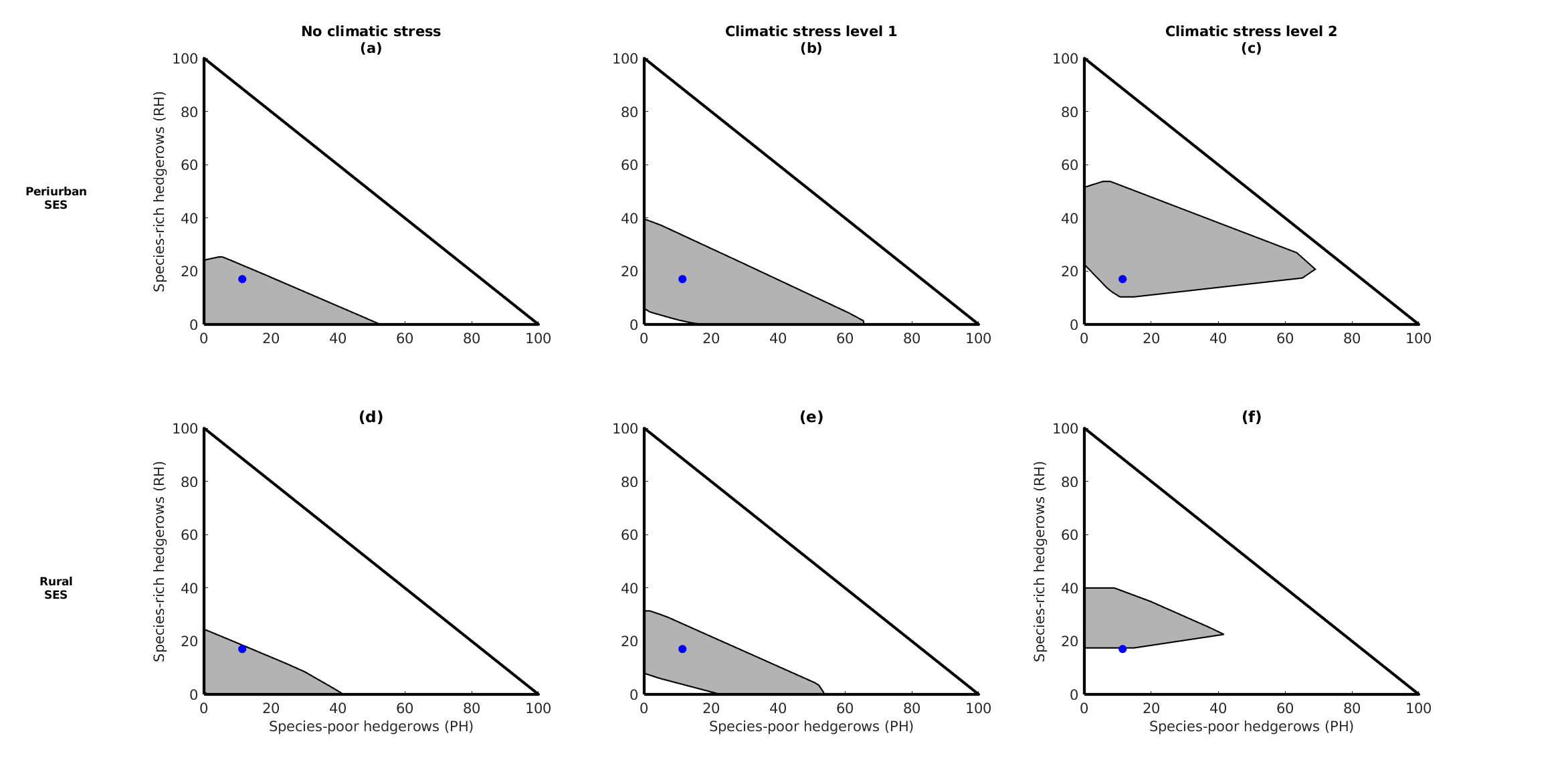

Supplement: Supplemental Information 4 [file peerj-13-18938-s004.zip › PACSEN-main/figures/vk_div.png]
