# Supplementary material for: Integrating adaptation pathways and Ostrom’s framework for sustainable governance of social-ecological systems in a changing world
Source: PeerJ. 2025 Feb 24;13:e18938. doi: 10.7717/peerj.18938 (PMC11867035; doi:10.7717/peerj.18938)
Supplement: Supplemental Information 5 — Archetypal results for the nine pathways where the CCA and KCA have been fixed and do not change during 80 years. [file peerj-13-18938-s005.pdf]

# Supplementary Results S5

## S5.0. Supplementary figures associated with Figures 9 and 10 for tall and short hedgerows

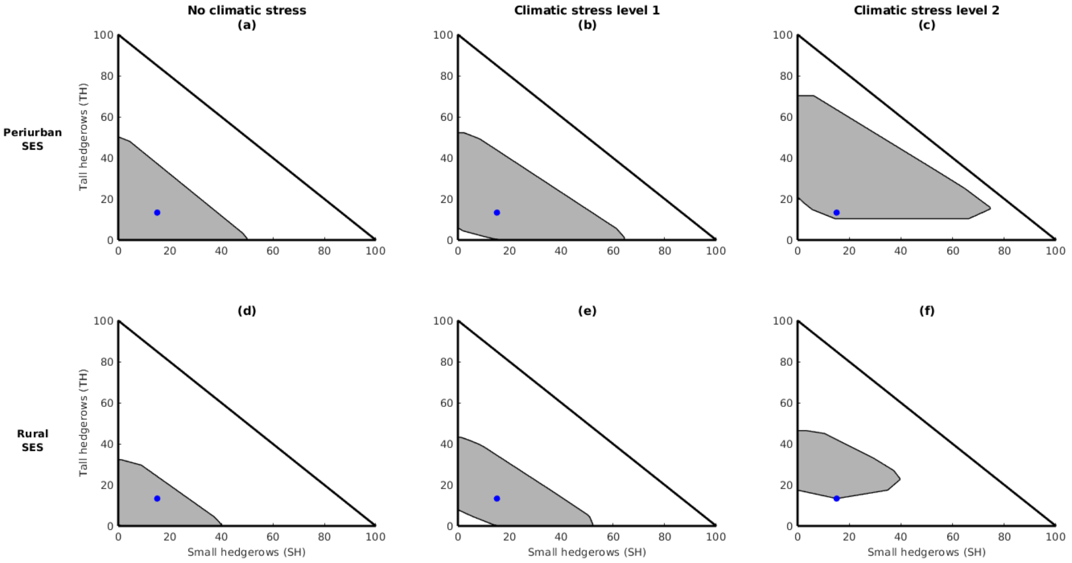


**Figure S9. Influence of hedgerow height on the size and shape of the viability kernel.** 2D cut of the four dimensional viability kernel, and expressed as a function of only small hedgerows (SH) and tall hedgerows (TH) for the two types of SES (rural, peri-urban), and the three levels of climate stress. The blue dots represent the initial state of hedgerow types in both rural and peri-urban SES, and were selected to be the same to facilitate comparison. Blue dots close to the viability kernel limit mean that there are less options to adapt the hedgerow network through different nested governance arrangements changes, and thus are less secured.

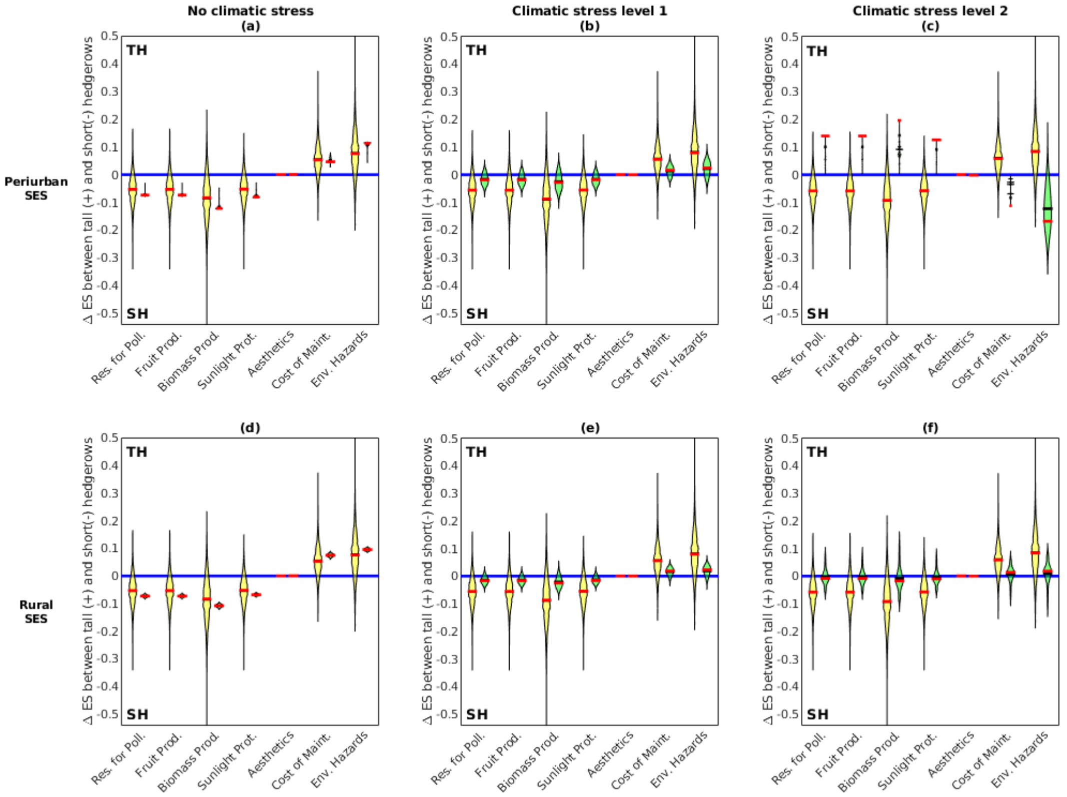


**Figure S10.** Measure of the security gains $\Delta ES_{TH\text{-}SH}$ relative to the limits of the ES satisfactory domain (i.e. distance from the blue baseline) for every ecosystem service (ES+) and disservice (ES-), when switching the target of management from short hedgerows (SH) to tall ones (TH), during an adaptive governance pathway. Panels a,b,c are for peri-urban SES and d,e,f for rural SES, along with increasing climatic stress. As per explanation in method section 2.6.2, $\Delta ES_{TH\text{-}SH} > 0$ means greater security gains (and thus incentives) when acting more on TH than on SH; whereas $\Delta ES_{TH\text{-}SH} < 0$ means greater incentives to act more on SH than on TH. The violins represent the probability density of $\Delta ES$ values (with median in red) associated with all the adaptation pathways that are both viable and non-viable (yellow violins), or viable only (green violins). ES full name are provided in figure 8.

## S5.1. Ecosystem services dynamics per hedgerow type for DAPP maps maximizing independently one of the seven ES

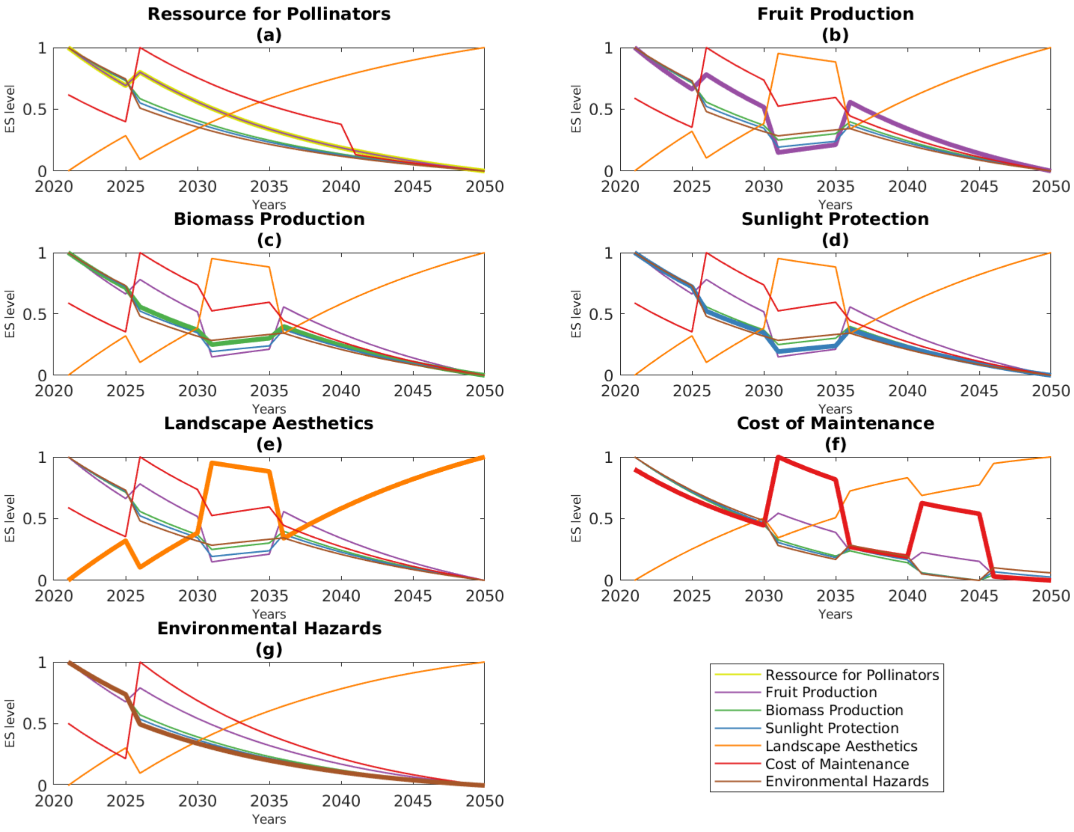


**Figure S5.1.1.** Temporal dynamics of ecosystem (dis)services (ES) delivered by small hedges with low diversity, extracted from the optimal DAPP map presented in figure 9a for a peri-urban SES with climatic stress level 1. Panels show the dynamics of seven ES as a response to the selection of the most optimal adaptation pathway that aim at minimizing or maximizing a) pollination, b) fruit production, c) biomass production, d) sunlight protection, e) landscape aesthetics, f) cost of maintenance, g) environmental hazards.

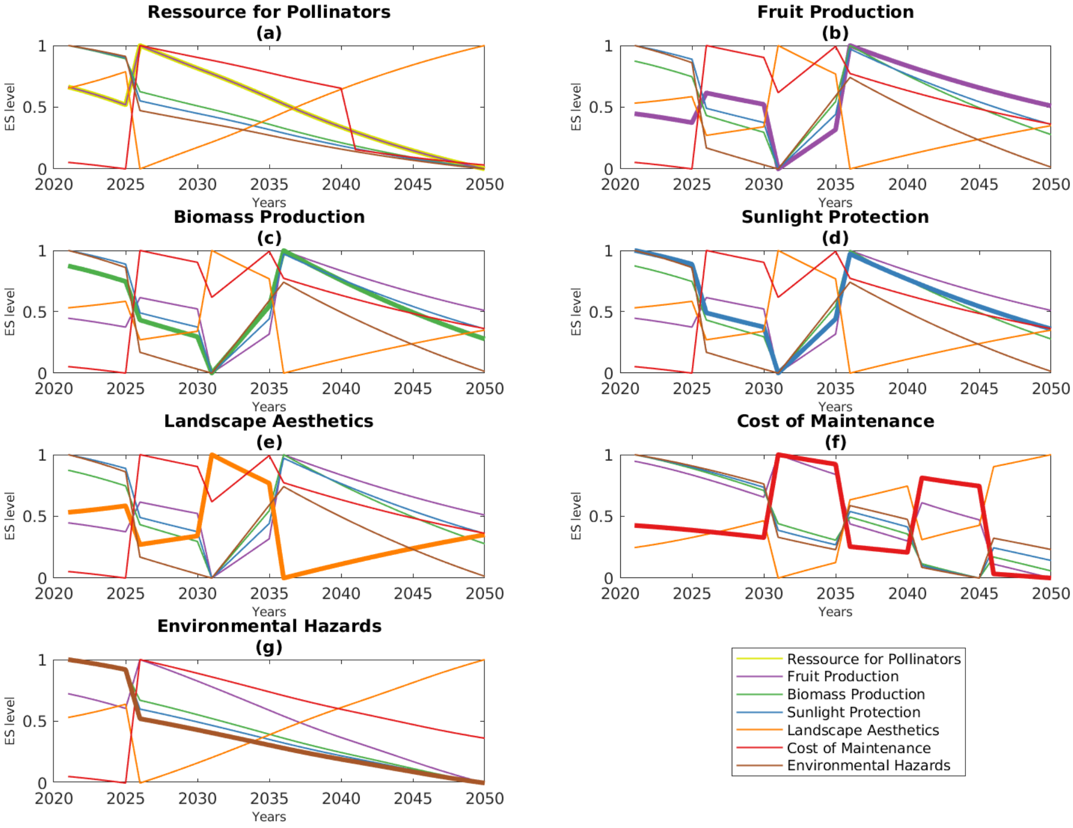


**Figure S5.1.2.** Temporal dynamics of ecosystem (dis)services (ES) delivered by tall hedges with low diversity, extracted from the optimal DAPP map presented in figure 9a for a peri-urban SES with climatic stress level 1. Panels show the dynamics of seven ES as a response to the selection of the most optimal adaptation pathway that aim at minimizing or maximizing a) pollination, b) fruit production, c) biomass production, d) sunlight protection, e) landscape aesthetics, f) cost of maintenance, g) environmental hazards.

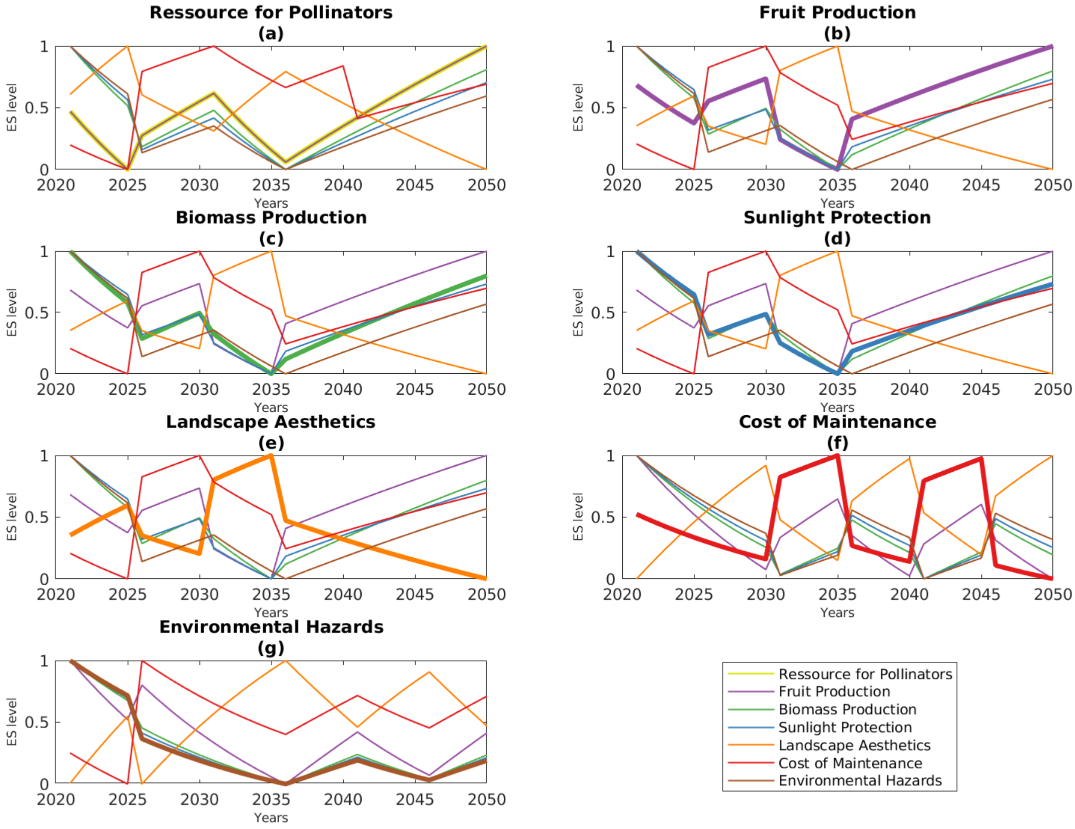


**Figure S5.1.3.** Temporal dynamics of ecosystem (dis)services (ES) delivered by small hedges with high diversity, extracted from the optimal DAPP map presented in figure 9a for a peri-urban SES with climatic stress level 1. Panels show the dynamics of seven ES as a response to the selection of the most optimal adaptation pathway that aim at minimizing or maximizing a) pollination, b) fruit production, c) biomass production, d) sunlight protection, e) landscape aesthetics, f) cost of maintenance, g) environmental hazards.

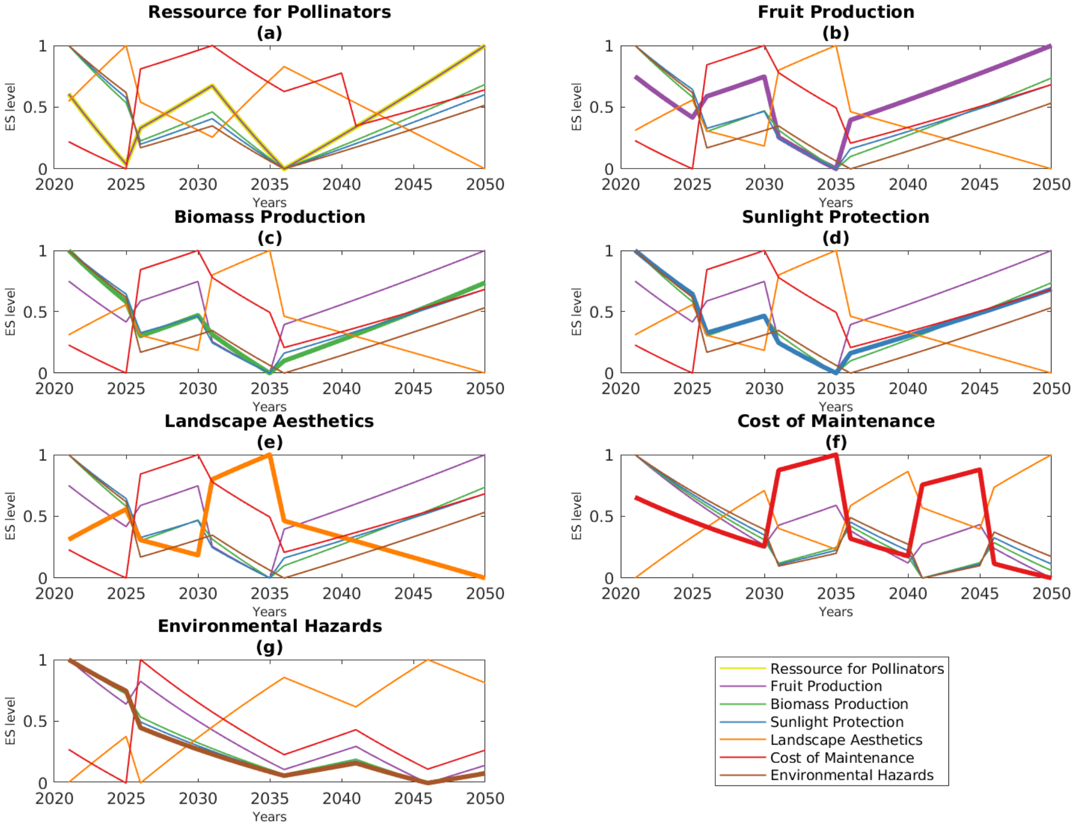


Figure S5.1.4. Temporal dynamics of ecosystem (dis)services (ES) delivered by tall hedges with high diversity, extracted from the optimal DAPP map presented in figure 9a for a peri-urban SES with climatic stress level 1. Panels show the dynamics of seven ES as a response to the selection of the most optimal adaptation pathway that aim at minimizing or maximizing a) pollination, b) fruit production, c) biomass production, d) sunlight protection, e) landscape aesthetics, f) cost of maintenance, g) environmental hazards.

## S5.2. Results for archetypal scenarios of adaptation pathways where the CCA and KCA never change during 30 years.

### Scenario $CCA_1$ with collective-choice arrangement KCA1 (see table S1)

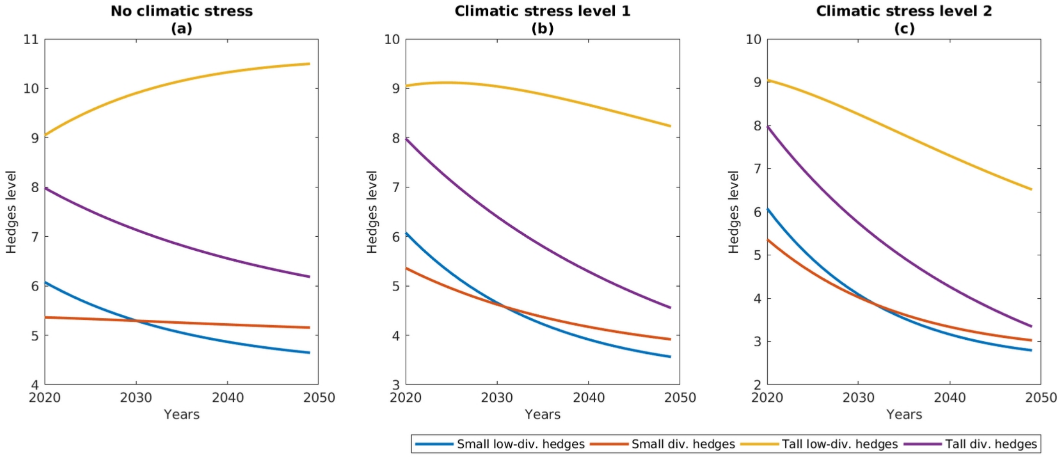


**Figure S5.2.1**. Evolution of the relative proportion of the four types of hedgerows under a 30 years scenario of constitutional-choice $CCA_1$, for three levels of climatic stress, for two types of social-ecological systems (SES).

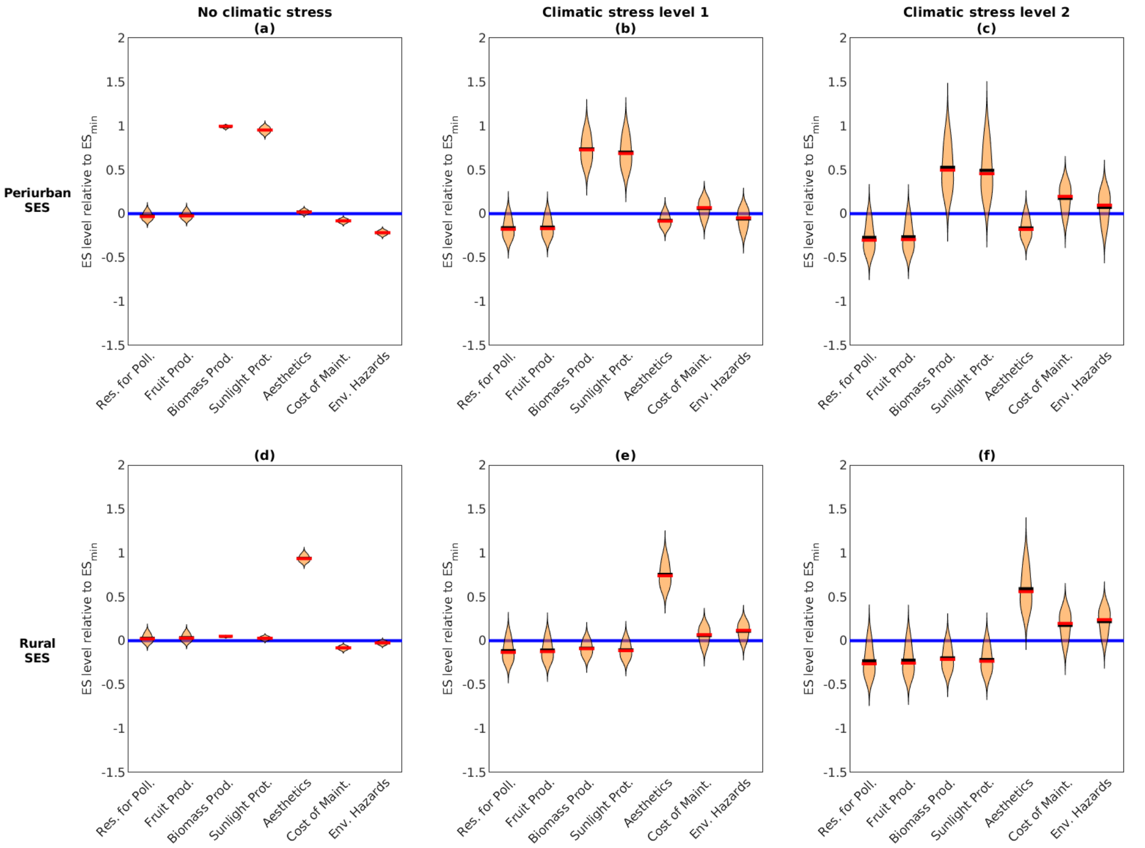


**Figure S5.2.2**. ES provisioning under a 30 years constitutional-choice arrangement $CCA_1$ and collective-choice arrangement $KCA_1$ for three climate stress levels and two social-ecological systems (SES). See explanations in figure 10 for explanations on how to read the figure

## Scenario $CCA_2$ with collective-choice arrangement $KCA_2$ (see table S1)

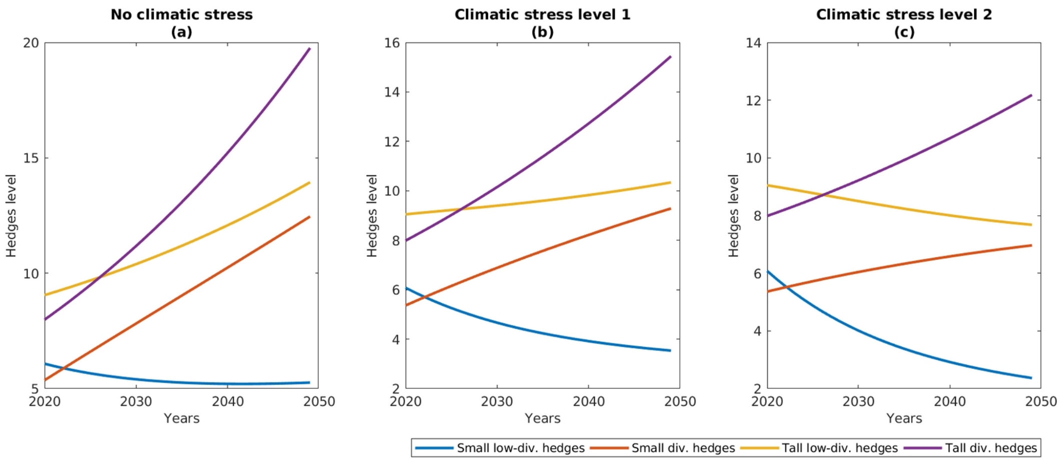


**Figure S5.2.3.** Evolution of the relative proportion of the four types of hedgerows under a 30 years scenario of constitutional-choice $CCA_2$ and collective-choice arrangement $KCA_2$, for three levels of climatic stress and two types of social-ecological systems (SES).

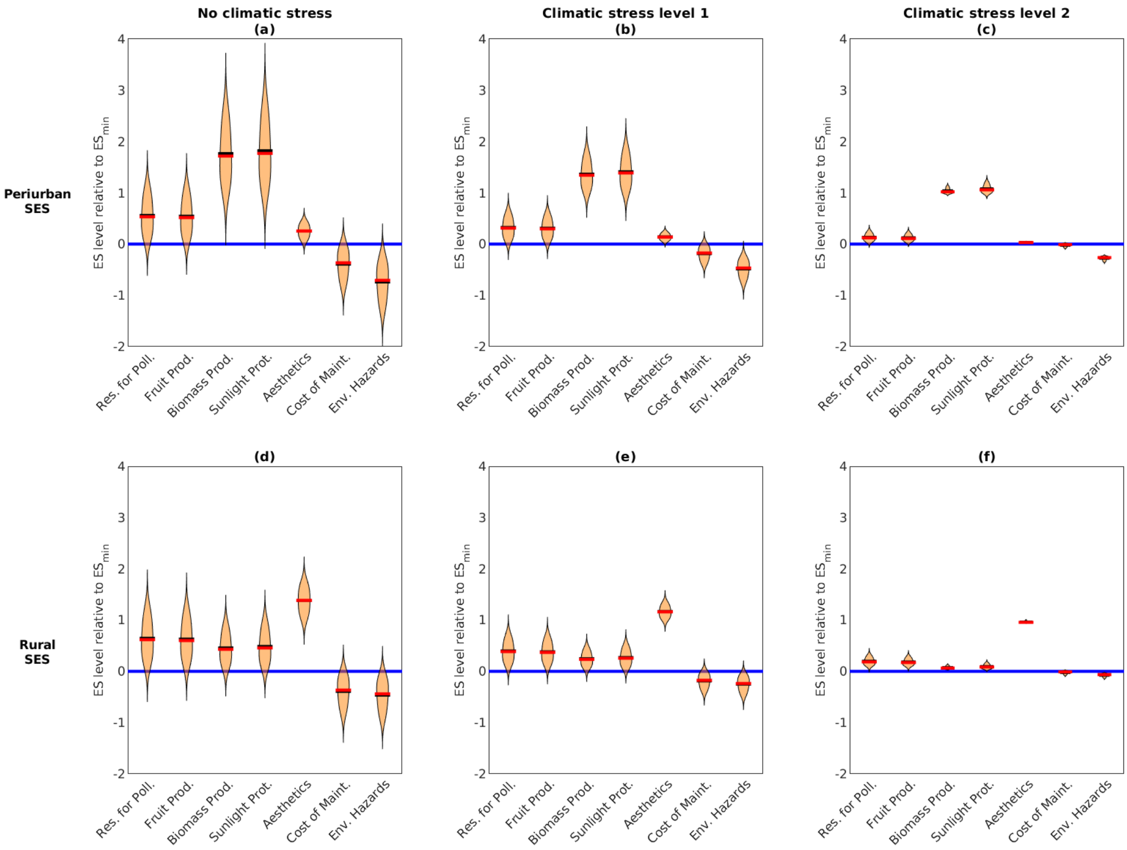


**Figure S4.2.4.** ES provisioning under a 30 years scenario of constitutional-choice $CCA_2$ and collective-choice arrangement $KCA_2$, for three climate stress levels and two social-ecological systems (SES). See explanations in figure 10 for explanations on how to read the figure

# Scenario CCA-B with collective-choice arrangement KCA-2  (see table S1)

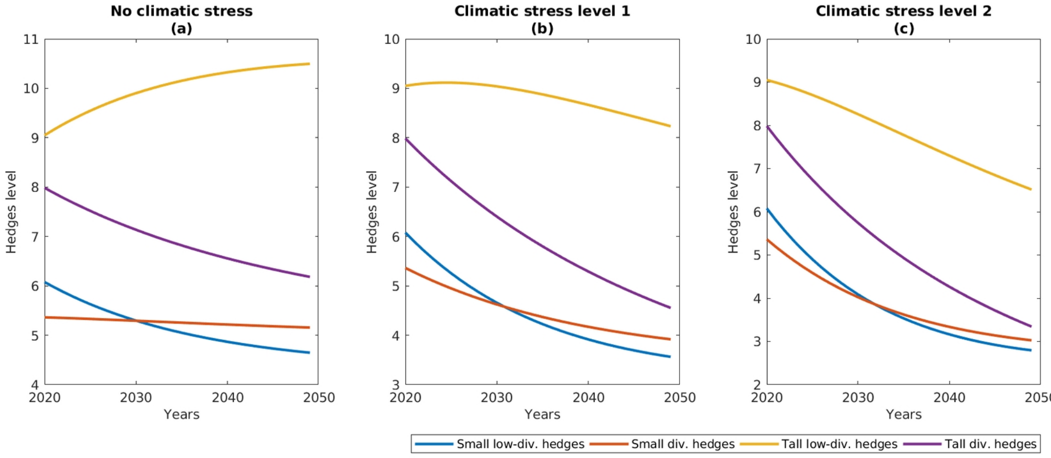


**Figure S4.2.5**. Evolution of the relative proportion of the four types of hedgerows under a 30 years scenario of constitutional-choice $CCA_2$ and collective-choice arrangement $KCA_3$ and three levels of climatic stress, for two types of social-ecological systems (SES).

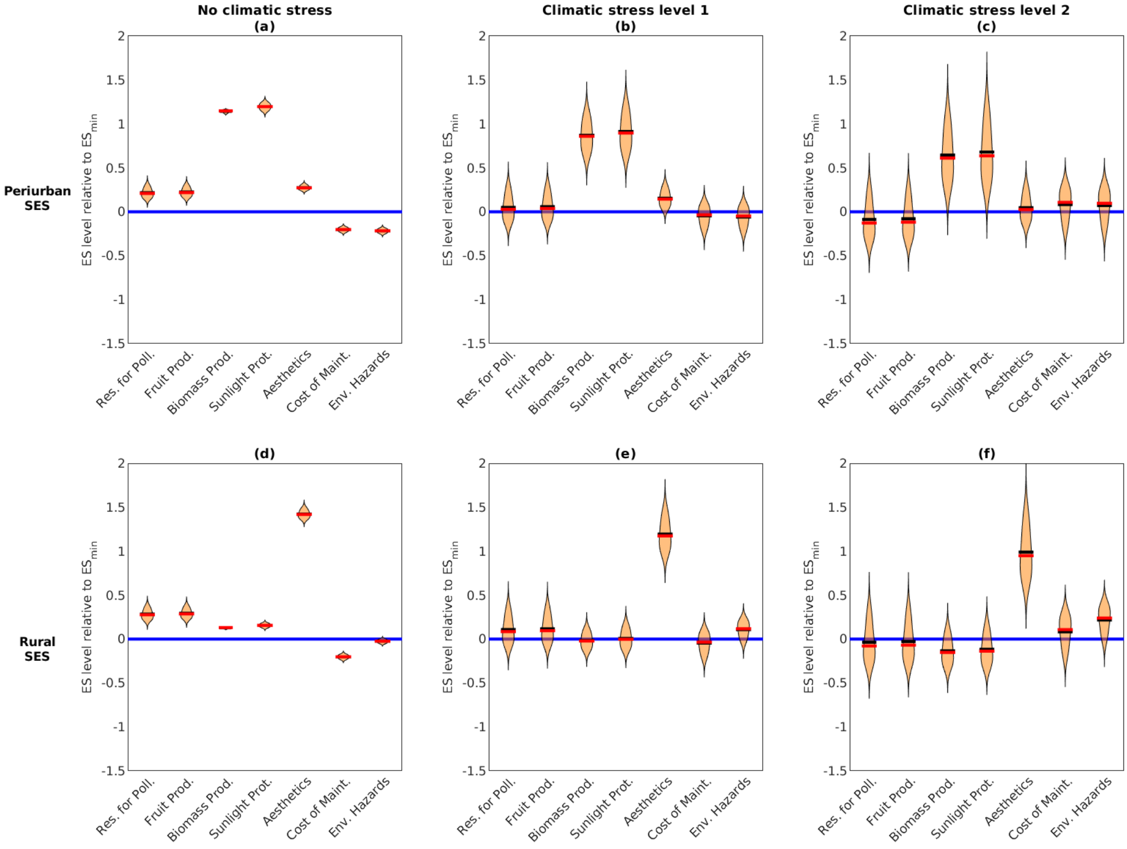


**Figure S4.2.6**. ES provisioning under a 30 years scenario of constitutional-choice CCA-B and collective-choice arrangement KCA-2, for three climate stress levels and two social-ecological systems (SES). See explanations in figure 10 for explanations on how to read the figure.

# Constitutional choice arrangement $CCA_3$ with collective-choice arrangement $KCA_4$

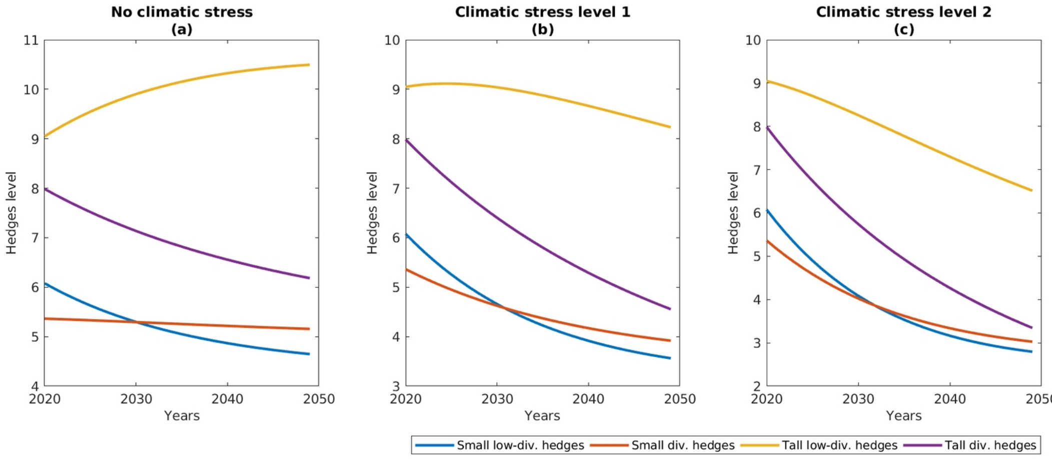


**Figure S5.2.7.** Evolution of the relative proportion of the four types of hedgerows under a 30 years scenario of constitutional-choice $CCA_3$ and collective-choice arrangement $KCA_4$ and three levels of climatic stress, for two types of social-ecological systems (SES).

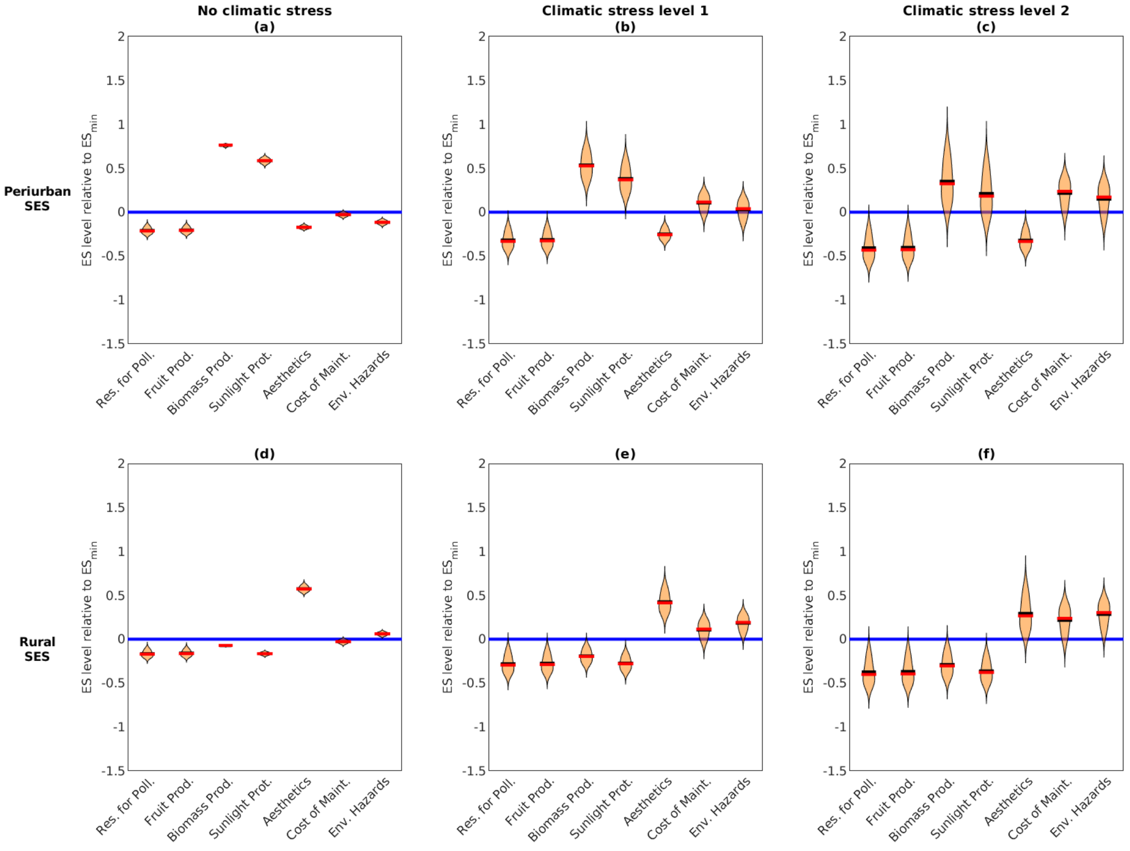


**Figure S5.2.8.** ES provisioning under a 30 years scenario of constitutional-choice $CCA_3$ and collective-choice arrangement $KCA_4$, for three climate stress levels and two social-ecological systems (SES). See explanations in figure 10 for explanations on how to read the figure.

## Constitutional choice arrangement $CCA_3$ with collective-choice arrangement $KCA_5$

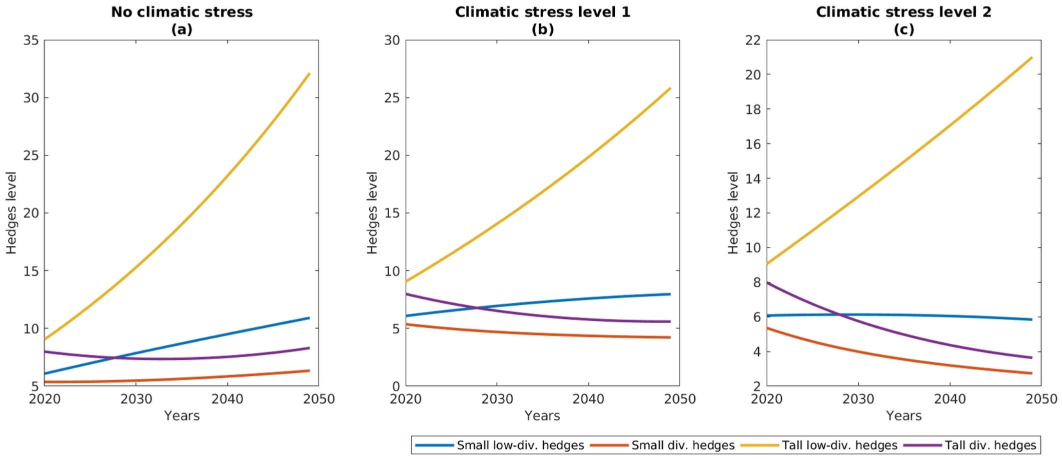


**Figure S5.2.9.** Evolution of the relative proportion of the four types of hedgerows under a 30 years scenario of constitutional-choice $CCA_3$ and collective-choice arrangement $KCA_5$ and three levels of climatic stress, for two types of social-ecological systems (SES).

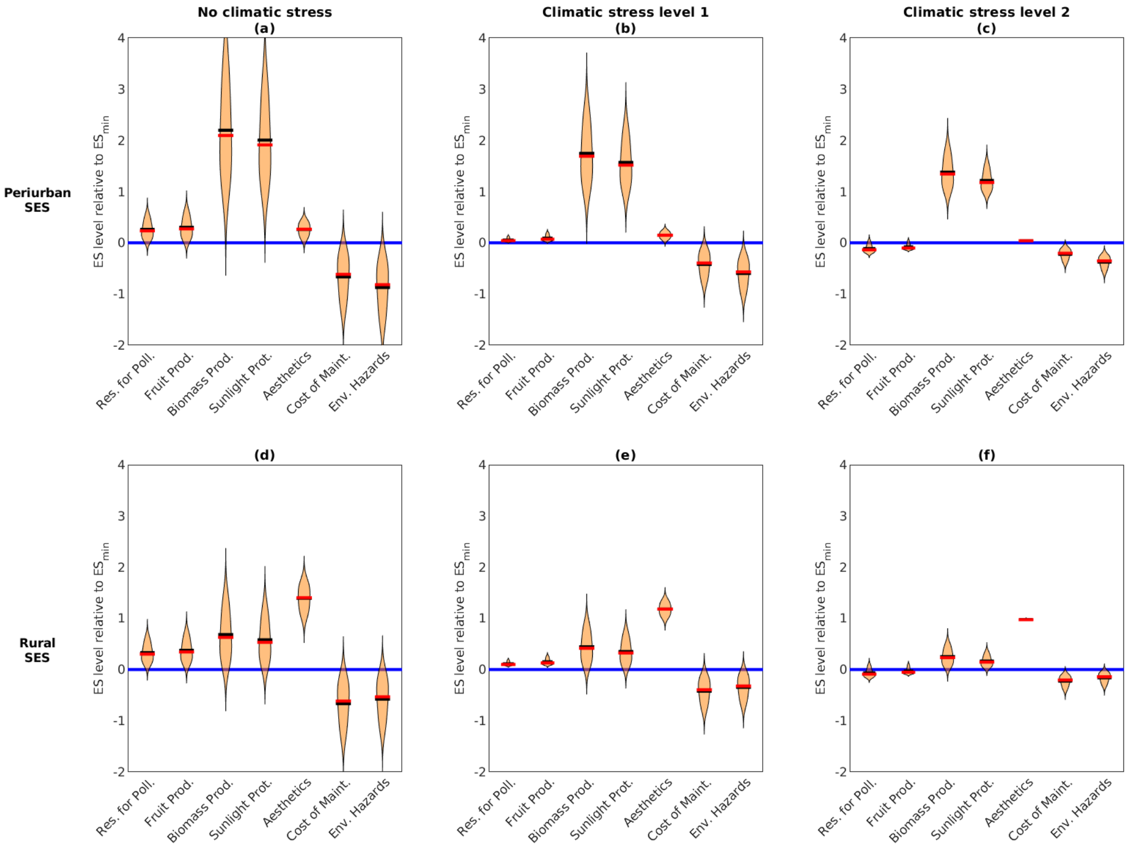


**Figure S5.2.10.** ES provisioning under a 30 years scenario of constitutional-choice $CCA_3$ and collective-choice arrangement $KCA_5$, for three climate stress levels and two social-ecological systems (SES). See explanations in figure 10 for on how to read the figure.

## Constitutional choice arrangement $CCA_3$ with collective-choice arrangement $KCA_6$

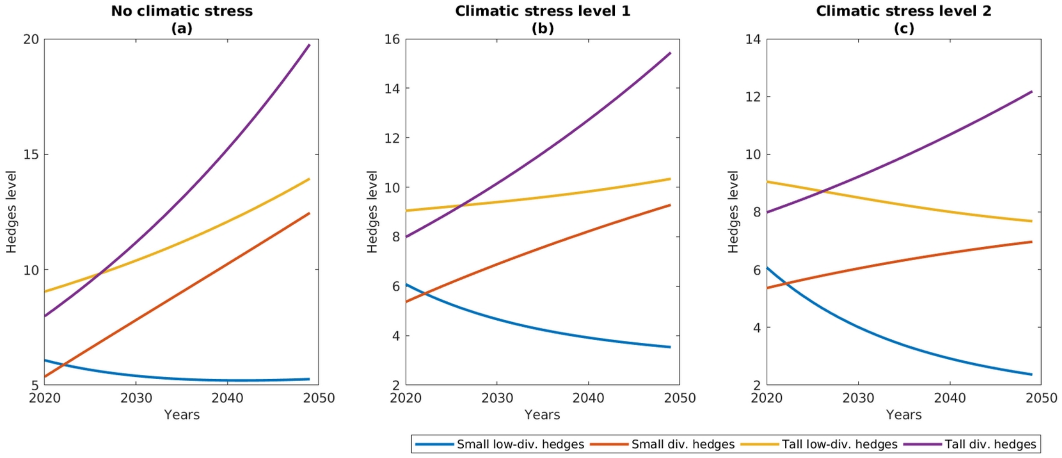


**Figure S5.2.11.** Evolution of the relative proportion of the four types of hedgerows under a 30 years scenario of constitutional-choice $CCA_3$ and collective-choice arrangement $KCA_6$ and three levels of climatic stress, for two types of social-ecological systems (SES).

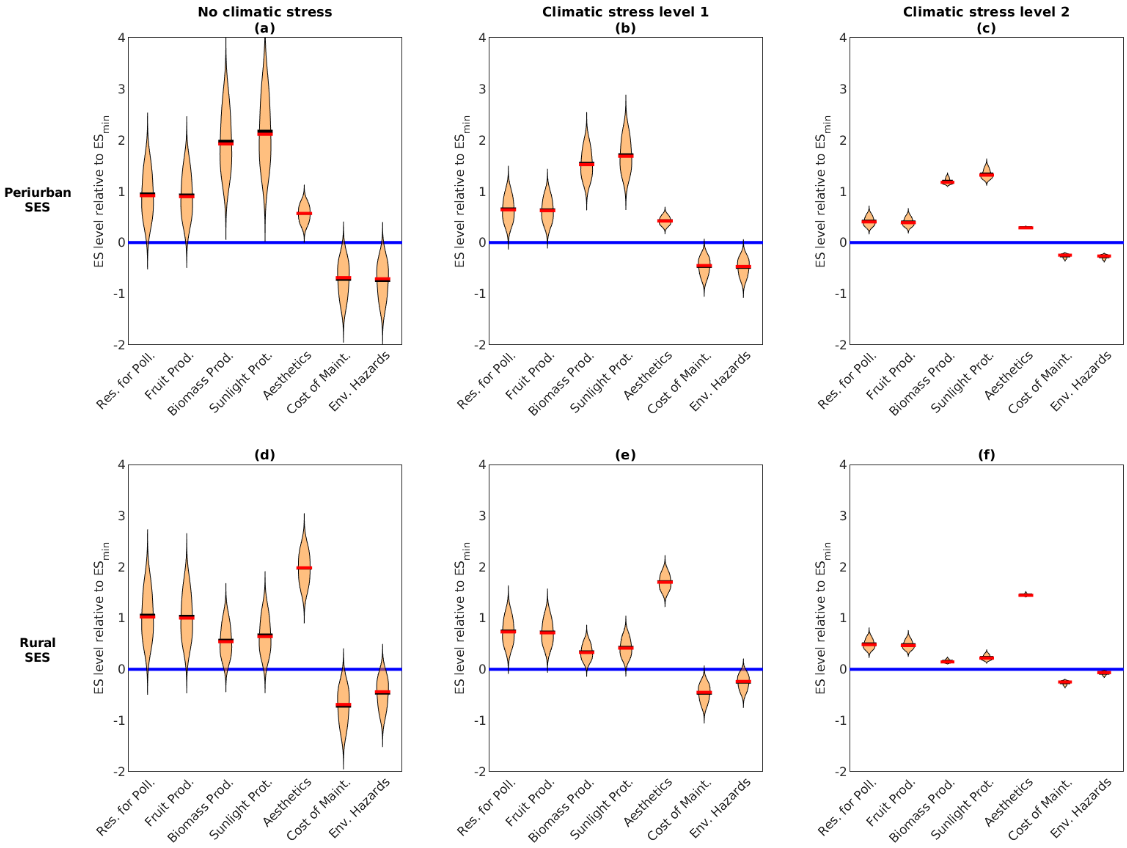


**Figure S5.2.12.** ES provisioning under a 30 years scenario of constitutional-choice $CCA_3$ and collective-choice arrangement $KCA_6$, for three climate stress levels and two social-ecological systems (SES). See explanations in figure 10 for explanations on how to read the figure.

## Constitutional choice arrangement $CCA_3$ with collective-choice arrangement $KCA_7$

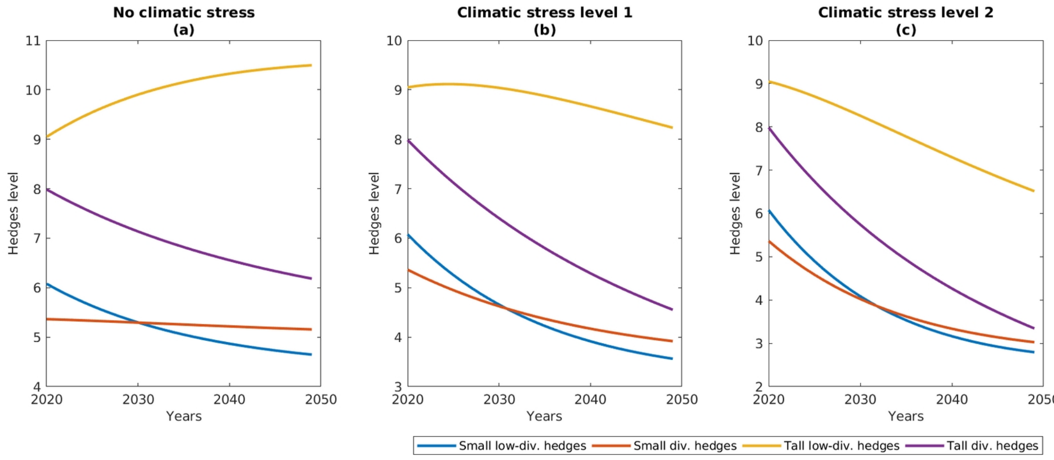


**Figure S5.2.13.** Evolution of the relative proportion of the four types of hedgerows under a 30 years scenario of constitutional-choice $CCA_3$ and collective-choice arrangement $KCA_7$ and three levels of climatic stress and two types of social-ecological systems (SES).

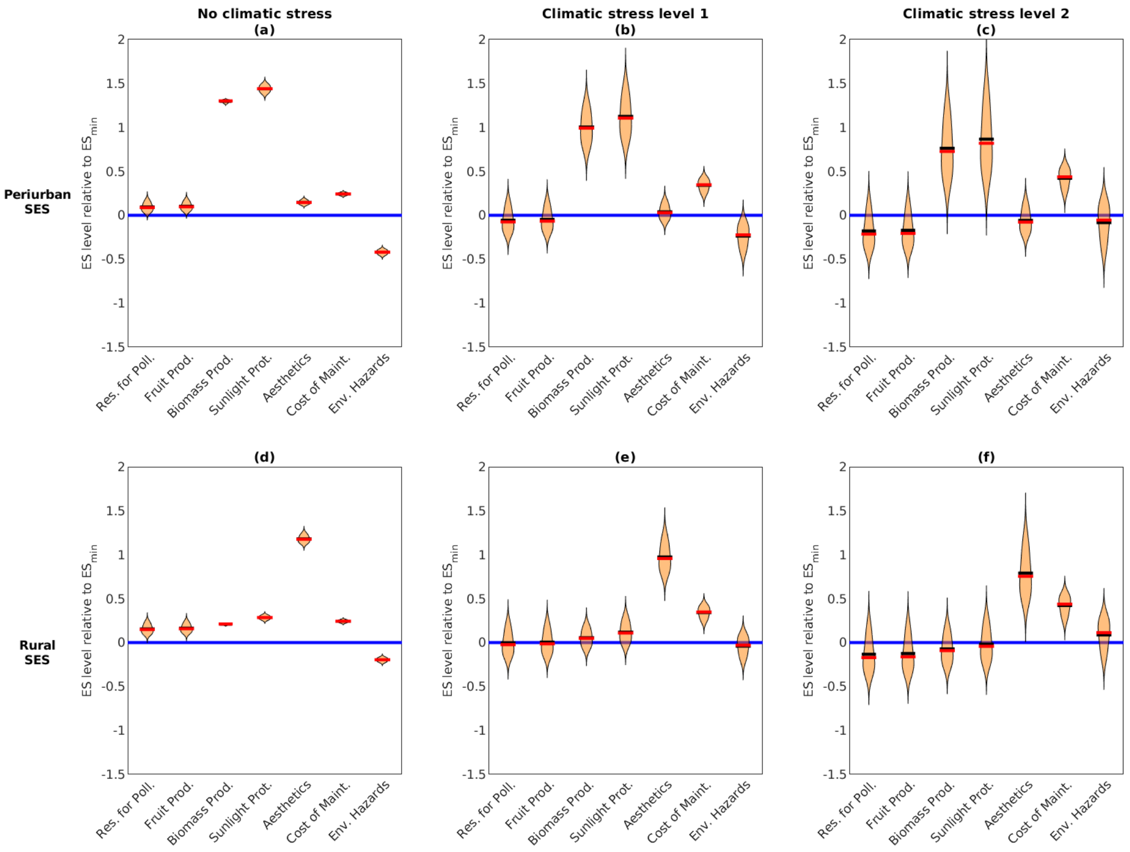


**Figure S5.2.14.** ES provisioning under a 30 years scenario of constitutional-choice $CCA_3$ and collective-choice arrangement $KCA_7$, for three climate stress levels and two social-ecological systems (SES). See explanations in figure 10 for explanations on how to read the figure.

## Constitutional choice arrangement CCA$_4$ with collective-choice arrangement KCA$_8$

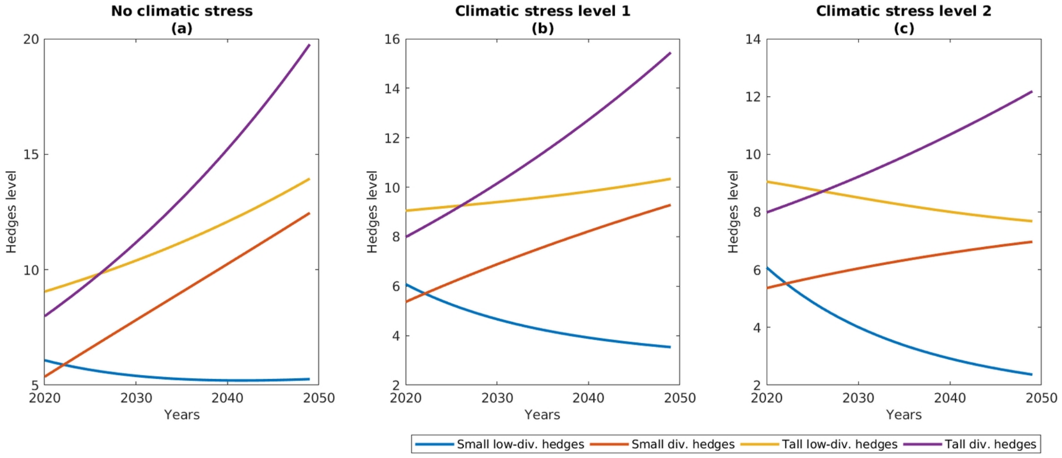


**Figure S5.2.15.** Evolution of the relative proportion of the four types of hedgerows under a 30 years scenario of constitutional-choice CCA$_4$ and collective-choice arrangement KCA$_8$ and three levels of climatic stress and two social-ecological systems (SES).

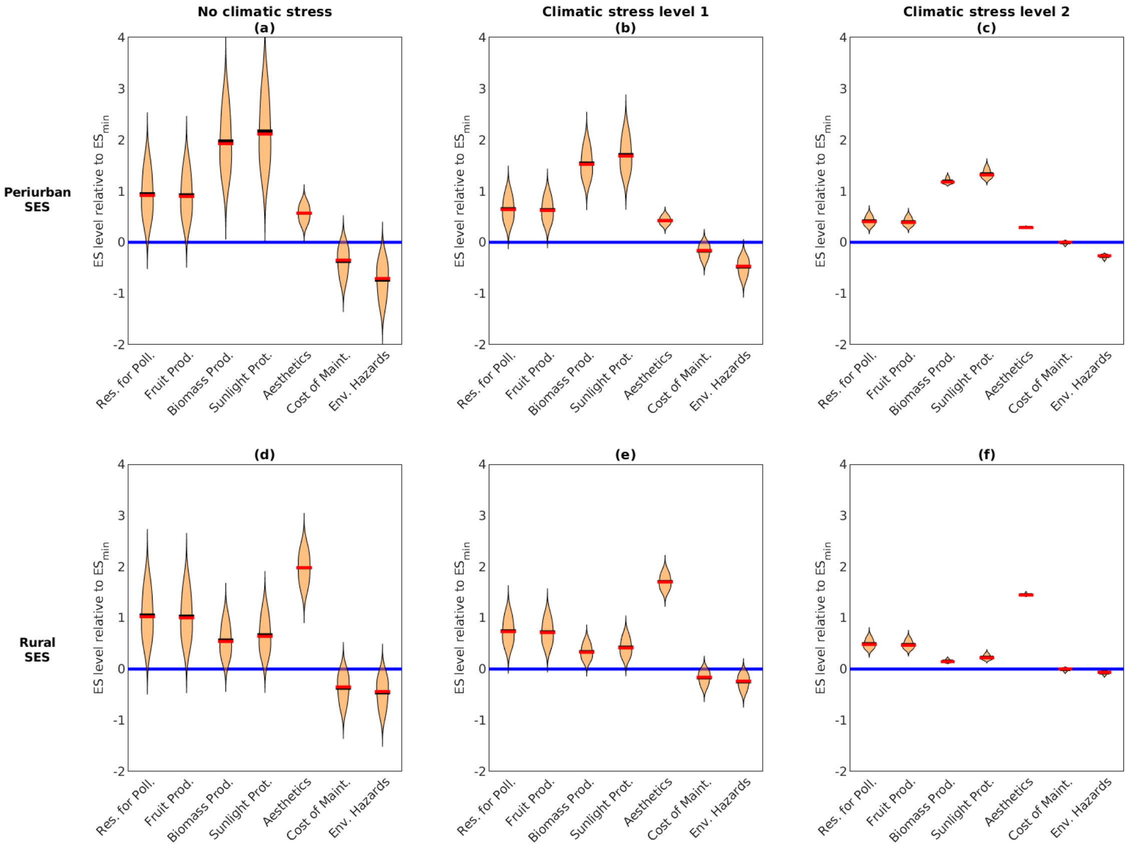


**Figure S5.2.16.** ES provisioning under a 30 years scenario of constitutional-choice CCA$_4$ and collective-choice arrangement KCA$_8$, for three climate stress levels and two social-ecological systems (SES). See explanations in figure 10 for explanations on how to read the figure.

## Constitutional choice arrangement $CCA_4$ with collective-choice arrangement $KCA_9$

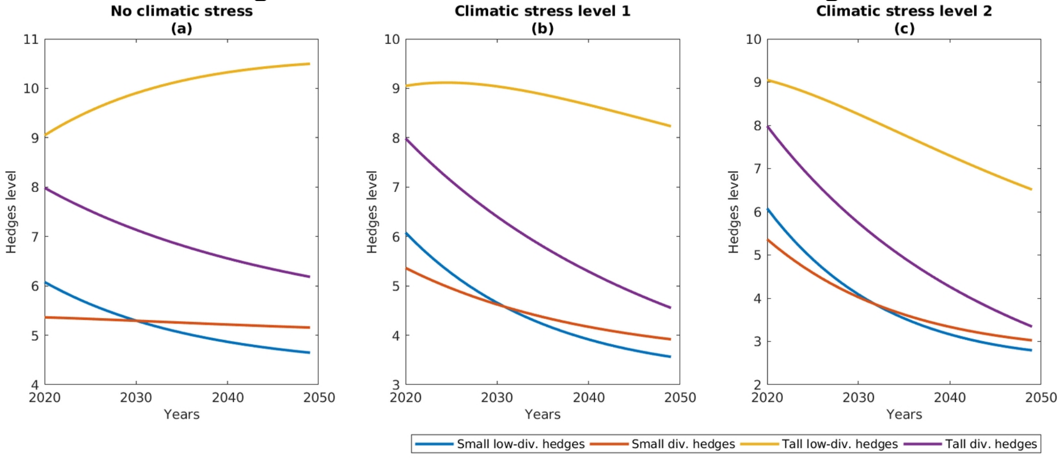


**Figure S5.2.17.** Evolution of the relative proportion of the four types of hedgerows under a 30 years scenario of constitutional-choice $CCA_4$ and collective-choice arrangement $KCA_9$ and three levels of climatic stress and two types of social-ecological systems.

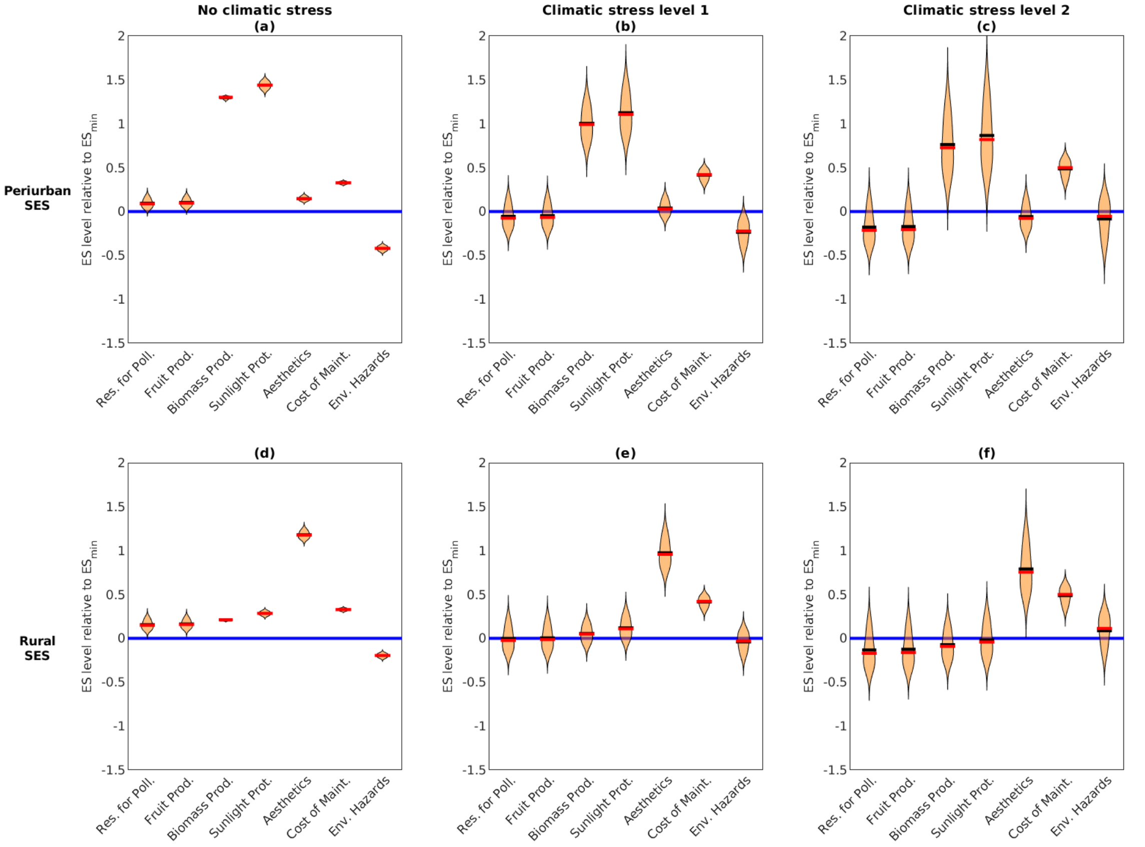


**Figure S5.2.18.** ES provisioning under a 30 years scenario of constitutional-choice $CCA_4$ and collective-choice arrangement $KCA_9$, for three climate stress levels and two social-ecological systems (peri-urban and rural). See explanations in figure 10 for explanations on how to read the figure.
